# CDT1 inhibits CMG helicase in early S phase to separate origin licensing from DNA synthesis

**DOI:** 10.1016/j.molcel.2022.12.004

**Published:** 2023-01-05

**Authors:** Nalin Ratnayeke, Yasemin Baris, Mingyu Chung, Joseph T.P. Yeeles, Tobias Meyer

**Affiliations:** 1 Department of Chemical and Systems Biology, Stanford University School of Medicine, Stanford, CA 94305, USA; 2 Department of Cell and Developmental Biology, Weill Cornell Medical College, New York, NY 10065, USA; 3 Laboratory of Molecular Biology, Medical Research Council, Cambridge CB2 0QH, UK

## Abstract

Human cells license tens of thousands of origins of replication in G1 and then must stop all licensing before DNA synthesis in S phase to prevent re-replication and genome instability that ensue when an origin is licensed on replicated DNA. However, the E3 ubiquitin ligase CRL4^Cdt2^ only starts to degrade the licensing factor CDT1 after origin firing, raising the question of how cells prevent re-replication before CDT1 is fully degraded. Here, using quantitative microscopy and *in-vitro*-reconstituted human DNA replication, we show that CDT1 inhibits DNA synthesis during an overlap period when CDT1 is still present after origin firing. CDT1 inhibits DNA synthesis by suppressing CMG helicase at replication forks, and DNA synthesis commences once CDT1 is degraded. Thus, in contrast to the prevailing model that human cells prevent re-replication by strictly separating licensing from firing, licensing and firing overlap, and cells instead separate licensing from DNA synthesis.

## Graphical abstract

**Graphical abstract:**
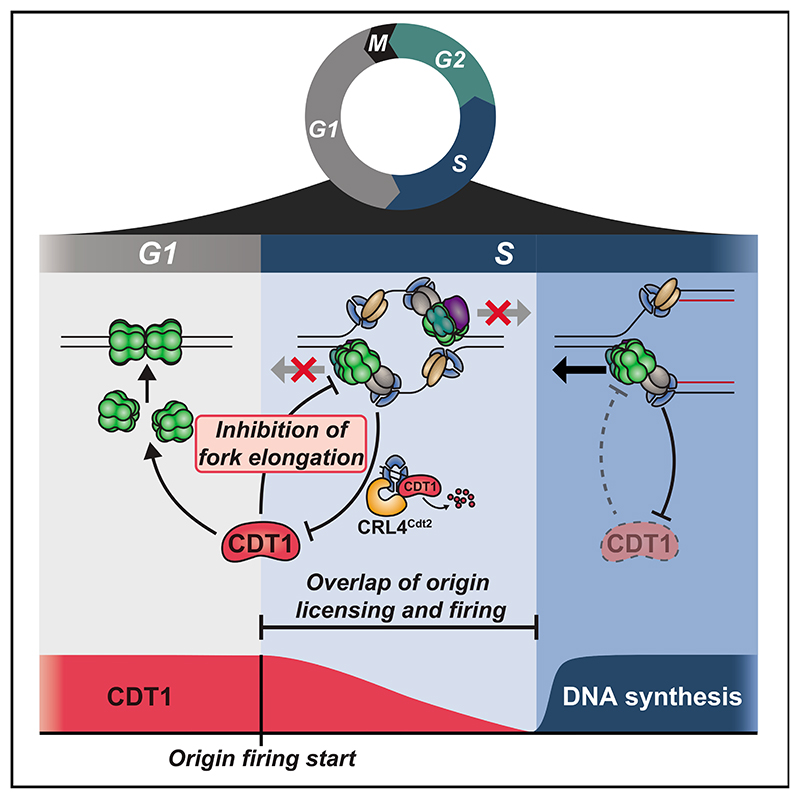

## Introduction

To duplicate their genome precisely once, eukaryotic cells are thought to strictly separate DNA replication into a period of replication origin licensing and a period of origin firing.^1–3^ During licensing in G1 phase, cells demarcate future sites of DNA synthesis by loading inactive minichromosome maintenance (MCM)2–7 helicases onto origins of replication. At the start of S phase, cells begin origin firing, whereby replication factors are recruited to the inactive helicases to form active CDC45-MCM2–7-GINS (CMG) helicases and replication forks that duplicate DNA. Critically, it is thought that origin licensing must be strictly separated in time from origin firing to avoid re-replication, which occurs when newly synthesized DNA is re-licensed and replicated again within the same cell cycle.^1,3,4^ Avoiding re-replication is crucial for maintaining genome stability, and failure to do so results in gene amplification, DNA damage, oncogenesis, and cell death.^1,5^

The G1/S transition is a particularly vulnerable period in the cell cycle when cells must simultaneously inactivate licensing and initiate origin firing. In humans and other vertebrates, avoiding re-replication is critically dependent on the repression of the essential licensing factor CDT1 from the start of S phase through anaphase.^5^ Activation of CDT1 during this period is sufficient to trigger re-replication.^6–9^ CDT1 activity can be repressed by CDT1 degradation mediated by cullin-RING E3 ubiquitin ligases CRL4^Cdt2^ and SCF^Skp2^ (also known as CRL1^Skp2^), as well as by GMNN (geminin) binding to CDT1 and hyperphosphorylation of CDT1 by cyclin A-CDK1.^5,10^ Both geminin and cyclin A are degraded during G1 by E3 ubiquitin ligase APC/C^Cdh1^ and only begin to accumulate after APC/C^Cdh1^ inactivation at the start of S phase,^11–13^ while SCF^Skp2^-mediated CDT1 degradation is thought to only begin in mid-S phase.^14,15^ These findings suggest that degradation of CDT1 by CRL4^Cdt2^ alone is responsible for preventing re-replication in early S phase.

However, the exclusive role of CRL4^Cdt2^ in inactivating CDT1 at the start of S phase poses a conundrum: for CRL4^Cdt2^ to ubiquitinate and degrade CDT1 in S phase, CDT1 must first bind to the replication fork component proliferating cell nuclear antigen (PCNA),^16,17^ and therefore, CDT1 degradation can only start after origins have already fired. This regulation would result in an overlap period in early S phase when cells fire origins and could still license DNA before CDT1 is fully degraded.^1,4,17^ Since it is expected that fired origins immediately synthesize DNA, this overlap period would be susceptible to re-licensing and re-replication. Even a small overlap could cause re-replication, since human diploid cells contain approximately 6 Gb pairs of DNA and replicate DNA at thousands of sites simultaneously, each of which provides an opportunity for re-replication.

Here, using a single-cell-microscopy-based analysis of human cells, we show that there is an overlap period in early S phase that lasts approximately 30 min, during which active CDT1 is present together with fired origins. Strikingly, using single-cell microscopy and *in-vitro*-reconstituted human DNA synthesis, we show that in addition to licensing origins in G1, CDT1 has an unexpected second role of inhibiting CMG helicase at replication forks during this overlap. Thus, cells can fire origins while inhibiting DNA synthesis, and this inhibition is only relieved once CDT1 is fully degraded. In this way, cells restrict the amount of synthesized DNA produced in the presence of CDT1 to deter re-replication. Conceptually, our study suggests that instead of temporally separating licensing and firing of origins in early S phase, human cells safeguard genome integrity by using CDT1-mediated CMG helicase inhibition to separate licensing and DNA synthesis.

## Results

### Active CDT1 is present together with fired origins in early S phase

**Figure 1:**
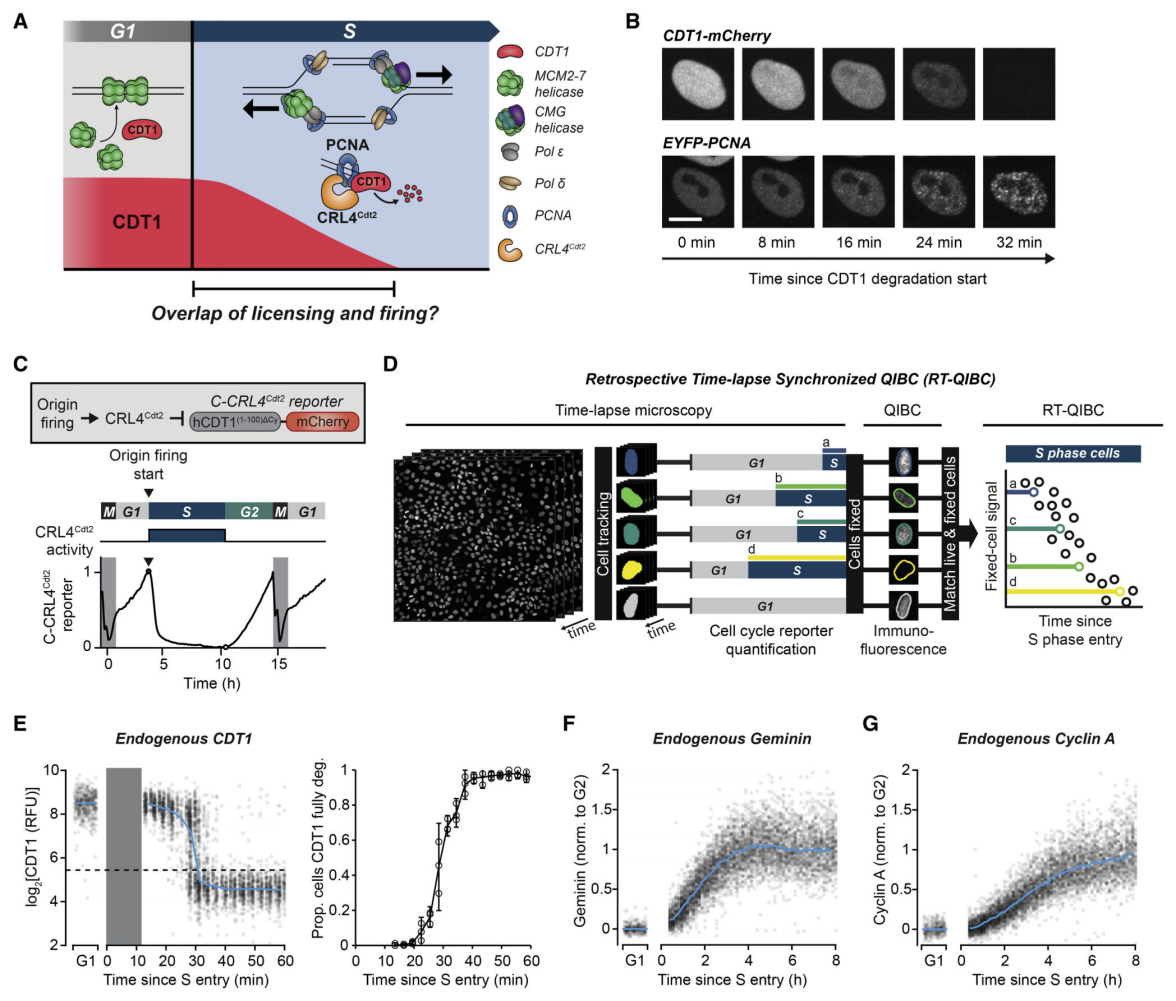
Active CDT1 is present together with fired origins in early S phase (A) Predicted overlap between CDT1 and origin firing in early S phase. (B) MCF10A cells expressing EYFP-PCNA and doxycycline-inducible CDT1-mCherry, induced 6 h before imaging. Representative of 54 cells. Scale bars, 10 μm. Quantification in Figures S1A–S1C and expression in Figure S1F. (C) Top: live-cell reporter of CRL4^Cdt2^ activity. Bottom: example trace of C-CRL4^Cdt2^ reporter in a single MCF10A cell. Reporter is degraded at S entry. (D) Diagram of retrospective time-lapse synchronized QIBC (RT-QIBC). Time-lapse microscopy of H2B-mTurquioise and QIBC of CDT1 immunofluorescence (IF). (E–G) RT-QIBC aligned to S entry (C-CRL4^Cdt2^ reporter degradation). G1 cells are 1–2 h after anaphase. Solid blue curves are median value. Representative of 3 independent experiments. (E) Left:CDT1 IF(n = 3,710 Scells, 500G1 cells). Dashed line is threshold for fully degraded CDT1. Gray bar is period that is not observed due to the requirement of 12 min of reporter degradation to identify S entry. Right: quantification of left. Proportion of cells with fully degraded CDT1 over time within 3 min bins for 3 experiments (n ≥ 36 cells per point). Error bars are 95% confidence intervals. (F) Geminin IF (n = 13,262 S cells, 300 G1 cells). (G) Cyclin AIF(n = 13,262 S cells, 300 G1 cells). See also Figures S1 and S2.

To determine whether CDT1 protein is present together with fired origins of replication (Figure 1A), we monitored the degradation of a doxycycline (Dox)-inducible CDT1-mCherry fusion protein in live MCF10A cells (a non-transformed human epithelial cell line). We simultaneously imaged EYFP-tagged PCNA, which forms foci at sites of origin firing and DNA synthesis.^18,19^ In line with previous studies,^14,20^ CDT1-mCherry degradation at S-phase start was coupled to the formation of PCNA foci (Figures 1B and S1A–S1C). Mutant analysis of CDT1 degrons confirmed that CDT1-mCherry degradation at S-phase start is mediated by CRL4^Cdt2^, while SCF^Skp2^ does not contribute until mid-S phase (Figures S1D–S1F). There is approximately 30 min between the start and completion of CDT1-mCherry degradation (Figures S1A and S1B), suggesting that there is an extended overlap period in early S phase when CDT1 is present together with fired origins.

To determine whether endogenous CDT1 similarly overlaps with fired origins in early S phase, we combined live-cell microscopy of S-phase entry reporters with fixed-cell immunofluorescence (IF) microscopy of endogenous CDT1. To precisely measure S-phase entry in live cells, we imaged a component of the FUCCI(CA) cell-cycle reporter system, human CDT1^(1–100)ΔCy^, which is rapidly degraded by CRL4^Cdt2^ in response to origin firing at S-phase start^15^ (Figure 1C). We used this reporter in its original N-terminal mCherry-tagged orientation (referred to here as N-CRL4^Cdt2^ reporter) and also created a C-terminal tagged reporter (C-CRL4^Cdt2^ reporter), which is degraded with slightly faster kinetics and further facilitates precise measurement of S-phase entry (see STAR Methods for discussion of reporters and Figures 1C, S1G, and S1H). We use the term S-phase entry to refer to the start of origin firing, which is marked by the degradation of the CRL4^Cdt2^ reporters (Figure 1C).

We live-imaged thousands of asynchronously proliferating cells and detected S-phase entry using automated time-lapse analysis of the C-CRL4^Cdt2^ reporter. Cells were immediately fixed after live-cell imaging and stained for endogenous CDT1. Single-cell CDT1 levels were then measured by quantitative image-based cytometry (QIBC).^21^ Finally, we computationally matched each cell from the fixed-cell QIBC analysis to its S-phase entry time from live-cell measurements.^22–25^ This allowed us to analyze endogenous CDT1 levels as a function of time after S-phase entry (Figure 1D). We refer to this combined live and fixed-cell technique as retrospective time-lapse synchronized QIBC (RT-QIBC).

Based on RT-QIBC of endogenous CDT1 IF staining in asynchronously cycling cells, endogenous CDT1 takes approximately 30 min to degrade following the start of S phase (Figure 1E). Control experiments showed that the start of S phase, as measured by the C-CRL4^Cdt2^ reporter, coincided with the appearance of chromatin-bound PCNA (Figure S1I), confirming that origins had fired. Thus, there is an overlap period after the start of S phase where endogenous CDT1 is present together with fired origins.

During this overlap period, CDT1 could be active or could, at least in principle, be inhibited through binding by geminin or hyperphosphorylation by cyclin A-CDK1. However, analysis of an APC/C^Cdh1^ reporter showed that APC/C^Cdh1^, which degrades geminin and cyclin A, is only inactivated near the start of S phase and can in some cells be inactivated after the start of S phase (Figures S1G and S1H), in line with previous findings.^14,15^ Accordingly, we observed low levels of geminin and cyclin A in the first 30 min of S phase using RT-QIBC analysis (Figures 1F, 1G, and S2A–S2D). This result is consistent with previous studies showing that geminin and cyclin A contribute to CDT1 inhibition only later in S and G2 after they accumulate to high enough levels.^8,10^ Since cyclin E-CDK2 does not hyperphosphorylate CDT1,^10^ and since cyclin A and geminin are low in early S phase, we conclude that the CDT1 present in early S phase is active.

Our analysis revealed that unsynchronized cells start S phase with high CDT1 and low geminin and cyclin A (Figures 1E–1G). In contrast, we found that cells synchronized in early S phase using aphidicolin or thymidine^26^ start S phase in a dramatically different state with low CDT1 and high levels of geminin and cy-clin A (Figures S2E and S2F). This is consistent with the mechanisms of aphidicolin and thymidine, which block DNA synthesis after origin firing but allow geminin and cyclin A accumulation, as well as Cdt1 degradation in response to chromatin-bound PCNA (Figure S2E and S2F). These findings argue that single-cell analysis of asynchronous cells is needed to study regulatory events in early S phase.

We conclude that early S phase is characterized by an approximately 30-min-long overlap period, during which replication origins have fired and CDT1 is still present and active. This presents a problem in the maintenance of genome integrity, as synthesized DNA at these fired origins would be susceptible to re-licensing by CDT1 and re-replication.

### DNA synthesis is inhibited in the presence of CDT1

**Figure 2:**
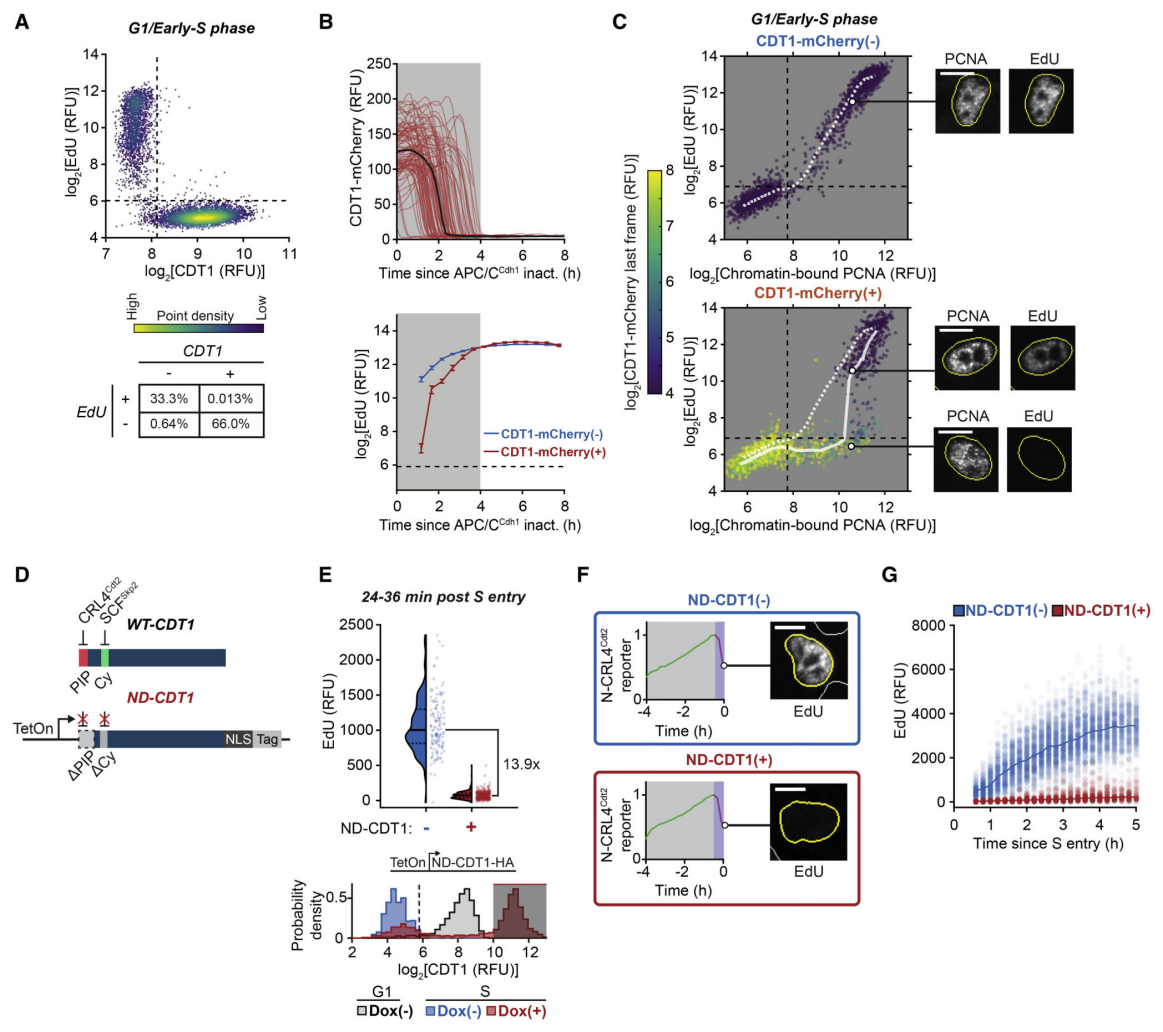
DNA synthesis is inhibited in the presence of CDT1 (A) CDT1 immunofluorescence (IF) and EdU in MCF10A cells in late G1 to early S (see STAR Methods). n = 7,486 cells, representative of 2 independent experiments. Percentage of cells in bottom table. (B and C) RT-QIBC in mitogen-released MCF10A cells with APC/C^Cdh1^ reporter and doxycycline (Dox)-inducible CDT1-mCherry. CDT1-mCherry expression in Figure S3C. (B) Top: live-cell CDT1-mCherry (100 traces). Black curve is median trace (n = 7,059 cells). Bottom: RT-QIBC of EdU in S cells (PCNA positive) aligned to APC/C^Cdh1^ inactivation. CDT1-mCherry(−): 18,367 cells, CDT1-mCherry(+): 6,970 cells. Error bars are mean and bootstrapped 95% confidence interval ofcells within 30 min bins (n ≥ 93 per bin). (C) Chromatin-bound PCNA and EdU in 2N DNA cells (G1/early S), colored by live-imaged CDT1-mCherry. CDT1-mCherry(−): n = 3,000 cells, CDT1-mCherry(+): 2,000 cells. White lines are median EdU. Representative cells shown.(D–F) RT-QIBC in MCF10A cells over expressing Dox-inducible non-degradable CDT1 (ND-CDT1), induced with Dox, live-imaged for 6 h and aligned to S entry (N-CRL4^Cdt2^ reporter degradation). Cells born within 1 h of Dox addition were analyzed. Representative of 2 independent experiments. (D) Diagram of ND-CDT1 construct. (E) Top: cells 24–36 min afterSentry. ND-CDT1(−): n = 141 cells, ND-CDT1(+): n = 400 cells. Bottom: CDT1 in G1 cells (1–2 h after mitosis) (gray, n = 2,191 cells) and in S cells (0.5–1 h after S entry) with either ND-CDT1 induced (red, n = 6,389 cells) or not induced (blue, n = 783 cells). Shaded area represents cells selected for ND-CDT1(+). (F) Representative N-CRL4^Cdt2^ reporter trace (magenta area represents time following S entry) and corresponding EdU image. (G) RT-QIBC in mitogen-released MCF10Acells. Cellsweretreated with control siRNA(same experiment as Figure 3F) and induced with Dox. ND-CDT1(+) cells selected based on gating in Figure S3H. ND-CDT1(−): n = 5,500 cells, ND-CDT1(+): n = 2,000 cells. Line is median value at each time point. Representative of 3 independent experiments. Dashed lines are negative staining. Dashed and solid lines in violin plots are IQR and median. Scale bars, 10 μm. See also Figure S3.

We next determined how much DNA is synthesized during the overlap period when origins have fired and CDT1 is still present. We measured the levels of CDT1 together with DNA synthesis, measured by the incorporation of 5-ethynyl-2'-deoxyuridine (EdU) into synthesized DNA in an 8 min period just before cell fixation. Strikingly, as cells transitioned from G1 to S phase, CDT1 and EdU staining were mutually exclusive (Figure 2A), arguing that while origins fire in the presence of CDT1, there is no detectable DNA synthesis occurring during the overlap period.

One possible explanation for the lack of EdU incorporation is that CDT1 itself suppresses DNA synthesis. To explore this possibility, we examined EdU incorporation by RT-QIBC cells expressing an APC/C^Cdh1^ reporter together with high levels of Dox-inducible CDT1-mCherry that remained present in early S phase for a longer period (Figures 2B and S3A–S3C). Markedly, these cells also exhibited a period of inhibited EdU incorporation after APC/C^Cdh1^ inactivation, which closely corresponded to the prolonged time during which CDT1-mCherry was still being degraded (Figure 2B, shaded area). In line with this interpretation, we identified a prominent population of cells with chromatin-bound PCNA but low EdU incorporation, corresponding to cells that had fired origins but had not yet fully degraded CDT1-mCherry (Figure 2C, lower-right quadrant).

Since CDT1-mCherry in the above experiments was still ultimately degraded in S phase, we more directly tested for a suppressive role of CDT1 by engineering a non-degradable mutant of CDT1 (ND-CDT1). Degradation of ND-CDT1 was prevented by both removing the PCNA-interacting protein (PIP) degron, which is required for PCNA binding and CRL4^Cdt2^-mediated degradation, and mutating the Cy motif, which is required for degradation by SCF^Skp2^(Figures 2D, S3D, and S3E).^15,16,20^ Markedly, similar to full-length CDT1, ND-CDT1 inhibited EdU incorporation (Figures 2E and 2F). Critically, inhibition of DNA synthesis did not prevent the firing of origins since the CRL4^Cdt2^ reporter was degraded similarly to control cells (Figures S3F and S3G). Furthermore, continued expression of ND-CDT1 persistently inhibited EdU incorporation and prevented progression through S phase as measured by DNA content (Figures 2G, S3H, and S3I). To ensure that ND-CDT1 did not interfere with G1 origin licensing, we measured chromatinbound MCM2 to quantify origin licensing^27,28^ and found no change (Figures S3J and S3K). ND-CDT1 also inhibited DNA synthesis in U2OS and HeLa cells, arguing that this inhibition is not cell-type specific and occurs in both non-transformed and transformed cells (Figures S3L and S3M).

**Figure 3:**
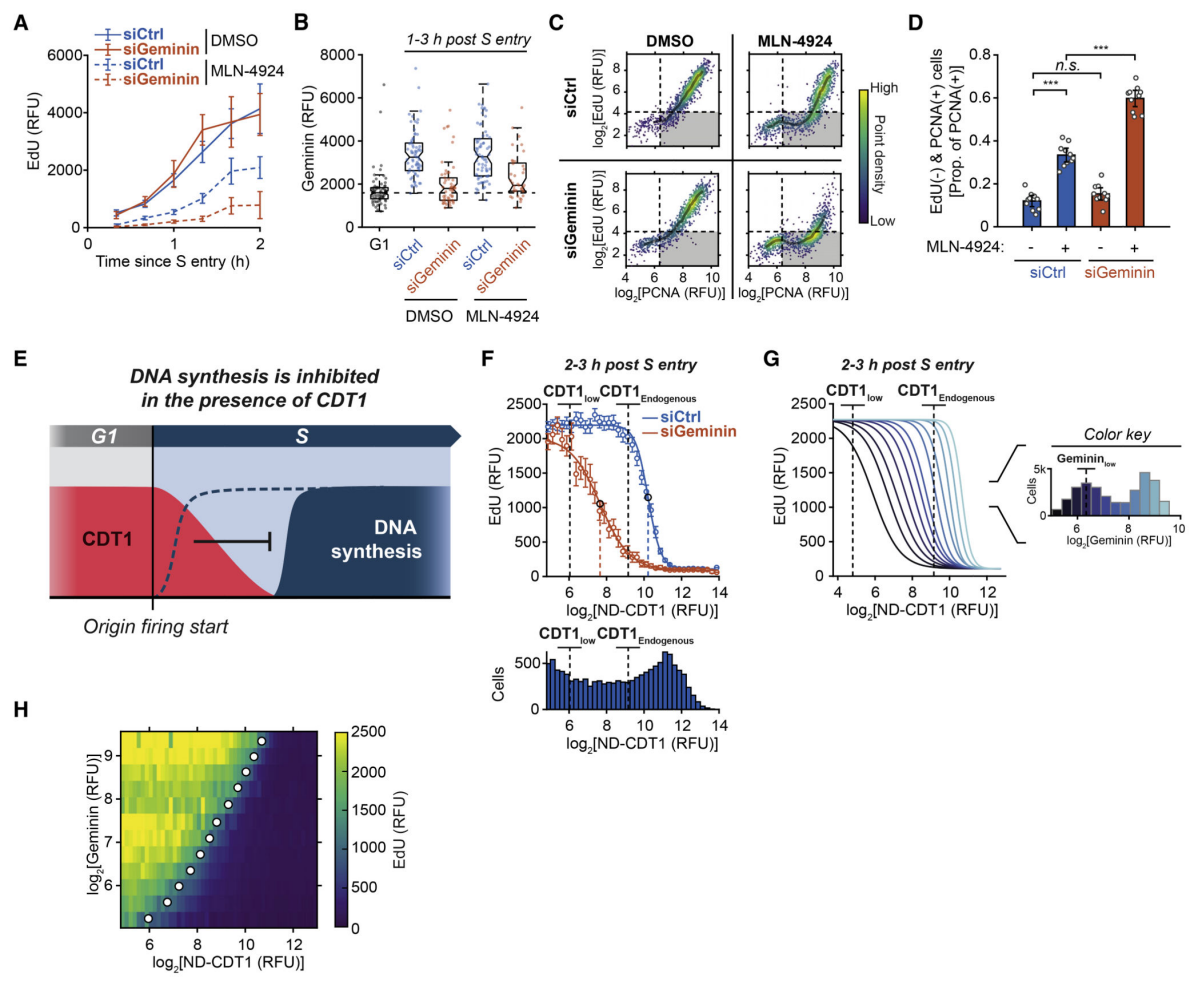
Endogenous CDT1 can inhibit DNA synthesis and is counteracted by geminin (A and B) RT-QIBC aligned to Sentry(PCNAfoci) in MCF10A cells treated with 2 μM MLN-4924 for 3.5 h during live-cell imaging. Representative of 2 independent experiments. (A) EdU. Error bars are mean ±2 × SEM for each time point (n ≥ 8 cells for all time points, n ≥ 613 cells per condition). (B) Geminin immunofluorescence 1–3 h after S entry. Box is IQR and median, and whiskers are 1.5 × IQR. G1 cells are DMSO-treated G1 cells (basal geminin), and dashed line is median of G1 cells. n ≥ 37 cells per condition. (C and D) RT-QIBC in mitogen-released MCF10A cells. MLN-4924 added 4 h before fixation. Cells 2-3 h after APC/C^Cdh1^ inactivation with cyclin E/A-CDK activity ≥0.8. (C) Dashed lines are negative thresholds, and shaded curves are median in bins of PCNA levels (n ≥ 1,369 cells pooled from 10 replicate wells). (D) Proportion of PCNA(+) cells that are EdU(−) in each of 10 replicate wells. Error bars are mean ±2 × SEM. Two-sample t test p values: siCtrl DMSO vs. siCtrl MLN-4924 (7.8 × 10^−6^), siCtrl DMSO vs. siGeminin DMSO (0.38), siCtrl MLN-4924 vs. siGeminin MLN-4924 (2.3 × 10^−5^). (E) DNA synthesis is inhibited in the presence of CDT1 after origin firing. (F-H) RT-QIBC in mitogen-released MCF10A cells 2-3 h after S entry. Representative of 3 independent experiments. (F) Top:dose-response of EdU to ND-CDT1 expression.Error bars are mean ±2 × SEM for bins of ND-CDT1 expression (bins ≥ 75 cells, 12,039 cells total siCtrl, 4,573 cells total siGeminin). Curves are fit Hill equations. Fit parameters in STAR Methods. Bottom: corresponding cell counts for bins. Dashed lines are endogenous CDT1 levels (CDT1_Endogenous_) and degraded CDT1 (CDT1 _low_), calculated from Figure S3H, and fit IC_50_. (G) Left: fit ND-CDT1 dose-response curves at 12 levels of geminin expression. Right: color code for geminin expression. Geminin_Low_ is negative threshold. n ≥ 660 cells per fit. (H) Heatmap of median EdU (color) at given geminin and ND-CDT1 levels. Points represent IC_50_ at each geminin level. (n = 29,350 cells.) See also Figure S4.

As an additional control, we confirmed that endogenous CDT1, not just overexpressed CDT1, can inhibit DNA synthesis when it fails to be degraded in S phase. To prevent the degradation of CDT1 in S phase, we acutely treated cells with MLN-4924, which blocks the activity of cullin-RING E3 ubiquitin ligases, including CRL4^Cdt2^ (Figures S4A and S4B).^29^ Cells treated with MLN-4924 had suppressed EdU incorporation following S-phase entry (Figure 3A, siCtrl conditions). Similar to overexpressed CDT1-mCherry, MLN-4924 increased the population of cells with chromatin-bound PCNA and low EdU incorporation (Figures 3C and 3D, siCtrl conditions). Knockdown of CDT1 partially rescued EdU incorporation, while knockdown of CDKN1A (p21) or CDKN1B (p27), other proteins stabilized by MLN-4924,^30^ did not (Figures S4B and S4C). We conclude that both overexpressed and endogenous CDT1 can suppress DNA synthesis during S phase. These findings provide a potential explanation of how cells avoid re-replication during the overlap period when CDT1 is present together with fired origins in early S phase, as the amount of synthesized DNA, the substrate of re-replication, at these fired origins would be reduced until CDT1 is fully degraded (Figure 3E).

### Geminin counteracts the inhibition of DNA synthesis by CDT1

When we prevented CDT1 degradation using MLN-4924, cells still started to increase EdU incorporation in S phase after a delay (Figure 3A). While geminin is initially very low in early S phase, it gradually accumulates throughout S phase (Figure 1F). We considered whether geminin binding to CDT1 could ultimately abrogate the inhibitory action of CDT1 on DNA synthesis. Consistent with this hypothesis, geminin knockdown prolonged the suppression of EdU incorporation in MLN-4924-treated cells (Figures 3A–3D, S4B, and S4C).

To more directly determine whether geminin antagonizes inhibition of DNA synthesis by CDT1, we measured the suppression of DNA synthesis at different levels of ND-CDT1 in the presence or absence of geminin. Cell populations with inducible ND-CDT1 showed variable expression between cells, and after computationally stratifying cells by their ND-CDT1 expression, we found that EdU incorporation was inhibited in a dose-dependent manner (Figure 3F). When geminin was allowed to accumulate, only cells with moderate-to-high ND-CDT1 could suppress DNA synthesis 2–3 h post S-phase entry. However, when we knocked down geminin, DNA synthesis stayed suppressed at much lower levels of ND-CDT1 (Figures 3F and S4D). By analyzing dose responses at a range of geminin levels (Figure S4E), we observed that DNA synthesis was increasingly inhibited by ND-CDT1 as geminin decreased, with the IC_50_ for ND-CDT1 decreasing linearly with geminin levels (Figures 3G, 3H, S4F, and S4G).

We conclude that geminin not only has a role in inhibiting CDT1 licensing^5^ but also prevents CDT1 from inhibiting DNA synthesis. In this way, DNA synthesis can proceed either after CDT1 is degraded or after geminin levels have sufficiently increased to inhibit CDT1. Nevertheless, during the overlap period of CDT1 and fired origins in early S phase, geminin is low and cannot inhibit CDT1, arguing that suppression of DNA synthesis by CDT1 alone deters re-replication during the overlap period.

### CDT1 suppresses DNA synthesis during the overlap period of licensing and firing

If endogenous CDT1 is indeed responsible for inhibiting DNA synthesis during the first 30 min of S phase, prematurely inactivating CDT1 should accelerate the start of DNA synthesis. Since CDT1 is required for origin licensing, we could not use long-term CDT1 knockdown. Instead, we made use of the licensing kinetics in MCF10A cells, which complete the majority of origin licensing shortly after anaphase and then further boost licensing during G1 (Figures S5A and S5B). In this way, acutely inactivating CDT1 during G1 would reduce but not prevent origin licensing, which cells can tolerate.^31^

**Figure 4:**
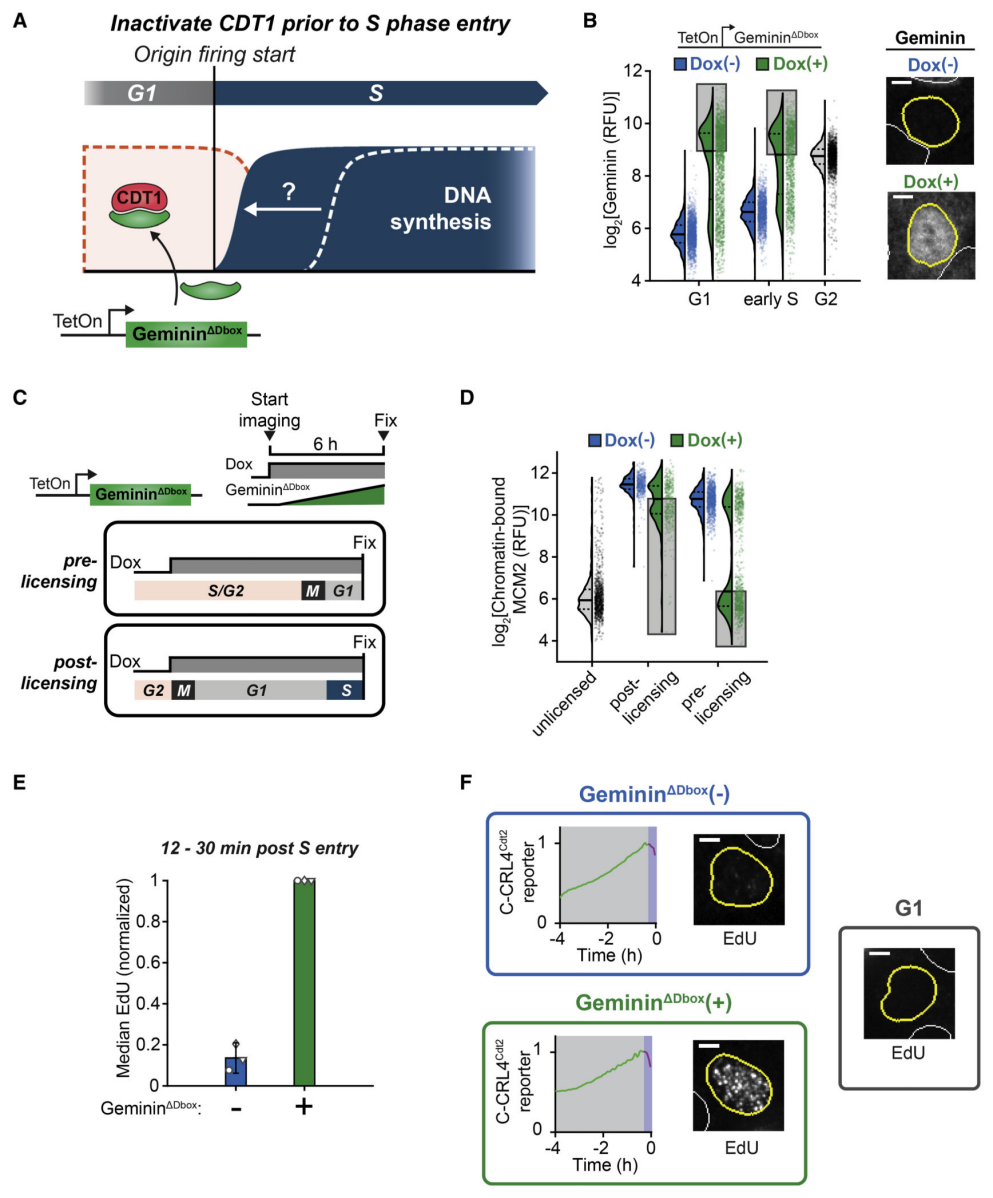
CDT1 suppresses DNA synthesis during the overlap period of licensing and firing (A) Prematurely inactivating CDT1 with doxycycline (Dox)-inducible Geminin^ΔDbox^ in G1 to accelerate the start of DNA synthesis. (B-D) RT-QIBC in MCF10A cells with Geminin^ΔDbox^ induced during live-cell imaging of C-CRL4^Cdt2^ reporter. Dashed and solid lines in violin plots are IQR and median. Representative of 2 (D) or 3 (B) independent experiments. Scale bars, 5 μm. (B) Geminin immunofluorescence (detects geminin and Geminin^ΔDbox^). Left: G1 (1–2 h post anaphase), S (≤0.5 h in S) and G2 (4N DNA, EdU(−), no Dox). Shaded area is upper 50% of cells, which induce Geminin^ΔDbox^ higher than median G2. n ≥ 1,316 cells per condition. Right: example cells 1 h in G1. (C) Cells were identified in two groups. Pre-licensing: Dox added ≥5 h before mitosis, and fixed ≤1 hinG1. Post-licensing:Dox was added ≤1 hbefore mitosis and fixed ≤ 1 h in S. (D) Pre-extracted cells. MCM2 from unlicensed cells was estimated from G2 MCM2 signal (4N DNA and PCNA negative). Shaded area is lower 50% of cells, corresponding to cells in shaded bar in Figure 4B. n ≥ 434 per condition. (E and F) RT-QIBC in post-licensing Dox addition MCF10A cells. EdU added 30 min before fixation. Cells that were in S phase for 12–30 min and had not fully degraded CDT1 were analyzed. Geminin^ΔDbox^ (+) cells selected based on Geminin stain. (E) For each of 3 independent experiments, the median of cells was taken (n ≥ 31 cells per replicate per condition; Geminin^ΔDbox^ (−): 120 cells total; Geminin^ΔDbox^ (+): 213 cells total) and normalized to Geminin^ΔDbox^ (+) condition. Error bars are mean ±2 × SEM (Geminin^ΔDbox^ (−) cells are 13.6% ± 7.4% of Geminin^ΔDbox^ (+) cells). See Figure S5G for estimated absolute quantification. (F) Representative EdU images and matching C-CRL4^Cdt2^ traces (magenta trace represents time following S entry). Geminin^ΔDbox^ (−) and Geminin^ΔDbox^ (+) cells 17.2 and 16.9 min in S phase. Scale bars, 5 μm. See also Figure S5.

We generated a cell line with Dox-inducible geminin to prematurely inactivate CDT1 during G1 (Figures 4A and S5C). To prevent geminin degradation in G1 by APC/C^Cdh1^, we mutated the geminin D-box motif,^13,32^ and the resulting cell line induced Geminin^ΔDbox^ to levels higher than normal G2 geminin levels in ~50% of G1 cells (Figure 4B).

In initial control experiments, where we induced Geminin^ΔDbox^ in asynchronous cells, cells that received Dox well before mitosis and thus expressed Geminin^ΔDbox^ by the time they reached anaphase had completely inhibited origin licensing, indicating that Geminin^ΔDb0x^ had fully inhibited CDT1 (“pre-licensing,” Figures 4C and 4D). In contrast, cells that divided shortly after Dox addition, thus going through early G1 without Geminin^ΔDbox^, only exhibited the expected moderate reduction in origin licensing resulting from inhibited licensing late in G1 (“post-licensing,” Figures 4C and 4D). We could therefore examine the latter group of cells to determine whether premature CDT1 inactivation accelerates DNA synthesis at S-phase start. Markedly, in the first 30 min of S phase, cells with CDT1 neutralized by Geminin^DDbox^ exhibited approximately 5-to 10-fold higher EdU incorporation than control cells (Figures 4E, 4F, and S5D–S5G). We thus conclude that CDT1 is present in early S phase and suppresses DNA synthesis, providing a protective mechanism against re-replication during the overlap period where CDT1 is present together with fired origins.

### CDT1 inhibits DNA synthesis independently of the intra-S-phase checkpoint and re-replication

Next, we sought to determine the mechanism by which CDT1 inhibits DNA synthesis. The intra-S-phase checkpoint, which limits the rate of DNA synthesis, can be activated in response to rereplication and DNA damage caused by CDT1 dysregulation.^9,33–35^ Alternatively, the addition of high levels of CDT1 to replicating *Xenopus* egg extracts not only triggers checkpoint activation but also directly inhibits replication fork elongation,^36,38^ suggesting other plausible mechanisms. Of note, CDT1-mediated inhibition of DNA synthesis in *Xenopus* egg extract was interpreted to be a safety response to aberrant CDT1 expression during S phase, rather than a protective mechanism routinely utilized during an overlap period in early S phase.

**Figure 5:**
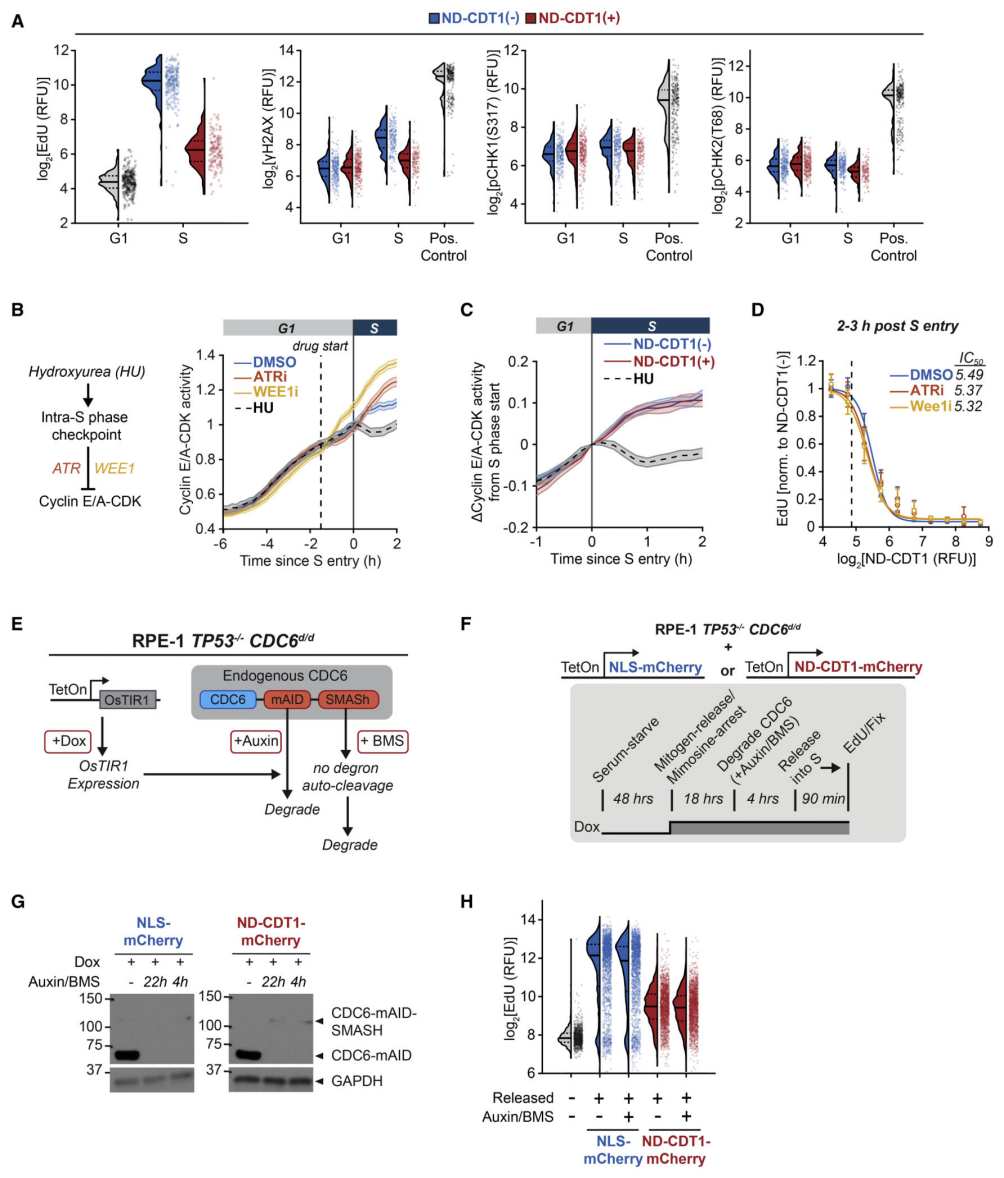
CDT1 inhibits DNA synthesis independently of the global intra-S-phase checkpoint and re-replication (A) RT-QIBC in siGeminin-treated, mitogen-released MCF10A cells. S cells were 1–2 h into S phase. Positive controls were cells treated with 1 μM MK-1775 (WEE1i) for 4 h in S cells, which caused DNA damage. n ≥ 146 cells per condition. (B) Mitogen-released MCF10A cells. 2 μM AZ-20 (ATRi), 1 μM WEE1i, or 2 mM hydroxyurea (HU) were added to cells 14 h after release. Cells that received drug 1–2 h before S entry were analyzed (dashed line is 1.5 h). Shaded area is mean ± 2 × SEM. n = 167 (DMSO), 145 (ATRi), 239 (WEE1i), and 119 (HU) cells. (C) Mitogen-released MCF10A cells. 2 mM HU added to HU condition 1–2 h before S entry. Shaded area is mean ± 2 × SEM. n ≥ 336 cells per condition. (D) RT-QIBC of mitogen-released, siGeminin-treated MCF10A cells 2–3 h in S phase. 2 μM ATRi or 1 μM WEE1i were added 4 h before fixation. Error bars are mean ±2 × SEM for bins of ND-CDT1 (≥12 cells per bin, ≥770 cells per condition). Dashed line is threshold for ND-CDT1(−). Lines are fit Hill equations (fit parameters in STAR Methods). (E) RPE-1 *TP53^−/−^ CDC6^d/d^* cells degrade endogenous CDC6 with addition of Auxin and BMS-650032 (BMS). (F-H) Dox-inducible ND-CDT1-mCherry or NLS-mCherry in RPE-1 *TP53^−/−^ CDC6^d/d^* cells. Cells were mitogen-released in the presence of mimosine and doxycycline (Dox) for 18 h. CDC6 was degraded for 4 h, and then cells were released from mimosine arrest for 1.5 h, pulsed with EdU, and fixed. (G) Western blot of CDC6 levels after acute 4 h or long-term 22 h degradation. Upper band is CDC6 with uncleaved SMASh-tag. (H) QIBC of EdU in cells with inactive APC/C^Cdh1^. NLS-mCherry/ND-CDT1-mCherry-positive cells were chosen based on gates Figure S5M. n = 1,120 cells (unreleased), 2,000 cells (other conditions). Dashed and solid lines in violin plots are IQR and median. Data representative of 2 independent experiments. See also Figure S5.

We first focused on whether the intra-S-phase checkpoint mediates the inhibition of DNA synthesis by CDT1. We overexpressed ND-CDT1 in cells with geminin knocked down to maximize the possibility of producing DNA damage and observed no increases in _γ_H2AX, phospho-Chk1(S317) and phospho-Chk2(T68), markers of DNA damage and the intra-S-phase checkpoint (Figure 5A). As an independent measure of checkpoint activation, we turned to a live-cell reporter of the activity of cyclin E/A complexed with CDK2/1 (cyclin E/A-CDK) (Figure S5H).^39,40^ Cyclin E/A-CDK activity increases throughout the cell cycle starting in G1. Cyclin A knockdown confirmed that cyclin A activity does not contribute to the G1 increase in reporter activity (Figures S5I and S5J), consistent with the low cyclin A IF staining during G1- and S-phase start (Figure 1G). Since cyclin E/A-CDK activity is partially inhibited by the intra-S-phase checkpoint, it can be used as a proxy for checkpoint activation.^41^ Accordingly, hydroxyurea (HU) reduced cyclin E/A-CDK activity in S phase, while inhibitors of checkpoint mediators ATR or WEE1 increased cyclin E/A-CDK activity (Figure 5B). However, ND-CDT1 expression did not decrease cyclin E/A-CDK activity (Figure 5C). Furthermore, ND-CDT1 suppressed EdU incorporation with the same IC50 in the presence of ATR and WEE1 inhibitors (Figure 5D). Together, these results show that the intra-S-phase checkpoint does not mediate the suppression of DNA synthesis by CDT1.

It has also been suggested that re-replication can inhibit DNA synthesis independently of the intra-S-phase checkpoint.^33,42^ If re-replication is necessary for the suppression of DNA synthesis by CDT1, blocking licensing activity in S phase, which is necessary for re-replication, should rescue DNA synthesis. To inhibit licensing, we used a previously developed RPE-1 *TP53^−/−^* cell line with mAID and SMASh-tag inducible degrons knocked into both copies of *CDC6*(referred to here as *CDC6^d/d^*), another essential licensing factor (Figure 5E).^43^ In this cell line, CDC6 can be rapidly degraded to very low levels within 4 h, and control experiments confirmed that degrading CDC6 inhibited origin licensing (Figure S5K).

We introduced Dox-inducible constructs for ND-CDT1-mCherry and control NLS-mCherry into the *CDC6^d/d^* cell line and synchronized cells in late G1 by releasing cells from G0 into a mimosine arrest, which blocks cells after origin licensing (Figure S5L).^44^ In the final 4 h before releasing cells from mimosine arrest into S phase, we degraded CDC6 to prevent potential further licensing during S phase (Figures 5F and 5G). ND-CDT1-mCherry inhibited EdU incorporation following mimosine release, regardless of CDC6 degradation, arguing that re-replication is not necessary for CDT1 to inhibit DNA synthesis (Figures 5H and S5M). Furthermore, this cell line does not have functional TP53 (p53), which has also been implicated in the DNA damage response to re-replication.^9^ We conclude that rereplication, p53, and intra-S-phase checkpoint activation are not required for CDT1-mediated inhibition of DNA synthesis in early S phase, arguing that CDT1 directly suppresses DNA synthesis.

### CDT1 inhibits replication fork elongation while permitting origin firing

**Figure 6:**
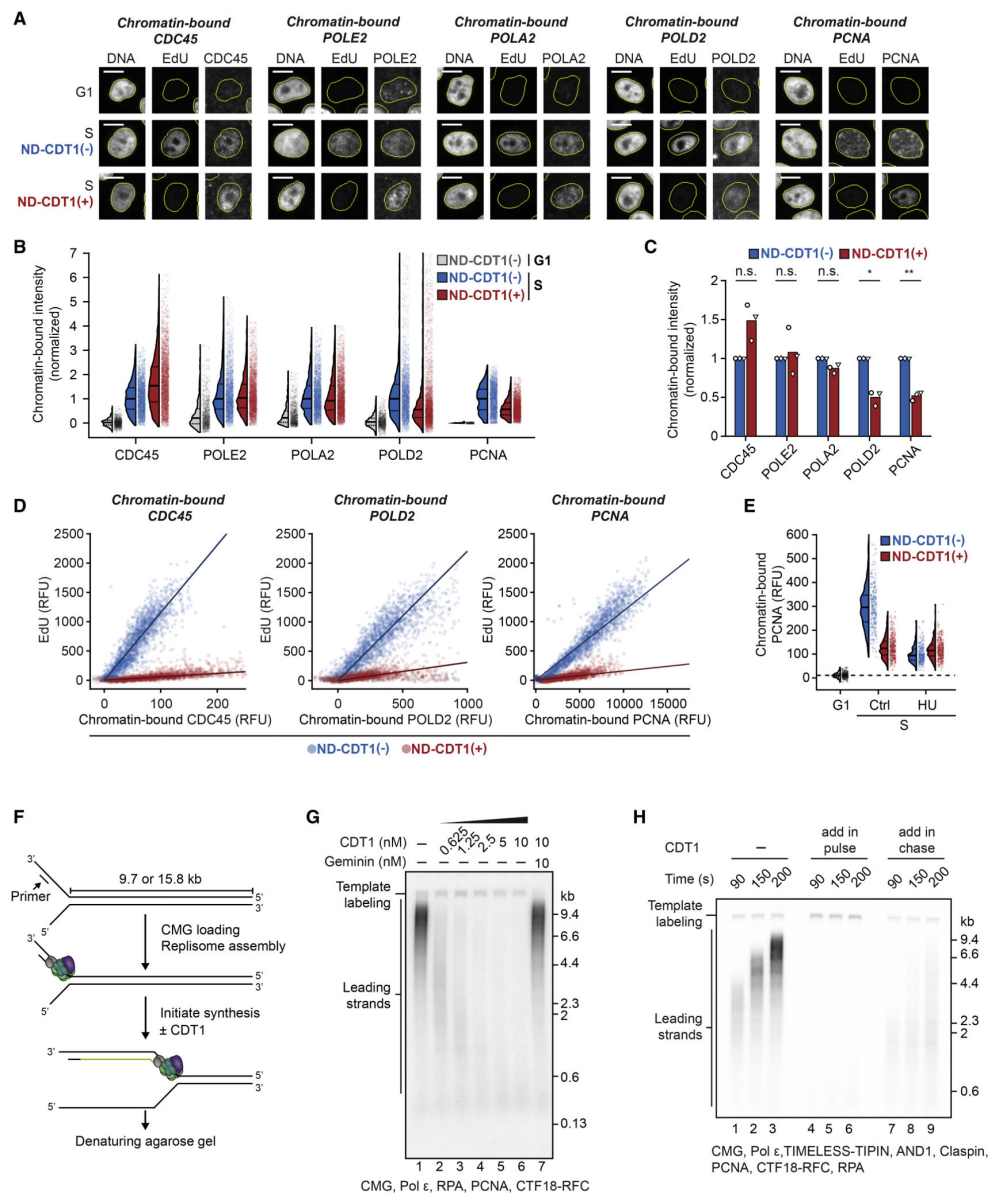
CDT1 inhibits replication fork elongation while permitting origin firing (A–E) RT-QIBC of chromatin-bound replisome components in mitogen-released MCF10A cells. G1 and S cells were identified (see STAR Methods). Analysis for additional proteins in Figures S6A–S6E. (A) Representative images of EdU and CDC45, Pol ε (POLE2), Pol α (POLA2), Pol δ (POLD2), and PCNA. Scale bars, 10 μm. (B) G1 mode intensities were subtracted off signals, and values were normalized to ND-CDT1(−) median. n = 2,000 cells per condition. (C) Summary of median values from 3 independent experiments of Figure 6B. One-sample Student’s t test: p values CDC45(n.s.): 6.94 × 10^−2^, POLE2 (n.s.):.693, POLA2 (n.s.): 6.41 × 10^−2^, POLD2 (*): 1.21 × 10^−2^, PCNA (**): 4.3 × 10^−3^. (D) EdU vs. replisome components in S cells. Fit line from linear regression (n = 2,000 cells per condition). (E) Cells were treated with 2 mM hydroxyurea (HU) during final 4 h of imaging and then fixed. Dashed line is median G1 signal. n ≥ 281 cells per condition. (F) CMG is loaded onto forked DNA template, replisome is assembled, and replication is initiated in the presence or absence of CDT1. Synthesized DNA is radiolabeled for visualization (green strand). (G) Denaturing agarose gel analysis of 6 min replication reactions (15.8 kbp DNA template) with indicated proteins. 10 nM geminin was pre-incubated with equimolar CDT1 where indicated. CMG-independent template labeling is marked. (H) Denaturing agarose gel analysis of pulse-chase experiment (15.8 kbp DNA template) with the indicated proteins. Synthesized DNA was labeled in pulse, and chase was added after 50 s. 10 nM CDT1 was added with pulse or chase. Dashed and solid lines in violin plots are IQR and median. Data representative of 3 independent experiments. See also Figure S6.

We next determined whether CDT1 suppresses DNA synthesis by inhibiting origin firing, or by inhibiting replication fork elongation after origin firing. To quantify origin firing and recruitment of replication factors to the replication fork in the presence of ND-CDT1, we measured chromatin-bound CDC45, TIMELESS, DNA polymerases epsilon, alpha, and delta (Pol ε, α, and δ), and PCNA (Figures 6A and S6A). Replication factors that are part of or bind to the CMG helicase (CDC45, TIMELESS, Pol ε, and Pol a) did not have impaired chromatin association in the presence of ND-CDT1, while Pol δ, which synthesizes lagging strands, and PCNA were present but approximately 50% reduced (Figures 6A–6C and S6A–S6C).^45^ These findings are consistent with our observation that CRL4^Cdt2^, which depends on chromatin-bound PCNA, is still activated in the presence of ND-CDT1 (Figures S3F and S3G).

To determine whether reduced chromatin-bound Pol δ and PCNA are responsible for inhibited DNA synthesis, we simultaneously measured chromatin-bound replication factors and EdU incorporation. In control conditions there was a linear relationship between each chromatin-bound protein (CDC45, TIMELESS, Pol ε, Pol α, Pol δ, and PCNA) and EdU incorporation, indicating that each chromatin-bound protein signal is proportional to the number of active replication forks (Figures 6D and S6D). However, EdU incorporation was greatly reduced at matching levels of chromatin-bound protein in the presence of ND-CDT1 (Figures 6D, S6D, and S6E). This was the case even for Pol δ and PCNA, where EdU incorporation was much lower than expected given a 2-fold reduction in chromatin-bound levels. These results are consistent with CDT1 inhibiting replication fork elongation at fired origins.

Since PCNA and Pol δ are found at lagging-strand Okazaki fragments,^45,46^ we hypothesized that the approximately 2-fold reduction in chromatin-bound PCNA and Pol δ in the presence of ND-CDT1 was a consequence of reduced fork elongation. Accordingly, chromatin-bound PCNA was lowered by HU, which blocks fork elongation, but was not further lowered in cells also expressing ND-CDT1 (Figure 6E). Such a reduction of PCNA at stalled forks has previously been reported.^47,48^ In aggregate, our results indicate that CDT1 does not act by inhibiting origin firing but rather inhibits replication fork elongation.

To confirm that CDT1 can inhibit replication fork elongation, we used a recently developed *in vitro* assay for reconstituting CMG-dependent DNA synthesis using purified human proteins.^49^ In this assay, replisome components (including CMG helicase) are loaded onto forked DNA templates and can perform leading-strand DNA synthesis alone or leading- and lagging-strand DNA synthesis (Figure 6F). Importantly, this assay is independent of origin firing. In this system, addition of CDT1 strongly inhibited DNA synthesis in a dose-dependent manner (Figures 6G and S6F). CDT1 inhibited both leading- and lagging-strand DNA synthesis (Figures 6G and S6G) and inhibited DNA synthesis not only when added to reactions from the start but also when added to already elongating forks (Figure 6H). Pre-incubation of CDT1 together with geminin neutralized its inhibitory effect (Figures 6G and S6G). Finally, CDT1 inhibited DNA synthesis in a replisome composed only of CMG, Pol ε, and replication protein A (RPA) (Figure S6H). These *in vitro* reconstitution experiments demonstrate that CDT1 inhibits replication fork elongation and DNA synthesis.

### CDT1 inhibits CMG helicase through its MCM-binding domains

We next focused on how CDT1 suppresses replication fork elongation in early S phase. Replication fork elongation could be suppressed by inhibition of the replicative DNA polymerases or by inhibition of CMG helicase, which is responsible for unwinding double-stranded DNA. CDT1 contains a high-affinity interaction with PCNA through its PIP degron, and it has been suggested that PIP-degron-containing proteins can interfere with the binding of polymerases to PCNA, thereby inhibiting DNA synthesis.^50,51^ However, ND-CDT1 has its PIP degron removed (Figure 2D), and CDT1 inhibits replication reactions *in vitro* independently of PCNA (Figure S6H), arguing against CDT1 inhibiting polymerases.

**Figure 7:**
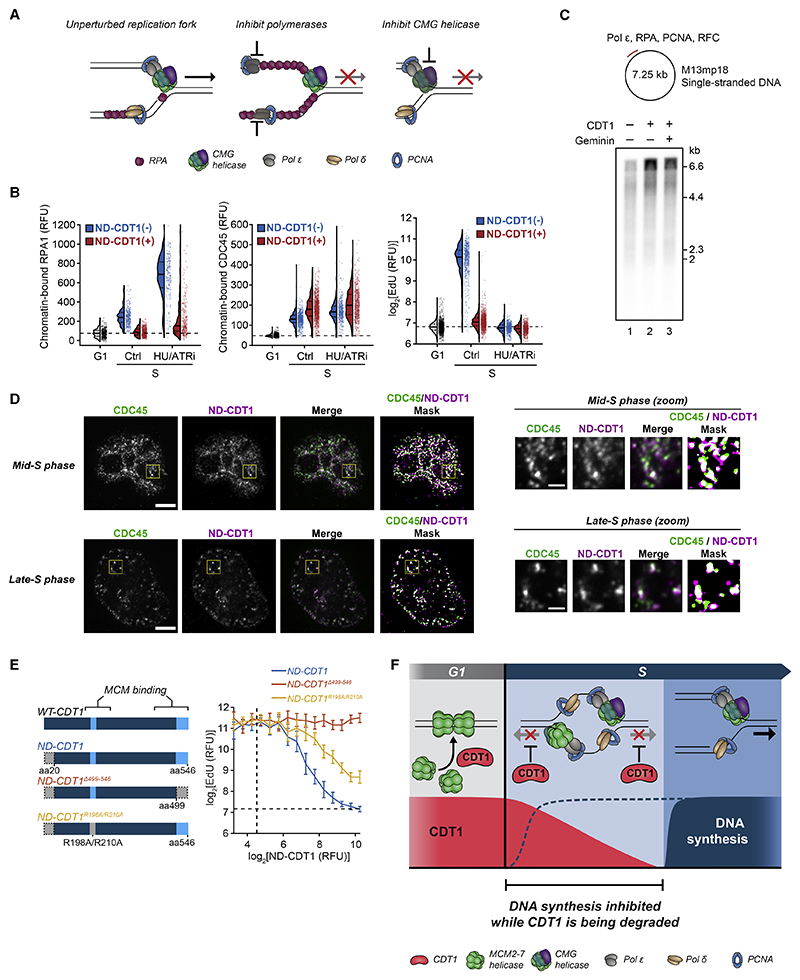
CDT1 inhibits CMG helicase through its MCM-binding domains (A) Mechanisms underlying inhibited replication fork progression. (B) Mitogen-released MCF10A cells were treated with 2 mM hydroxyurea (HU) and 2 μM AZ-20 (ATRi) during the final 4 h. G1 and S cells were identified (see STAR Methods). n ≥ 182 cells per condition. Dashed line is G1 signal median. Representative of 3 independent experiments. (C) Top: polymerase epsilon primer extension assay (M13mp18 circular single-stranded DNA template). Bottom: denaturing agarose gel analysis of 15 min primer extension reactions. 10 nM CDT1 pre-incubated with equimolar geminin where indicated. Representative of 3 independent experiments. (D) CDC45-GFP U2OS cells with doxycycline (Dox-inducible ND-CDT1 were treated with Dox for 8 h, pre-extracted, and co-stained for ND-CDT1(anti-HA-tag) and CDC45 (anti-GFP). Mid- or late-S-phase cells identified by CDC45 pattern. Signals were masked by thresholding. Yellow box is zoomed region. Representative of 35 (mid S) or 18 (late S) cells. Scale bars, 5 μm (full image) and 1 μm (zoom). Co-localization analysis in Figure S7B. (E) Left: MCM-binding region mutants of ND-CDT1. WT-CDT1, wild-type CDT1. Right: dose responses of EdU to ND-CDT1 (anti-HA-tag), 1–2 h after S entry in mitogen-released siGeminin-treated MCF10A cells. Error bars are mean ± 2 × SEM(n ≥ 26 cells per bin, n ≥ 1,048 cells per condition). Dashed lines are negative threshold. Mutant expression in Figure S7C. (F) Diagram of CDT1 regulation of DNA synthesis in early S. See also Figure S7.

To determine whether CDT1 inhibits polymerases or CMG helicase, we examined the production of single-stranded DNA (ssDNA) at the replication fork. If CDT1 inhibited polymerases, ssDNA would accumulate as CMG helicase unwound DNA that the polymerases could not fill (Figure 7A).^21,36,52^ As a measure of ssDNA, we examined the amounts of chromatin-bound ssDNA-binding protein RPA. In control cells without ND-CDT1 expression, there was measurable chromatin-bound RPA in S phase due to the normal production of ssDNA at replication forks (Figure 7B). Markedly, S-phase cells expressing ND-CDT1 had diminished RPA binding compared with control cells despite having similar amounts of origin firing, as measured by chromatin-bound CDC45. ND-CDT1 also prevented the increase in chromatin-bound RPA in response to HU and ATR inhibitor cotreatment (Figure 7B), which generates a large increase in ssDNA.^21^ These results suggest that CMG helicase-mediated unwinding of DNA is suppressed in the presence of CDT1. These findings are supported by findings in *Xenopus* egg extract, where the addition of CDT1 also reduced chromatin-bound RPA.^36^ Furthermore, CDT1 did not inhibit DNA synthesis in a Pol ε primer extension assay, which uses an ssDNA template and does not contain or rely on CMG helicase (Figure 7C).^49^ In aggregate, our data are consistent with CDT1 inhibiting CMG helicase, rather than polymerase in early S phase.

As part of its role in origin licensing, CDT1 directly binds to soluble MCM helicases through two MCM-binding regions, which results in a conformational change in the MCM helicases that allows their loading onto origins.^5,20,53^ This interaction is blocked when CDT1 is bound by geminin.^5^ In *Xenopus* egg extracts, truncations in CDT1 that overlapped with MCM-binding regions interfered with the inhibition of DNA synthesis by CDT1, as did geminin addition.^37^ Given that a minimal replisome consisting of CMG, Pol ε, and RPA is inhibited by CDT1 *in vitro*(Figure S6H), and that Pol ε is not intrinsically inhibited by CDT1 (Figure 7C), we hypothesized that the same regions in CDT1 that mediate licensing by binding MCM might also interact with and inhibit the activated CMG helicase complex (which contains MCMs). Consistent with this hypothesis, chromatin-bound ND-CDT1 co-localized with chromatin-bound CDC45, a component of CMG helicase (Figures 7D, S7A, and S7B). Since ND-CDT1 cannot bind PCNA due to its lack of PIP degron,^16^ the co-localization was not the result of binding to PCNA at the replication fork.

Finally, we tested whether the MCM-binding regions of CDT1 are necessary for CDT1 to inhibit DNA synthesis in human cells. The first MCM-binding region is found at its C terminus, while a second MCM interaction interface was identified near R210 of human CDT1.^5,20^ We overexpressed ND-CDT1 with either a truncation at residue 498 in the C-terminal MCM-binding domain (ND-CDT1 ^Δ499–546^), which abolishes licensing and MCM-binding,^54^ or point mutations R198A/R210A in the other interface (ND-CDT1^R198A/R210A^), which severely diminishes licensing activity.^55^ We examined their inhibitory effect on DNA synthesis with geminin knocked down to account for potential differential geminin regulation of the mutants. ND-CDT1 ^Δ499–546^ could not inhibit EdU incorporation at all, while ND-CDT1^R198A/R210A^ was impaired in its ability to inhibit EdU, with an IC50 approximately double that of normal ND-CDT1 (Figures 7E and S7C). Consistent with this result, geminin, which prevents binding of CDT1 to MCM helicases,^5^ also prevented CDT1-mediated inhibition of DNA synthesis (Figure 3F). Together, our different lines of evidence support a model in which CDT1 suppresses DNA synthesis by inhibiting CMG helicase activity.

## Discussion

Our study focused on the fundamental problem in eukaryotic DNA replication of how cells duplicate the genome precisely once every cell cycle. It is generally thought that the solution to this problem is the strict temporal separation of origin licensing from origin firing to prevent re-replication. Vertebrate licensing regulation is centered around the inhibition of licensing factor CDT1 from S-phase entry to anaphase through CDT1 degradation by CRL4^Cdt2^ and SCF^Skp2^, and inhibition by geminin and cyclin A.^5^ However, only CRL4^Cdt2^ was proposed to prevent re-licensing in early S phase, and, given the dependence of CRL4^Cdt2^ activity on PCNA bound to replication forks, it has been noted this mechanism cannot fully separate licensing and firing in early S phase.^1,4,17^ Considering the large number of replication origins, even a short overlap of CDT1 and fired origins while CDT1 is being degraded could allow for the re-licensing of DNA, raising the question of how cells might prevent re-replication during this vulnerable period.

In this work, we identified an overlap period of CDT1 protein with fired origins in early S phase that lasts approximately 30 min in human cells, during which geminin and cyclin A levels are still low. Strikingly, we found that CDT1 inhibits DNA synthesis during this overlap period, and this inhibition is only relieved once CDT1 is fully degraded. We found that CDT1 suppresses CMG helicase, and thus replication fork elongation, at fired origins through its MCM-binding domains. By suppressing replication fork elongation after origin firing, CDT1 allows its own degradation by CRL4^Cdt2^ to be completed before DNA synthesis commences (Figure 7F). Importantly, this mechanism is robust toward changes in CDT1 expression levels, as cells with higher amounts of CDT1 that take longer to degrade would suppress DNA synthesis longer.

Mechanistically, the ability of CDT1 to suppress CMG helicase during fork elongation while allowing origin firing implies that CDT1 does not prevent the initial origin unwinding performed by CMG during origin firing, and only acts afterward on CMG at elongating forks. One possible explanation could be that origin firing factors, which bind and activate MCM helicases and then are released, can prevent CDT1 from inhibiting CMG specifically during origin firing.

Previous studies have identified responses to re-replication and DNA damage in human cells that reduce DNA synthesis in response to aberrant CDT1 regulation.^7,35,54,56^ Critically, most characterized mechanisms require that cells first re-replicate DNA, and the resulting reduced DNA synthesis serves to minimize further damage. In contrast, the CMG helicase inhibition by CDT1 identified in our study can act before re-replication is produced.

Nevertheless, the finding that CDT1 can directly inhibit DNA synthesis still raises the question of why dysregulated CDT1 can result in re-replication and DNA damage at all.^6–9,57^ A likely explanation is that the small amount of DNA synthesis in the presence of CDT1 can, over long periods, allow for enough residual DNA synthesis to produce re-replication. Furthermore, since overexpressed or dysregulated CDT1 might be incompletely degraded, CDT1 could be reduced to levels too low for effective suppression of DNA synthesis, but high enough for some re-licensing to occur over time. In support of this, non-degradable mutants of CDT1 paradoxically produce less re-replication than wild-type CDT1.^54,56^

Overall, our study shows that licensing and firing have an overlap period, arguing for a revision of the concept that origin licensing must be separated from origin firing to prevent re-replication. Instead, cells separate origin licensing from DNA synthesis in early S phase. Importantly, we identify that this separation is enforced by CDT1 inhibiting the CMG helicase after origin firing until CDT1 is fully degraded. All previously identified re-replication prevention mechanisms center around the inhibition of licensing factors as cells enter S phase.^1^ In contrast, we identified a new class of licensing regulation whereby a licensing factor itself inhibits S-phase progression. We propose that both classes of regulation are critical for safeguarding genome integrity.

### Limitations of the study

Based on (1) CDT1 reducing ssDNA produced by CMG, (2) *in vitro* assays showing CDT1 inhibits CMG-dependent DNA synthesis but does not inhibit Pol ε itself, and (3) the dependence of inhibition on the MCM-binding domains of CDT1, we argue that CDT1 directly inhibits CMG helicase. However, the data cannot exclude the possibility that CDT1 impacts another aspect of replisome function, such as helicase-polymerase coupling, and thereby reduces helicase activity. Additional *in vitro* assays examining the CMG activity in the absence of polymerase will be needed to distinguish these possibilities. Furthermore, while we found that inhibition of CMG helicase by CDT1 is dependent on the MCM-binding domain of CDT1, we have yet to elucidate the structural and biophysical basis of this inhibition. Finally, while our work in human cells and work in *Xenopus* egg extract suggests that CDT1 inhibits DNA synthesis in vertebrates, future work is needed to examine whether this mechanism is conserved other organisms such as yeast and *Drosophila*, which many studies have used as model systems to study DNA replication.

## Star★Methods

### Key Resources Table

**Table:** 

REAGENT or RESOURCE	SOURCE	IDENTIFIERr
Antibodies		
rabbit anti-CDT1 mAb [EPR17891]	Abcam	Cat# ab202067, RRID:AB_2651122
rabbit anti-Geminin pAb	Atlas Antibodies	Cat# HPA049977, RRID:AB_2680978
mouse anti-Cyclin A mAb [B-8]	Santa Cruz Biotech	Cat# sc-271682, RRID:AB_10709300
mouse anti-PCNA mAb [PC10]	Santa Cruz Biotech	Cat# sc-56, RRID:AB_628110
rabbit anti-MCM2 mAb [D7G11] XP	Cell Signaling Technology	Cat# 3619, RRID:AB_2142137
rabbit anti-p21 mAb [12D1]	Cell Signaling Technology	Cat# 2947, RRID:AB_823586
rabbit anti-HA tag mAb [C29F4]	Cell Signaling Technology	Cat# 3724, RRID:AB_1549585
rabbit anti-CDC45 mAb [D7G6]	Cell Signaling Technology	Cat# 11881, RRID:AB_2715569
rabbit anti-POLA2 pAb	Atlas Antibodies	Cat# HPA037570, RRID:AB_10672280
rabbit anti-POLD2 pAb	Atlas Antibodies	Cat# HPA026745, RRID:AB_1855520
rabbit anti-POLE2 pAb	Atlas Antibodies	Cat# HPA027555, RRID:AB_10610282
rabbit anti-Timeless mAb [EPR5275]	Abcam	Cat# ab109512, RRID:AB_10863023
rabbit anti-phospho-Chk1(S317) mAb [D12H3]	Cell Signaling Technology	Cat# 12302, RRID:AB_2783865
rabbit anti-phospho-Chk2(T68) pAb	Cell Signaling Technology	Cat# 2661, RRID:AB_331479
rabbit anti-phospho-histone H2A.X(S139) pAb	Cell Signaling Technology	Cat# 2577, RRID:AB_2118010
rabbit anti-RPA70/RPA1 mAb [EPR3472]	Abcam	Cat# ab79398, RRID:AB_1603759
mouse anti-CDC6 mAb [180.2]	Santa Cruz Biotech	Cat# sc-9964, RRID:AB_627236
rabbit anti-GAPDH mAb [D16H11] XP	Cell Signaling Technology	Cat# 5174, RRID:AB_10622025
mouse anti-GFP [9F9.F9] mAb	Abcam	Cat# ab1218, RRID:AB_298911
rabbit anti-p27 mAb [D69C12] XP mAb	Cell Signaling Technology	Cat# 3686, RRID:AB_2077850
rabbit anti-Geminin pAb	Proteintech	Cat# 10802-1-AP, RRID:AB_2110945
mouse anti-GAPDH (6C5) mAb	Santa Cruz Biotech	Cat# sc-32233, RRID:AB_627679
goat anti-rabbit IgG, HRP-linked Antibody	Cell Signaling Technology	Cat# 7074, RRID:AB_2099233
horse anti-mouse IgG, HRP-linked Antibody	Cell Signaling Technology	Cat# 7076, RRID:AB_330924
goat anti-rabbit IgG Alexa Fluor 647	Thermo Fisher Scientific	Cat# A-21245, RRID:AB_2535813
goat anti-rabbit IgG Alexa Fluor 514	Thermo Fisher Scientific	Cat#A31558, RRID:AB_10375589
goat anti-mouse IgG Alexa Fluor 647	Thermo Fisher Scientific	Cat# A-21235, RRID:AB_2535804
goat anti-mouse IgG Alexa Fluor 514	Thermo Fisher Scientific	Cat# A-31555, RRID:AB_2536171
goat anti-mouse IgG Alexa Fluor 488	Thermo Fisher Scientific	Cat# A-11029, RRID:AB_2534088
goat anti-rabbit IgG Alexa Fluor 568	Thermo Fisher Scientific	Cat# A-11036, RRID:AB_10563566
Chemicals, peptides, and recombinant proteins		
DMSO	Sigma-Aldrich	Cat# D2650
Hydroxyurea	Cayman Chemical	Cat# 23725
AZ-20 (ATRi)	Cayman Chemical	Cat# 17589
MK-1775 (WEE1i)	Cayman Chemical	Cat# 21266
Doxycycline hyclate (Dox)	Sigma-Aldrich	Cat# D9891
MLN-4924	Abcam	Cat# ab216470
indole-3 acetic acid (auxin)	MP Biomedicals	Cat# 0210203705
BMS-650032	Adooq Bioscience	Cat# A112955
L-mimosine	Cayman Chemical	Cat# 14337
Hoechst 33342	Invitrogen	Cat# H3570
5-ethynyl-2’-deoxyuridine (EdU)	Cayman Chemical	Cat# 20518
AFDye 488 picolyl azide	Click Chemistry Tools	Cat# 1276
AFDye 647 picolyl azide	Click Chemistry Tools	Cat# 1300
DAPI	Thermo Fisher Scientific	Cat# 62248
thymidine	Sigma-Aldrich	Cat# T9250-1G
aphidicolin	Sigma-Aldrich	Cat# 5047440001
Recombinant human CMG (CDC45, MCM2, MCM3, MCM4, MCM5, MCM6, MCM7, PSF1, PSF2, PSF3, SLD5)	This study	N/A
Recombinant human Pol ε (POLE1, POLE2, POLE3, POLE4)	This study	N/A
Recombinant human Pol α (POLA1, POLA2, PRIM1, PRIM2)	This study	N/A
Recombinant human Pol δ (POLD1, POLD2, POLD3, POLD4)	This study	N/A
Recombinant human CTF18-RFC (CTF18, CTF8, DCC1, RFC2, RFC3, RFC4, RFC5)	This study	N/A
Recombinant human TIMELESS-TIPIN (TIMELESS, TIPIN)	This study	N/A
Recombinant human AND1	This study	N/A
Recominant human CLASPIN	This study	N/A
Recombinant human PCNA	This study	N/A
Recombinant human RPA (RPA1, RPA2, RPA3)	This study	N/A
Recombinant human CDT1	This study	N/A
Recombinant human Geminin	Abcam	Cat# ab86447
Deposited data		
Data for plotting figures	Dryad (This study)	https://doi.org/10.5061/dryad.4xgxd2599
Original images and uncropped gels	Mendeley Data (This study)	https://doi.org/10.17632/6c63g8jjg7.1
Experimental models: Cell lines		
Human: MCF-10A	ATCC	Cat# CRL-10317, RRID:CVCL_0598
Human: MCF-10A(H2B-Turq +EYFP-PCNA)	This study	N/A
Human: MCF-10A(H2B-Turq + EYFP-PCNA + TetOn-CDT1-mCherry)	This study	N/A
Human: MCF-10A(H2B-Turq + EYFP-PCNA + TetOn-CDT1ΔCy-mCherry)	This study	N/A
Human: MCF-10A(H2B-Turq + EYFP-PCNA + TetOn-CDT1ΔPIP-mCherry)	This study	N/A
Human: MCF-10A(H2B-Turq + C-CRL4^Cdt2^ reporter + APC/C reporter)	This study	N/A
Human: MCF-10A(H2B-Turq + C-CRL4^Cdt2^ reporter + APC/C reporter + TetOn-GemininΔDbox)	This study	N/A
Human: MCF-10A(H2B-Turq + N-CRL4^Cdt2^ reporter + APC/C reporter)	This study	N/A
Human: MCF-10A(H2B-Turq + N-CRL4^Cdt2^ reporter + APC/C reporter + TetOn-ND-CDT1-HA)	This study	N/A
Human: MCF-10A(H2B-Turq + N-CRL4^Cdt2^ reporter + APC/C reporter + TetOn-ND-CDT1 Δ499-546-HA)	This study	N/A
Human: MCF-10A(H2B-Turq + N-CRL4^Cdt2^ reporter + APC/C reporter + TetOn-ND-CDT1R198A/R210A-HA)	This study	N/A
Human: MCF-10A(H2B-Turq + N-CRL4^Cdt2^ reporter + Cyclin E/A-CDK reporter + TetOn-ND-CDT1-HA)	This study	N/A
Human: MCF-10A(H2B-Turq + APC/C reporter + TetOn-CDT1-mCherry)	This study	N/A
Human: MCF-10A(H2B-Turq + APC/C reporter + TetOn-ND-CDT1-mCherry)	This study	N/A
Human: MCF-10A(H2B-Turq + APC/C reporter + Cyclin E/A-CDK reporter)	Meyer Laboratory; Cappell et al.^22^	N/A
Human: MCF-10A(H2B-Turq + APC/C reporter + Cyclin E/A-CDK reporter + CDK4/6 reporter)	Meyer Laboratory; Yang et al.^58^	N/A
Human: MCF-10A(H2B-Turq + N-CRL4^Cdt2^ reporter + APC/C reporter + Cyclin E/A-CDK reporter)	This study	N/A
Human: RPE-1 *TP53^-/-^ CDC6^d/d^*	Laboratory of Arne Lindqvist ; Lemmens et al.^43^	N/A
Human: RPE-1 *TP53^-/-^ CDC6^d/d^* (H2B-Turq + APC/C reporter + TetOn-ND-CDT1-mCherry)	This study	N/A
Human: RPE-1 *TP53^-/-^ CDC6^d/d^* (H2B-Turq + APC/C reporter + TetOn-NLS-mCherry)	This study	N/A
Human: U2OS	ATCC	Cat#HTB-96, RRID:CVCL_0042
Human: U2OS (H2B-Turq + N-CRL4^Cdt2^ reporter + APC/C reporter + Cyclin E/A-CDK reporter)	This study	N/A
Human: U2OS GFP-CDC45	Laboratory of Jiri Lukas; Sedlackova et al.^59^	N/A
Human: U2OS GFP-CDC45(TetOn-ND-CDT1-mCherry)	This study	N/A
Human: U2OS GFP-CDC45(TetOn-ND-CDT1-HA)	This study	N/A
Human: HeLa	ATCC	Cat#CCL-2, RRID:CVCL_0030
Human: HeLa(H2B-Turq + APC/C reporter)	This study	N/A
Oligonucleotides		
siRNA	See Table S1	N/A
Recombinant DNA		
tFucci(CA)2/pCSII-EF (N-CRL4^Cdt2^ reporter + APC/C reporter)	RIKEN BRC: Laboratory of Atsushi Miyawaki; Sakaue-Sawano et al.^15^	Cat# RDB15446
pLV-hCDT1(1-100)ΔCy-mCherry-P2A-mVenus-hGeminin(1-110)-IRES-Blast (C-CRL4^Cdt2^ reporter + APC/C reporter)	This study	Addgene # 193139
pLV-mCherry-hCDT1 (1-100)ΔCy (N-CRL4^Cdt2^ reporter)	This study	Addgene# 193759
CSII-pEF1a-H2B-mTurquoise	Meyer Laboratory; Spencer et al.^40^	N/A
CSII-pEF1a-hDHB(994-1087)-mVenus(Cyclin E/A-CDK reporter)	Meyer Laboratory; Spencer et al.^40^	Addgene# 136461
CSII-pEF1a-mVenus-hGeminin(1-110) (APC/C reporter)	Meyer Laboratory	N/A
pLV-mCherry-hGeminin(1-110)-IRES-Puro(APC/C reporter)	Meyer Laboratory	N/A
pLV-H2B-miRFP670	Meyer Laboratory	N/A
pLV-hDHB(994-1087)-mTurquoise (Cyclin E/A-CDK reporter)	Meyer Laboratory	N/A
pLV-EYFP-PCNA	Meyer Laboratory; Hahn et al.^18^	N/A
pLV-rtTA3-IRES-Puro	Meyer Laboratory	N/A
pLV-TetOn-CDT1-mCherry	This study	Addgene# 193760
pLV-TetOn-ND-CDT1-mCherry	This study	Addgene# 193761
pLV-TetOn-NLS-mCherry	This study	Addgene# 193762
pCW-CDT1-mCherry-Puro	This study	Addgene# 193763
pCW-CDT1 ΔCy-mCherry-Puro	This study	Addgene# 193764
pCW-CDT1 ΔPIP-mCherry-Puro	This study	Addgene# 193765
pCW-ND-CDT1-mCherry-Puro	This study	Addgene# 193766
pCW-ND-CDT1-HA-Puro	This study	Addgene# 193767
pCW-ND-CDT1 Δ499-546-HA-Puro	This study	Addgene# 193768
pCW-ND-CDT1 ΔR198A/R210A-HA-Puro	This study	Addgene# 193769
pCW-GemininΔDbox-HA-Puro	This study	Addgene# 193770
pC1-ND-CDT1-mCitrine	This study	Addgene# 193771
pMDLg/pRRE	Addgene: Laboratory of Didier Trono; Dull etal.^60^	Addgene# 12251
pRSV-rev	Addgene: Laboratory of Didier Trono; Dull etal.^60^	Addgene# 12253
pCMV-VSV-G	Addgene: Laboratory of Bob Weinberg;Stewart et al.^61^	Addgene # 8454
2X FLAG-CDT1-pACEBac1	This study	N/A
Software and algorithms		
Image processing software (MATLAB)	This study (https://github.com/MeyerLab/image-analysis-ratnayeke-2022)	https://doi.org/10.5281/zenodo.7183750
MATLAB R2020a	MathWorks	N/A
ImageJ v1.53c (Fiji distribution)	Schindelin et al.^62^	N/A
QuickFigures (ImageJ plugin)	Mazo 2020	N/A
Other		
96-well glass bottomed plates	Cellvis	Cat# P96-1.5H-N
Lipofectamine 2000	Thermo Fisher Scientific	Cat# 11668019
DharmaFECT 1	Dharmacon	Cat# T-2001-03

### Resource Availability

#### Lead contact

Further information and requests for resources and reagents should be directed to and will be fulfilled by the lead contact, Tobias Meyer (tom4003@med.cornell.edu).

#### Materials availability

Plasmids and cell lines generated in this study are available upon request to lead contact.

### Experimental Model and Subject Details

#### Cell culture

All experiments were performed with MCF10A human mammary epithelial cells (ATCC Cat# CRL-10317, RRID:CVCL_0598) unless otherwise noted. MCF10A cells were cultured in DMEM/F12 growth media with HEPES (Gibco Cat# 11039047), supplemented with 5% horse serum (Gibco Cat# 16050122), 20 ng/mL EGF (PeproTech Cat# AF-100-15), 0.5 μg/mL hydrocortisone (Sigma: H0888), 100 ng/mL cholera toxin (Sigma Cat# C8052) and 10 μg/mL insulin (Sigma Cat# I1882). Cells were passaged using trypsin-EDTA (0.05%, Gibco Cat# 25300054) and trypsin was neutralized in DMEM/F12 supplemented with 20% horse serum. RPE-1 *TP53^-/-^* cells with double-degron endogenous-tagged CDC6 (RPE-1 *TP53^-/-^ CDC6^d/d^*) were a kind gift from Arne Lindqvist^43^ and cultured in DMEM/F12 with HEPES supplemented with 10% FBS (Sigma Cat# TMS-013-B). U2OS cells (ATCC Cat#HTB-96, RRID:CVCL_0042) and U2OS cells with endogenously GFP-tagged CDC45 (a kind gift from Jiri Lukas^59^) were cultured in DMEM growth media (Gibco Cat# 11995065) with 10% FBS. HeLa cells (ATCC Cat#CCL-2, RRID:CVCL_0030) were cultured in DMEM growth media with 10% FBS. For MCF10A serum-starvation prior to mitogen-release, cells were cultured in starvation media (growth media without horse serum, EGF, and insulin and supplemented with 0.3% BSA) after two washes of starvation media. For mitogen-release, starvation media was exchanged with growth media. All cells were cultured at 37°C and 5% CO2. For microscopy experiments, 96-well glass-bottomed plates (Cellvis Cat# P96-1.5H-N) were collagen-coated (Advanced Biomatrix Cat# 5005-B, 60 μg/mL dilution 2 h – 24 h), and cells were seeded into wells at least the night before performing experiments.

#### Cell line generation

Cell cycle reporter cell lines were generated using third-generation lentiviral transduction.^60,61^ In short, lentivirus was produced in HEK-293Tcellsco-transfected with packaging plasmids pMDLg/pRRE (Addgene# 12251, RRID:Addgene_12251), pRSV-rev(Addgene # 1225, RRID:Addgene_12253), and pCMV-VSV-G (Addgene # 8454, RRID:Addgene_8454) together with the lentiviral plasmid with Lipofectamine 2000 (Thermo Cat# 11668019). 72 h after transfection, virus was collected from the supernatant, filtered with a .22 μm filter (Millipore Cat# SCGP00525), and concentrated using 100 kDa centrifugal filters (Millipore Cat# UFC910024). Virus was then transduced into cells in growth media. For constitutively expressed fluorescent constructs, positive fluorescent cells were sorted using a BD Influx cell sorter (performed in Stanford Shared FACS Facility), while Dox-inducible constructs (TetOn in pCW backbone with puromycin selection marker) were selected with 1 μg/mL puromycin until control cells died unless otherwise stated. TetOn cells were grown in the absence of Dox until the time of the experiment unless otherwise stated. See Table S1 for a list of cell lines with reporter combinations.

All MCF10A reporter cell lines were generated from a base cell line transduced with CSII-pEF1a-H2B-mTurquoise or pLV-H2B-miRFP670 as a nuclear tracking marker. Cells with EYFP-PCNA or the APC/C^Cdh1^ reporter were generated by transducing H2B-mTurquoise cells with pLV-EYFP-PCNA or CSII-pEF1a-mVenus-hGeminin(1-110) respectively. Cells containing the APC/C^Cdh1^ reporter together with either N- or C-CRL4^Cdt2^ reporter were generated by transducing H2B-mTurquoise cells with bicistronic vector tFucci(CA)2/pCSII-EF^15^ or pLV-hCDT1(1-100)ΔCy-mCherry-P2A-mVenus-hGeminin(1-110) respectively. Cells with the Cyclin E/A-CDK reporter together with N-CRL4^Cdt2^ were created by transduction of H2B-mTurquoise cells with CSII-pEF1a-hDHB(994-1087)-mVenus and pLV-mCherry-hCDT1(1-100)ΔCy. MCF10A and U2OS cells with the Cyclin E/A-CDK, APC/C^Cdh1^, and N-CRL4^Cdt2^ reporters were generated by transduction of H2B-miRFP670 cells with pLV-hDHB(994-1087)-mTurquoise and tFucci(CA)2/pCSII-EF.

pCW constructs (TetOn Dox-inducible) expressing HA or mCherry-tagged CDT1 mutants or Geminin^ΔDbox^ were introduced into these fluorescent reporter cell lines in combinations found in the Table S1. For cells with the APC/C^Cdh1^ reporter and TetOn-CDT1-mCherry, cells were transduced with CSII-pEF1a-mVenus-hGeminin(1-110), followed by pLV-rtTA3-IRES-Puro, and then pLV-TetOn-CDT1-mCherry. Cell lines transduced with CDT1-mCherry constructs were induced with 500 ng/mL Dox while serum-starved to sort for expressing cells. mCherry positive cells were sorted, and media was then switched to growth media without Dox. MCF10A cells containing a CDK4/6 reporter (not analyzed), Cyclin E/A-CDK reporter, and APC/C^Cdh1^ reporter used in Figures S4B and S4C were described previously.^58^ RPE-1 *TP53^-/-^ CDC6^d/d^* cells were transduced with CSII-pEF1a-H2B-mTurquoise and CSII-pEF1a-mVenus-hGeminin(1-110), and then pLV-TetOn-ND-CDT1-mCherry or pLV-TetOn-NLS-mCherry. U2OS *CDC45-GFP* cells were transduced with pCW-ND-CDT1-mCherry-Puro or pCW-ND-CDT1-HA-Puro. HeLa cells were transduced with CSII-pEF1a-H2B-mTurquoise and pLV-mCherry-hGeminin(1-110)-IRES-Puro.

### Method Details

#### Cell cycle reporters

Cell cycle reporters of CRL4^Cdt2^ and APC/C^Cdh1^ activity were used in this study. These reporters were originally developed as the two components of the FUCCI(CA) reporter system.^15^ The CRL4^Cdt2^ activity reporter corresponds to CDT1(1-100)^ΔCy^ in the FUCCI(CA) system and is based on a fragment of human CDT1 corresponding to the amino acid 1-100, which is inactive with respect to origin licensing. CDT1(1-100)^ΔCy^ contains a PCNA-interacting protein (PIP) degron, which mediates CDT1 degradation by CRL4^Cdt2^ in response to PCNA at fired origins, and has a removed Cy motif to prevent degradation by SCF^Skp2^.^15^ The CRL4^Cdt2^ reporter is rapidly degraded to low levels at S phase start and reaccumulates at the start of G2. Conversely, the APC/C^Cdh1^ reporter corresponds to Geminin^(1-110)^ from the FUCCI(CA) system and is based on amino acids 1-110 of human Geminin fused to a fluorescent protein (either mVenusormCherry). Geminin^(1-110)^ is degraded at anaphase byAPC/C^Cdc20^ and then by APC/C^Cdh1^ throughout G1, and reaccumulates at the time of APC/C^Cdh1^ inactivation at the G1/S transition. Thus, Geminin^(1-110)^ reports APC/C^Cdh1^ activity at the G1/S transition. APC/C^Cdh1^ inactivation represents a commitment point in the cell cycle and typically occurs near the time of S phase entry and DNA replication, though CRL4^Cdt2^ activation in response to origin firing is an explicit measure of S phase entry.^14,15^ While these reporters are typically quantified by their presence or absence in single-timepoint measurements, when reporter fluorescence kinetics are measured in single cells using time-lapse microscopy, the precise time of CRL4^Cdt2^ activation (the start of degradation of the CRL4^Cdt2^ reporter) and APC/C^Cdh1^ inactivation (the stabilization of the APC/C^Cdh1^ reporter) can be identified.

In this study, we used two versions of the CRL4^Cdt2^ reporter, one of which is an N-terminal mCherry-tagged CDT1(1-100)^ΔCy^ (referred to as the N-CRL4^Cdt2^ reporter), which is identical to the construct used in the FUCCI(CA) reporter system. Since the N-CRL4^Cdt2^ reporter is fused to a fluorescent protein on the N-terminus, the PIP degron is in the middle of the construct. Since CDT1 naturally has an N-terminal PIP degron, we hypothesized that reversing the order of the fluorescent protein fusion in a reporter could confer a faster response to the initial origins that are fired in early S phase. As a result, we created a C-terminally tagged CDT1(1-100)^ΔCy^ (referred to as the C-CRL4^Cdt2^ reporter). We found that C-CRL4^Cdt2^ responds with slightly faster kinetics at S phase start than N-CRL4^Cdt2^ (Figure S1H), which was necessary for looking within the first 15-30 min of S phase by RT-QIBC in Figures 1 and 4. However, both reporters are well suited for RT-QIBC looking at times after the first 15-30 min of S phase, and we use both reporters in this study. C-CRL4^Cdt2^ has a similar orientation to the PIP-FUCCI cell cycle reporter,^14^ which is based on CDT1(1-17) and is also degraded throughout S phase. We consider the N-CRL4^Cdt2^ (originally used in the FUCCI(CA)) system, C-CRL4^Cdt2^, and PIP-FUCCI reporters to all be reporters of CRL4^Cdt2^ activity and should all be suitable for use with RT-QIBC.

The cyclin E/A-CDK reporter is a translocation-based reporter that is phosphorylated by cyclin E or A complexed with CDK2 or CDK1 (referred to collectively as cyclin E/A-CDK).^40^ It is based on a fragment of human DNA helicase B (amino acids 994-1087), which is phosphorylated by cyclin E/A-CDK. When unphosphorylated in G0 and early G1, this reporter is localized in the nucleus, and as cyclin E/A-CDK activity increases throughout the cell cycle, the reporter becomes progressively localized to the cytoplasm due to increased phosphorylation. Thus, the cytoplasm to nuclear ratio of intensity is a readout of cyclin E/A-CDK activity. Critically, the increase in cyclin E/A-CDK activity in G1 is the result of increasing Cyclin E activity, rather than Cyclin A, which isonly present after APC/C^Cdh1^ inactivation when it is stabilized (Figure S5I).

#### Plasmid generation

Plasmids generated in this study were assembled using Gibson assembly of PCR amplified inserts and restriction enzyme digested plasmid backbones. Human full-length CDT1 was amplified out of MCF10A cDNA for CDT1 overexpression, and mutations and tags were introduced through primers or gene synthesis (IDT). ND-CDT1 constructs were created through a truncation of wild-type CDT1 (aa20-546), which removes the CDT1 PIP degron. The CDT1 Cy motif (aa68-70 of full-length CDT1) was mutated to alanine (ΔCy) to prevent degradation by SCF^Skp2^. This sequence was fused at the C-terminus to a flexible linker, SV40 NLS, and either an mCherry or HA tag. For the CDT1^ΔPIP^ -mCherry construct, CDT1 (aa20-546) was fused to a flexible linker and mCherry at the C-terminus. For CDT1^ΔCy^ -mCherry construct, full-length CDT1 with the Cy motif mutated to alanine was fused to a flexible linker and mCherry at the C-terminus. ND-CDT1^Δ499-546^ was generated from ND-CDT1 and had a truncating mutation which removed the C-terminal amino acids corresponding to residues 499-546 of CDT1. ND-CDT1^R198A/R210A^ was generated from ND-CDT1 and had alanine mutations introduced at amino acids corresponding to residues 198 and 210 in CDT1. The Geminin^ΔDbox^ (human Geminin with R23A and L26A mutations) sequence was generated using gene synthesis and HA-tagged. For Dox-inducible TetOn constructs, PCR products were inserted into the pCW backbone (derived from pCW-Cas9, a gift from Eric Lander & David Sabatini, Addgene plasmid # 50661, RRID:Addgene_50661), a bicistronic vector with a TetOn promoter driving gene expression in addition to a constitutive PGK promoter-driven PuroR-T2A-rtTA. pC1-ND-CDT1-mCitrine was created by cloning ND-CDT1 into the pC1 backbone, derived from C1-F-tractin-mCitrine.^63^ pLV-hCDT1(1-100)ΔCy-mCherry-P2A-mVenus-hGeminin(1-110) was generated from full-length CDT1 and Geminin (Human ORFeome V5.1). The N-CRL4^Cdt2^ reporter was amplified from tFucci(CA)2/pCSII-EF, and inserted into the pLV backbone to generate pLV-mCherry-hCDT1(1-100)ΔCy. pLV, CSII and pCW are lentiviral expression plasmids, while pC1 is a mammalian expression plasmid.

#### siRNA and plasmid transfection

MCF10A cells were transfected with siRNA using DharmaFECT 1 (Dharmacon Cat# T-2001-03) according to the manufacturer’s protocol using 20 nM siRNA and 1:500 diluted DharmaFECT 1 final concentration unless otherwise stated. Cells were incubated in transfection mixture for 6-24 h in either growth or serum-starvation media, followed by a media change. Pools of 3-4 siRNA oligos (ON-TARGETplus, Dharmacon) were used for siCtrl, siCDT1, siGeminin (siGMNN), siCyclin A (siCCNA2) and si-p21(siCDKN1A). For siCDT1 and siGeminin, siRNAs that do not target hCDT1(1-100) and hGeminin(1-110) were selected to avoid knockdown of the CRL4^Cdt2^ and APC/C^Cdh1^ reporters, respectively. CCNA2 was knocked down since CCNA1 is not expressed in our cell lines. For siGeminin and siCyclin A in cycling cells (Figures 3A, 3B, S5I, and S5J), cells were transfected for 4–6 h and then immediately live imaged. These proteins can be suppressed even with short siRNA treatment due to their rapid protein degradation starting at anaphase. A list of siRNA oligos is in Table S1. HeLa cells were transiently transfected with pC1-ND-CDT1-mCitrine plasmid using Lipofectamine 2000 according to the manufacturer’s protocol using 2 ng/μL final concentration of plasmid complexed with 1:400 diluted Lipofectamine 2000 final concentration. Media was exchanged with growth media after 2 h, and cells were then immediately live-imaged. siRNA and plasmids were both complexed in Opti-MEM serum-free media (Gibco Cat# 31985070).

#### Drugs

Stock solutions of drugs were dissolved in DMSO (Sigma-Aldrich Cat# D2650) and used at the given working concentration unless otherwise stated: 2 mM hydroxyurea (HU, dissolved in water, Cayman Chemical Cat# 23725), 2 mM thymidine (dissolved in PBS at 100 mM, Sigma-Aldrich Cat#T9250-1G), 2 mg/mLaphidicolin (Sigma-Aldrich Cat# 5047440001), 2 μM AZ-20 (ATRi, Cayman Chemical Cat# 17589), 1 μM MK-1775 (WEE1i, Cayman Chemical Cat# 21266), 1 μg/mL Doxycycline hyclate (Sigma-Aldrich Cat# D9891), 2 μM MLN-4924 (Abcam Cat# ab216470), 500 μM indole-3 acetic acid (auxin, MP Biomedicals Cat# 0210203705), 100 nM BMS-650032 (Adooq Bioscience Cat# A112955), 500 μM L-mimosine (20x stock solution dissolved in DMEM/F12, Cayman Chemical Cat# 14337). For release from mimosine arrest, cells were washed three times in growth media. For all experiments where drugs or Doxycycline were added to cells, DMSO (vehicle) was added to control cells, with the exception of HU, which was dissolved in water.

##### *In vitro* replication assays

###### Protein expression

Proteins used in in vitro replication and primer extension assays were expressed and purified as previously described.^49,64^ Recombinant Geminin was purchased from Abcam (Cat# ab86447). The sequence for the expression of N-terminal 2X FLAG-tagged CDT1 (FLAG-tag: MDYKDDDGDYKDDD) was codon optimized for expression in insect cells and synthesized by Epoch Life Science Gene Synthesis. To prepare baculovirus for CDT1 expression, the 2X FLAG-CDT1-pACEBac1 construct was transformed into EMBacY *E. coli* competent cells for bacmid generation. Isolated bacmid was transfected into Sf9 cells using FuGENE® HD (Promega Cat# E2311). Baculovirus was amplified, and the resulting virus was used to infect 1 L of Hi5 cells at a density of 1x 10^6^ / mL. Cells were harvested on day 3 after infection.

###### CDT1 purification

The cell pellet obtained from 1 L of baculovirus-infected culture was resuspended in lysis buffer (50 mM Tris-HCl pH 7.5, 10% glycerol, 0.05% NP-40,1 mM EDTA, 1 mM DTT) + protease inhibitors (cOmplete, EDTA-free, Roche; one tablet per 50 mL buffer, Sigma-Aldrich Cat# 11873580001). Cells were lysed by dounce homogenization, and insoluble material was removed by centrifugation (235,000*g* at 4 °C for 45 min). 2 mL Anti-FLAG M2 affinity gel (Sigma-Aldrich Cat#A2220) was added to the supernatant and incubated for 3 h at 4 °C. Resin was collected in a disposable gravity flow column (Bio-Rad) and was washed with 100 mL lysis buffer. CDT1 was eluted in 2 mL lysis buffer + 0.4 mg/mL 3× FLAG peptide and 4 mL buffer + 0.2 mg/mL 3× FLAG peptide. Eluates were pooled and applied to 1 mL HiTrap heparin column equilibrated in PBS (137 mM NaCl, 2.7 mM KCl, 10 mM Na_2_HPO_4_, and 1.8 mM KH_2_PO_4_) pH 7.5,0.5 mM TCEP, 10% glycerol. CDT1 was eluted using a 40 column volume gradient to 1 M NaCl. Peak fractions were pooled, frozen in liquid nitrogen, and stored at -80°C.

###### DNA replication assays

Experiments were performed as previously described.^49^ Reactions were conducted in replication buffer (25 mM HEPES-KOH (pH 7.6), 0.01% NP-40, 100 mM potassium glutamate, 1 mM DTT, 10 mM Mg(OAc)_2_and 0.1 mg ml^−1^ BSA) at 37°C. Protein and nucleotide concentrations in the final reactions were: 1 nM DNA, 25 nM CMG, 20 nM Pol ε, 20 nM RFC, 20 nM PCNA, 20 nM AND-1, 10 nM Pol α, 5 nM Pol δ, 100 nM RPA, 20 nM CLASPIN, 20 nM TIMELESS–TIPIN, 10 nM CDT1, 10 nM Geminin, 20 nM CTF18—RFC, 4 mM ATP, 30 μM dC/dT/dG/dATP, 200 μM C/G/UTPand 33 nM α-[^32^ P]-dCTP. 2 nM 9.7 kbp or 15.8 kbp linear forked DNA template^49^ was incubated with 50 nM CMG for 10 min in replication buffer. The reaction was diluted two-fold by the addition of replication buffer containing dA/dCTP and, as indicated in the figures, PCNA, RFC, Pol ε, Pol α, CLASPIN, TIM–TIPIN, AND-1, CTF18–RFC, and Pol δ. Replication was initiated by addition of a 10x solution containing ATP, dTTP, GTP, CTP, UTP, dGTP, α-[^32^P]-dCTP and RPA. In reactions containing Pol δ, RFC was added when replication was initiated. Where indicated in the figure legends, 10 nM CDT1 and 10 nM Geminin were pre-incubated on ice for 5 min before addition to the replication reaction. Reactions were stopped by the addition of 50 mM EDTA. Post-reaction processing was performed as previously detailed.^49^

###### Primer extension assay

Experiments were conducted as previously described.^49^ Where indicated in the figure, 10 nM CDT1 was pre-incubated with 10 nM Geminin, and the protein mix was added to the reaction before initiating primer extension by the addition of Pol ε.

###### Pulse-chase experiments

Experiments were performed as previously described.^49^ Replication reaction conditions were applied, except that the concentration of dCTP in the pulse was reduced to 3 μM. The concentrations of dCTP, dGTP, dATP, and dTTP were increased to 600 μM in the chase. The chase was added after 50 s.

#### Western blot

Cells were grown in 6-well plates. At the time of lysis, cells were washed in ice-cold PBS, lysed in 2x Laemmli sample buffer with 100 mM DTT and a cell scraper, passed through a 25G needle 10 times, and heated at 90°C for 5 min. Samples were then separated with SDS-PAGE using 7.5% or 4-20% Mini-PROTEAN TGX gels (Bio-Rad Cat# 4561025, 4561095) in Tris/Glycine/SDS running buffer (Bio-Rad Cat#161-0772). Proteins were transferred to membranes by semi-dry transfer (Bio-Rad Trans-Blot SD, Cat# 1703940) onto 0.45 μm PVDF membranes (Millipore Cat# IPFL00010) with Tris/Glycine buffer (Bio-Rad Cat# 1610734) + 10% MeOH or semi-dry transfer (Bio-Rad Trans-Blot Turbo Cat# 1704150) onto 0.2 μm PVDF membranes with Trans-Blot Turbo Transfer Packs (Bio-Rad Cat# 1704156). Membranes were washed in TBST (20 mM Tris, pH 7.5, 150 mM NaCI, 0.1% Tween 20), blocked for 30 min in 5% milk in TBST, and incubated overnight with primary antibodies in 5% BSA + 0.01% NaN_3_ in TBST. Primary antibodies used were mouse anti-CDC6 antibody (1:500, Santa Cruz Biotech. Cat# sc-9964, RRID:AB_627236), rabbit anti-GAPDH (1:1000, CST Cat# 5174, RRID:AB_10622025), rabbit anti-p21(1:1000, CST Cat# 2947, RRID:AB_823586), rabbit anti-CDT1 (1:1000, Abcam Cat# ab202067, RRID:AB_2651122), rabbit anti-Geminin(1:1000, Proteintech Cat# 10802-1-AP, RRID:AB_2110945) and mouse anti-GAPDH (1:2000, Santa Cruz Biotechnology Cat# sc-32233, RRID:AB_627679). Membranes were then incubated in anti-rabbit or anti-mouse HRP secondary antibodies (1:5000, CST Cat# 7074, RRID:AB_2099233 or CST Cat# 7076, RRID:AB_330924) for 30 min in 5% BSA + 0.01% NaN_3_ in TBST, treated with chemiluminescent substrate (Thermo Cat # 34580) and detected on film (Thomas Sci. Cat# EK-5130) and scanned, or imaged directly using a Licor Odyssey Fc. For membrane reblotting (with antibodies of different species), membranes were washed in TBST and treated as in the first round, starting with blocking.

#### Fixed-cell sample preparation

##### General protocol

Staining and imaging were performed in 96-well glass-bottomed plates (Cellvis Cat# P96-1.5H-N). Cells were fixed in 4% paraformaldehyde in PBS (diluted from Fisher Cat# NC1537886) for 10 min at room temperature, followed by PBS wash. If cells expressed fluorescent proteins which spectrally overlapped with the fluorophores used in later steps, the fluorescent proteins were chemically bleached^65^ in 3% H_2_O_2_+ 20 mM HCI in PBS for 1 h, washed in PBS, and checked under a microscope to ensure there was negligible residual signal. If fluorescent proteins needed to be quantified in fixed cells prior to immunofluorescence, cells were initially imaged before bleaching and reimaged after staining. For PCNA and CDC45 staining, cells were incubated in ice-cold methanol for 15 min after fixation and then washed in PBS. Cells were permeabilized in 0.2% Triton X-100 in PBS for 10 min and then blocked in blocking buffer A (10% FBS, 1% BSA, 0.1% Triton X-100, 0.01% NaN_3_in PBS)for1 h. Cells were then incubated with primary antibodies overnight in blocking buffer A at 4°C, washed in PBS, and then incubated with secondary antibodies in blocking buffer A for 1 hat RT. Cells were washed with PBS and then incubated in 1 μg/mL Hoechst 33342 (Invitrogen Cat# H3570) or in 1 μg/mL DAPI (Thermo Cat#62248) in PBS for 10 min, followed by a final PBS wash prior to imaging. Unless otherwise stated, all washes were done with an automated plate washer (aspirate to 50 μL, dispense 250 μL, repeated 9 times, BioTek 405 LS) or by hand (for pre-extracted samples, 3 washes aspirating all liquid).

##### Iterative immunofluorescence

If simultaneously staining for targets with antibodies of the same species, the iterative indirect immunofluorescence imaging (4i) technique was used to sequentially image multiple antibodies.^66^ In short, the first round of imaging was identical to the general immunofluorescence protocol, with the exception that cells after the post-Hoechst PBS wash were washed in ddH_2_O and then placed in imaging buffer (700 mM N-acetyl cysteine in ddH_2_O, pH 7.4, Sigma-Aldrich A7250). Cells were imaged and then washed in ddH_2_O. The prior-round antibodies were eluted by 3× 10-min incubations in elution buffer, which consists of 0.5M glycine (Sigma-Aldrich), 3M urea (Sigma-Aldrich), 3M guanidinium chloride (Sigma-Aldrich) and 70 mM TCEP-HCI (Goldbio Cat#TCEP50) in ddH_2_O, pH 2.5, followed by a PBS wash. Cells were then checked under a fluorescence microscope to ensure proper elution. Cells were then blocked with blocking buffer B, consisting of 1% BSA in PBS supplemented with 150 mM maleimide (dissolved just prior to use, Sigma-Aldrich Cat# 129585) for 1 h and then washed in PBS. Cells were then blocked with blocking buffer A for 30 min, followed by primary antibody incubation, and the subsequent steps were the same as in the first round, repeated as needed. Control wells leaving out primary antibodies were always included to ensure there was no residual signal from prior rounds of imaging.

##### Pre-extraction for chromatin-bound protein

If chromatin-bound proteins were being stained for, soluble proteins were extracted from cells. Just prior to fixation, media was aspirated off cells, and the plate was placed on ice. Cells were incubated in ice-cold pre-extraction buffer, consisting of 0.2% Triton X-100 (Sigma-Aldrich Cat# x100) + 1x Halt Protease Inhibitor Cocktail (Thermo Cat# 78439) in the selected aqueous buffer. For all proteins pre-extracted except RPA1, pre-extraction buffer was made with PBS, while CSK buffer was used for RPA1, consisting of 10 mM PIPES (Sigma-Aldrich), 100 mM NaCl (Sigma-Aldrich), 300 mM sucrose (Sigma-Aldrich), 3 mM MgCl_2_ (Sigma-Aldrich) at pH 7.0. After a set extraction time, 8% PFAin H_2_O wasdirectly added to wells 1:1 with wide-orifice tips to minimize cell detachment, and cells were fixed for 25 min at room temperature, after which the sample was treated with the general staining protocol. Extraction times were: 4-5 min (PCNA, MCM2, TIMELESS), 3 min (POLA2, POLD2, POLE2), and 2 min (ND-CDT1 and CDC45-GFP in U2OS SoRa imaging and MCM2 in RPE-1).

##### EdU incorporation and labeling

If measuring 5-ethynyl-2'-deoxyuridine (EdU) incorporation, cells were pulsed with 50 μM EdU (Cayman Chemical Cat# 20518) in growth media for 8 min prior to fixation and pre-extraction, unless otherwise stated. EdU is incorporated throughout the EdU pulse, such that incorporated EdU reflects the average rate of DNA synthesis over the length of the pulse. Thus an 8 min short EdU pulse is more reflective of the instantaneous DNA synthesis rate compared to a longer pulse such as 1 h. After blocking cells (prior to primary antibodies), cells were washed once with PBS, and then a click reaction^67^ was performed in 2 mM CuSO_4_, 20 mg/mL sodium ascorbate in TBS (Tris 50 mM, NaCl 150 mM pH 8.3) with 3 μM AFDye 488 picolyl azide (Click Chemistry Tools Cat# 1276) or AFDye 647 picolyl azide (Click Chemistry Tools Cat# 1300) for 30 min, followed by a PBS wash.

##### Antibodies

The following primary antibodies were used for immunofluorescence: rabbit anti-CDT1 (1:500, Abcam Cat# ab202067, RRID:AB_2651122), rabbit anti-Geminin (1:800, Atlas Antibodies Cat# HPA049977, RRID:AB_2680978), mouse anti-Cyclin A (1:250, Santa Cruz Biotech Cat# sc-271682, RRID:AB_10709300), mouse anti-PCNA (1:200, Santa Cruz Biotech. Cat# sc-56, RRID:AB_628110), rabbit anti-MCM2 (1:800, CST Cat# 3619, RRID:AB_2142137), rabbit anti-p21 (1:2500, CST Cat# 2947, RRID:AB_823586), rabbit anti-p27 (1:1600, Cell Signaling Technology Cat# 3686, RRID:AB_2077850), rabbit anti-HA tag (1:1000, CST Cat# 3724, RRID:AB_1549585), mouse anti-GFP (1:500, Abcam Cat# ab1218, RRID:AB_298911), rabbit anti-CDC45 (1:100, CST Cat# 11881, RRID:AB_2715569), rabbit anti-POLA2 (1:100, Atlas Antibodies Cat# HPA037570, RRID:AB_10672280), rabbit anti-POLD2 (1:100, Atlas Antibodies Cat# HPA026745, RRID:AB_1855520), rabbit anti-POLE2 (1:100, Atlas Antibodies Cat# HPA027555, RRID:AB_10610282), rabbit anti-Timeless (1:800, Abcam Cat# ab109512, RRID:AB_10863023), rabbit anti-phospho-Chk1(S317) (1:500, CST Cat# 12302, RRID:AB_2783865), rabbit anti-phospho-Chk2(T68) (1:200, CST Cat# 2661, RRID:AB_331479), rabbit anti-phospho-histone H2A.X(S139) (1:500, CST Cat# 2577, RRID:AB_2118010), rabbit anti-RPA70/RPA1 (1:200, Abcam Cat# ab79398, RRID:AB_1603759). The epitopes for anti-CDT1 and anti-Geminin antibodies do not detect hCDT1(1-100)^ΔCy^ and hGeminin(1-110) of the CRL4^Cdt2^ and APC/C^Cdh1^ reporters. For secondary antibodies, antibodies targeting the appropriate species and with no spectral overlap were selected from the following and diluted 1:1000: goat anti-rabbit IgG Alexa Fluor 647 (Thermo Cat# A-21245, RRID:AB_2535813), goat anti-rabbit IgG Alexa Fluor 514 (Thermo Cat#A31558, RRID:AB_10375589), goat anti-mouse IgG Alexa Fluor 647 (Thermo Cat# A-21235, RRID:AB_2535804), goat anti-mouse IgG Alexa Fluor 514 (Thermo Cat# A-31555, RRID:AB_2536171), goat anti-mouse IgG Alexa Fluor 488 (Thermo Cat# A-11029, RRID:AB_2534088), goat anti-rabbit IgG Alexa Fluor 568 (Thermo Cat# A-11036, RRID:AB_10563566)

#### Microscopy

##### Time-lapse imaging, RT-QIBC, and QIBC

For automated epifluorescence microscopy, cells were imaged using a Ti2-E inverted microscope (Nikon) or ImageXpress Micro XLS microscope (Molecular Devices). For imaging on the Ti2-E, multichannel fluorescent images were acquired using triple-band (ECFP/ EYFP/mCherry, Chroma: 89006) or quad-band (DAPI/FITC/TRITC/Cy5, Chroma: 89402) Sedat filter sets using an LED light source (Lumencor Spectra X) and Hamamatsu ORCA-Flash4.0 V3 sCMOS camera. 10x (Nikon CFI Plan Apo Lambda, NA 0.45) or 20x (Nikon CFI Plan Apo Lambda, 0.75 NA) objectives were used to acquire images. For imaging on the ImageXpress, images were taken with appropriate single-band filter sets with a white-light source, using a 10x (Nikon CFI Plan Fluor, NA 0.3) or 20x (Nikon CFI Plan Apo Lambda, 0.75 NA) and Andor Zyla 4.2 sCMOS camera. All images were acquired in 16-bit mode, and acquisition settings were chosen to not saturate the signal. Fluorophores and imaging channels were chosen to minimize bleedthrough, and in the case of detectable bleedthrough, it was corrected using bleedthrough coefficients estimated from single fluorophore controls.

For live-cell time-lapse imaging, 96-well plates were imaged within an enclosed 37°C, 5% CO2 environmental chamber in 200 μL of growth media. 4-9 sites were imaged in each well (with the number of wells imaged varying depending on experiment and imaging interval) every 3-12 min (3 min for measurements of CDT1 in early S phase as in Figure 1E, or in Figure S1E or S1I. Longer intervals for measuring longer-term dynamics). Light exposure to cells was limited by using the minimum exposure necessary to maintain an acceptable signal-to-noise ratio on a per-channel basis, and total light exposure was always limited to below 300 ms per site each timepoint. Images were taken with the 10x objective for all live-cell imaging except for experiments shown in Figures 3A, S1E, S5H, and S1I to maximize the number of cells in the field of view. When performing the live-cell imaging for RT-QIBC, cells were immediately taken off the microscope following the final time point and fixed.

For fixed-cell imaging for RT-QIBC and QIBC, tiled images of the majority of each well (16–36 sites per well) were taken using the 20x objective. When reimaging fixed cells (matching back to either live-cell imaging for RT-QIBC or previous rounds of fixed-cell imaging), the plate position (which can shift slightly when replacing the plate on the microscope) was aligned to approximately the same location and further aligned computationally during image analysis.

##### Spinning-disk confocal microscopy

For high-resolution live-cell imaging of EYFP-PCNA and CDT1-mCherry (Figures 1B and S1A-S1C), cells were imaged on an automated spinning-disk confocal microscope (Intelligent Imaging Innovations, 3i). This system used a Nikon Ti-E stand, motorized XY stage with piezoelectric Z movement (3i), AndorZyla 4.2 sCMOS camera, CSU-W1 confocal scanner unit (Yokogawa), and 405/445/488/514/561/640 nm LaserStack (3i), controlled using SlideBook 6 (3i). Cells were imaged in a 37°C environmental chamber (growth media was HEPES buffered) using a 60x/1.35NA oil objective (Nikon) with 2x camera binning. Images at the nucleus midplane were taken every 2-3 min in a 5x5 montage which was stitched together after acquisition. H2B-mTurquoise, EYFP-PCNA, and CDT1-mCherry were imaged using a triple-band 445/515/561 excitation filter set.

For fixed-cell imaging of chromatin-bound ND-CDT1 localization, pre-extracted U2OS CDC45-GFP cells were imaged on a SoRa spinning-disk confocal microscope (Marianas system, 3i). This system was similar to the previously described 3i microscope, except it used a Zeiss Axio Observer 7 stand, ORCA-Fusion BT sCMOS Camera (Hamamatsu), CSU-W1 SoRa confocal scanner unit (Yokogawa), and 405/445/488/514/561/637 nm LaserStack (3i). Cells were stained using rabbit anti-HA and anti-rabbit Alexa Fluor 568 (for detecting ND-CDT1) together with mouse anti-GFP and anti-mouse Alexa Fluor 488 to image endogenously tagged CDC45-GFP, together with Hoechst. Images were taken using the 405, 488, and 561 channels using a quad-band 405/488/561/640 nm excitation filter set (3i), with a 63x/1.4NA Plan-Apochromat Oil M27 objective (Zeiss) and 4x magnification changer and no camera binning. The field of view was manually searched without 4x magnification and low exposure in the 488 channel to identify cells that were in S phase. Cells were then imaged in both channels at 5 Z-positions around the midplane of the nucleus (1 μm spaced, only the midplane is shown). No deconvolution was performed, and controls were tested to ensure there was no spectral bleedthrough or cross-binding of secondary antibodies.

#### Experimental details

##### MCF10A mitogen-release experiments

For mitogen-release experiments, MCF10A cells were serum-starved the day following seeding onto plates (Day 1). For experiments with siRNA knockdowns, cells were transfected with siRNA the next day (Day 2) in serum-free media. Media was changed the following day (Day 3, approximately 48 h after serum-starvation). If inducing ND-CDT1, Dox was added during the media change 6 h before mitogen release. On Day 3, cells were mitogen-released with full growth media and live-imaged. For RT-QIBC and QIBC, cells were fixed 18 h after mitogen-release. For drug treatments (hydroxyurea, AZ-20, MK-1775, MLN-4924), drugs were added 14 h after mitogen-release during live-cell imaging.

##### CDC6 degradation experiments

RPE-1 *TP53^-/-^ CDC6^d/d^* cells^43^ had an auxin-inducible degron (mAID), and SMASh-tag knocked into both endogenous *CDC6* loci. Cells contained a Dox-inducible OsTIR1 E3 ubiquitin ligase component which is required for mAID degradation. The addition of auxin to cells triggers the degradation of mAID-containing proteins. The SMASh-tag contains degron that is auto-cleaved after protein translation by a protease domain. Addition of BMS-650032 (BMS) inhibits this auto-cleavage, resulting in protein degradation. Thus, addition of Dox/Auxin/BMS triggers a robust degradation of endogenous CDC6.

For Figures 5F-5H, Dox-inducible ND-CDT1-mCherry or NLS-mCherry were introduced into RPE-1 *TP53^-/-^ CDC6^d/d^* cells with the APC/C^Cdh1^ reporter. Cells were mitogen-released in the presence of mimosine and Dox for 18 h. CDC6 was then degraded by adding auxin and BMS-650032 (BMS) for 4 h, and then cells were released from mimosine arrest for 1.5 h, followed by an EdU pulse and fixation. Control unreleased cells were not released from mimosine arrest.

##### Protein nomenclature

For simplicity, we refer to several human proteins by their colloquial names. Namely, we refer to CDT1 (encoded by *CDT1* gene), Geminin (encoded by *GMNN* gene), Cyclin A (in our cell lines, only Cyclin A2, encoded by *CCNA2* gene, is expressed), CRL4^Cdt2^ (Cdt2 is encoded by *DTL* gene), APC/C^Cdh1^ (Cdh1 is encoded by *FZR1* gene), SCF^Skp2^ (aIso known as CRL1^Skp2^, Skp2 is encoded by *SKP2* gene), CDC6 (encoded by *CDC6* gene), Claspin (encoded by *CLSPN* gene), AND1 (encoded by *WDHD1* gene), CTF18 (encoded by *CHTF18* gene), Chk1 (encoded by *CHEK1* gene), Chk2 (encoded by *CHEK2* gene), p21 (encoded by *CDKN1A* gene), p27 (encoded by *CDKN1B* gene), p53 (encoded by *TP53* gene) and Cyclin E (both Cyclin E1 and E2, encoded by *CCNE1* and *CCNE2* genes). Furthermore, we measure several protein complexes through an individual subunit (all of which are constitutive complexes): DNA polymerases epsilon (measured by subunit POLE2), alpha (measured by subunit POLA2) and delta (measured by subunit POLD2), and RPA (measured by subunit RPA1, also known as RPA70). Furthermore, we use common acronyms for protein complexes defined as follows: CMG (CMG-MCM2-7-GINS), MCM (minichromosome maintenance), Pol α (DNA polymerase alpha), Pol δ (DNA polymerase delta), Pol ε (DNA polymerase epsilon), CRL4 (cullin-RING ubiquitin ligase complex 4), CRL1 (cullin-RING ubiquitin ligase complex 1), SCF (SKP1-CUL1-F-box), CDK (cyclin-dependent kinase), APC/C^Cdh1^ (anaphase-promoting complex/cyclosome with subunit Cdh1), PCNA (proliferating cell nuclear antigen), RPA (replication protein A), CTF18-RFC (RFC: replication factor C, CTF18-RFC refers to RFC containing CTF18), and TIM-TIPIN (TIMELESS, TIPIN complex).

### Quantification and Statistical Analysis

#### Image analysis

Automated analysis of time-lapse imaging of cell cycle reporters, quantitative image-based cytometry (QIBC), and Retrospective Time-lapse Synchronized QIBC (RT-QIBC) were performed using a custom MATLAB (R2020a, MathWorks) pipeline based on previous work.^22^ QIBC here is considered to be the high-throughput single-cell quantification of fixed-cell signals (fluorescent proteins, immunofluorescence, EdU staining, DNA stain), while RT-QIBC involves the assignment of QIBC measurements to an explicit time in the cell cycle based on prior time-lapse imaging of cell cycle reporters (N- and C-CRL4^Cdt2^ reporters, APC/C^Cdh1^ reporter, and EYFP-PCNA). In principle, RT-QIBC can be used to quantify any fixed-cell signal (immunofluorescence used in this study, as well as mRNA or DNA FISH, for example) and retrospectively analyze any live-cell reporter or imaging measurements. In this way, the dynamics of fixed cell measurements can be reconstructed with temporal resolution that is only limited by imaging frequency (in this study 3-12 min). The image processing pipeline and code used to generate all figures in this study have been deposited on Github and Zenodo (https://github.com/MeyerLab/image-analysis-ratnayeke-2022, https://doi.org/10.5281/zenodo.7183750), and data can be downloaded at Dryad (https://doi.org/10.5061/dryad.4xgxd2599).

##### Segmentation and signal quantification

Raw images were flat-field corrected (also known as shading corrected) to correct for uneven sample illumination. Since images output by the sCMOS camera are the sum of a camera offset value together with the actual detected signal (which is proportional to the sample illumination), we subtracted off the camera offset value and then divided the image by an empirically determined illumination profile. This profile was calculated either from the background autofluorescence in areas without cells in live-cell images (aggregated over a large number of sites) or from sample-free wells filled with autofluorescent blocking buffer A for fixed cell imaging. Confocal movies were not flat-field corrected due to a lack of uneven illumination.

For live-cell imaging, nuclei were automatically segmented from H2B signal using a Laplacian of Gaussian blob detector, which in the case of movies with low contrast, was further refined with active contours. For fixed-cell imaging, nuclei were segmented from the Hoechst or DAPI stain using a threshold determined from histogram curvature. Detected nuclei larger than the median object size were checked using a curvature-based object splitting algorithm that splits cells along two points of high perimeter curvature. If there are more than two putative split points, pairs of points are chosen based on pairs with the highest distance along the perimeter between points divided by the Euclidean distance of the points. For multi-round fixed cell imaging, each imaging round was segmented and aligned to a common round of images. A segmentation mask from a single round (typically the first round) was designated the primary mask and used for quantification of all rounds.

To quantify nuclear cell cycle reporters and fixed-cell signals, the background signal was estimated by taking the 25^th^ percentile of pixels outside of a dilated nuclear mask (dilated 7.8 μm for predominantly nuclear signals, 15.6 μm for signals with cytoplasmic components) and subtracted off of images. For chromatin-bound CDC45, POLA2, POLD2, POLE2, and TIMELESS signals, the background was not subtracted during image processing but accounted for later during analysis. The mean and median signal within the nucleus were then calculated, and for signals with a cytoplasmic component, the median signal within a ring outside of the nucleus was calculated (region 0.65 μm to 3.25 μm outside the edge of the nucleus). To quantify the puncta area of EYFP-PCNA, a tophat filter (3 pixel radius for confocal imaging, 2 pixel radius for wide-field) was applied to the image, and a series of thresholds of different stringencies were manually chosen and applied to minimize false positives and negatives. The total area of pixels above the thresholds was quantified.

##### Time-lapse tracking

For time-lapse imaging, nuclei were tracked using a nearest-neighbor algorithm between each frame and its previous frame. To increase tracking fidelity, the total nuclear signal (the sum of nuclear intensity) was used as an invariant quantity which does not change significantly between frames. Using this, putative aberrant merging and splitting of nuclei during segmentation could be detected and corrected. Mitotic events are detected when two daughter nuclei are detected within the vicinity of a previous nucleus and have a total nuclear signal which is approximately equal to the previous nucleus.

To match fixed-cells to live cells, fixed-cell images were computationally aligned to live-cell images using 2D cross-correlation, and cells with their associated measurements were assigned to their nearest live-cell neighbor. When matching the 20x fixed-cell images back to 10x live-cell images, live images were resized using bicubic interpolation (for alignment and tracking purposes only), or fixed images were mean-value binned.

##### Cell cycle annotation of live-cell data

Mitosis was annotated during the process of tracking cells, defined as the separation of the two sets of chromosomes at anaphase. CRL4^Cdt2^ activation (defined as the start of CRL4^Cdt2^ reporter degradation) and CDT1-mCherry degradation start were annotated by subjecting traces following mitosis or mitogen-release to a drop detection algorithm. This algorithm detects degradation of the reporter at a given time based on a set number of frames following it (the number of frames after a corresponds to the minimum detectable time since degradation start, typically ~3 frames). By detecting points using only a set number of frames beyond the degradation point, we avoid biases in the accuracy of detecting cells that just recently degraded compared to cells that degraded much earlier. Points were checked based on the slope and curvature of the trace within the window being low and high enough, respectively, and having a set decrease in the reporter signal (normalized to reporter expression). APC/C^Cdh1^ inactivation (defined as the start of APC/C^Cdh1^ reporter accumulation) was detected in a complementary way, identifying the first point where the slope increases to a threshold level and the reporter increases from a low level to a threshold-value of persistent increase. All threshold values were empirically determined and validated by eye on at least 200 traces. For both CRL4^Cdt2^ and APC/C^Cdh1^ reporters, the integrated intensity within the nucleus was quantified for trace analysis.

For identification of the start of S phase from PCNA foci, foci were segmented, and the total area of foci was quantified. The transition from G1 to S phase is characterized by an increase of low foci signal to high foci signal. For RT-QIBC analysis, the same algorithm was used for the APC/C^Cdh1^ reporter (as the increase in puncta area mirrors the rise in APC/C^Cdh1^ reporter levels). For confocal imaging, a dual threshold algorithm was used. A high foci signal threshold that robustly identified S phase cells was manually determined (50 pixels), and the first point at which the foci area increased above this threshold and was higher than the previous 4 frames was identified. Since the high foci signal threshold typically identified a point well after S phase entry, the final frame before this high identified point which was below a low threshold (3 pixels) was identified as the true S phase entry point.

##### RT-QIBC

After automated tracking and quantification of live- and fixed-cell imaging, each cell was associated with its corresponding annotated cell cycle reporter traces, as well as multidimensional fixed-cell measurements from QIBC. Based on this, the time elapsed from a point of interest (such as CRL4^Cdt2^ activation or mitosis) was used to arrange fixed-cell measurements based on time. Conversely, live-cell traces can be selected based on QIBC measurements (such as the expression of ND-CDT1). For analyses with high time resolution (e.g. Figures 1E-1G and S11), time offsets for each imaging site and well were accounted for based on the order of well acquisition.

##### Chromatin-bound ND-CDT1 localization analysis

We measured the Pearson correlation coefficient of chromatin-bound ND-CDT1 relative to chromatin-bound CDC45 (which marks CMG at sites of DNA synthesis) as a measure of colocalization. First, nuclei were segmented using Hoechst DNA stain to confine the analysis to signals within the nucleus. ND-CDT1 and CDC45 signals were masked by the nuclear mask, and then the Pearson correlation coefficient was calculated between these signals. To gauge the significance of these correlations, the ND-CDT1 and CDC45 were randomized by shifting the two channels 40 pixels (1.038 μm) relative to each other. Pearson correlation coefficients were calculated between shifted images (only analyzing pixels within the nucleus) in each of 4 shifting directions (up, down, left, and right), and the mean correlation coefficient of these 4 measurements was considered the randomized data for a given cell. To visualize the ND-CDT1 and CDC45 segmented signals in Figure 7D, each signal was processed with a top-hat filter with a 10-pixel radius and then a Gaussian filter with a 1-pixel radius. Signals were segmented by applying a uniform threshold to the filtered images.

#### Cell gating

To estimate thresholds in an automated manner for determining cells positive and negative for QIBC staining (for example, chromatin-bound PCNA positive cells for S phase cells) or 2N DNA content, cells known to be in either G1 or S phase based on livecell imaging were identified, and then the 99^th^ percentile (95^th^ for Figure S11) was chosen as the threshold.

For Dox-inducible cell lines, cells within populations expressed different amounts of protein in response to uniform Dox, with a small subset of cells not inducing protein to detectable levels. To correct for this, cells were selected for analysis based on either immunofluorescence staining of induced-protein at the end of the experiment (e.g. HA-tag, CDT1 or Geminin staining), or in the case of fluorescent protein-tagged protein induction (e.g. ND-CDT1-mCherry), cells were selected based on live-cell imaging of the induced protein. Thresholds were based on uninduced cells.

G1 and S phase cells were identified through a combination of live-cell reporter quantification and fixed-cell stains. In Figure 2A, late G1 to early S cells were defined by 2N DNA and intermediated Cyclin E/A-CDK activity (0.7 - 1.2). In Figure 5A, G1 cells had inactive CRL4^Cdt2^ (reporter not degraded) and 2N DNA. For Figures 6A–6D, G1 cells had active APC/C^Cdh1^ (APC/C^Cdh1^ reporter low) without chromatin-bound PCNA, while S phase cells were 2-3 h after APC/C^Cdh1^ inactivation and chromatin-bound PCNA positive. For Figure 7B, G1 cells had active APC/C^Cdh1^ without chromatin-bound PCNA, while S phase cells were fixed 2-3 h after APC/C^Cdh1^ inactivation and were chromatin-bound PCNA positive. For remaining figures, gating criteria are listed in Figure legends.

#### Quantification corrections

For experiments with chromatin-bound proteins measured after pre-extraction, there were rare sections of the samples that were incompletely extracted of soluble proteins. As a proxy for extraction efficiency, in experiments with APC/C^Cdh1^ or Cyclin E/A-CDK reporters (which are soluble), the residual fluorescent protein signal was imaged in addition to immunofluorescence. Cells that had high fluorescent protein signal for the reporters were considered incompletely extracted and removed from the analysis.

For the staining of replication factors in Figures 6 and S6, staining was performed in two rounds. In the first round, chromatin-bound replication proteins (CDC45, TIMELESS, POLE2, POLA2, POLD2, PCNA) were stained using Alexa Fluor 647 secondary antibodies simultaneously with EdU staining with AFDye 488. Fluorophores were then bleached for 1 h and re-stained for PCNA using an Alexa Fluor 647 secondary antibody to identify S phase cells. However, there was a low-intensity residual signal from the first round of staining, which was corrected by using an empirically determined residual signal scaling factor.

For Figures S4B and S4C, H2B-iRFP670 was expressed in a bicistronic vector together with the APC/C^Cdh1^ reporter (P2A sequence). As a result, the APC/C^Cdh1^ reporter signal could be normalized by the H2B-iRFP670 signal to control for differential expression of the construct between cells.

For pre-extraction experiments of replication factors (Figures 6 and S6), outliers resulting from incompletely extracted cells and imaging artifacts were removed by removing the top 1 percentile of data. In Figure S3B, the outer 1 percentile of CRL4C^dt2^ activation delays from APC/C^Cdh1^ inactivation was removed to account for misidentified cell cycle transitions.

When equal numbers of cells were plotted from different conditions, cells were randomly subsampled to equalize conditions without replacement.

#### Data normalization

For normalized stain quantification, a baseline was calculated from G1 levels of the stain and subtracted off of all values, followed by division by the group to be normalized. For EdU quantification shown on a linear scale (for dose-responses and chromatin-bound stain linear fits), the G1 background signal was subtracted off values for a true zero. For Figures 1F and 1G, measurements were normalized to the median G2 signal of each protein. For Figures 4E, S5F, and S5G, the EdU signal was background subtracted and divided by the Dox(-) EdU signal 1 – 1.2 h after S phase entry to standardize values between replicates. For Figure 5C, the Cyclin E/A-CDK activity at S phase start was subtracted off values to plot the change of Cyclin E/A-CDK activity from S phase start over time.

#### Statistical analysis

Details of statistical tests can be found in the figure legends. Comparisons were made with either paired *t*-tests (for tests between multiple independent replicate experiments) or two-sample *t*-tests for within-experiment comparisons of measurements with an α of.05.

For linear fits of chromatin-bound stains (Figure 6), a linear model with a fixed zero-intercept was fit using robust fitting with a bisquare weight function (tuning constant of 2). For fitting dose-response curves cells could be stratified based on their ND-CDT1 expression. Single-cell measurements of EdU and the single-cell expression of ND-CDT1 were fit to a Hill equation of the form

$$EdU([NDCd1])=EdU_{max}-\frac{EdU_{max}-EdU_{min}}{1+{\left(\frac{IC_{50}}{\left[NDCd1\right]}\right)}^{n}}$$

using nonlinear regression. *EdU_max_* is the EdU incorporation of cells without ND-CDT1, which was set using cells not expressing ND-CDT1. *EdU_min_* represents the minimum EdU incorporation, *IC*_50_is the 50% inhibitory concentration of ND-CDT1 concentration [*NDCdt1*], and *n* is the Hill coefficient. For Figures 3F and 5D, *EdU_min_*, *IC_50_* and *n* were all fit parameters, while for Figures 3G, 3H, S4F, and S4G, *EdU_min_* was set based on high levels of ND-CDT1 expression. Nonlinear regression was performed using the Levenberg-Marquardt algorithm (nlinfit() in MATLAB). Initialization parameters for *EdU_inhib_* were estimated from the 5^th^ percentile of EdU signal, while *EdU_inhib_* was initialized based on the median [*NDCdt* 1] in the cell population, and was *n* initialized as 1.

For Figure 3F, maximum EdU inhibition (*EdU_min_= EdU_max_*) was estimated from Hill equation fit curve to be 22.0-fold (siCtrl) and 23.0-fold (siGeminin). IC_50_ was 10.2 (siCtrl), and 7.7 (siGeminin). Hill coefficients were 4.2 (siCtrl) and 1.8 (siGeminin). For Figure 5D, maximum EdU inhibition of 25.7-fold (DMSO), 17.6-fold (ATRi), and 17.0-fold (WEE1i). IC_50_ was 5.49 (DMSO), 5.37 (ATRi), and 5.32 (WEE1i).

For bootstrapped estimators, samples were resampled at least 1000 times, and confidence intervals were calculated using the percentile method. For raincloud plots,^68^ which are combined violin and jitter plots, the data distribution was estimated using a kernel smoothing density estimate. The solid and dashed lines in the violin plot correspond to the median and inter-quartile range (IQR).

#### Visualization

All example cells were extracted from full-sized images through MATLAB scripts and selected based on RT-QIBC or time-lapse analysis. For Figure 7D, cells were selected for imaging based on being in early or late S phase. An example cell was chosen and visualized using ImageJ (v1.53, Fiji distribution)^62^ with the QuickFigures plugin.^69^

## Supplementary Material

Article & Supplementary Information

Figures S1-S7

Table S1

## Data Availability

Original microscopy images and uncropped gel images corresponding to images shown in the figures have been deposited to Mendeley Data and are publicly available as of the date of publication. Full raw imaging datasets generated in this study cannot be deposited in a public repository due to storage limitations but are available from the lead contact upon request. Processed data (single-cell quantifications) generated from image datasets that were used for analysis and generation of the figures in this study have been deposited at Dryad and are publicly available as of the date of publication. DOIs are listed in the key resources table. All original code, including custom MATLAB image-processing pipeline and scripts used to generate the figures from this study, have been deposited at Github (https://github.com/MeyerLab/image-analysis-ratnayeke-2022) and Zenodo, and are publicly available as of the date of publication. Zenodo DOI is listed in the key resources table. Any additional information required to reanalyze the data reported in this paper is available from the lead contact upon request.

## Highlights

- Licensing factor CDT1 is present with fired replication origins in early S phase
- Cdt1 inhibits CMG helicase after origins fire until CRL4^Cdt2^ fully degrades CDT1
- Inhibiting CMG helicase suppresses DNA synthesis and thus deters re-replication
- Mechanistic insight from parallel single-cell analysis and *in vitro* reconstitution

## In brief

Ratnayeke et al. reveal how cells duplicate DNA precisely once during a period in early S phase when CDT1 overlaps with fired replication origins. They find that, after licensing origins in G1, CDT1 suppresses DNA synthesis at fired origins until CDT1 is fully degraded to deter re-replication during the overlap.
