# Supplementary material for: CDT1 inhibits CMG helicase in early S phase to separate origin licensing from DNA synthesis
Source: Mol Cell. Author manuscript; Available in PMC 2023 Jun 15. (PMC7614657; doi:10.1016/j.molcel.2022.12.004)
Supplement: Article & Supplementary Information [file EMS176983-supplement-Article___Supplementary_Information.pdf]

Molecular Cell

Article

CDT1 inhibits CMG helicase in early S phase to separate origin licensing from DNA synthesis

Graphical abstract

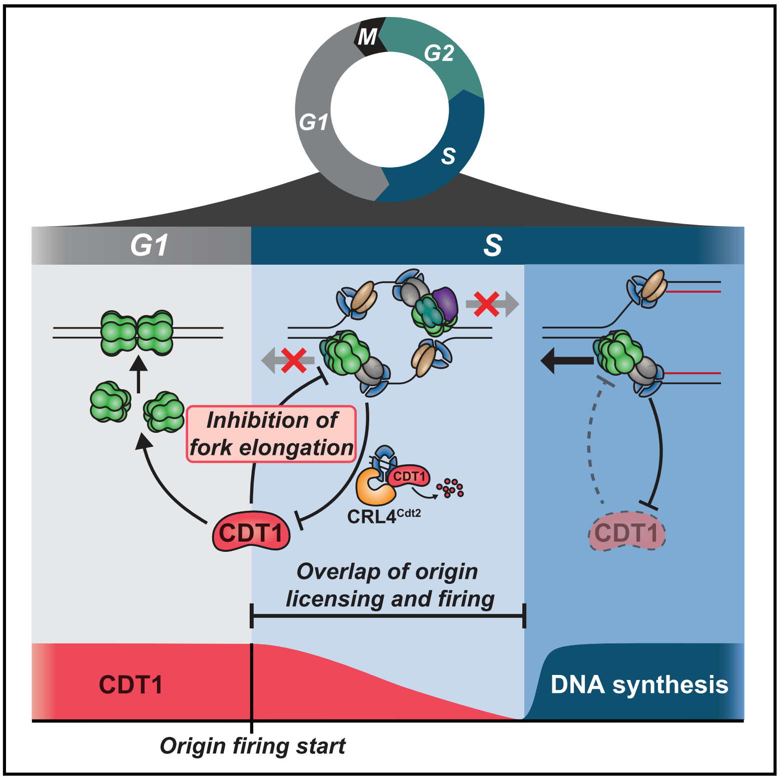

Highlights

- Licensing factor CDT1 is present with fired replication origins in early S phase
- Cdt1 inhibits CMG helicase after origins fire until CRL4^{Cdt2} fully degrades CDT1
- Inhibiting CMG helicase suppresses DNA synthesis and thus deters re-replication
- Mechanistic insight from parallel single-cell analysis and *in vitro* reconstitution

Authors

Nalin Ratnayeke, Yasemin Baris, Mingyu Chung, Joseph T.P. Yeeles, Tobias Meyer

Correspondence

tom4003@med.cornell.edu

In brief

Ratnayeke et al. reveal how cells duplicate DNA precisely once during a period in early S phase when CDT1 overlaps with fired replication origins. They find that, after licensing origins in G1, CDT1 suppresses DNA synthesis at fired origins until CDT1 is fully degraded to deter re-replication during the overlap.

Ratnayeke et al., 2023, Molecular Cell *83*, 26–42
January 5, 2023 © 2022 Published by Elsevier Inc.
https://doi.org/10.1016/j.molcel.2022.12.004

CellPress

Article

CDT1 inhibits CMG helicase in early S phase to separate origin licensing from DNA synthesis

Nalin Ratnayeke,[1,2] Yasemin Baris,[3] Mingyu Chung,[1] Joseph T.P. Yeeles,[3] and Tobias Meyer[1,2,4,*]
[1]Department of Chemical and Systems Biology, Stanford University School of Medicine, Stanford, CA 94305, USA
[2]Department of Cell and Developmental Biology, Weill Cornell Medical College, New York, NY 10065, USA
[3]Laboratory of Molecular Biology, Medical Research Council, Cambridge CB2 0QH, UK
[4]Lead contact
*Correspondence: tom4003@med.cornell.edu
https://doi.org/10.1016/j.molcel.2022.12.004

SUMMARY

Human cells license tens of thousands of origins of replication in G1 and then must stop all licensing before DNA synthesis in S phase to prevent re-replication and genome instability that ensue when an origin is licensed on replicated DNA. However, the E3 ubiquitin ligase CRL4^{Cdt2} only starts to degrade the licensing factor CDT1 after origin firing, raising the question of how cells prevent re-replication before CDT1 is fully degraded. Here, using quantitative microscopy and *in-vitro*-reconstituted human DNA replication, we show that CDT1 inhibits DNA synthesis during an overlap period when CDT1 is still present after origin firing. CDT1 inhibits DNA synthesis by suppressing CMG helicase at replication forks, and DNA synthesis commences once CDT1 is degraded. Thus, in contrast to the prevailing model that human cells prevent re-replication by strictly separating licensing from firing, licensing and firing overlap, and cells instead separate licensing from DNA synthesis.

INTRODUCTION

To duplicate their genome precisely once, eukaryotic cells are thought to strictly separate DNA replication into a period of replication origin licensing and a period of origin firing.[1–3] During licensing in G1 phase, cells demarcate future sites of DNA synthesis by loading inactive minichromosome maintenance (MCM)2–7 helicases onto origins of replication. At the start of S phase, cells begin origin firing, whereby replication factors are recruited to the inactive helicases to form active CDC45-MCM2–7-GINS (CMG) helicases and replication forks that duplicate DNA. Critically, it is thought that origin licensing must be strictly separated in time from origin firing to avoid re-replication, which occurs when newly synthesized DNA is re-licensed and replicated again within the same cell cycle.[1,3,4] Avoiding re-replication is crucial for maintaining genome stability, and failure to do so results in gene amplification, DNA damage, oncogenesis, and cell death.[1,5]

The G1/S transition is a particularly vulnerable period in the cell cycle when cells must simultaneously inactivate licensing and initiate origin firing. In humans and other vertebrates, avoiding re-replication is critically dependent on the repression of the essential licensing factor CDT1 from the start of S phase through anaphase.[5] Activation of CDT1 during this period is sufficient to trigger re-replication.[6–9] CDT1 activity can be repressed by CDT1 degradation mediated by cullin-RING E3 ubiquitin ligases CRL4^{Cdt2} and SCF^{Skp2} (also known as CRL1^{Skp2}), as well as by GMNN (geminin) binding to CDT1 and hyperphosphorylation of CDT1 by cyclin A-CDK1.[5,10] Both geminin and cyclin A are degraded during G1 by E3 ubiquitin ligase APC/C^{Cdh1} and only begin to accumulate after APC/C^{Cdh1} inactivation at the start of S phase,[11–13] while SCF^{Skp2}-mediated CDT1 degradation is thought to only begin in mid-S phase.[14,15] These findings suggest that degradation of CDT1 by CRL4^{Cdt2} alone is responsible for preventing re-replication in early S phase.

However, the exclusive role of CRL4^{Cdt2} in inactivating CDT1 at the start of S phase poses a conundrum: for CRL4^{Cdt2} to ubiquitinate and degrade CDT1 in S phase, CDT1 must first bind to the replication fork component proliferating cell nuclear antigen (PCNA),[16,17] and therefore, CDT1 degradation can only start after origins have already fired. This regulation would result in an overlap period in early S phase when cells fire origins and could still license DNA before CDT1 is fully degraded.[1,4,17] Since it is expected that fired origins immediately synthesize DNA, this overlap period would be susceptible to re-licensing and re-replication. Even a small overlap could cause re-replication, since human diploid cells contain approximately 6 Gb pairs of DNA and replicate DNA at thousands of sites simultaneously, each of which provides an opportunity for re-replication.

Here, using a single-cell-microscopy-based analysis of human cells, we show that there is an overlap period in early S phase that lasts approximately 30 min, during which active CDT1 is present together with fired origins. Strikingly, using single-cell microscopy and *in-vitro*-reconstituted human DNA synthesis, we

 © 2022 Published by Elsevier Inc.

show that in addition to licensing origins in G1, CDT1 has an unexpected second role of inhibiting CMG helicase at replication forks during this overlap. Thus, cells can fire origins while inhibiting DNA synthesis, and this inhibition is only relieved once CDT1 is fully degraded. In this way, cells restrict the amount of synthesized DNA produced in the presence of CDT1 to deter re-replication. Conceptually, our study suggests that instead of temporally separating licensing and firing of origins in early S phase, human cells safeguard genome integrity by using CDT1-mediated CMG helicase inhibition to separate licensing and DNA synthesis.

RESULTS

Active CDT1 is present together with fired origins in early S phase

To determine whether CDT1 protein is present together with fired origins of replication (Figure 1A), we monitored the degradation of a doxycycline (Dox)-inducible CDT1-mCherry fusion protein in live MCF10A cells (a non-transformed human epithelial cell line). We simultaneously imaged EYFP-tagged PCNA, which forms foci at sites of origin firing and DNA synthesis.[18,19] In line with previous studies,[14,20] CDT1-mCherry degradation at S-phase start was coupled to the formation of PCNA foci (Figures 1B and S1A–S1C). Mutant analysis of CDT1 degrons confirmed that CDT1-mCherry degradation at S-phase start is mediated by $CRL4^{Cdt2}$, while SCF^{Skp2} does not contribute until mid-S phase (Figures S1D–S1F). There is approximately 30 min between the start and completion of CDT1-mCherry degradation (Figures S1A and S1B), suggesting that there is an extended overlap period in early S phase when CDT1 is present together with fired origins.

To determine whether endogenous CDT1 similarly overlaps with fired origins in early S phase, we combined live-cell microscopy of S-phase entry reporters with fixed-cell immunofluorescence (IF) microscopy of endogenous CDT1. To precisely measure S-phase entry in live cells, we imaged a component of the FUCCI(CA) cell-cycle reporter system, human $CDT1^{(1-100)\Delta Cy}$, which is rapidly degraded by $CRL4^{Cdt2}$ in response to origin firing at S-phase start[15] (Figure 1C). We used this reporter in its original N-terminal mCherry-tagged orientation (referred to here as N-$CRL4^{Cdt2}$ reporter) and also created a C-terminal tagged reporter (C-$CRL4^{Cdt2}$ reporter), which is degraded with slightly faster kinetics and further facilitates precise measurement of S-phase entry (see STAR Methods for discussion of reporters and Figures 1C, S1G, and S1H). We use the term S-phase entry to refer to the start of origin firing, which is marked by the degradation of the $CRL4^{Cdt2}$ reporters (Figure 1C).

We live-imaged thousands of asynchronously proliferating cells and detected S-phase entry using automated time-lapse analysis of the C-$CRL4^{Cdt2}$ reporter. Cells were immediately fixed after live-cell imaging and stained for endogenous CDT1. Single-cell CDT1 levels were then measured by quantitative image-based cytometry (QIBC).[21] Finally, we computationally matched each cell from the fixed-cell QIBC analysis to its S-phase entry time from live-cell measurements.[22–25] This allowed us to analyze endogenous CDT1 levels as a function of time after S-phase entry (Figure 1D). We refer to this combined live and fixed-cell technique as retrospective time-lapse synchronized QIBC (RT-QIBC).

Based on RT-QIBC of endogenous CDT1 IF staining in asynchronously cycling cells, endogenous CDT1 takes approximately 30 min to degrade following the start of S phase (Figure 1E). Control experiments showed that the start of S phase, as measured by the C-$CRL4^{Cdt2}$ reporter, coincided with the appearance of chromatin-bound PCNA (Figure S1I), confirming that origins had fired. Thus, there is an overlap period after the start of S phase where endogenous CDT1 is present together with fired origins.

During this overlap period, CDT1 could be active or could, at least in principle, be inhibited through binding by geminin or hyperphosphorylation by cyclin A-CDK1. However, analysis of an APC/C^{Cdh1} reporter showed that APC/C^{Cdh1}, which degrades geminin and cyclin A, is only inactivated near the start of S phase and can in some cells be inactivated after the start of S phase (Figures S1G and S1H), in line with previous findings.[14,15] Accordingly, we observed low levels of geminin and cyclin A in the first 30 min of S phase using RT-QIBC analysis (Figures 1F, 1G, and S2A–S2D). This result is consistent with previous studies showing that geminin and cyclin A contribute to CDT1 inhibition only later in S and G2 after they accumulate to high enough levels.[8,10] Since cyclin E-CDK2 does not hyperphosphorylate CDT1,[10] and since cyclin A and geminin are low in early S phase, we conclude that the CDT1 present in early S phase is active.

Our analysis revealed that unsynchronized cells start S phase with high CDT1 and low geminin and cyclin A (Figures 1E–1G). In contrast, we found that cells synchronized in early S phase using aphidicolin or thymidine[26] start S phase in a dramatically different state with low CDT1 and high levels of geminin and cyclin A (Figures S2E and S2F). This is consistent with the mechanisms of aphidicolin and thymidine, which block DNA synthesis after origin firing but allow geminin and cyclin A accumulation, as well as Cdt1 degradation in response to chromatin-bound PCNA (Figure S2E and S2F). These findings argue that single-cell analysis of asynchronous cells is needed to study regulatory events in early S phase.

We conclude that early S phase is characterized by an approximately 30-min-long overlap period, during which replication origins have fired and CDT1 is still present and active. This presents a problem in the maintenance of genome integrity, as synthesized DNA at these fired origins would be susceptible to re-licensing by CDT1 and re-replication.

DNA synthesis is inhibited in the presence of CDT1

We next determined how much DNA is synthesized during the overlap period when origins have fired and CDT1 is still present. We measured the levels of CDT1 together with DNA synthesis, measured by the incorporation of 5-ethynyl-2′-deoxyuridine (EdU) into synthesized DNA in an 8 min period just before cell fixation. Strikingly, as cells transitioned from G1 to S phase, CDT1 and EdU staining were mutually exclusive (Figure 2A), arguing that while origins fire in the presence of CDT1, there is no detectable DNA synthesis occurring during the overlap period.

One possible explanation for the lack of EdU incorporation is that CDT1 itself suppresses DNA synthesis. To explore this possibility, we examined EdU incorporation by RT-QIBC

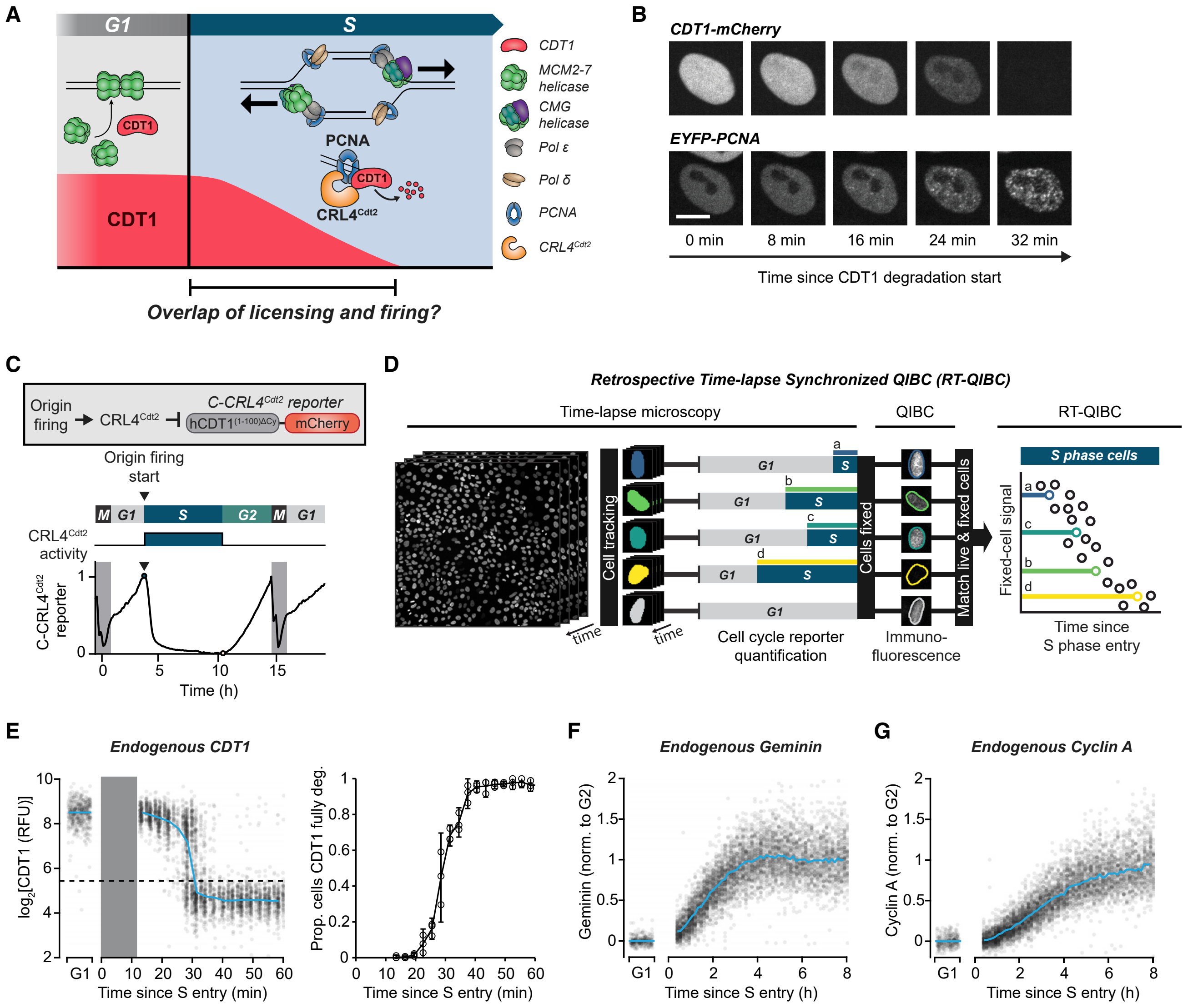

Figure 1. Active CDT1 is present together with fired origins in early S phase

(A) Predicted overlap between CDT1 and origin firing in early S phase.

(B) MCF10A cells expressing EYFP-PCNA and doxycycline-inducible CDT1-mCherry, induced 6 h before imaging. Representative of 54 cells. Scale bars, 10 μm. Quantification in Figures S1A–S1C and expression in Figure S1F.

(C) Top: live-cell reporter of CRL4^{Cdt2} activity. Bottom: example trace of C-CRL4^{Cdt2} reporter in a single MCF10A cell. Reporter is degraded at S entry.

(D) Diagram of retrospective time-lapse synchronized QIBC (RT-QIBC). Time-lapse microscopy of H2B-mTurquioise and QIBC of CDT1 immunofluorescence (IF).

(E–G) RT-QIBC aligned to S entry (C-CRL4^{Cdt2} reporter degradation). G1 cells are 1–2 h after anaphase. Solid blue curves are median value. Representative of 3 independent experiments.

(E) Left: CDT1 IF (n = 3,710 S cells, 500 G1 cells). Dashed line is threshold for fully degraded CDT1. Gray bar is period that is not observed due to the requirement of 12 min of reporter degradation to identify S entry. Right: quantification of left. Proportion of cells with fully degraded CDT1 over time within 3 min bins for 3 experiments (n ≥ 36 cells per point). Error bars are 95% confidence intervals.

(F) Geminin IF (n = 13,262 S cells, 300 G1 cells).

(G) Cyclin A IF (n = 13,262 S cells, 300 G1 cells).

See also Figures S1 and S2.

cells expressing an APC/C^{Cdh1} reporter together with high levels of Dox-inducible CDT1-mCherry that remained present in early S phase for a longer period (Figures 2B and S3A–S3C). Markedly, these cells also exhibited a period of inhibited EdU incorporation after APC/C^{Cdh1} inactivation, which closely corresponded to the prolonged time during which CDT1-mCherry was still being degraded (Figure 2B, shaded area). In line with this interpretation, we identified a prominent population of cells with chromatin-bound PCNA but low EdU incorporation, corresponding to cells that had fired origins but had not yet

CellPress

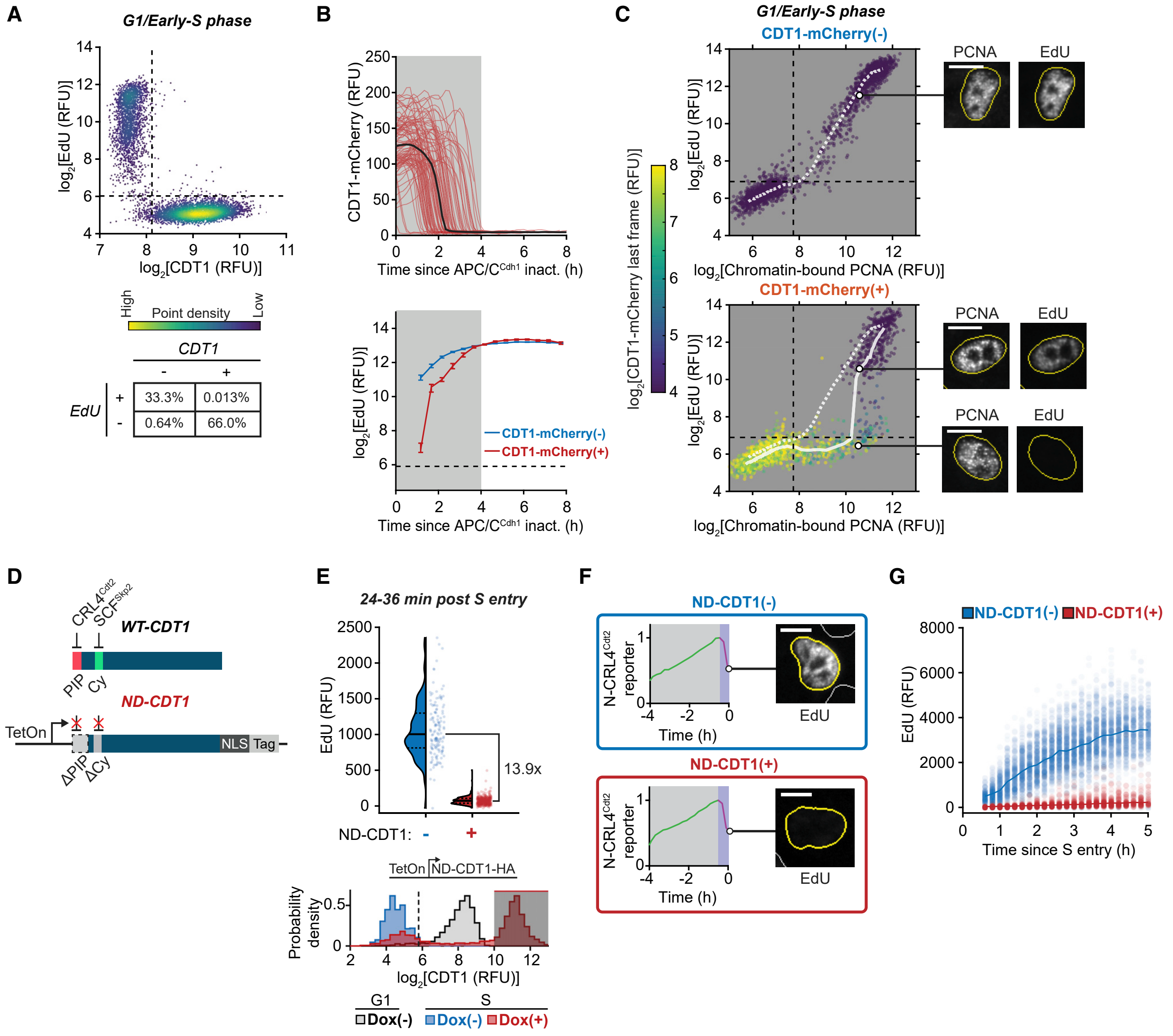

Figure 2. DNA synthesis is inhibited in the presence of CDT1

(A) CDT1 immunofluorescence (IF) and EdU in MCF10A cells in late G1 to early S (see STAR Methods). n = 7,486 cells, representative of 2 independent experiments. Percentage of cells in bottom table.

(B and C) RT-QIBC in mitogen-released MCF10A cells with APC/C^{Cdh1} reporter and doxycycline (Dox)-inducible CDT1-mCherry. CDT1-mCherry expression in Figure S3C.

(B) Top: live-cell CDT1-mCherry (100 traces). Black curve is median trace (n = 7,059 cells). Bottom: RT-QIBC of EdU in S cells (PCNA positive) aligned to APC/C^{Cdh1} inactivation. CDT1-mCherry(–): 18,367 cells, CDT1-mCherry(+): 6,970 cells. Error bars are mean and bootstrapped 95% confidence interval of cells within 30 min bins ($n \geq 93$ per bin).

(C) Chromatin-bound PCNA and EdU in 2N DNA cells (G1/early S), colored by live-imaged CDT1-mCherry. CDT1-mCherry(–): n = 3,000 cells, CDT1-mCherry(+): 2,000 cells. White lines are median EdU. Representative cells shown.

(D–F) RT-QIBC in MCF10A cells overexpressing Dox-inducible non-degradable CDT1 (ND-CDT1), induced with Dox, live-imaged for 6 h and aligned to S entry (N-CRL4^{Cdt2} reporter degradation). Cells born within 1 h of Dox addition were analyzed. Representative of 2 independent experiments.

(D) Diagram of ND-CDT1 construct.

(E) Top: cells 24–36 min after S entry. ND-CDT1(–): n = 141 cells, ND-CDT1(+): n = 400 cells. Bottom: CDT1 in G1 cells (1–2 h after mitosis) (gray, n = 2,191 cells) and in S cells (0.5–1 h after S entry) with either ND-CDT1 induced (red, n = 6,389 cells) or not induced (blue, n = 783 cells). Shaded area represents cells selected for ND-CDT1(+).

(F) Representative N-CRL4^{Cdt2} reporter trace (magenta area represents time following S entry) and corresponding EdU image.

(legend continued on next page)

fully degraded CDT1-mCherry (Figure 2C, lower-right quadrant).

Since CDT1-mCherry in the above experiments was still ultimately degraded in S phase, we more directly tested for a suppressive role of CDT1 by engineering a non-degradable mutant of CDT1 (ND-CDT1). Degradation of ND-CDT1 was prevented by both removing the PCNA-interacting protein (PIP) degron, which is required for PCNA binding and $CRL4^{Cdt2}$-mediated degradation, and mutating the Cy motif, which is required for degradation by SCF^{Skp2} (Figures 2D, S3D, and S3E).[15,16,20] Markedly, similar to full-length CDT1, ND-CDT1 inhibited EdU incorporation (Figures 2E and 2F). Critically, inhibition of DNA synthesis did not prevent the firing of origins since the $CRL4^{Cdt2}$ reporter was degraded similarly to control cells (Figures S3F and S3G). Furthermore, continued expression of ND-CDT1 persistently inhibited EdU incorporation and prevented progression through S phase as measured by DNA content (Figures 2G, S3H, and S3I). To ensure that ND-CDT1 did not interfere with G1 origin licensing, we measured chromatin-bound MCM2 to quantify origin licensing[27,28] and found no change (Figures S3J and S3K). ND-CDT1 also inhibited DNA synthesis in U2OS and HeLa cells, arguing that this inhibition is not cell-type specific and occurs in both non-transformed and transformed cells (Figures S3L and S3M).

As an additional control, we confirmed that endogenous CDT1, not just overexpressed CDT1, can inhibit DNA synthesis when it fails to be degraded in S phase. To prevent the degradation of CDT1 in S phase, we acutely treated cells with MLN-4924, which blocks the activity of cullin-RING E3 ubiquitin ligases, including $CRL4^{Cdt2}$ (Figures S4A and S4B).[29] Cells treated with MLN-4924 had suppressed EdU incorporation following S-phase entry (Figure 3A, siCtrl conditions). Similar to overexpressed CDT1-mCherry, MLN-4924 increased the population of cells with chromatin-bound PCNA and low EdU incorporation (Figures 3C and 3D, siCtrl conditions). Knockdown of CDT1 partially rescued EdU incorporation, while knockdown of CDKN1A (p21) or CDKN1B (p27), other proteins stabilized by MLN-4924,[30] did not (Figures S4B and S4C). We conclude that both overexpressed and endogenous CDT1 can suppress DNA synthesis during S phase. These findings provide a potential explanation of how cells avoid re-replication during the overlap period when CDT1 is present together with fired origins in early S phase, as the amount of synthesized DNA, the substrate of re-replication, at these fired origins would be reduced until CDT1 is fully degraded (Figure 3E).

Geminin counteracts the inhibition of DNA synthesis by CDT1

When we prevented CDT1 degradation using MLN-4924, cells still started to increase EdU incorporation in S phase after a delay (Figure 3A). While geminin is initially very low in early S phase, it gradually accumulates throughout S phase (Figure 1F). We considered whether geminin binding to CDT1 could ultimately abrogate the inhibitory action of CDT1 on DNA synthesis. Consistent with this hypothesis, geminin knockdown prolonged the suppression of EdU incorporation in MLN-4924-treated cells (Figures 3A–3D, S4B, and S4C).

To more directly determine whether geminin antagonizes inhibition of DNA synthesis by CDT1, we measured the suppression of DNA synthesis at different levels of ND-CDT1 in the presence or absence of geminin. Cell populations with inducible ND-CDT1 showed variable expression between cells, and after computationally stratifying cells by their ND-CDT1 expression, we found that EdU incorporation was inhibited in a dose-dependent manner (Figure 3F). When geminin was allowed to accumulate, only cells with moderate-to-high ND-CDT1 could suppress DNA synthesis 2–3 h post S-phase entry. However, when we knocked down geminin, DNA synthesis stayed suppressed at much lower levels of ND-CDT1 (Figures 3F and S4D). By analyzing dose responses at a range of geminin levels (Figure S4E), we observed that DNA synthesis was increasingly inhibited by ND-CDT1 as geminin decreased, with the IC_{50} for ND-CDT1 decreasing linearly with geminin levels (Figures 3G, 3H, S4F, and S4G).

We conclude that geminin not only has a role in inhibiting CDT1 licensing[5] but also prevents CDT1 from inhibiting DNA synthesis. In this way, DNA synthesis can proceed either after CDT1 is degraded or after geminin levels have sufficiently increased to inhibit CDT1. Nevertheless, during the overlap period of CDT1 and fired origins in early S phase, geminin is low and cannot inhibit CDT1, arguing that suppression of DNA synthesis by CDT1 alone deters re-replication during the overlap period.

CDT1 suppresses DNA synthesis during the overlap period of licensing and firing

If endogenous CDT1 is indeed responsible for inhibiting DNA synthesis during the first 30 min of S phase, prematurely inactivating CDT1 should accelerate the start of DNA synthesis. Since CDT1 is required for origin licensing, we could not use long-term CDT1 knockdown. Instead, we made use of the licensing kinetics in MCF10A cells, which complete the majority of origin licensing shortly after anaphase and then further boost licensing during G1 (Figures S5A and S5B). In this way, acutely inactivating CDT1 during G1 would reduce but not prevent origin licensing, which cells can tolerate.[31]

We generated a cell line with Dox-inducible geminin to prematurely inactivate CDT1 during G1 (Figures 4A and S5C). To prevent geminin degradation in G1 by APC/C^{Cdh1}, we mutated the geminin D-box motif,[13,32] and the resulting cell line induced $Geminin^{\Delta Dbox}$ to levels higher than normal G2 geminin levels in ~50% of G1 cells (Figure 4B).

In initial control experiments, where we induced $Geminin^{\Delta Dbox}$ in asynchronous cells, cells that received Dox well before mitosis and thus expressed $Geminin^{\Delta Dbox}$ by the time they reached

(G) RT-QIBC in mitogen-released MCF10A cells. Cells were treated with control siRNA (same experiment as Figure 3F) and induced with Dox. ND-CDT1(+) cells selected based on gating in Figure S3H. ND-CDT1(−): n = 5,500 cells, ND-CDT1(+): n = 2,000 cells. Line is median value at each time point. Representative of 3 independent experiments.
Dashed lines are negative staining. Dashed and solid lines in violin plots are IQR and median. Scale bars, 10 μm.
See also Figure S3.

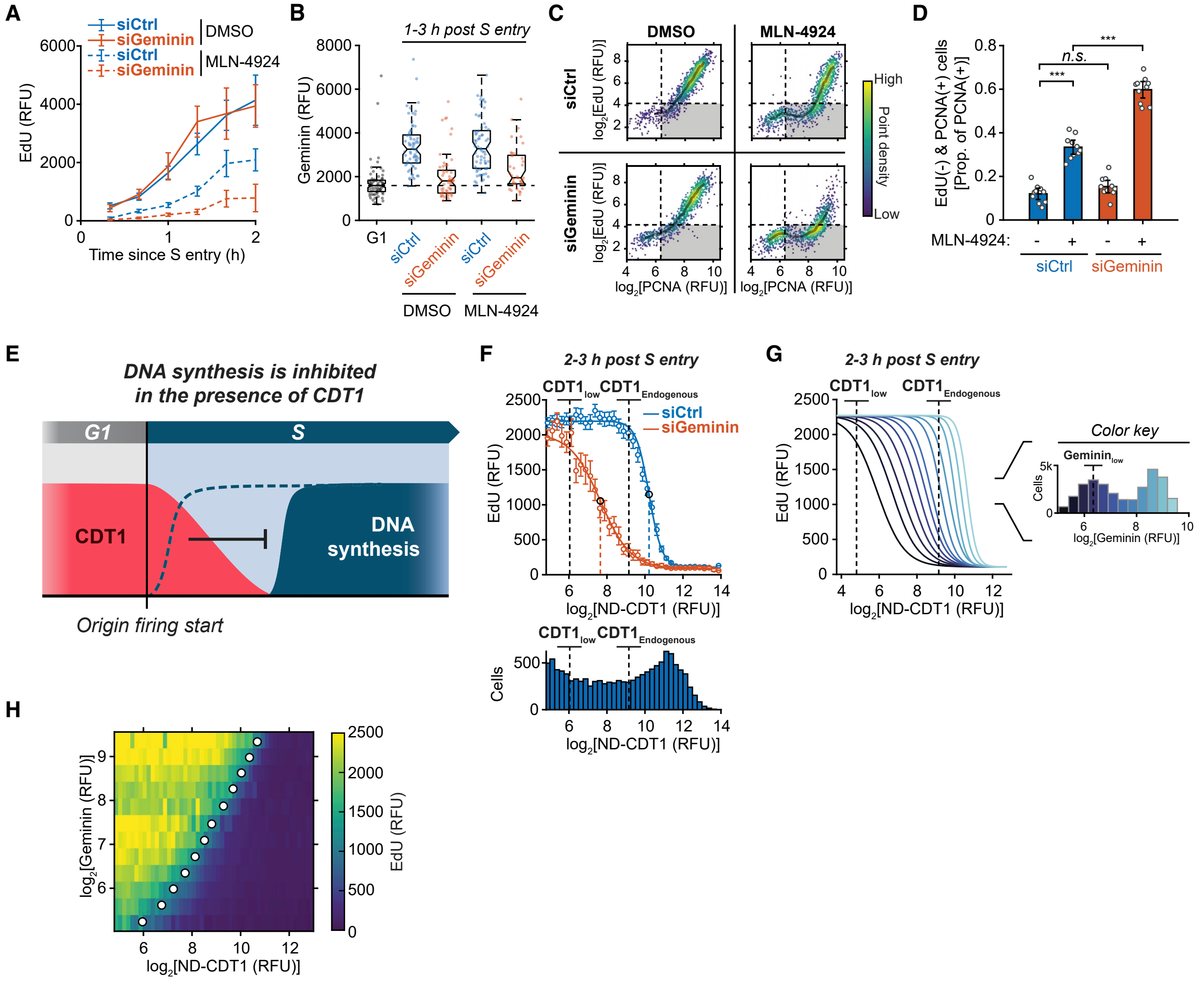

Figure 3. Endogenous CDT1 can inhibit DNA synthesis and is counteracted by geminin

(A and B) RT-QIBC aligned to S entry (PCNA foci) in MCF10A cells treated with 2 μM MLN-4924 for 3.5 h during live-cell imaging. Representative of 2 independent experiments. (A) EdU. Error bars are mean ± 2 × SEM for each time point ($n \geq 8$ cells for all time points, $n \geq 613$ cells per condition). (B) Geminin immunofluorescence 1–3 h after S entry. Box is IQR and median, and whiskers are 1.5 × IQR. G1 cells are DMSO-treated G1 cells (basal geminin), and dashed line is median of G1 cells. $n \geq 37$ cells per condition.

(C and D) RT-QIBC in mitogen-released MCF10A cells. MLN-4924 added 4 h before fixation. Cells 2–3 h after APC/C^{Cdh1} inactivation with cyclin E/A-CDK activity ≥ 0.8.

(C) Dashed lines are negative thresholds, and shaded curves are median in bins of PCNA levels ($n \geq$ 1,369 cells pooled from 10 replicate wells).

(D) Proportion of PCNA(+) cells that are EdU(−) in each of 10 replicate wells. Error bars are mean ± 2 × SEM. Two-sample t test p values: siCtrl DMSO vs. siCtrl MLN-4924 (7.8×10^{-6}), siCtrl DMSO vs. siGeminin DMSO (0.38), siCtrl MLN-4924 vs. siGeminin MLN-4924 (2.3×10^{-5}).

(E) DNA synthesis is inhibited in the presence of CDT1 after origin firing.

(F–H) RT-QIBC in mitogen-released MCF10A cells 2–3 h after S entry. Representative of 3 independent experiments.

(F) Top: dose-response of EdU to ND-CDT1 expression. Error bars are mean ± 2 × SEM for bins of ND-CDT1 expression (bins $\geq$ 75 cells, 12,039 cells total siCtrl, 4,573 cells total siGeminin). Curves are fit Hill equations. Fit parameters in STAR Methods. Bottom: corresponding cell counts for bins. Dashed lines are endogenous CDT1 levels ($CDT1_{Endogenous}$) and degraded CDT1 ($CDT1_{low}$), calculated from Figure S3H, and fit IC_{50}.

(G) Left: fit ND-CDT1 dose-response curves at 12 levels of geminin expression. Right: color code for geminin expression. $Geminin_{Low}$ is negative threshold. $n \geq 660$ cells per fit.

(H) Heatmap of median EdU (color) at given geminin and ND-CDT1 levels. Points represent IC_{50} at each geminin level. (n = 29,350 cells.)

See also Figure S4.

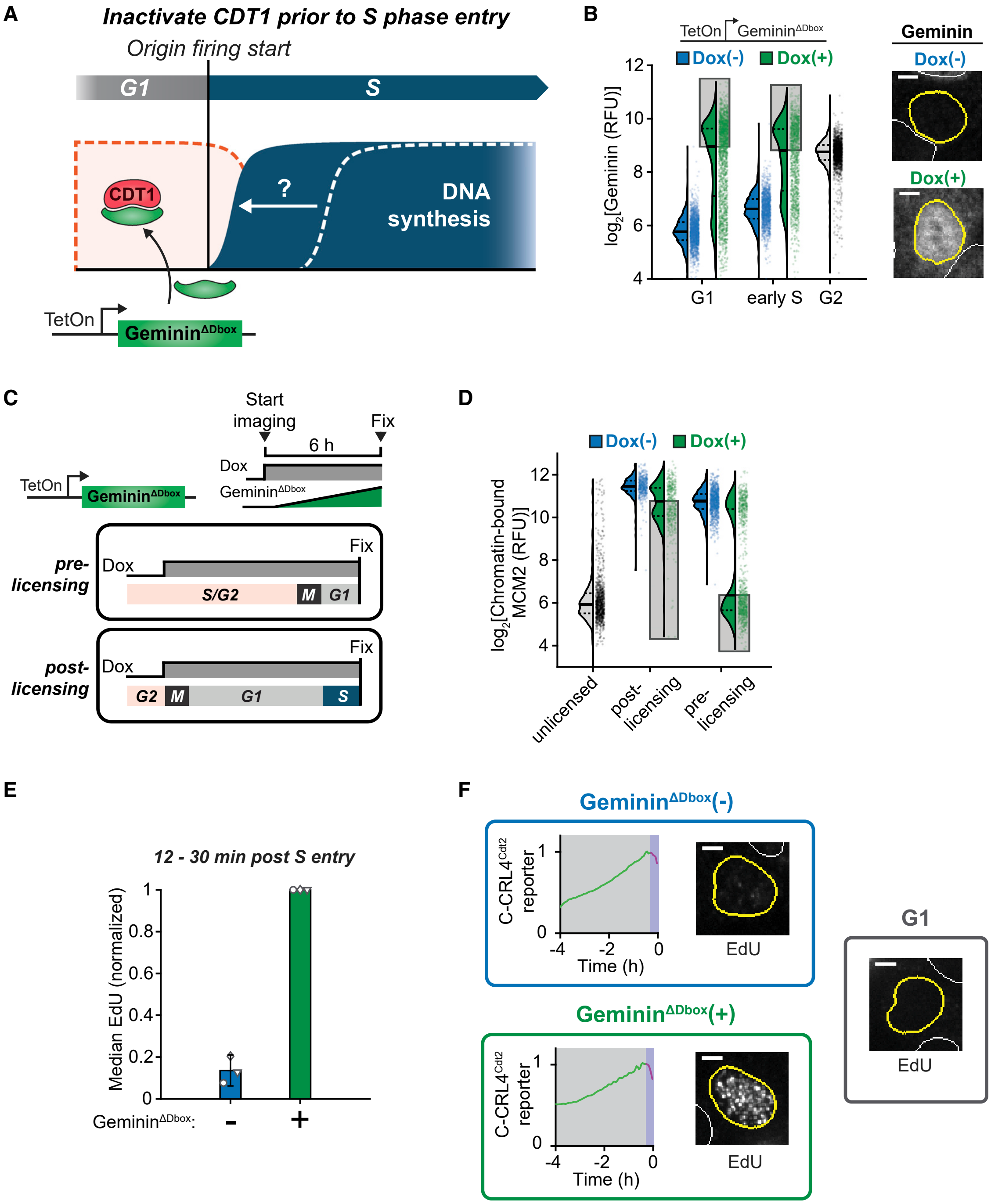

Figure 4. CDT1 suppresses DNA synthesis during the overlap period of licensing and firing

(A) Prematurely inactivating CDT1 with doxycycline (Dox)-inducible Geminin$^{\Delta Dbox}$ in G1 to accelerate the start of DNA synthesis.

(B–D) RT-QIBC in MCF10A cells with Geminin$^{\Delta Dbox}$ induced during live-cell imaging of C-CRL4^{Cdt2} reporter. Dashed and solid lines in violin plots are IQR and median. Representative of 2 (D) or 3 (B) independent experiments. Scale bars, 5 μm.

(B) Geminin immunofluorescence (detects geminin and Geminin$^{\Delta Dbox}$). Left: G1 (1–2 h post anaphase), S (≤0.5 h in S) and G2 (4N DNA, EdU(−), no Dox). Shaded area is upper 50% of cells, which induce Geminin$^{\Delta Dbox}$ higher than median G2. n ≥ 1,316 cells per condition. Right: example cells 1 h in G1.

(C) Cells were identified in two groups. Pre-licensing: Dox added ≥5 h before mitosis, and fixed ≤1 h in G1. Post-licensing: Dox was added ≤1 h before mitosis and fixed ≤1 h in S.

(D) Pre-extracted cells. MCM2 from unlicensed cells was estimated from G2 MCM2 signal (4N DNA and PCNA negative). Shaded area is lower 50% of cells, corresponding to cells in shaded bar in Figure 4B. n ≥ 434 per condition.

(legend continued on next page)

anaphase had completely inhibited origin licensing, indicating that Geminin$^{\Delta Dbox}$ had fully inhibited CDT1 (“pre-licensing,” Figures 4C and 4D). In contrast, cells that divided shortly after Dox addition, thus going through early G1 without Geminin$^{\Delta Dbox}$, only exhibited the expected moderate reduction in origin licensing resulting from inhibited licensing late in G1 (“post-licensing,” Figures 4C and 4D). We could therefore examine the latter group of cells to determine whether premature CDT1 inactivation accelerates DNA synthesis at S-phase start. Markedly, in the first 30 min of S phase, cells with CDT1 neutralized by Geminin$^{\Delta Dbox}$ exhibited approximately 5- to 10-fold higher EdU incorporation than control cells (Figures 4E, 4F, and S5D–S5G). We thus conclude that CDT1 is present in early S phase and suppresses DNA synthesis, providing a protective mechanism against re-replication during the overlap period where CDT1 is present together with fired origins.

CDT1 inhibits DNA synthesis independently of the intra-S-phase checkpoint and re-replication

Next, we sought to determine the mechanism by which CDT1 inhibits DNA synthesis. The intra-S-phase checkpoint, which limits the rate of DNA synthesis, can be activated in response to re-replication and DNA damage caused by CDT1 dysregulation.[9,33–35] Alternatively, the addition of high levels of CDT1 to replicating *Xenopus* egg extracts not only triggers checkpoint activation but also directly inhibits replication fork elongation,[36–38] suggesting other plausible mechanisms. Of note, CDT1-mediated inhibition of DNA synthesis in *Xenopus* egg extract was interpreted to be a safety response to aberrant CDT1 expression during S phase, rather than a protective mechanism routinely utilized during an overlap period in early S phase.

We first focused on whether the intra-S-phase checkpoint mediates the inhibition of DNA synthesis by CDT1. We overexpressed ND-CDT1 in cells with geminin knocked down to maximize the possibility of producing DNA damage and observed no increases in γH2AX, phospho-Chk1(S317) and phospho-Chk2(T68), markers of DNA damage and the intra-S-phase checkpoint (Figure 5A). As an independent measure of checkpoint activation, we turned to a live-cell reporter of the activity of cyclin E/A complexed with CDK2/1 (cyclin E/A-CDK) (Figure S5H).[39,40] Cyclin E/A-CDK activity increases throughout the cell cycle starting in G1. Cyclin A knockdown confirmed that cyclin A activity does not contribute to the G1 increase in reporter activity (Figures S5I and S5J), consistent with the low cyclin A IF staining during G1- and S-phase start (Figure 1G). Since cyclin E/A-CDK activity is partially inhibited by the intra-S-phase checkpoint, it can be used as a proxy for checkpoint activation.[41] Accordingly, hydroxyurea (HU) reduced cyclin E/A-CDK activity in S phase, while inhibitors of checkpoint mediators ATR or WEE1 increased cyclin E/A-CDK activity (Figure 5B). However, ND-CDT1 expression did not decrease cyclin E/A-CDK activity (Figure 5C). Furthermore, ND-CDT1 suppressed EdU incorporation with the same IC_{50} in the presence of ATR and WEE1 inhibitors (Figure 5D). Together, these results show that the intra-S-phase checkpoint does not mediate the suppression of DNA synthesis by CDT1.

It has also been suggested that re-replication can inhibit DNA synthesis independently of the intra-S-phase checkpoint.[33,42] If re-replication is necessary for the suppression of DNA synthesis by CDT1, blocking licensing activity in S phase, which is necessary for re-replication, should rescue DNA synthesis. To inhibit licensing, we used a previously developed RPE-1 $TP53^{-/-}$ cell line with mAID and SMASh-tag inducible degrons knocked into both copies of *CDC6* (referred to here as $CDC6^{d/d}$), another essential licensing factor (Figure 5E).[43] In this cell line, CDC6 can be rapidly degraded to very low levels within 4 h, and control experiments confirmed that degrading CDC6 inhibited origin licensing (Figure S5K).

We introduced Dox-inducible constructs for ND-CDT1-mCherry and control NLS-mCherry into the $CDC6^{d/d}$ cell line and synchronized cells in late G1 by releasing cells from G0 into a mimosine arrest, which blocks cells after origin licensing (Figure S5L).[44] In the final 4 h before releasing cells from mimosine arrest into S phase, we degraded CDC6 to prevent potential further licensing during S phase (Figures 5F and 5G). ND-CDT1-mCherry inhibited EdU incorporation following mimosine release, regardless of CDC6 degradation, arguing that re-replication is not necessary for CDT1 to inhibit DNA synthesis (Figures 5H and S5M). Furthermore, this cell line does not have functional TP53 (p53), which has also been implicated in the DNA damage response to re-replication.[9] We conclude that re-replication, p53, and intra-S-phase checkpoint activation are not required for CDT1-mediated inhibition of DNA synthesis in early S phase, arguing that CDT1 directly suppresses DNA synthesis.

CDT1 inhibits replication fork elongation while permitting origin firing

We next determined whether CDT1 suppresses DNA synthesis by inhibiting origin firing, or by inhibiting replication fork elongation after origin firing. To quantify origin firing and recruitment of replication factors to the replication fork in the presence of ND-CDT1, we measured chromatin-bound CDC45, TIMELESS, DNA polymerases epsilon, alpha, and delta (Pol ε, α, and δ), and PCNA (Figures 6A and S6A). Replication factors that are part of or bind to the CMG helicase (CDC45, TIMELESS, Pol ε, and Pol α) did not have impaired chromatin association in the presence of ND-CDT1, while Pol δ, which synthesizes lagging

(E and F) RT-QIBC in post-licensing Dox addition MCF10A cells. EdU added 30 min before fixation. Cells that were in S phase for 12–30 min and had not fully degraded CDT1 were analyzed. Geminin$^{\Delta Dbox}$(+) cells selected based on Geminin stain.

(E) For each of 3 independent experiments, the median of cells was taken (n ≥ 31 cells per replicate per condition; Geminin$^{\Delta Dbox}$(−): 120 cells total; Geminin$^{\Delta Dbox}$(+): 213 cells total) and normalized to Geminin$^{\Delta Dbox}$(+) condition. Error bars are mean ± 2 × SEM (Geminin$^{\Delta Dbox}$(−) cells are 13.6% ± 7.4% of Geminin$^{\Delta Dbox}$(+) cells). See Figure S5G for estimated absolute quantification.

(F) Representative EdU images and matching C-CRL4^{Cdt2} traces (magenta trace represents time following S entry). Geminin$^{\Delta Dbox}$(−) and Geminin$^{\Delta Dbox}$(+) cells 17.2 and 16.9 min in S phase. Scale bars, 5 μm.

See also Figure S5.

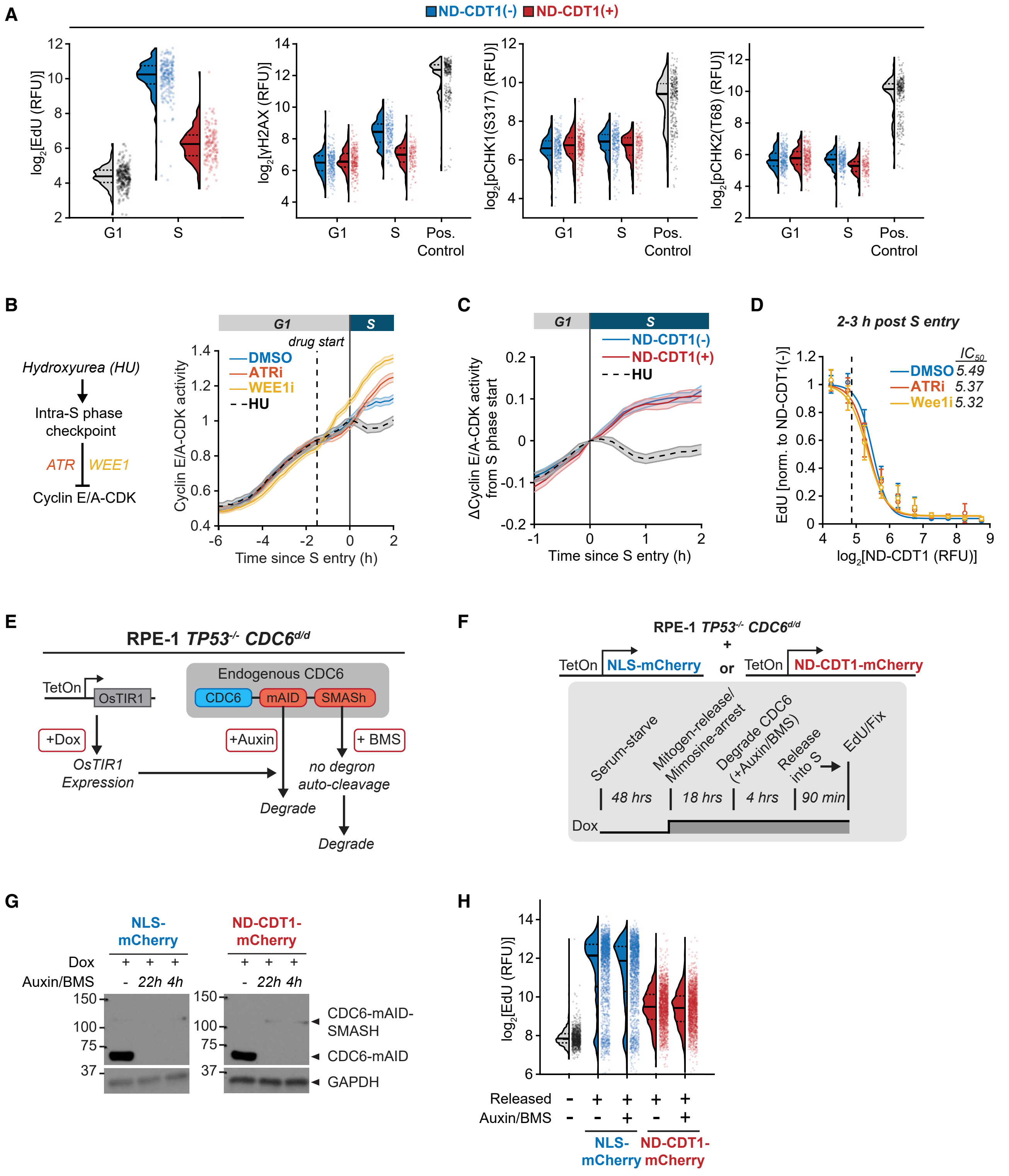

Figure 5. CDT1 inhibits DNA synthesis independently of the global intra-S-phase checkpoint and re-replication

(A) RT-QIBC in siGeminin-treated, mitogen-released MCF10A cells. S cells were 1–2 h into S phase. Positive controls were cells treated with 1 μM MK-1775 (WEE1i) for 4 h in S cells, which caused DNA damage. n ≥ 146 cells per condition.

(B) Mitogen-released MCF10A cells. 2 μM AZ-20 (ATRi), 1 μM WEE1i, or 2 mM hydroxyurea (HU) were added to cells 14 h after release. Cells that received drug 1–2 h before S entry were analyzed (dashed line is 1.5 h). Shaded area is mean ± 2 × SEM. n = 167 (DMSO), 145 (ATRi), 239 (WEE1i), and 119 (HU) cells.

(legend continued on next page)

strands, and PCNA were present but approximately 50% reduced (Figures 6A–6C and S6A–S6C).[45] These findings are consistent with our observation that CRL4^{Cdt2}, which depends on chromatin-bound PCNA, is still activated in the presence of ND-CDT1 (Figures S3F and S3G).

To determine whether reduced chromatin-bound Pol δ and PCNA are responsible for inhibited DNA synthesis, we simultaneously measured chromatin-bound replication factors and EdU incorporation. In control conditions there was a linear relationship between each chromatin-bound protein (CDC45, TIMELESS, Pol ε, Pol α, Pol δ, and PCNA) and EdU incorporation, indicating that each chromatin-bound protein signal is proportional to the number of active replication forks (Figures 6D and S6D). However, EdU incorporation was greatly reduced at matching levels of chromatin-bound protein in the presence of ND-CDT1 (Figures 6D, S6D, and S6E). This was the case even for Pol δ and PCNA, where EdU incorporation was much lower than expected given a 2-fold reduction in chromatin-bound levels. These results are consistent with CDT1 inhibiting replication fork elongation at fired origins.

Since PCNA and Pol δ are found at lagging-strand Okazaki fragments,[45,46] we hypothesized that the approximately 2-fold reduction in chromatin-bound PCNA and Pol δ in the presence of ND-CDT1 was a consequence of reduced fork elongation. Accordingly, chromatin-bound PCNA was lowered by HU, which blocks fork elongation, but was not further lowered in cells also expressing ND-CDT1 (Figure 6E). Such a reduction of PCNA at stalled forks has previously been reported.[47,48] In aggregate, our results indicate that CDT1 does not act by inhibiting origin firing but rather inhibits replication fork elongation.

To confirm that CDT1 can inhibit replication fork elongation, we used a recently developed *in vitro* assay for reconstituting CMG-dependent DNA synthesis using purified human proteins.[49] In this assay, replisome components (including CMG helicase) are loaded onto forked DNA templates and can perform leading-strand DNA synthesis alone or leading- and lagging-strand DNA synthesis (Figure 6F). Importantly, this assay is independent of origin firing. In this system, addition of CDT1 strongly inhibited DNA synthesis in a dose-dependent manner (Figures 6G and S6F). CDT1 inhibited both leading- and lagging-strand DNA synthesis (Figures 6G and S6G) and inhibited DNA synthesis not only when added to reactions from the start but also when added to already elongating forks (Figure 6H). Preincubation of CDT1 together with geminin neutralized its inhibitory effect (Figures 6G and S6G). Finally, CDT1 inhibited DNA synthesis in a replisome composed only of CMG, Pol ε, and replication protein A (RPA) (Figure S6H). These *in vitro* reconstitution experiments demonstrate that CDT1 inhibits replication fork elongation and DNA synthesis.

CDT1 inhibits CMG helicase through its MCM-binding domains

We next focused on how CDT1 suppresses replication fork elongation in early S phase. Replication fork elongation could be suppressed by inhibition of the replicative DNA polymerases or by inhibition of CMG helicase, which is responsible for unwinding double-stranded DNA. CDT1 contains a high-affinity interaction with PCNA through its PIP degron, and it has been suggested that PIP-degron-containing proteins can interfere with the binding of polymerases to PCNA, thereby inhibiting DNA synthesis.[50,51] However, ND-CDT1 has its PIP degron removed (Figure 2D), and CDT1 inhibits replication reactions *in vitro* independently of PCNA (Figure S6H), arguing against CDT1 inhibiting polymerases.

To determine whether CDT1 inhibits polymerases or CMG helicase, we examined the production of single-stranded DNA (ssDNA) at the replication fork. If CDT1 inhibited polymerases, ssDNA would accumulate as CMG helicase unwound DNA that the polymerases could not fill (Figure 7A).[21,36,52] As a measure of ssDNA, we examined the amounts of chromatin-bound ssDNA-binding protein RPA. In control cells without ND-CDT1 expression, there was measurable chromatin-bound RPA in S phase due to the normal production of ssDNA at replication forks (Figure 7B). Markedly, S-phase cells expressing ND-CDT1 had diminished RPA binding compared with control cells despite having similar amounts of origin firing, as measured by chromatin-bound CDC45. ND-CDT1 also prevented the increase in chromatin-bound RPA in response to HU and ATR inhibitor cotreatment (Figure 7B), which generates a large increase in ssDNA.[21] These results suggest that CMG helicase-mediated unwinding of DNA is suppressed in the presence of CDT1. These findings are supported by findings in *Xenopus* egg extract, where the addition of CDT1 also reduced chromatin-bound RPA.[36] Furthermore, CDT1 did not inhibit DNA synthesis in a Pol ε primer extension assay, which uses an ssDNA template and does not contain or rely on CMG helicase (Figure 7C).[49] In aggregate, our data are consistent with CDT1 inhibiting CMG helicase, rather than polymerase in early S phase.

As part of its role in origin licensing, CDT1 directly binds to soluble MCM helicases through two MCM-binding regions, which results in a conformational change in the MCM helicases that allows their loading onto origins.[5,20,53] This interaction is blocked

(C) Mitogen-released MCF10A cells. 2 mM HU added to HU condition 1–2 h before S entry. Shaded area is mean ± 2 × SEM. n $\geq$ 336 cells per condition.

(D) RT-QIBC of mitogen-released, siGeminin-treated MCF10A cells 2–3 h in S phase. 2 μM ATRi or 1 μM WEE1i were added 4 h before fixation. Error bars are mean ± 2 × SEM for bins of ND-CDT1 ($\geq$12 cells per bin, $\geq$770 cells per condition). Dashed line is threshold for ND-CDT1(−). Lines are fit Hill equations (fit parameters in STAR Methods).

(E) RPE-1 *TP53*$^{-/-}$ *CDC6*$^{d/d}$ cells degrade endogenous CDC6 with addition of Auxin and BMS-650032 (BMS).

(F–H) Dox-inducible ND-CDT1-mCherry or NLS-mCherry in RPE-1 *TP53*$^{-/-}$ *CDC6*$^{d/d}$ cells. Cells were mitogen-released in the presence of mimosine and doxycycline (Dox) for 18 h. CDC6 was degraded for 4 h, and then cells were released from mimosine arrest for 1.5 h, pulsed with EdU, and fixed.

(G) Western blot of CDC6 levels after acute 4 h or long-term 22 h degradation. Upper band is CDC6 with uncleaved SMASh-tag.

(H) QIBC of EdU in cells with inactive APC/C^{Cdh1}. NLS-mCherry/ND-CDT1-mCherry-positive cells were chosen based on gates Figure S5M. n = 1,120 cells (unreleased), 2,000 cells (other conditions).

Dashed and solid lines in violin plots are IQR and median. Data representative of 2 independent experiments.

See also Figure S5.

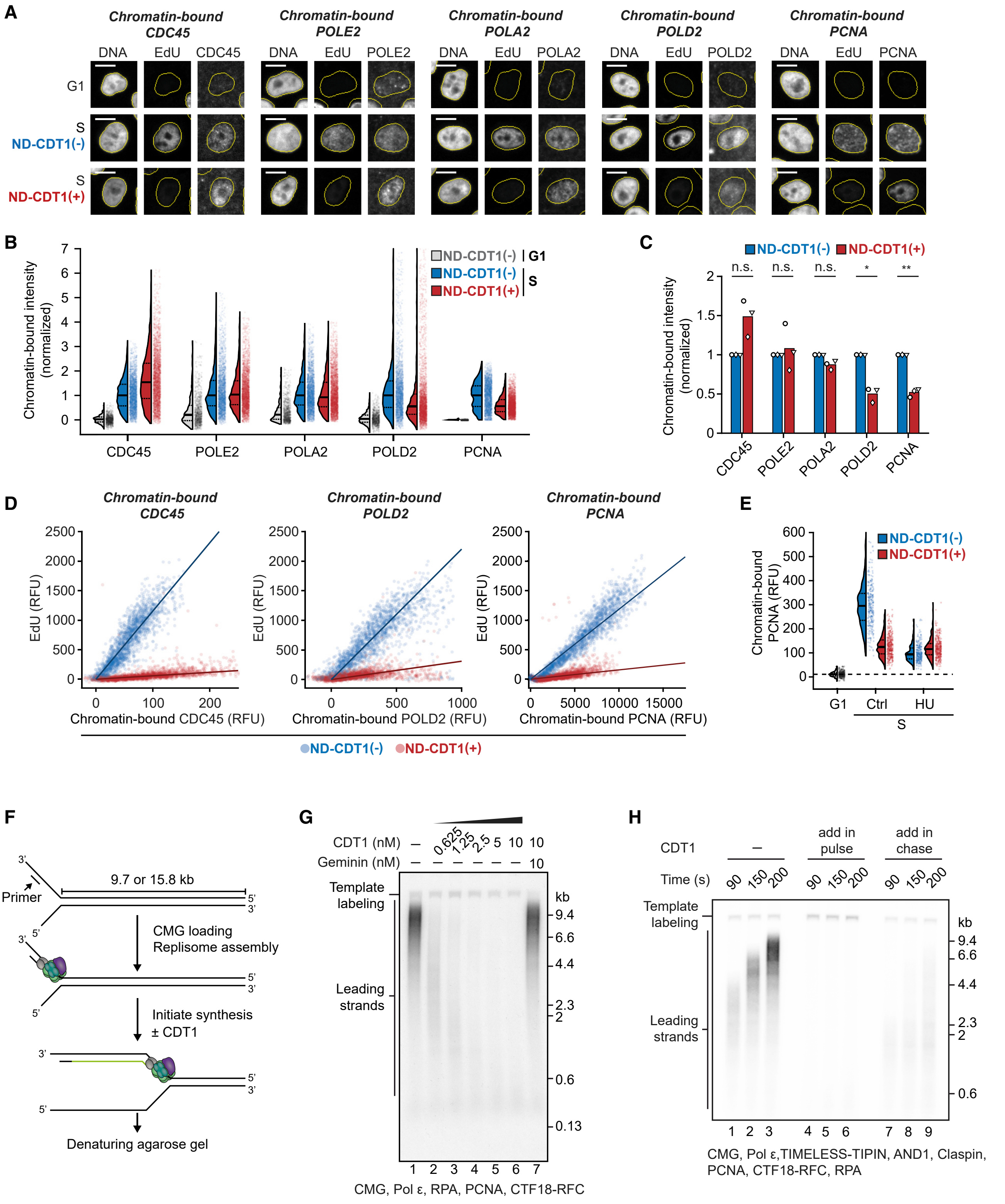

Figure 6. CDT1 inhibits replication fork elongation while permitting origin firing

(A–E) RT-QIBC of chromatin-bound replisome components in mitogen-released MCF10A cells. G1 and S cells were identified (see STAR Methods). Analysis for additional proteins in Figures S6A–S6E.

(A) Representative images of EdU and CDC45, Pol ε (POLE2), Pol α (POLA2), Pol δ (POLD2), and PCNA. Scale bars, 10 μm.

(legend continued on next page)

when CDT1 is bound by geminin.[5] In *Xenopus* egg extracts, truncations in CDT1 that overlapped with MCM-binding regions interfered with the inhibition of DNA synthesis by CDT1, as did geminin addition.[37] Given that a minimal replisome consisting of CMG, Pol ε, and RPA is inhibited by CDT1 *in vitro* (Figure S6H), and that Pol ε is not intrinsically inhibited by CDT1 (Figure 7C), we hypothesized that the same regions in CDT1 that mediate licensing by binding MCM might also interact with and inhibit the activated CMG helicase complex (which contains MCMs). Consistent with this hypothesis, chromatin-bound ND-CDT1 co-localized with chromatin-bound CDC45, a component of CMG helicase (Figures 7D, S7A, and S7B). Since ND-CDT1 cannot bind PCNA due to its lack of PIP degron,[16] the co-localization was not the result of binding to PCNA at the replication fork.

Finally, we tested whether the MCM-binding regions of CDT1 are necessary for CDT1 to inhibit DNA synthesis in human cells. The first MCM-binding region is found at its C terminus, while a second MCM interaction interface was identified near R210 of human CDT1.[5,20] We overexpressed ND-CDT1 with either a truncation at residue 498 in the C-terminal MCM-binding domain (ND-CDT1$^{\Delta 499–546}$), which abolishes licensing and MCM-binding,[54] or point mutations R198A/R210A in the other interface (ND-CDT1$^{R198A/R210A}$), which severely diminishes licensing activity.[55] We examined their inhibitory effect on DNA synthesis with geminin knocked down to account for potential differential geminin regulation of the mutants. ND-CDT1$^{\Delta 499–546}$ could not inhibit EdU incorporation at all, while ND-CDT1$^{R198A/R210A}$ was impaired in its ability to inhibit EdU, with an IC_{50} approximately double that of normal ND-CDT1 (Figures 7E and S7C). Consistent with this result, geminin, which prevents binding of CDT1 to MCM helicases,[5] also prevented CDT1-mediated inhibition of DNA synthesis (Figure 3F). Together, our different lines of evidence support a model in which CDT1 suppresses DNA synthesis by inhibiting CMG helicase activity.

DISCUSSION

Our study focused on the fundamental problem in eukaryotic DNA replication of how cells duplicate the genome precisely once every cell cycle. It is generally thought that the solution to this problem is the strict temporal separation of origin licensing from origin firing to prevent re-replication. Vertebrate licensing regulation is centered around the inhibition of licensing factor CDT1 from S-phase entry to anaphase through CDT1 degradation by CRL4^{Cdt2} and SCF^{Skp2}, and inhibition by geminin and cyclin A.[5] However, only CRL4^{Cdt2} was proposed to prevent re-licensing in early S phase, and, given the dependence of CRL4^{Cdt2} activity on PCNA bound to replication forks, it has been noted this mechanism cannot fully separate licensing and firing in early S phase.[1,4,17] Considering the large number of replication origins, even a short overlap of CDT1 and fired origins while CDT1 is being degraded could allow for the re-licensing of DNA, raising the question of how cells might prevent re-replication during this vulnerable period.

In this work, we identified an overlap period of CDT1 protein with fired origins in early S phase that lasts approximately 30 min in human cells, during which geminin and cyclin A levels are still low. Strikingly, we found that CDT1 inhibits DNA synthesis during this overlap period, and this inhibition is only relieved once CDT1 is fully degraded. We found that CDT1 suppresses CMG helicase, and thus replication fork elongation, at fired origins through its MCM-binding domains. By suppressing replication fork elongation after origin firing, CDT1 allows its own degradation by CRL4^{Cdt2} to be completed before DNA synthesis commences (Figure 7F). Importantly, this mechanism is robust toward changes in CDT1 expression levels, as cells with higher amounts of CDT1 that take longer to degrade would suppress DNA synthesis longer.

Mechanistically, the ability of CDT1 to suppress CMG helicase during fork elongation while allowing origin firing implies that CDT1 does not prevent the initial origin unwinding performed by CMG during origin firing, and only acts afterward on CMG at elongating forks. One possible explanation could be that origin firing factors, which bind and activate MCM helicases and then are released, can prevent CDT1 from inhibiting CMG specifically during origin firing.

Previous studies have identified responses to re-replication and DNA damage in human cells that reduce DNA synthesis in response to aberrant CDT1 regulation.[7,35,54,56] Critically, most characterized mechanisms require that cells first re-replicate DNA, and the resulting reduced DNA synthesis serves to minimize further damage. In contrast, the CMG helicase inhibition by CDT1 identified in our study can act before re-replication is produced.

Nevertheless, the finding that CDT1 can directly inhibit DNA synthesis still raises the question of why dysregulated CDT1 can result in re-replication and DNA damage at all.[6–9,57] A likely explanation is that the small amount of DNA synthesis in the presence of CDT1 can, over long periods, allow for enough residual DNA synthesis to produce re-replication. Furthermore, since overexpressed or dysregulated CDT1 might be incompletely degraded, CDT1 could be reduced to levels too low for effective

(B) G1 mode intensities were subtracted off signals, and values were normalized to ND-CDT1(−) median. n = 2,000 cells per condition.

(C) Summary of median values from 3 independent experiments of Figure 6B. One-sample Student's t test: p values CDC45 (n.s.): 6.94 × 10^{-2}, POLE2 (n.s.):.693, POLA2 (n.s.): 6.41 × 10^{-2}, POLD2 (*): 1.21 × 10^{-2}, PCNA (**): 4.3 × 10^{-3}.

(D) EdU vs. replisome components in S cells. Fit line from linear regression (n = 2,000 cells per condition).

(E) Cells were treated with 2 mM hydroxyurea (HU) during final 4 h of imaging and then fixed. Dashed line is median G1 signal. n $\geq$ 281 cells per condition.

(F) CMG is loaded onto forked DNA template, replisome is assembled, and replication is initiated in the presence or absence of CDT1. Synthesized DNA is radiolabeled for visualization (green strand).

(G) Denaturing agarose gel analysis of 6 min replication reactions (15.8 kbp DNA template) with indicated proteins. 10 nM geminin was pre-incubated with equimolar CDT1 where indicated. CMG-independent template labeling is marked.

(H) Denaturing agarose gel analysis of pulse-chase experiment (15.8 kbp DNA template) with the indicated proteins. Synthesized DNA was labeled in pulse, and chase was added after 50 s. 10 nM CDT1 was added with pulse or chase.

Dashed and solid lines in violin plots are IQR and median. Data representative of 3 independent experiments.

See also Figure S6.

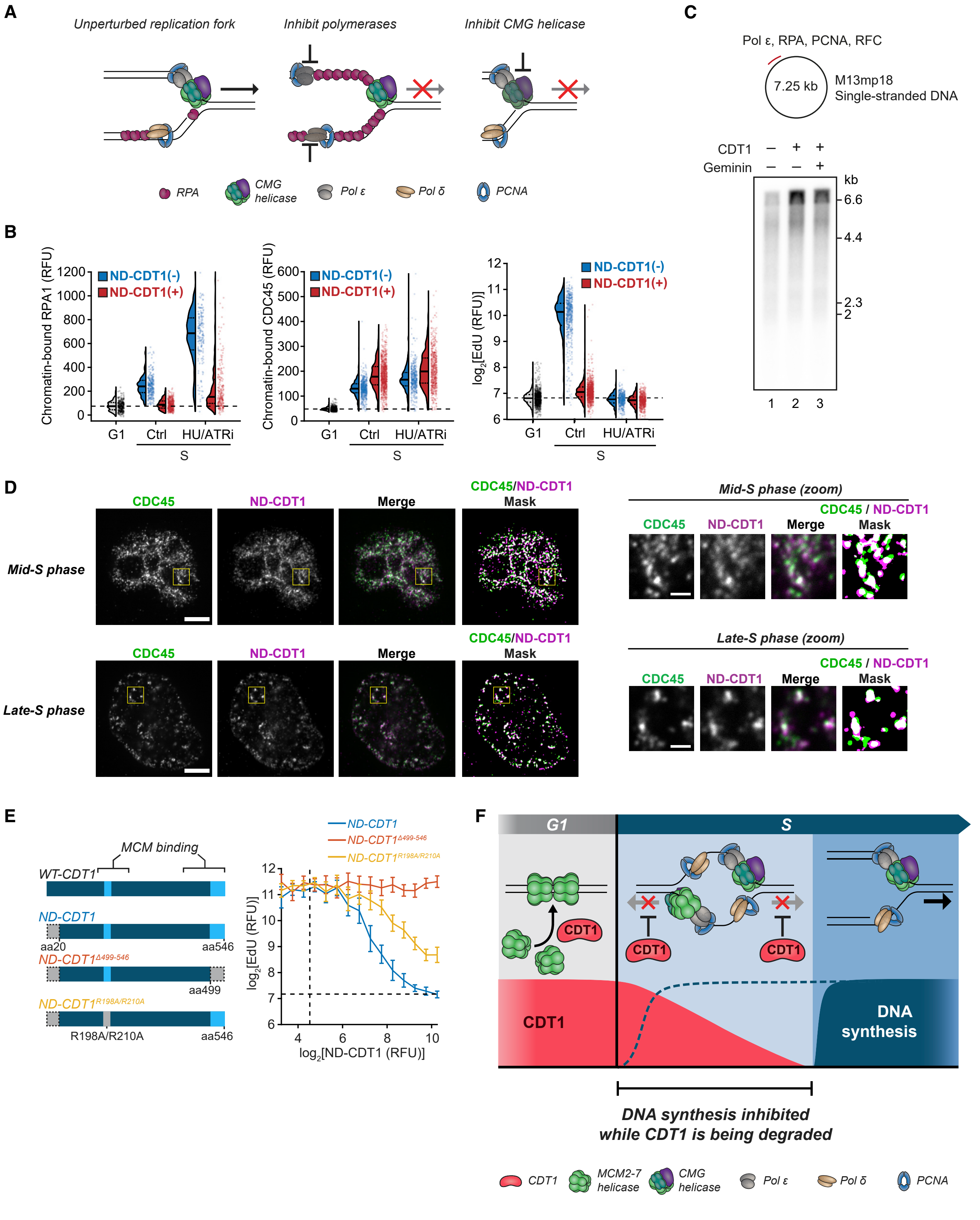

(legend on next page)

suppression of DNA synthesis, but high enough for some re-licensing to occur over time. In support of this, non-degradable mutants of CDT1 paradoxically produce less re-replication than wild-type CDT1.[54,56]

Overall, our study shows that licensing and firing have an overlap period, arguing for a revision of the concept that origin licensing must be separated from origin firing to prevent re-replication. Instead, cells separate origin licensing from DNA synthesis in early S phase. Importantly, we identify that this separation is enforced by CDT1 inhibiting the CMG helicase after origin firing until CDT1 is fully degraded. All previously identified re-replication prevention mechanisms center around the inhibition of licensing factors as cells enter S phase.[1] In contrast, we identified a new class of licensing regulation whereby a licensing factor itself inhibits S-phase progression. We propose that both classes of regulation are critical for safeguarding genome integrity.

Limitations of the study

Based on (1) CDT1 reducing ssDNA produced by CMG, (2) *in vitro* assays showing CDT1 inhibits CMG-dependent DNA synthesis but does not inhibit Pol ε itself, and (3) the dependence of inhibition on the MCM-binding domains of CDT1, we argue that CDT1 directly inhibits CMG helicase. However, the data cannot exclude the possibility that CDT1 impacts another aspect of replisome function, such as helicase-polymerase coupling, and thereby reduces helicase activity. Additional *in vitro* assays examining the CMG activity in the absence of polymerase will be needed to distinguish these possibilities. Furthermore, while we found that inhibition of CMG helicase by CDT1 is dependent on the MCM-binding domain of CDT1, we have yet to elucidate the structural and biophysical basis of this inhibition. Finally, while our work in human cells and work in *Xenopus* egg extract suggests that CDT1 inhibits DNA synthesis in vertebrates, future work is needed to examine whether this mechanism is conserved other organisms such as yeast and *Drosophila*, which many studies have used as model systems to study DNA replication.

STAR★METHODS

Detailed methods are provided in the online version of this paper and include the following:

- KEY RESOURCES TABLE
- RESOURCE AVAILABILITY
  - Lead contact
  - Materials availability
  - Data and code availability
- EXPERIMENTAL MODEL AND SUBJECT DETAILS
  - Cell culture
  - Cell line generation
- METHOD DETAILS
  - Cell cycle reporters
  - Plasmid generation
  - siRNA and plasmid transfection
  - Drugs
  - *In vitro* replication assays
  - Western blot
  - Fixed-cell sample preparation
  - Microscopy
  - Experimental details
- QUANTIFICATION AND STATISTICAL ANALYSIS
  - Image analysis
  - Cell gating
  - Quantification corrections
  - Data normalization
  - Statistical analysis
  - Visualization

SUPPLEMENTAL INFORMATION

Supplemental information can be found online at https://doi.org/10.1016/j.molcel.2022.12.004.

ACKNOWLEDGMENTS

We thank Michael Hodskinson for purification of CDT1. We thank Arne Lindqvist for providing RPE-1 *TP53*$^{-/-}$ *CDC6*$^{d/d}$ cells; Hana Sedlackova and Jiri Lukas for providing U2OS CDC45-GFP cells; Yilin Fan, Lindsey Pack, Anjali Bisaria, Damien Garbett, Steven Cappell, and Arnold Hayer for technical support; Meredith Weglarz and the Stanford Shared FACS Facility for cell sorting; Lindsey Pack, Yilin Fan, and Katherine Ferrick for critical reading of the manuscript; and Karlene Cimprich, James Ferrell, Gheorghe Chistol, Daniel Jarosz, and all members of the Meyer laboratory for helpful discussions. This work was funded by a National Institute of General Medical Sciences (NIGMS) R35 grant (5R35GM127026-05) to T.M. and by MRC as part of UK Research and Innovation (MRC grant MC_UP_1201/12 to J.T.P.Y.). N.R. was supported by an NSF Graduate Research Fellowship (DGE-1147470). Y.B. was supported by an LMB Cambridge Trust International Scholarship.

Figure 7. CDT1 inhibits CMG helicase through its MCM-binding domains

(A) Mechanisms underlying inhibited replication fork progression.

(B) Mitogen-released MCF10A cells were treated with 2 mM hydroxyurea (HU) and 2 μM AZ-20 (ATRi) during the final 4 h. G1 and S cells were identified (see STAR Methods). n ≥ 182 cells per condition. Dashed line is G1 signal median. Representative of 3 independent experiments.

(C) Top: polymerase epsilon primer extension assay (M13mp18 circular single-stranded DNA template). Bottom: denaturing agarose gel analysis of 15 min primer extension reactions. 10 nM CDT1 pre-incubated with equimolar geminin where indicated. Representative of 3 independent experiments.

(D) CDC45-GFP U2OS cells with doxycycline (Dox-inducible ND-CDT1 were treated with Dox for 8 h, pre-extracted, and co-stained for ND-CDT1(anti-HA-tag) and CDC45 (anti-GFP). Mid- or late-S-phase cells identified by CDC45 pattern. Signals were masked by thresholding. Yellow box is zoomed region. Representative of 35 (mid S) or 18 (late S) cells. Scale bars, 5 μm (full image) and 1 μm (zoom). Co-localization analysis in Figure S7B.

(E) Left: MCM-binding region mutants of ND-CDT1. WT-CDT1, wild-type CDT1. Right: dose responses of EdU to ND-CDT1 (anti-HA-tag), 1–2 h after S entry in mitogen-released siGeminin-treated MCF10A cells. Error bars are mean ± 2 × SEM (n ≥ 26 cells per bin, n ≥ 1,048 cells per condition). Dashed lines are negative threshold. Mutant expression in Figure S7C.

(F) Diagram of CDT1 regulation of DNA synthesis in early S.

See also Figure S7.

AUTHOR CONTRIBUTIONS

Conceptualization, N.R., M.C., and T.M.; methodology, N.R., M.C., Y.B., J.T.P.Y., and T.M.; software, N.R.; formal analysis, N.R.; investigation, N.R., Y.B., and M.C.; data curation, N.R.; writing – original draft, N.R. and T.M.; writing – review & editing, N.R., M.C., Y.B., J.T.P.Y., and T.M.; visualization, N.R. and Y.B.; supervision, T.M. and J.T.P.Y.; funding acquisition, T.M. and J.T.P.Y.

DECLARATION OF INTERESTS

The authors declare no competing interests.

Received: July 12, 2022
Revised: September 16, 2022
Accepted: December 8, 2022
Published: January 5, 2023

REFERENCES

1. Arias, E.E., and Walter, J.C. (2007). Strength in numbers: preventing rereplication via multiple mechanisms in eukaryotic cells. Genes Dev. *21*, 497–518. https://doi.org/10.1101/gad.1508907.
2. Diffley, J.F.X. (2011). Quality control in the initiation of eukaryotic DNA replication. Philos. Trans. R. Soc. Lond. B Biol. Sci. *366*, 3545–3553. https://doi.org/10.1098/rstb.2011.0073.
3. Limas, J.C., and Cook, J.G. (2019). Preparation for DNA replication: the key to a successful S phase. FEBS Lett. *593*, 2853–2867. https://doi.org/10.1002/1873-3468.13619.
4. Reusswig, K.-U., and Pfander, B. (2019). Control of eukaryotic DNA replication initiation—mechanisms to ensure smooth transitions. Genes-Basel *10*, 99. https://doi.org/10.3390/genes10020099.
5. Pozo, P.N., and Cook, J.G. (2016). Regulation and function of Cdt1; A key factor in cell proliferation and genome stability. Genes-Basel *8*, 2. https://doi.org/10.3390/genes8010002.
6. Arias, E.E., and Walter, J.C. (2005). Replication-dependent destruction of Cdt1 limits DNA replication to a single round per cell cycle in Xenopus egg extracts. Genes Dev. *19*, 114–126. https://doi.org/10.1101/gad.1255805.
7. Dorn, E.S., Chastain, P.D., Hall, J.R., and Cook, J.G. (2009). Analysis of rereplication from deregulated origin licensing by DNA fiber spreading. Nucleic Acids Res. *37*, 60–69. https://doi.org/10.1093/nar/gkn912.
8. Klotz-Noack, K., McIntosh, D., Schurch, N., Pratt, N., and Blow, J.J. (2012). Re-replication induced by geminin depletion occurs from G2 and is enhanced by checkpoint activation. J. Cell Sci. *125*, 2436–2445. https://doi.org/10.1242/jcs.100883.
9. Vaziri, C., Saxena, S., Jeon, Y., Lee, C., Murata, K., Machida, Y., Wagle, N., Hwang, D.S., and Dutta, A. (2003). A p53-dependent checkpoint pathway prevents rereplication. Mol. Cell *11*, 997–1008. https://doi.org/10.1016/S1097-2765(03)00099-6.
10. Zhou, Y., Pozo, P.N., Oh, S., Stone, H.M., and Cook, J.G. (2020). Distinct and sequential re-replication barriers ensure precise genome duplication. PLoS Genet. *16*. e1008988. https://doi.org/10.1371/journal.pgen.1008988.
11. Bastians, H., Topper, L.M., Gorbsky, G.L., and Ruderman, J.V. (1999). Cell cycle–regulated proteolysis of mitotic target proteins. Mol. Biol. Cell *10*, 3927–3941. https://doi.org/10.1091/mbc.10.11.3927.
12. Geley, S., Kramer, E., Gieffers, C., Gannon, J., Peters, J.-M., and Hunt, T. (2001). Anaphase-promoting complex/cyclosome–dependent proteolysis of human cyclin A starts at the beginning of mitosis and is not subject to the spindle assembly checkpoint. J. Cell Biol. *153*, 137–148. https://doi.org/10.1083/jcb.153.1.137.
13. McGarry, T.J., and Kirschner, M.W. (1998). Geminin, an inhibitor of DNA replication, is degraded during mitosis. Cell *93*, 1043–1053. https://doi.org/10.1016/s0092-8674(00)81209-x.
14. Grant, G.D., Kedziora, K.M., Limas, J.C., Cook, J.G., and Purvis, J.E. (2018). Accurate delineation of cell cycle phase transitions in living cells with PIP-FUCCI. Cell Cycle *17*, 2496–2516. https://doi.org/10.1080/15384101.2018.1547001.
15. Sakaue-Sawano, A., Yo, M., Komatsu, N., Hiratsuka, T., Kogure, T., Hoshida, T., Goshima, N., Matsuda, M., Miyoshi, H., and Miyawaki, A. (2017). Genetically encoded Tools for optical dissection of the mammalian cell cycle. Mol. Cell *68*, 626–640.e5. https://doi.org/10.1016/j.molcel.2017.10.001.
16. Havens, C.G., and Walter, J.C. (2009). Docking of a specialized PIP box onto chromatin-bound PCNA creates a Degron for the ubiquitin ligase CRL4Cdt2. Mol. Cell *35*, 93–104. https://doi.org/10.1016/j.molcel.2009.05.012.
17. Havens, C.G., and Walter, J.C. (2011). Mechanism of CRL4Cdt2, a PCNA-dependent E3 ubiquitin ligase. Genes Dev. *25*, 1568–1582. https://doi.org/10.1101/gad.2068611.
18. Hahn, A.T., Jones, J.T., and Meyer, T. (2009). Quantitative analysis of cell cycle phase durations and PC12 differentiation using fluorescent biosensors. Cell Cycle *8*, 1044–1052. https://doi.org/10.4161/cc.8.7.8042.
19. Leonhardt, H., Rahn, H.-P., Weinzierl, P., Sporbert, A., Cremer, T., Zink, D., and Cardoso, M.C. (2000). Dynamics of DNA replication factories in living cells. J. Cell Biol. *149*, 271–280. https://doi.org/10.1083/jcb.149.2.271.
20. Pozo, P.N., Matson, J.P., Cole, Y., Kedziora, K.M., Grant, G.D., Temple, B., and Cook, J.G. (2018). Cdt1 variants reveal unanticipated aspects of interactions with cyclin/CDK and MCM important for normal genome replication. Mol. Biol. Cell *29*, 2989–3002. https://doi.org/10.1091/mbc.E18-04-0242.
21. Toledo, L.I., Altmeyer, M., Rask, M.-B., Lukas, C., Larsen, D.H., Povlsen, L.K., Bekker-Jensen, S., Mailand, N., Bartek, J., and Lukas, J. (2013). ATR prohibits replication catastrophe by preventing global exhaustion of RPA. Cell *155*, 1088–1103. https://doi.org/10.1016/j.cell.2013.10.043.
22. Cappell, S.D., Chung, M., Jaimovich, A., Spencer, S.L., and Meyer, T. (2016). Irreversible APC Cdh1 inactivation underlies the point of no return for cell-cycle entry. Cell *166*, 167–180. https://doi.org/10.1016/j.cell.2016.05.077.
23. Cappell, S.D., Mark, K.G., Garbett, D., Pack, L.R., Rape, M., and Meyer, T. (2018). EMI1 switches from being a substrate to an inhibitor of APC/CCDH1 to start the cell cycle. Nature *558*, 313–317. https://doi.org/10.1038/s41586-018-0199-7.
24. Gookin, S., Min, M., Phadke, H., Chung, M., Moser, J., Miller, I., Carter, D., and Spencer, S.L. (2017). A map of protein dynamics during cell-cycle progression and cell-cycle exit. PLOS Biol. *15*. e2003268. https://doi.org/10.1371/journal.pbio.2003268.
25. Stallaert, W., Kedziora, K.M., Taylor, C.D., Zikry, T.M., Ranek, J.S., Sobon, H.K., Taylor, S.R., Young, C.L., Cook, J.G., and Purvis, J.E. (2022). The structure of the human cell cycle. Cell Syst. *13*, 230–240.e3. https://doi.org/10.1016/j.cels.2021.10.007.
26. Ligasová, A., and Koberna, K. (2021). Strengths and weaknesses of cell synchronization protocols based on inhibition of DNA synthesis. Int. J. Mol. Sci. *22*, 10759. https://doi.org/10.3390/ijms221910759.
27. Håland, T.W., Boye, E., Stokke, T., Grallert, B., and Syljuåsen, R.G. (2015). Simultaneous measurement of passage through the restriction point and MCM loading in single cells. Nucleic Acids Res. *43*, e150. https://doi.org/10.1093/nar/gkv744.
28. Matson, J.P., Dumitru, R., Coryell, P., Baxley, R.M., Chen, W., Twaroski, K., Webber, B.R., Tolar, J., Bielinsky, A.-K., Purvis, J.E., et al. (2017). Rapid DNA replication origin licensing protects stem cell pluripotency. eLife *6*, e30473. https://doi.org/10.7554/eLife.30473.
29. Lin, J.J., Milhollen, M.A., Smith, P.G., Narayanan, U., and Dutta, A. (2010). NEDD8-targeting drug MLN4924 elicits DNA rereplication by stabilizing Cdt1 in S phase, triggering checkpoint activation, apoptosis, and

senescence in cancer cells. Cancer Res. *70*, 10310–10320. https://doi.org/10.1158/0008-5472.CAN-10-2062.

30. Lan, H., Tang, Z., Jin, H., and Sun, Y. (2016). Neddylation inhibitor MLN4924 suppresses growth and migration of human gastric cancer cells. Sci. Rep. *6*, 24218. https://doi.org/10.1038/srep24218.
31. McIntosh, D., and Blow, J.J. (2012). Dormant origins, the licensing checkpoint, and the response to replicative stresses. Cold Spring Harb. Perspect. Biol. *4*. a012955. https://doi.org/10.1101/cshperspect.a012955.
32. Shreeram, S., Sparks, A., Lane, D.P., and Blow, J.J. (2002). Cell type-specific responses of human cells to inhibition of replication licensing. Oncogene *21*, 6624–6632. https://doi.org/10.1038/sj.onc.1205910.
33. Davidson, I.F., Li, A., and Blow, J.J. (2006). Deregulated replication licensing causes DNA fragmentation consistent with head-to-tail fork collision. Mol. Cell *24*, 433–443. https://doi.org/10.1016/j.molcel.2006.09.010.
34. Liu, E., Lee, A.Y.-L., Chiba, T., Olson, E., Sun, P., and Wu, X. (2007). The ATR-mediated S phase checkpoint prevents rereplication in mammalian cells when licensing control is disrupted. J. Cell Biol. *179*, 643–657. https://doi.org/10.1083/jcb.200704138.
35. Truong, L.N., and Wu, X. (2011). Prevention of DNA re-replication in eukaryotic cells. J. Mol. Cell Biol. *3*, 13–22. https://doi.org/10.1093/jmcb/mjq052.
36. Nakazaki, Y., Tsuyama, T., Seki, M., Takahashi, M., Enomoto, T., and Tada, S. (2016). Excess Cdt1 inhibits nascent strand elongation by repressing the progression of replication forks in Xenopus egg extracts. Biochem. Biophys. Res. Commun. *470*, 405–410. https://doi.org/10.1016/j.bbrc.2016.01.028.
37. Nakazaki, Y., Tsuyama, T., Azuma, Y., Takahashi, M., and Tada, S. (2017). Mutant analysis of Cdt1's function in suppressing nascent strand elongation during DNA replication in Xenopus egg extracts. Biochem. Biophys. Res. Commun. *490*, 1375–1380. https://doi.org/10.1016/j.bbrc.2017.07.034.
38. Tsuyama, T., Watanabe, S., Aoki, A., Cho, Y., Seki, M., Enomoto, T., and Tada, S. (2009). Repression of nascent strand elongation by deregulated Cdt1 during DNA replication in Xenopus egg extracts. Mol. Biol. Cell *20*, 937–947. https://doi.org/10.1091/mbc.e08-06-0613.
39. Chung, M., Liu, C., Yang, H.W., Köberlin, M.S., Cappell, S.D., and Meyer, T. (2019). Transient hysteresis in CDK4/6 activity underlies passage of the restriction point in G1. Mol. Cell *76*, 562–573.e4. https://doi.org/10.1016/j.molcel.2019.08.020.
40. Spencer, S.L., Cappell, S.D., Tsai, F.-C., Overton, K.W., Wang, C.L., and Meyer, T. (2013). The proliferation-quiescence decision is controlled by a bifurcation in CDK2 activity at mitotic exit. Cell *155*, 369–383. https://doi.org/10.1016/j.cell.2013.08.062.
41. Daigh, L.H., Liu, C., Chung, M., Cimprich, K.A., and Meyer, T. (2018). Stochastic endogenous replication stress causes ATR-triggered fluctuations in CDK2 activity that dynamically adjust global DNA synthesis rates. Cell Syst. *7*, 17–27.e3. https://doi.org/10.1016/j.cels.2018.05.011.
42. Neelsen, K.J., Zanini, I.M.Y., Mijic, S., Herrador, R., Zellweger, R., Ray Chaudhuri, A.R., Creavin, K.D., Blow, J.J., and Lopes, M. (2013). Deregulated origin licensing leads to chromosomal breaks by rereplication of a gapped DNA template. Genes Dev. *27*, 2537–2542. https://doi.org/10.1101/gad.226373.113.
43. Lemmens, B., Hegarat, N., Akopyan, K., Sala-Gaston, J., Bartek, J., Hochegger, H., and Lindqvist, A. (2018). DNA replication determines timing of mitosis by restricting CDK1 and PLK1 activation. Mol. Cell *71*, 117–128.e3. https://doi.org/10.1016/j.molcel.2018.05.026.
44. Kubota, S., Fukumoto, Y., Ishibashi, K., Soeda, S., Kubota, S., Yuki, R., Nakayama, Y., Aoyama, K., Yamaguchi, N., and Yamaguchi, N. (2014). Activation of the prereplication complex is blocked by mimosine through reactive oxygen species-activated ataxia telangiectasia mutated (ATM) protein without DNA damage. J. Biol. Chem. *289*, 5730–5746. https://doi.org/10.1074/jbc.M113.546655.
45. Burgers, P.M.J., and Kunkel, T.A. (2016). Eukaryotic DNA replication fork. Annu. Rev. Biochem. *86*, 1–22. https://doi.org/10.1146/annurev-biochem-061516-044709.
46. Lee, K.Y., Fu, H., Aladjem, M.I., and Myung, K. (2013). ATAD5 regulates the lifespan of DNA replication factories by modulating PCNA level on the chromatin. J. Cell Biol. *200*, 31–44. https://doi.org/10.1083/jcb.201206084.
47. Sirbu, B.M., Couch, F.B., Feigerle, J.T., Bhaskara, S., Hiebert, S.W., and Cortez, D. (2011). Analysis of protein dynamics at active, stalled, and collapsed replication forks. Genes Dev. *25*, 1320–1327. https://doi.org/10.1101/gad.2053211.
48. Yu, C., Gan, H., Han, J., Zhou, Z.-X., Jia, S., Chabes, A., Farrugia, G., Ordog, T., and Zhang, Z. (2014). Strand-specific analysis shows protein binding at replication forks and PCNA unloading from lagging strands when forks stall. Mol. Cell *56*, 551–563. https://doi.org/10.1016/j.molcel.2014.09.017.
49. Baris, Y., Taylor, M.R.G., Aria, V., and Yeeles, J.T.P. (2022). Fast and efficient DNA replication with purified human proteins. Nature *606*, 204–210. https://doi.org/10.1038/s41586-022-04759-1.
50. Mansilla, S.F., de la Vega, M.B.D.L., Calzetta, N.L., Siri, S.O., and Gottifredi, V. (2020). CDK-independent and PCNA-dependent functions of p21 in DNA Replication. Genes (Basel) *11*, 593. https://doi.org/10.3390/genes11060593.
51. Tsanov, N., Kermi, C., Coulombe, P., der Laan, S.V., Hodroj, D., and Maiorano, D. (2014). PIP degron proteins, substrates of CRL4Cdt2, and not PIP boxes, interfere with DNA polymerase η and κ focus formation on UV damage. Nucleic Acids Res. *42*, 3692–3706. https://doi.org/10.1093/nar/gkt1400.
52. Zeman, M.K., and Cimprich, K.A. (2014). Causes and consequences of replication stress. Nat. Cell Biol. *16*, 2–9. https://doi.org/10.1038/ncb2897.
53. Frigola, J., He, J., Kinkelin, K., Pye, V.E., Renault, L., Douglas, M.E., Remus, D., Cherepanov, P., Costa, A., and Diffley, J.F.X. (2017). Cdt1 stabilizes an open MCM ring for helicase loading. Nat. Commun. *8*, 15720. https://doi.org/10.1038/ncomms15720.
54. Teer, J.K., and Dutta, A. (2008). Human Cdt1 lacking the evolutionarily conserved region that interacts with MCM2–7 is capable of inducing re-replication*. J. Biol. Chem. *283*, 6817–6825. https://doi.org/10.1074/jbc.M708767200.
55. De Marco, V., Gillespie, P.J., Li, A., Karantzelis, N., Christodoulou, E., Klompmaker, R., van Gerwe, S., Fish, A., Petoukhov, M.V., Iliou, M.S., et al. (2009). Quaternary structure of the human Cdt1-Geminin complex regulates DNA replication licensing. Proc. Natl. Acad. Sci. USA *106*, 19807–19812. https://doi.org/10.1073/pnas.0905281106.
56. Takeda, D.Y., Parvin, J.D., and Dutta, A. (2005). Degradation of Cdt1 during S phase is Skp2-independent and is required for efficient progression of mammalian cells through S phase. J. Biol. Chem. *280*, 23416–23423. https://doi.org/10.1074/jbc.M501208200.
57. Arias, E.E., and Walter, J.C. (2006). PCNA functions as a molecular platform to trigger Cdt1 destruction and prevent re-replication. Nat. Cell Biol. *8*, 84–90. https://doi.org/10.1038/ncb1346.
58. Yang, H.W., Cappell, S.D., Jaimovich, A., Liu, C., Chung, M., Daigh, L.H., Pack, L.R., Fan, Y., Regot, S., Covert, M., et al. (2020). Stress-mediated exit to quiescence restricted by increasing persistence in CDK4/6 activation. eLife *9*, e44571. https://doi.org/10.7554/eLife.44571.
59. Sedlackova, H., Rask, M.-B., Gupta, R., Choudhary, C., Somyajit, K., and Lukas, J. (2020). Equilibrium between nascent and parental MCM proteins protects replicating genomes. Nature *587*, 297–302. https://doi.org/10.1038/s41586-020-2842-3.
60. Dull, T., Zufferey, R., Kelly, M., Mandel, R.J., Nguyen, M., Trono, D., and Naldini, L. (1998). A third-generation lentivirus vector with a conditional packaging system. J. Virol. *72*, 8463–8471. https://doi.org/10.1128/JVI.72.11.8463-8471.1998.

61. Stewart, S.A., Dykxhoorn, D.M., Palliser, D., Mizuno, H., Yu, E.Y., An, D.S., Sabatini, D.M., Chen, I.S.Y., Hahn, W.C., Sharp, P.A., et al. (2003). Lentivirus-delivered stable gene silencing by RNAi in primary cells. Rna *9*, 493–501. https://doi.org/10.1261/rna.2192803.

62. Schindelin, J., Arganda-Carreras, I., Frise, E., Kaynig, V., Longair, M., Pietzsch, T., Preibisch, S., Rueden, C., Saalfeld, S., Schmid, B., et al. (2012). Fiji: an open-source platform for biological-image analysis. Nat. Methods *9*, 676–682. https://doi.org/10.1038/nmeth.2019.

63. Bisaria, A., Hayer, A., Garbett, D., Cohen, D., and Meyer, T. (2020). Membrane-proximal F-actin restricts local membrane protrusions and directs cell migration. Science *368*, 1205–1210. https://doi.org/10.1126/science.aay7794.

64. Jones, M.L., Baris, Y., Taylor, M.R.G., and Yeeles, J.T.P. (2021). Structure of a human replisome shows the organisation and interactions of a DNA replication machine. EMBO J. *40*. e108819. https://doi.org/10.15252/embj.2021108819.

65. Lin, J.-R., Fallahi-Sichani, M., and Sorger, P.K. (2015). Highly multiplexed imaging of single cells using a high-throughput cyclic immunofluorescence method. Nat. Commun. *6*, 8390. https://doi.org/10.1038/ncomms9390.

66. Gut, G., Herrmann, M.D., and Pelkmans, L. (2018). Multiplexed protein maps link subcellular organization to cellular states. Science *361*. eaar7042. https://doi.org/10.1126/science.aar7042.

67. Salic, A., and Mitchison, T.J. (2008). A chemical method for fast and sensitive detection of DNA synthesis in vivo. Proc. Natl. Acad. Sci. USA *105*, 2415–2420. https://doi.org/10.1073/pnas.0712168105.

68. Allen, M., Poggiali, D., Whitaker, K., Marshall, T.R., Langen van, J., and Kievit, R.A. (2019). Raincloud plots: a multi-platform tool for robust data visualization. Wellcome Open Res. *4*, 63. https://doi.org/10.12688/wellcomeopenres.15191.1.

69. Mazo, G. (2021). QuickFigures: A toolkit and ImageJ PlugIn to quickly transform microscope images into scientific figures. PLoS One *16*. e0240280. https://doi.org/10.1371/journal.pone.0240280.

STAR★METHODS

KEY RESOURCES TABLE

REAGENT or RESOURCE	SOURCE	IDENTIFIER
Antibodies		
rabbit anti-CDT1 mAb [EPR17891]	Abcam	Cat# ab202067, RRID:AB_2651122
rabbit anti-Geminin pAb	Atlas Antibodies	Cat# HPA049977, RRID:AB_2680978
mouse anti-Cyclin A mAb [B-8]	Santa Cruz Biotech	Cat# sc-271682, RRID:AB_10709300
mouse anti-PCNA mAb [PC10]	Santa Cruz Biotech	Cat# sc-56, RRID:AB_628110
rabbit anti-MCM2 mAb [D7G11] XP	Cell Signaling Technology	Cat# 3619, RRID:AB_2142137
rabbit anti-p21 mAb [12D1]	Cell Signaling Technology	Cat# 2947, RRID:AB_823586
rabbit anti-HA tag mAb [C29F4]	Cell Signaling Technology	Cat# 3724, RRID:AB_1549585
rabbit anti-CDC45 mAb [D7G6]	Cell Signaling Technology	Cat# 11881, RRID:AB_2715569
rabbit anti-POLA2 pAb	Atlas Antibodies	Cat# HPA037570, RRID:AB_10672280
rabbit anti-POLD2 pAb	Atlas Antibodies	Cat# HPA026745, RRID:AB_1855520
rabbit anti-POLE2 pAb	Atlas Antibodies	Cat# HPA027555, RRID:AB_10610282
rabbit anti-Timeless mAb [EPR5275]	Abcam	Cat# ab109512, RRID:AB_10863023
rabbit anti-phospho-Chk1(S317) mAb [D12H3]	Cell Signaling Technology	Cat# 12302, RRID:AB_2783865
rabbit anti-phospho-Chk2(T68) pAb	Cell Signaling Technology	Cat# 2661, RRID:AB_331479
rabbit anti-phospho-histone H2A.X(S139) pAb	Cell Signaling Technology	Cat# 2577, RRID:AB_2118010
rabbit anti-RPA70/RPA1 mAb [EPR3472]	Abcam	Cat# ab79398, RRID:AB_1603759
mouse anti-CDC6 mAb [180.2]	Santa Cruz Biotech	Cat# sc-9964, RRID:AB_627236
rabbit anti-GAPDH mAb [D16H11] XP	Cell Signaling Technology	Cat# 5174, RRID:AB_10622025
mouse anti-GFP [9F9.F9] mAb	Abcam	Cat# ab1218, RRID:AB_298911
rabbit anti-p27 mAb [D69C12] XP mAb	Cell Signaling Technology	Cat# 3686, RRID:AB_2077850
rabbit anti-Geminin pAb	Proteintech	Cat# 10802-1-AP, RRID:AB_2110945
mouse anti-GAPDH (6C5) mAb	Santa Cruz Biotech	Cat# sc-32233, RRID:AB_627679
goat anti-rabbit IgG, HRP-linked Antibody	Cell Signaling Technology	Cat# 7074, RRID:AB_2099233
horse anti-mouse IgG, HRP-linked Antibody	Cell Signaling Technology	Cat# 7076, RRID:AB_330924
goat anti-rabbit IgG Alexa Fluor 647	Thermo Fisher Scientific	Cat# A-21245, RRID:AB_2535813
goat anti-rabbit IgG Alexa Fluor 514	Thermo Fisher Scientific	Cat# A31558, RRID:AB_10375589
goat anti-mouse IgG Alexa Fluor 647	Thermo Fisher Scientific	Cat# A-21235, RRID:AB_2535804
goat anti-mouse IgG Alexa Fluor 514	Thermo Fisher Scientific	Cat# A-31555, RRID:AB_2536171
goat anti-mouse IgG Alexa Fluor 488	Thermo Fisher Scientific	Cat# A-11029, RRID:AB_2534088
goat anti-rabbit IgG Alexa Fluor 568	Thermo Fisher Scientific	Cat# A-11036, RRID:AB_10563566
Chemicals, peptides, and recombinant proteins		
DMSO	Sigma-Aldrich	Cat# D2650
Hydroxyurea	Cayman Chemical	Cat# 23725
AZ-20 (ATRi)	Cayman Chemical	Cat# 17589
MK-1775 (WEE1i)	Cayman Chemical	Cat# 21266
Doxycycline hyclate (Dox)	Sigma-Aldrich	Cat# D9891
MLN-4924	Abcam	Cat# ab216470
indole-3 acetic acid (auxin)	MP Biomedicals	Cat# 0210203705
BMS-650032	Adooq Bioscience	Cat# A112955
L-mimosine	Cayman Chemical	Cat# 14337
Hoechst 33342	Invitrogen	Cat# H3570

(*Continued on next page*)

Continued

REAGENT or RESOURCE	SOURCE	IDENTIFIER
5-ethynyl-2′-deoxyuridine (EdU)	Cayman Chemical	Cat# 20518
AFDye 488 picolyl azide	Click Chemistry Tools	Cat# 1276
AFDye 647 picolyl azide	Click Chemistry Tools	Cat# 1300
DAPI	Thermo Fisher Scientific	Cat# 62248
thymidine	Sigma-Aldrich	Cat# T9250-1G
aphidicolin	Sigma-Aldrich	Cat# 5047440001
Recombinant human CMG (CDC45, MCM2, MCM3, MCM4, MCM5, MCM6, MCM7, PSF1, PSF2, PSF3, SLD5)	This study	N/A
Recombinant human Pol ε (POLE1, POLE2, POLE3, POLE4)	This study	N/A
Recombinant human Pol α (POLA1, POLA2, PRIM1, PRIM2)	This study	N/A
Recombinant human Pol δ (POLD1, POLD2, POLD3, POLD4)	This study	N/A
Recombinant human CTF18-RFC (CTF18, CTF8, DCC1, RFC2, RFC3, RFC4, RFC5)	This study	N/A
Recombinant human TIMELESS-TIPIN (TIMELESS, TIPIN)	This study	N/A
Recombinant human AND1	This study	N/A
Recominant human CLASPIN	This study	N/A
Recombinant human PCNA	This study	N/A
Recombinant human RPA (RPA1, RPA2, RPA3)	This study	N/A
Recombinant human CDT1	This study	N/A
Recombinant human Geminin	Abcam	Cat# ab86447
Deposited data		
Data for plotting figures	Dryad (This study)	https://doi.org/10.5061/dryad.4xgxd2599
Original images and uncropped gels	Mendeley Data (This study)	https://doi.org/10.17632/6c63g8jjg7.1
Experimental models: Cell lines		
Human: MCF-10A	ATCC	Cat# CRL-10317, RRID:CVCL_0598
Human: MCF-10A(H2B-Turq + EYFP-PCNA)	This study	N/A
Human: MCF-10A(H2B-Turq + EYFP-PCNA + TetOn-CDT1-mCherry)	This study	N/A
Human: MCF-10A(H2B-Turq + EYFP-PCNA + TetOn-CDT1ΔCy-mCherry)	This study	N/A
Human: MCF-10A(H2B-Turq + EYFP-PCNA + TetOn-CDT1ΔPIP-mCherry)	This study	N/A
Human: MCF-10A(H2B-Turq + C-CRL4^{Cdt2} reporter + APC/C reporter)	This study	N/A
Human: MCF-10A(H2B-Turq + C-CRL4^{Cdt2} reporter + APC/C reporter + TetOn-GemininΔDbox)	This study	N/A
Human: MCF-10A(H2B-Turq + N-CRL4^{Cdt2} reporter + APC/C reporter)	This study	N/A
Human: MCF-10A(H2B-Turq + N-CRL4^{Cdt2} reporter + APC/C reporter + TetOn-ND-CDT1-HA)	This study	N/A
Human: MCF-10A(H2B-Turq + N-CRL4^{Cdt2} reporter + APC/C reporter + TetOn-ND-CDT1Δ499-546-HA)	This study	N/A

(*Continued on next page*)

Continued

REAGENT or RESOURCE	SOURCE	IDENTIFIER
Human: MCF-10A(H2B-Turq + N-CRL4^{Cdt2} reporter + APC/C reporter + TetOn-ND-CDT1R198A/R210A-HA)	This study	N/A
Human: MCF-10A(H2B-Turq + N-CRL4^{Cdt2} reporter + Cyclin E/A-CDK reporter + TetOn-ND-CDT1-HA)	This study	N/A
Human: MCF-10A(H2B-Turq + APC/C reporter + TetOn-CDT1-mCherry)	This study	N/A
Human: MCF-10A(H2B-Turq + APC/C reporter + TetOn-ND-CDT1-mCherry)	This study	N/A
Human: MCF-10A(H2B-Turq + APC/C reporter + Cyclin E/A-CDK reporter)	Meyer Laboratory; Cappell et al.[22]	N/A
Human: MCF-10A(H2B-Turq + APC/C reporter + Cyclin E/A-CDK reporter + CDK4/6 reporter)	Meyer Laboratory; Yang et al.[58]	N/A
Human: MCF-10A(H2B-Turq + N-CRL4^{Cdt2} reporter + APC/C reporter + Cyclin E/A-CDK reporter)	This study	N/A
Human: RPE-1 *TP53*$^{-/-}$ *CDC6*$^{d/d}$	Laboratory of Arne Lindqvist ; Lemmens et al.[43]	N/A
Human: RPE-1 *TP53*$^{-/-}$ *CDC6*$^{d/d}$ (H2B-Turq + APC/C reporter + TetOn-ND-CDT1-mCherry)	This study	N/A
Human: RPE-1 *TP53*$^{-/-}$ *CDC6*$^{d/d}$ (H2B-Turq + APC/C reporter + TetOn-NLS-mCherry)	This study	N/A
Human: U2OS	ATCC	Cat#HTB-96, RRID:CVCL_0042
Human: U2OS (H2B-Turq + N-CRL4^{Cdt2} reporter + APC/C reporter + Cyclin E/A-CDK reporter)	This study	N/A
Human: U2OS GFP-CDC45	Laboratory of Jiri Lukas; Sedlackova et al.[59]	N/A
Human: U2OS GFP-CDC45(TetOn-ND-CDT1-mCherry)	This study	N/A
Human: U2OS GFP-CDC45(TetOn-ND-CDT1-HA)	This study	N/A
Human: HeLa	ATCC	Cat#CCL-2, RRID:CVCL_0030
Human: HeLa(H2B-Turq + APC/C reporter)	This study	N/A
**Oligonucleotides**		
siRNA	See Table S1	N/A
**Recombinant DNA**		
tFucci(CA)2/pCSII-EF (N-CRL4^{Cdt2} reporter + APC/C reporter)	RIKEN BRC: Laboratory of Atsushi Miyawaki; Sakaue-Sawano et al.[15]	Cat# RDB15446
pLV-hCDT1(1-100)ΔCy-mCherry-P2A-mVenus-hGeminin(1-110)-IRES-Blast (C-CRL4^{Cdt2} reporter + APC/C reporter)	This study	Addgene # 193139
pLV-mCherry-hCDT1(1-100)ΔCy (N-CRL4^{Cdt2} reporter)	This study	Addgene # 193759
CSII-pEF1a-H2B-mTurquoise	Meyer Laboratory; Spencer et al.[40]	N/A
CSII-pEF1a-hDHB(994-1087)-mVenus (Cyclin E/A-CDK reporter)	Meyer Laboratory; Spencer et al.[40]	Addgene # 136461
CSII-pEF1a-mVenus-hGeminin(1-110) (APC/C reporter)	Meyer Laboratory	N/A

(*Continued on next page*)

Continued		
REAGENT or RESOURCE	SOURCE	IDENTIFIER
pLV-mCherry-hGeminin(1-110)-IRES-Puro (APC/C reporter)	Meyer Laboratory	N/A
pLV-H2B-miRFP670	Meyer Laboratory	N/A
pLV-hDHB(994-1087)-mTurquoise (Cyclin E/A-CDK reporter)	Meyer Laboratory	N/A
pLV-EYFP-PCNA	Meyer Laboratory; Hahn et al.[18]	N/A
pLV-rtTA3-IRES-Puro	Meyer Laboratory	N/A
pLV-TetOn-CDT1-mCherry	This study	Addgene # 193760
pLV-TetOn-ND-CDT1-mCherry	This study	Addgene # 193761
pLV-TetOn-NLS-mCherry	This study	Addgene # 193762
pCW-CDT1-mCherry-Puro	This study	Addgene # 193763
pCW-CDT1ΔCy-mCherry-Puro	This study	Addgene # 193764
pCW-CDT1ΔPIP-mCherry-Puro	This study	Addgene # 193765
pCW-ND-CDT1-mCherry-Puro	This study	Addgene # 193766
pCW-ND-CDT1-HA-Puro	This study	Addgene # 193767
pCW-ND-CDT1Δ499-546-HA-Puro	This study	Addgene # 193768
pCW-ND-CDT1ΔR198A/R210A-HA-Puro	This study	Addgene # 193769
pCW-GemininΔDbox-HA-Puro	This study	Addgene # 193770
pC1-ND-CDT1-mCitrine	This study	Addgene # 193771
pMDLg/pRRE	Addgene: Laboratory of Didier Trono; Dull et al.[60]	Addgene # 12251
pRSV-rev	Addgene: Laboratory of Didier Trono; Dull et al.[60]	Addgene # 12253
pCMV-VSV-G	Addgene: Laboratory of Bob Weinberg; Stewart et al.[61]	Addgene # 8454
2X FLAG-CDT1-pACEBac1	This study	N/A
Software and algorithms		
Image processing software (MATLAB)	This study (https://github.com/MeyerLab/image-analysis-ratnayeke-2022)	https://doi.org/10.5281/zenodo.7183750
MATLAB R2020a	MathWorks	N/A
ImageJ v1.53c (Fiji distribution)	Schindelin et al.[62]	N/A
QuickFigures (ImageJ plugin)	Mazo 2020	N/A
Other		
96-well glass bottomed plates	Cellvis	Cat# P96-1.5H-N
Lipofectamine 2000	Thermo Fisher Scientific	Cat# 11668019
DharmaFECT 1	Dharmacon	Cat# T-2001-03

RESOURCE AVAILABILITY

Lead contact

Further information and requests for resources and reagents should be directed to and will be fulfilled by the lead contact, Tobias Meyer (tom4003@med.cornell.edu).

Materials availability

Plasmids and cell lines generated in this study are available upon request to lead contact.

Data and code availability

- Original microscopy images and uncropped gel images corresponding to images shown in the figures have been deposited to Mendeley Data and are publicly available as of the date of publication. Full raw imaging datasets generated in this study cannot be deposited in a public repository due to storage limitations but are available from the lead contact upon request. Processed data (single-cell quantifications) generated from image datasets that were used for analysis and generation of the figures in this

study have been deposited at Dryad and are publicly available as of the date of publication. DOIs are listed in the key resources table.

- All original code, including custom MATLAB image-processing pipeline and scripts used to generate the figures from this study, have been deposited at Github (https://github.com/MeyerLab/image-analysis-ratnayeke-2022) and Zenodo, and are publicly available as of the date of publication. Zenodo DOI is listed in the key resources table.
- Any additional information required to reanalyze the data reported in this paper is available from the lead contact upon request.

EXPERIMENTAL MODEL AND SUBJECT DETAILS

Cell culture

All experiments were performed with MCF10A human mammary epithelial cells (ATCC Cat# CRL-10317, RRID:CVCL_0598) unless otherwise noted. MCF10A cells were cultured in DMEM/F12 growth media with HEPES (Gibco Cat# 11039047), supplemented with 5% horse serum (Gibco Cat# 16050122), 20 ng/mL EGF (PeproTech Cat# AF-100-15), 0.5 μg/mL hydrocortisone (Sigma: H0888), 100 ng/mL cholera toxin (Sigma Cat# C8052) and 10 μg/mL insulin (Sigma Cat# I1882). Cells were passaged using trypsin-EDTA (0.05%, Gibco Cat# 25300054) and trypsin was neutralized in DMEM/F12 supplemented with 20% horse serum. RPE-1 *TP53*$^{-/-}$ cells with double-degron endogenous-tagged CDC6 (RPE-1 *TP53*$^{-/-}$ *CDC6*$^{d/d}$) were a kind gift from Arne Lindqvist[43] and cultured in DMEM/F12 with HEPES supplemented with 10% FBS (Sigma Cat# TMS-013-B). U2OS cells (ATCC Cat#HTB-96, RRID:CVCL_0042) and U2OS cells with endogenously GFP-tagged CDC45 (a kind gift from Jiri Lukas[59]) were cultured in DMEM growth media (Gibco Cat# 11995065) with 10% FBS. HeLa cells (ATCC Cat#CCL-2, RRID:CVCL_0030) were cultured in DMEM growth media with 10% FBS. For MCF10A serum-starvation prior to mitogen-release, cells were cultured in starvation media (growth media without horse serum, EGF, and insulin and supplemented with 0.3% BSA) after two washes of starvation media. For mitogen-release, starvation media was exchanged with growth media. All cells were cultured at 37°C and 5% CO2. For microscopy experiments, 96-well glass-bottomed plates (Cellvis Cat# P96-1.5H-N) were collagen-coated (Advanced Biomatrix Cat# 5005-B, 60 μg/mL dilution 2 h – 24 h), and cells were seeded into wells at least the night before performing experiments.

Cell line generation

Cell cycle reporter cell lines were generated using third-generation lentiviral transduction.[60,61] In short, lentivirus was produced in HEK-293T cells co-transfected with packaging plasmids pMDLg/pRRE (Addgene # 12251, RRID:Addgene_12251), pRSV-rev(Addgene # 1225, RRID:Addgene_12253), and pCMV-VSV-G (Addgene # 8454, RRID:Addgene_8454) together with the lentiviral plasmid with Lipofectamine 2000 (Thermo Cat# 11668019). 72 h after transfection, virus was collected from the supernatant, filtered with a .22 μm filter (Millipore Cat# SCGP00525), and concentrated using 100 kDa centrifugal filters (Millipore Cat# UFC910024). Virus was then transduced into cells in growth media. For constitutively expressed fluorescent constructs, positive fluorescent cells were sorted using a BD Influx cell sorter (performed in Stanford Shared FACS Facility), while Dox-inducible constructs (TetOn in pCW backbone with puromycin selection marker) were selected with 1 μg/mL puromycin until control cells died unless otherwise stated. TetOn cells were grown in the absence of Dox until the time of the experiment unless otherwise stated. See Table S1 for a list of cell lines with reporter combinations.

All MCF10A reporter cell lines were generated from a base cell line transduced with CSII-pEF1a-H2B-mTurquoise or pLV-H2B-miRFP670 as a nuclear tracking marker. Cells with EYFP-PCNA or the APC/C^{Cdh1} reporter were generated by transducing H2B-mTurquoise cells with pLV-EYFP-PCNA or CSII-pEF1a-mVenus-hGeminin(1-110) respectively. Cells containing the APC/C^{Cdh1} reporter together with either N- or C-CRL4^{Cdt2} reporter were generated by transducing H2B-mTurquoise cells with bicistronic vector tFucci(CA)2/pCSII-EF[15] or pLV-hCDT1(1-100)ΔCy-mCherry-P2A-mVenus-hGeminin(1-110) respectively. Cells with the Cyclin E/A-CDK reporter together with N-CRL4^{Cdt2} were created by transduction of H2B-mTurquoise cells with CSII-pEF1a-hDHB(994-1087)-mVenus and pLV-mCherry-hCDT1(1-100)ΔCy. MCF10A and U2OS cells with the Cyclin E/A-CDK, APC/C^{Cdh1}, and N-CRL4^{Cdt2} reporters were generated by transduction of H2B-miRFP670 cells with pLV- hDHB(994-1087)-mTurquoise and tFucci(CA)2/pCSII-EF.

pCW constructs (TetOn Dox-inducible) expressing HA or mCherry-tagged CDT1 mutants or Geminin$^{\Delta Dbox}$ were introduced into these fluorescent reporter cell lines in combinations found in the Table S1. For cells with the APC/C^{Cdh1} reporter and TetOn-CDT1-mCherry, cells were transduced with CSII-pEF1a-mVenus-hGeminin(1-110), followed by pLV-rtTA3-IRES-Puro, and then pLV-TetOn-CDT1-mCherry. Cell lines transduced with CDT1-mCherry constructs were induced with 500 ng/mL Dox while serum-starved to sort for expressing cells. mCherry positive cells were sorted, and media was then switched to growth media without Dox. MCF10A cells containing a CDK4/6 reporter (not analyzed), Cyclin E/A-CDK reporter, and APC/C^{Cdh1} reporter used in Figures S4B and S4C were described previously.[58] RPE-1 *TP53*$^{-/-}$ *CDC6*$^{d/d}$ cells were transduced with CSII-pEF1a-H2B-mTurquoise and CSII-pEF1a-mVenus-hGeminin(1-110), and then pLV-TetOn-ND-CDT1-mCherry or pLV-TetOn-NLS-mCherry. U2OS *CDC45-GFP* cells were transduced with pCW-ND-CDT1-mCherry-Puro or pCW-ND-CDT1-HA-Puro. HeLa cells were transduced with CSII-pEF1a-H2B-mTurquoise and pLV-mCherry-hGeminin(1-110)-IRES-Puro.

METHOD DETAILS

Cell cycle reporters

Cell cycle reporters of $CRL4^{Cdt2}$ and APC/C^{Cdh1} activity were used in this study. These reporters were originally developed as the two components of the FUCCI(CA) reporter system.[15] The $CRL4^{Cdt2}$ activity reporter corresponds to $CDT1(1\text{-}100)^{\Delta Cy}$ in the FUCCI(CA) system and is based on a fragment of human CDT1 corresponding to the amino acid 1-100, which is inactive with respect to origin licensing. $CDT1(1\text{-}100)^{\Delta Cy}$ contains a PCNA-interacting protein (PIP) degron, which mediates CDT1 degradation by $CRL4^{Cdt2}$ in response to PCNA at fired origins, and has a removed Cy motif to prevent degradation by SCF^{Skp2}.[15] The $CRL4^{Cdt2}$ reporter is rapidly degraded to low levels at S phase start and reaccumulates at the start of G2. Conversely, the APC/C^{Cdh1} reporter corresponds to $Geminin^{(1\text{-}110)}$ from the FUCCI(CA) system and is based on amino acids 1-110 of human Geminin fused to a fluorescent protein (either mVenus or mCherry). $Geminin^{(1\text{-}110)}$ is degraded at anaphase by APC/C^{Cdc20} and then by APC/C^{Cdh1} throughout G1, and reaccumulates at the time of APC/C^{Cdh1} inactivation at the G1/S transition. Thus, $Geminin^{(1\text{-}110)}$ reports APC/C^{Cdh1} activity at the G1/S transition. APC/C^{Cdh1} inactivation represents a commitment point in the cell cycle and typically occurs near the time of S phase entry and DNA replication, though $CRL4^{Cdt2}$ activation in response to origin firing is an explicit measure of S phase entry.[14,15] While these reporters are typically quantified by their presence or absence in single-timepoint measurements, when reporter fluorescence kinetics are measured in single cells using time-lapse microscopy, the precise time of $CRL4^{Cdt2}$ activation (the start of degradation of the $CRL4^{Cdt2}$ reporter) and APC/C^{Cdh1} inactivation (the stabilization of the APC/C^{Cdh1} reporter) can be identified.

In this study, we used two versions of the $CRL4^{Cdt2}$ reporter, one of which is an N-terminal mCherry-tagged $CDT1(1\text{-}100)^{\Delta Cy}$ (referred to as the N-$CRL4^{Cdt2}$ reporter), which is identical to the construct used in the FUCCI(CA) reporter system. Since the N-$CRL4^{Cdt2}$ reporter is fused to a fluorescent protein on the N-terminus, the PIP degron is in the middle of the construct. Since CDT1 naturally has an N-terminal PIP degron, we hypothesized that reversing the order of the fluorescent protein fusion in a reporter could confer a faster response to the initial origins that are fired in early S phase. As a result, we created a C-terminally tagged $CDT1(1\text{-}100)^{\Delta Cy}$ (referred to as the C-$CRL4^{Cdt2}$ reporter). We found that C-$CRL4^{Cdt2}$ responds with slightly faster kinetics at S phase start than N-$CRL4^{Cdt2}$ (Figure S1H), which was necessary for looking within the first 15-30 min of S phase by RT-QIBC in Figures 1 and 4. However, both reporters are well suited for RT-QIBC looking at times after the first 15-30 min of S phase, and we use both reporters in this study. C-$CRL4^{Cdt2}$ has a similar orientation to the PIP-FUCCI cell cycle reporter,[14] which is based on CDT1(1-17) and is also degraded throughout S phase. We consider the N-$CRL4^{Cdt2}$ (originally used in the FUCCI(CA)) system, C-$CRL4^{Cdt2}$, and PIP-FUCCI reporters to all be reporters of $CRL4^{Cdt2}$ activity and should all be suitable for use with RT-QIBC.

The cyclin E/A-CDK reporter is a translocation-based reporter that is phosphorylated by cyclin E or A complexed with CDK2 or CDK1 (referred to collectively as cyclin E/A-CDK).[40] It is based on a fragment of human DNA helicase B (amino acids 994-1087), which is phosphorylated by cyclin E/A-CDK. When unphosphorylated in G0 and early G1, this reporter is localized in the nucleus, and as cyclin E/A-CDK activity increases throughout the cell cycle, the reporter becomes progressively localized to the cytoplasm due to increased phosphorylation. Thus, the cytoplasm to nuclear ratio of intensity is a readout of cyclin E/A-CDK activity. Critically, the increase in cyclin E/A-CDK activity in G1 is the result of increasing Cyclin E activity, rather than Cyclin A, which is only present after APC/C^{Cdh1} inactivation when it is stabilized (Figure S5I).

Plasmid generation

Plasmids generated in this study were assembled using Gibson assembly of PCR amplified inserts and restriction enzyme digested plasmid backbones. Human full-length CDT1 was amplified out of MCF10A cDNA for CDT1 overexpression, and mutations and tags were introduced through primers or gene synthesis (IDT). ND-CDT1 constructs were created through a truncation of wild-type CDT1 (aa20-546), which removes the CDT1 PIP degron. The CDT1 Cy motif (aa68-70 of full-length CDT1) was mutated to alanine (ΔCy) to prevent degradation by SCF^{Skp2}. This sequence was fused at the C-terminus to a flexible linker, SV40 NLS, and either an mCherry or HA tag. For the $CDT1^{\Delta PIP}$-mCherry construct, CDT1 (aa20-546) was fused to a flexible linker and mCherry at the C-terminus. For $CDT1^{\Delta Cy}$-mCherry construct, full-length CDT1 with the Cy motif mutated to alanine was fused to a flexible linker and mCherry at the C-terminus. ND-$CDT1^{\Delta 499\text{-}546}$ was generated from ND-CDT1 and had a truncating mutation which removed the C-terminal amino acids corresponding to residues 499-546 of CDT1. ND-$CDT1^{R198A/R210A}$ was generated from ND-CDT1 and had alanine mutations introduced at amino acids corresponding to residues 198 and 210 in CDT1. The $Geminin^{\Delta Dbox}$ (human Geminin with R23A and L26A mutations) sequence was generated using gene synthesis and HA-tagged. For Dox-inducible TetOn constructs, PCR products were inserted into the pCW backbone (derived from pCW-Cas9, a gift from Eric Lander & David Sabatini, Addgene plasmid # 50661, RRID:Addgene_50661), a bicistronic vector with a TetOn promoter driving gene expression in addition to a constitutive PGK promoter-driven PuroR-T2A-rtTA. pC1-ND-CDT1-mCitrine was created by cloning ND-CDT1 into the pC1 backbone, derived from C1-F-tractin-mCitrine.[63] pLV-hCDT1(1-100)ΔCy-mCherry-P2A-mVenus-hGeminin(1-110) was generated from full-length CDT1 and Geminin (Human ORFeome V5.1). The N-$CRL4^{Cdt2}$ reporter was amplified from tFucci(CA)2/pCSII-EF, and inserted into the pLV backbone to generate pLV-mCherry-hCDT1(1-100)ΔCy. pLV, CSII and pCW are lentiviral expression plasmids, while pC1 is a mammalian expression plasmid.

siRNA and plasmid transfection

MCF10A cells were transfected with siRNA using DharmaFECT 1 (Dharmacon Cat# T-2001-03) according to the manufacturer's protocol using 20 nM siRNA and 1:500 diluted DharmaFECT 1 final concentration unless otherwise stated. Cells were incubated in transfection mixture for 6-24 h in either growth or serum-starvation media, followed by a media change. Pools of 3-4 siRNA oligos (ON-TARGETplus, Dharmacon) were used for siCtrl, siCDT1, siGeminin (siGMNN), siCyclin A (siCCNA2) and si-p21(siCDKN1A). For siCDT1 and siGeminin, siRNAs that do not target hCDT1(1-100) and hGeminin(1-110) were selected to avoid knockdown of the $CRL4^{Cdt2}$ and APC/C^{Cdh1} reporters, respectively. CCNA2 was knocked down since CCNA1 is not expressed in our cell lines. For siGeminin and siCyclin A in cycling cells (Figures 3A, 3B, S5I, and S5J), cells were transfected for 4–6 h and then immediately live imaged. These proteins can be suppressed even with short siRNA treatment due to their rapid protein degradation starting at anaphase. A list of siRNA oligos is in Table S1. HeLa cells were transiently transfected with pC1-ND-CDT1-mCitrine plasmid using Lipofectamine 2000 according to the manufacturer's protocol using 2 ng/μL final concentration of plasmid complexed with 1:400 diluted Lipofectamine 2000 final concentration. Media was exchanged with growth media after 2 h, and cells were then immediately live-imaged. siRNA and plasmids were both complexed in Opti-MEM serum-free media (Gibco Cat# 31985070).

Drugs

Stock solutions of drugs were dissolved in DMSO (Sigma-Aldrich Cat# D2650) and used at the given working concentration unless otherwise stated: 2 mM hydroxyurea (HU, dissolved in water, Cayman Chemical Cat# 23725), 2 mM thymidine (dissolved in PBS at 100 mM, Sigma-Aldrich Cat# T9250-1G), 2 mg/mL aphidicolin (Sigma-Aldrich Cat# 5047440001), 2 μM AZ-20 (ATRi, Cayman Chemical Cat# 17589), 1 μM MK-1775 (WEE1i, Cayman Chemical Cat# 21266), 1 μg/mL Doxycycline hyclate (Sigma-Aldrich Cat# D9891), 2 μM MLN-4924 (Abcam Cat# ab216470), 500 μM indole-3 acetic acid (auxin, MP Biomedicals Cat# 0210203705), 100 nM BMS-650032 (Adooq Bioscience Cat# A112955), 500 μM L-mimosine (20x stock solution dissolved in DMEM/F12, Cayman Chemical Cat# 14337). For release from mimosine arrest, cells were washed three times in growth media. For all experiments where drugs or Doxycycline were added to cells, DMSO (vehicle) was added to control cells, with the exception of HU, which was dissolved in water.

In vitro replication assays

Protein expression

Proteins used in in vitro replication and primer extension assays were expressed and purified as previously described.[49,64] Recombinant Geminin was purchased from Abcam (Cat# ab86447). The sequence for the expression of N-terminal 2X FLAG-tagged CDT1 (FLAG-tag: MDYKDDDGDYKDDD) was codon optimized for expression in insect cells and synthesized by Epoch Life Science Gene Synthesis. To prepare baculovirus for CDT1 expression, the 2X FLAG-CDT1-pACEBac1 construct was transformed into EMBacY *E. coli* competent cells for bacmid generation. Isolated bacmid was transfected into Sf9 cells using FuGENE® HD (Promega Cat# E2311). Baculovirus was amplified, and the resulting virus was used to infect 1 L of Hi5 cells at a density of 1x 10^6/ mL. Cells were harvested on day 3 after infection.

CDT1 purification

The cell pellet obtained from 1 L of baculovirus-infected culture was resuspended in lysis buffer (50 mM Tris-HCl pH 7.5, 10% glycerol, 0.05% NP-40, 1 mM EDTA, 1 mM DTT) + protease inhibitors (cOmplete, EDTA-free, Roche; one tablet per 50 mL buffer, Sigma-Aldrich Cat# 11873580001). Cells were lysed by dounce homogenization, and insoluble material was removed by centrifugation (235,000*g* at 4 °C for 45 min). 2 mL Anti-FLAG M2 affinity gel (Sigma-Aldrich Cat#A2220) was added to the supernatant and incubated for 3 h at 4 °C. Resin was collected in a disposable gravity flow column (Bio-Rad) and was washed with 100 mL lysis buffer. CDT1 was eluted in 2 mL lysis buffer + 0.4 mg/mL 3× FLAG peptide and 4 mL buffer + 0.2 mg/mL 3× FLAG peptide. Eluates were pooled and applied to 1 mL HiTrap heparin column equilibrated in PBS (137 mM NaCl, 2.7 mM KCl, 10 mM Na_2HPO_4, and 1.8 mM KH_2PO_4) pH 7.5, 0.5 mM TCEP, 10% glycerol. CDT1 was eluted using a 40 column volume gradient to 1 M NaCl. Peak fractions were pooled, frozen in liquid nitrogen, and stored at -80°C.

DNA replication assays

Experiments were performed as previously described.[49] Reactions were conducted in replication buffer (25 mM HEPES-KOH (pH 7.6), 0.01% NP-40, 100 mM potassium glutamate, 1 mM DTT, 10 mM $Mg(OAc)_2$ and 0.1 mg ml^{-1} BSA) at 37°C. Protein and nucleotide concentrations in the final reactions were: 1 nM DNA, 25 nM CMG, 20 nM Pol ε, 20 nM RFC, 20 nM PCNA, 20 nM AND-1, 10 nM Pol α, 5 nM Pol δ, 100 nM RPA, 20 nM CLASPIN, 20 nM TIMELESS–TIPIN, 10 nM CDT1, 10 nM Geminin, 20 nM CTF18−RFC, 4 mM ATP, 30 μM dC/dT/dG/dATP, 200 μM C/G/UTP and 33 nM α-[^{32}P]-dCTP. 2 nM 9.7 kbp or 15.8 kbp linear forked DNA template[49] was incubated with 50 nM CMG for 10 min in replication buffer. The reaction was diluted two-fold by the addition of replication buffer containing dA/dCTP and, as indicated in the figures, PCNA, RFC, Pol ε, Pol α, CLASPIN, TIM–TIPIN, AND-1, CTF18–RFC, and Pol δ. Replication was initiated by addition of a 10x solution containing ATP, dTTP, GTP, CTP, UTP, dGTP, α-[^{32}P]-dCTP and RPA. In reactions containing Pol δ, RFC was added when replication was initiated. Where indicated in the figure legends, 10 nM CDT1 and 10 nM Geminin were pre-incubated on ice for 5 min before addition to the replication reaction. Reactions were stopped by the addition of 50 mM EDTA. Post-reaction processing was performed as previously detailed.[49]

Primer extension assay

Experiments were conducted as previously described.[49] Where indicated in the figure, 10 nM CDT1 was pre-incubated with 10 nM Geminin, and the protein mix was added to the reaction before initiating primer extension by the addition of Pol ε.

Pulse-chase experiments

Experiments were performed as previously described.[49] Replication reaction conditions were applied, except that the concentration of dCTP in the pulse was reduced to 3 μM. The concentrations of dCTP, dGTP, dATP, and dTTP were increased to 600 μM in the chase. The chase was added after 50 s.

Western blot

Cells were grown in 6-well plates. At the time of lysis, cells were washed in ice-cold PBS, lysed in 2x Laemmli sample buffer with 100 mM DTT and a cell scraper, passed through a 25G needle 10 times, and heated at 90°C for 5 min. Samples were then separated with SDS-PAGE using 7.5% or 4-20% Mini-PROTEAN TGX gels (Bio-Rad Cat# 4561025, 4561095) in Tris/Glycine/SDS running buffer (Bio-Rad Cat#161-0772). Proteins were transferred to membranes by semi-dry transfer (Bio-Rad Trans-Blot SD, Cat# 1703940) onto 0.45 μm PVDF membranes (Millipore Cat# IPFL00010) with Tris/Glycine buffer (Bio-Rad Cat# 1610734) + 10% MeOH or semi-dry transfer (Bio-Rad Trans-Blot Turbo Cat# 1704150) onto 0.2 μm PVDF membranes with Trans-Blot Turbo Transfer Packs (Bio-Rad Cat# 1704156). Membranes were washed in TBST (20 mM Tris, pH 7.5, 150 mM NaCl, 0.1% Tween 20), blocked for 30 min in 5% milk in TBST, and incubated overnight with primary antibodies in 5% BSA + 0.01% NaN_3 in TBST. Primary antibodies used were mouse anti-CDC6 antibody (1:500, Santa Cruz Biotech. Cat# sc-9964, RRID:AB_627236), rabbit anti-GAPDH (1:1000, CST Cat# 5174, RRID:AB_10622025), rabbit anti-p21(1:1000, CST Cat# 2947, RRID:AB_823586), rabbit anti-CDT1 (1:1000, Abcam Cat# ab202067, RRID:AB_2651122), rabbit anti-Geminin(1:1000, Proteintech Cat# 10802-1-AP, RRID:AB_2110945) and mouse anti-GAPDH (1:2000, Santa Cruz Biotechnology Cat# sc-32233, RRID:AB_627679). Membranes were then incubated in anti-rabbit or anti-mouse HRP secondary antibodies (1:5000, CST Cat# 7074, RRID:AB_2099233 or CST Cat# 7076, RRID:AB_330924) for 30 min in 5% BSA + 0.01% NaN_3 in TBST, treated with chemiluminescent substrate (Thermo Cat # 34580) and detected on film (Thomas Sci. Cat# EK-5130) and scanned, or imaged directly using a Licor Odyssey Fc. For membrane reblotting (with antibodies of different species), membranes were washed in TBST and treated as in the first round, starting with blocking.

Fixed-cell sample preparation

General protocol

Staining and imaging were performed in 96-well glass-bottomed plates (Cellvis Cat# P96-1.5H-N). Cells were fixed in 4% paraformaldehyde in PBS (diluted from Fisher Cat# NC1537886) for 10 min at room temperature, followed by PBS wash. If cells expressed fluorescent proteins which spectrally overlapped with the fluorophores used in later steps, the fluorescent proteins were chemically bleached[65] in 3% H_2O_2 + 20 mM HCl in PBS for 1 h, washed in PBS, and checked under a microscope to ensure there was negligible residual signal. If fluorescent proteins needed to be quantified in fixed cells prior to immunofluorescence, cells were initially imaged before bleaching and reimaged after staining. For PCNA and CDC45 staining, cells were incubated in ice-cold methanol for 15 min after fixation and then washed in PBS. Cells were permeabilized in 0.2% Triton X-100 in PBS for 10 min and then blocked in blocking buffer A (10% FBS, 1% BSA, 0.1% Triton X-100, 0.01% NaN_3 in PBS) for 1 h. Cells were then incubated with primary antibodies overnight in blocking buffer A at 4°C, washed in PBS, and then incubated with secondary antibodies in blocking buffer A for 1 h at RT. Cells were washed with PBS and then incubated in 1 μg/mL Hoechst 33342 (Invitrogen Cat# H3570) or in 1 μg/mL DAPI (Thermo Cat#62248) in PBS for 10 min, followed by a final PBS wash prior to imaging. Unless otherwise stated, all washes were done with an automated plate washer (aspirate to 50 μL, dispense 250 μL, repeated 9 times, BioTek 405 LS) or by hand (for pre-extracted samples, 3 washes aspirating all liquid).

Iterative immunofluorescence

If simultaneously staining for targets with antibodies of the same species, the iterative indirect immunofluorescence imaging (4i) technique was used to sequentially image multiple antibodies.[66] In short, the first round of imaging was identical to the general immunofluorescence protocol, with the exception that cells after the post-Hoechst PBS wash were washed in ddH_2O and then placed in imaging buffer (700 mM N-acetyl cysteine in ddH_2O, pH 7.4, Sigma-Aldrich A7250). Cells were imaged and then washed in ddH_2O. The prior-round antibodies were eluted by 3×10-min incubations in elution buffer, which consists of 0.5M glycine (Sigma-Aldrich), 3M urea (Sigma-Aldrich), 3M guanidinium chloride (Sigma-Aldrich) and 70 mM TCEP-HCl (Goldbio Cat#TCEP50) in ddH_2O, pH 2.5, followed by a PBS wash. Cells were then checked under a fluorescence microscope to ensure proper elution. Cells were then blocked with blocking buffer B, consisting of 1% BSA in PBS supplemented with 150 mM maleimide (dissolved just prior to use, Sigma-Aldrich Cat# 129585) for 1 h and then washed in PBS. Cells were then blocked with blocking buffer A for 30 min, followed by primary antibody incubation, and the subsequent steps were the same as in the first round, repeated as needed. Control wells leaving out primary antibodies were always included to ensure there was no residual signal from prior rounds of imaging.

Pre-extraction for chromatin-bound protein

If chromatin-bound proteins were being stained for, soluble proteins were extracted from cells. Just prior to fixation, media was aspirated off cells, and the plate was placed on ice. Cells were incubated in ice-cold pre-extraction buffer, consisting of 0.2% Triton X-100 (Sigma-Aldrich Cat# x100) + 1x Halt Protease Inhibitor Cocktail (Thermo Cat# 78439) in the selected aqueous buffer. For all proteins pre-extracted except RPA1, pre-extraction buffer was made with PBS, while CSK buffer was used for RPA1, consisting of 10 mM

PIPES (Sigma-Aldrich), 100 mM NaCl (Sigma-Aldrich), 300 mM sucrose (Sigma-Aldrich), 3 mM $MgCl_2$ (Sigma-Aldrich) at pH 7.0. After a set extraction time, 8% PFA in H_2O was directly added to wells 1:1 with wide-orifice tips to minimize cell detachment, and cells were fixed for 25 min at room temperature, after which the sample was treated with the general staining protocol. Extraction times were: 4-5 min (PCNA, MCM2, TIMELESS), 3 min (POLA2, POLD2, POLE2), and 2 min (ND-CDT1 and CDC45-GFP in U2OS SoRa imaging and MCM2 in RPE-1).

EdU incorporation and labeling

If measuring 5-ethynyl-2′-deoxyuridine (EdU) incorporation, cells were pulsed with 50 μM EdU (Cayman Chemical Cat# 20518) in growth media for 8 min prior to fixation and pre-extraction, unless otherwise stated. EdU is incorporated throughout the EdU pulse, such that incorporated EdU reflects the average rate of DNA synthesis over the length of the pulse. Thus an 8 min short EdU pulse is more reflective of the instantaneous DNA synthesis rate compared to a longer pulse such as 1 h. After blocking cells (prior to primary antibodies), cells were washed once with PBS, and then a click reaction[67] was performed in 2 mM $CuSO_4$, 20 mg/mL sodium ascorbate in TBS (Tris 50 mM, NaCl 150 mM pH 8.3) with 3 μM AFDye 488 picolyl azide (Click Chemistry Tools Cat# 1276) or AFDye 647 picolyl azide (Click Chemistry Tools Cat# 1300) for 30 min, followed by a PBS wash.

Antibodies

The following primary antibodies were used for immunofluorescence: rabbit anti-CDT1 (1:500, Abcam Cat# ab202067, RRID:AB_2651122), rabbit anti-Geminin (1:800, Atlas Antibodies Cat# HPA049977, RRID:AB_2680978), mouse anti-Cyclin A (1:250, Santa Cruz Biotech Cat# sc-271682, RRID:AB_10709300), mouse anti-PCNA (1:200, Santa Cruz Biotech. Cat# sc-56, RRID:AB_628110), rabbit anti-MCM2 (1:800, CST Cat# 3619, RRID:AB_2142137), rabbit anti-p21 (1:2500, CST Cat# 2947, RRID:AB_823586), rabbit anti-p27 (1:1600, Cell Signaling Technology Cat# 3686, RRID:AB_2077850), rabbit anti-HA tag (1:1000, CST Cat# 3724, RRID:AB_1549585), mouse anti-GFP (1:500, Abcam Cat# ab1218, RRID:AB_298911), rabbit anti-CDC45 (1:100, CST Cat# 11881, RRID:AB_2715569), rabbit anti-POLA2 (1:100, Atlas Antibodies Cat# HPA037570, RRID:AB_10672280), rabbit anti-POLD2 (1:100, Atlas Antibodies Cat# HPA026745, RRID:AB_1855520), rabbit anti-POLE2 (1:100, Atlas Antibodies Cat# HPA027555, RRID:AB_10610282), rabbit anti-Timeless (1:800, Abcam Cat# ab109512, RRID:AB_10863023), rabbit anti-phospho-Chk1(S317) (1:500, CST Cat# 12302, RRID:AB_2783865), rabbit anti-phospho-Chk2(T68) (1:200, CST Cat# 2661, RRID:AB_331479), rabbit anti-phospho-histone H2A.X(S139) (1:500, CST Cat# 2577, RRID:AB_2118010), rabbit anti-RPA70/RPA1 (1:200, Abcam Cat# ab79398, RRID:AB_1603759). The epitopes for anti-CDT1 and anti-Geminin antibodies do not detect hCDT1(1-100)$^{\Delta Cy}$ and hGeminin(1-110) of the CRL4^{Cdt2} and APC/C^{Cdh1} reporters. For secondary antibodies, antibodies targeting the appropriate species and with no spectral overlap were selected from the following and diluted 1:1000: goat anti-rabbit IgG Alexa Fluor 647 (Thermo Cat# A-21245, RRID:AB_2535813), goat anti-rabbit IgG Alexa Fluor 514 (Thermo Cat# A31558, RRID:AB_10375589), goat anti-mouse IgG Alexa Fluor 647 (Thermo Cat# A-21235, RRID:AB_2535804), goat anti-mouse IgG Alexa Fluor 514 (Thermo Cat# A-31555, RRID:AB_2536171), goat anti-mouse IgG Alexa Fluor 488 (Thermo Cat# A-11029, RRID:AB_2534088), goat anti-rabbit IgG Alexa Fluor 568 (Thermo Cat# A-11036, RRID:AB_10563566)

Microscopy

Time-lapse imaging, RT-QIBC, and QIBC

For automated epifluorescence microscopy, cells were imaged using a Ti2-E inverted microscope (Nikon) or ImageXpress Micro XLS microscope (Molecular Devices). For imaging on the Ti2-E, multichannel fluorescent images were acquired using triple-band (ECFP/EYFP/mCherry, Chroma: 89006) or quad-band (DAPI/FITC/TRITC/Cy5, Chroma: 89402) Sedat filter sets using an LED light source (Lumencor Spectra X) and Hamamatsu ORCA-Flash4.0 V3 sCMOS camera. 10x (Nikon CFI Plan Apo Lambda, NA 0.45) or 20x (Nikon CFI Plan Apo Lambda, 0.75 NA) objectives were used to acquire images. For imaging on the ImageXpress, images were taken with appropriate single-band filter sets with a white-light source, using a 10x (Nikon CFI Plan Fluor, NA 0.3) or 20x (Nikon CFI Plan Apo Lambda, 0.75 NA) and Andor Zyla 4.2 sCMOS camera. All images were acquired in 16-bit mode, and acquisition settings were chosen to not saturate the signal. Fluorophores and imaging channels were chosen to minimize bleedthrough, and in the case of detectable bleedthrough, it was corrected using bleedthrough coefficients estimated from single fluorophore controls.

For live-cell time-lapse imaging, 96-well plates were imaged within an enclosed 37°C, 5% CO2 environmental chamber in 200 μL of growth media. 4-9 sites were imaged in each well (with the number of wells imaged varying depending on experiment and imaging interval) every 3-12 min (3 min for measurements of CDT1 in early S phase as in Figure 1E, or in Figure S1E or S1I. Longer intervals for measuring longer-term dynamics). Light exposure to cells was limited by using the minimum exposure necessary to maintain an acceptable signal-to-noise ratio on a per-channel basis, and total light exposure was always limited to below 300 ms per site each timepoint. Images were taken with the 10x objective for all live-cell imaging except for experiments shown in Figures 3A, S1E, S5H, and S1I to maximize the number of cells in the field of view. When performing the live-cell imaging for RT-QIBC, cells were immediately taken off the microscope following the final time point and fixed.

For fixed-cell imaging for RT-QIBC and QIBC, tiled images of the majority of each well (16–36 sites per well) were taken using the 20x objective. When reimaging fixed cells (matching back to either live-cell imaging for RT-QIBC or previous rounds of fixed-cell imaging), the plate position (which can shift slightly when replacing the plate on the microscope) was aligned to approximately the same location and further aligned computationally during image analysis.

Spinning-disk confocal microscopy

For high-resolution live-cell imaging of EYFP-PCNA and CDT1-mCherry (Figures 1B and S1A–S1C), cells were imaged on an automated spinning-disk confocal microscope (Intelligent Imaging Innovations, 3i). This system used a Nikon Ti-E stand, motorized XY stage with piezoelectric Z movement (3i), Andor Zyla 4.2 sCMOS camera, CSU-W1 confocal scanner unit (Yokogawa), and 405/445/488/514/561/640 nm LaserStack (3i), controlled using SlideBook 6 (3i). Cells were imaged in a 37°C environmental chamber (growth media was HEPES buffered) using a 60x/1.35NA oil objective (Nikon) with 2x camera binning. Images at the nucleus midplane were taken every 2-3 min in a 5x5 montage which was stitched together after acquisition. H2B-mTurquoise, EYFP-PCNA, and CDT1-mCherry were imaged using a triple-band 445/515/561 excitation filter set.

For fixed-cell imaging of chromatin-bound ND-CDT1 localization, pre-extracted U2OS CDC45-GFP cells were imaged on a SoRa spinning-disk confocal microscope (Marianas system, 3i). This system was similar to the previously described 3i microscope, except it used a Zeiss Axio Observer 7 stand, ORCA-Fusion BT sCMOS Camera (Hamamatsu), CSU-W1 SoRa confocal scanner unit (Yokogawa), and 405/445/488/514/561/637 nm LaserStack (3i). Cells were stained using rabbit anti-HA and anti-rabbit Alexa Fluor 568 (for detecting ND-CDT1) together with mouse anti-GFP and anti-mouse Alexa Fluor 488 to image endogenously tagged CDC45-GFP, together with Hoechst. Images were taken using the 405, 488, and 561 channels using a quad-band 405/488/561/640 nm excitation filter set (3i), with a 63x/1.4NA Plan-Apochromat Oil M27 objective (Zeiss) and 4x magnification changer and no camera binning. The field of view was manually searched without 4x magnification and low exposure in the 488 channel to identify cells that were in S phase. Cells were then imaged in both channels at 5 Z-positions around the midplane of the nucleus (1 μm spaced, only the midplane is shown). No deconvolution was performed, and controls were tested to ensure there was no spectral bleedthrough or cross-binding of secondary antibodies.

Experimental details

MCF10A mitogen-release experiments

For mitogen-release experiments, MCF10A cells were serum-starved the day following seeding onto plates (Day 1). For experiments with siRNA knockdowns, cells were transfected with siRNA the next day (Day 2) in serum-free media. Media was changed the following day (Day 3, approximately 48 h after serum-starvation). If inducing ND-CDT1, Dox was added during the media change 6 h before mitogen release. On Day 3, cells were mitogen-released with full growth media and live-imaged. For RT-QIBC and QIBC, cells were fixed 18 h after mitogen-release. For drug treatments (hydroxyurea, AZ-20, MK-1775, MLN-4924), drugs were added 14 h after mitogen-release during live-cell imaging.

CDC6 degradation experiments

RPE-1 *TP53*$^{-/-}$ *CDC6*$^{d/d}$ cells[43] had an auxin-inducible degron (mAID), and SMASh-tag knocked into both endogenous *CDC6* loci. Cells contained a Dox-inducible OsTIR1 E3 ubiquitin ligase component which is required for mAID degradation. The addition of auxin to cells triggers the degradation of mAID-containing proteins. The SMASh-tag contains degron that is auto-cleaved after protein translation by a protease domain. Addition of BMS-650032 (BMS) inhibits this auto-cleavage, resulting in protein degradation. Thus, addition of Dox/Auxin/BMS triggers a robust degradation of endogenous CDC6.

For Figures 5F–5H, Dox-inducible ND-CDT1-mCherry or NLS-mCherry were introduced into RPE-1 *TP53*$^{-/-}$ *CDC6*$^{d/d}$ cells with the APC/C^{Cdh1} reporter. Cells were mitogen-released in the presence of mimosine and Dox for 18 h. CDC6 was then degraded by adding auxin and BMS-650032 (BMS) for 4 h, and then cells were released from mimosine arrest for 1.5 h, followed by an EdU pulse and fixation. Control unreleased cells were not released from mimosine arrest.

Protein nomenclature

For simplicity, we refer to several human proteins by their colloquial names. Namely, we refer to CDT1 (encoded by *CDT1* gene), Geminin (encoded by *GMNN* gene), Cyclin A (in our cell lines, only Cyclin A2, encoded by *CCNA2* gene, is expressed), CRL4^{Cdt2} (Cdt2 is encoded by *DTL* gene), APC/C^{Cdh1} (Cdh1 is encoded by *FZR1* gene), SCF^{Skp2}(also known as CRL1^{Skp2}, Skp2 is encoded by *SKP2* gene), CDC6 (encoded by *CDC6* gene), Claspin (encoded by *CLSPN* gene), AND1 (encoded by *WDHD1* gene), CTF18 (encoded by *CHTF18* gene), Chk1 (encoded by *CHEK1* gene), Chk2 (encoded by *CHEK2* gene), p21 (encoded by *CDKN1A* gene), p27 (encoded by *CDKN1B* gene), p53 (encoded by *TP53* gene) and Cyclin E (both Cyclin E1 and E2, encoded by *CCNE1* and *CCNE2* genes). Furthermore, we measure several protein complexes through an individual subunit (all of which are constitutive complexes): DNA polymerases epsilon (measured by subunit POLE2), alpha (measured by subunit POLA2) and delta (measured by subunit POLD2), and RPA (measured by subunit RPA1, also known as RPA70). Furthermore, we use common acronyms for protein complexes defined as follows: CMG (CMG-MCM2-7-GINS), MCM (minichromosome maintenance), Pol α (DNA polymerase alpha), Pol δ (DNA polymerase delta), Pol ε (DNA polymerase epsilon), CRL4 (cullin-RING ubiquitin ligase complex 4), CRL1 (cullin-RING ubiquitin ligase complex 1), SCF (SKP1-CUL1-F-box), CDK (cyclin-dependent kinase), APC/C^{Cdh1} (anaphase-promoting complex/cyclosome with subunit Cdh1), PCNA (proliferating cell nuclear antigen), RPA (replication protein A), CTF18-RFC (RFC: replication factor C, CTF18-RFC refers to RFC containing CTF18), and TIM-TIPIN (TIMELESS, TIPIN complex).

QUANTIFICATION AND STATISTICAL ANALYSIS

Image analysis

Automated analysis of time-lapse imaging of cell cycle reporters, quantitative image-based cytometry (QIBC), and Retrospective Time-lapse Synchronized QIBC (RT-QIBC) were performed using a custom MATLAB (R2020a, MathWorks) pipeline based on previous work.[22] QIBC here is considered to be the high-throughput single-cell quantification of fixed-cell signals (fluorescent proteins, immunofluorescence, EdU staining, DNA stain), while RT-QIBC involves the assignment of QIBC measurements to an explicit time in the cell cycle based on prior time-lapse imaging of cell cycle reporters (N- and C-CRL4^{Cdt2} reporters, APC/C^{Cdh1} reporter, and EYFP-PCNA). In principle, RT-QIBC can be used to quantify any fixed-cell signal (immunofluorescence used in this study, as well as mRNA or DNA FISH, for example) and retrospectively analyze any live-cell reporter or imaging measurements. In this way, the dynamics of fixed cell measurements can be reconstructed with temporal resolution that is only limited by imaging frequency (in this study 3-12 min). The image processing pipeline and code used to generate all figures in this study have been deposited on Github and Zenodo (https://github.com/MeyerLab/image-analysis-ratnayeke-2022, https://doi.org/10.5281/zenodo.7183750), and data can be downloaded at Dryad (https://doi.org/10.5061/dryad.4xgxd2599).

Segmentation and signal quantification

Raw images were flat-field corrected (also known as shading corrected) to correct for uneven sample illumination. Since images output by the sCMOS camera are the sum of a camera offset value together with the actual detected signal (which is proportional to the sample illumination), we subtracted off the camera offset value and then divided the image by an empirically determined illumination profile. This profile was calculated either from the background autofluorescence in areas without cells in live-cell images (aggregated over a large number of sites) or from sample-free wells filled with autofluorescent blocking buffer A for fixed cell imaging. Confocal movies were not flat-field corrected due to a lack of uneven illumination.

For live-cell imaging, nuclei were automatically segmented from H2B signal using a Laplacian of Gaussian blob detector, which in the case of movies with low contrast, was further refined with active contours. For fixed-cell imaging, nuclei were segmented from the Hoechst or DAPI stain using a threshold determined from histogram curvature. Detected nuclei larger than the median object size were checked using a curvature-based object splitting algorithm that splits cells along two points of high perimeter curvature. If there are more than two putative split points, pairs of points are chosen based on pairs with the highest distance along the perimeter between points divided by the Euclidean distance of the points. For multi-round fixed cell imaging, each imaging round was segmented and aligned to a common round of images. A segmentation mask from a single round (typically the first round) was designated the primary mask and used for quantification of all rounds.

To quantify nuclear cell cycle reporters and fixed-cell signals, the background signal was estimated by taking the 25th percentile of pixels outside of a dilated nuclear mask (dilated 7.8 μm for predominantly nuclear signals, 15.6 μm for signals with cytoplasmic components) and subtracted off of images. For chromatin-bound CDC45, POLA2, POLD2, POLE2, and TIMELESS signals, the background was not subtracted during image processing but accounted for later during analysis. The mean and median signal within the nucleus were then calculated, and for signals with a cytoplasmic component, the median signal within a ring outside of the nucleus was calculated (region 0.65 μm to 3.25 μm outside the edge of the nucleus). To quantify the puncta area of EYFP-PCNA, a top-hat filter (3 pixel radius for confocal imaging, 2 pixel radius for wide-field) was applied to the image, and a series of thresholds of different stringencies were manually chosen and applied to minimize false positives and negatives. The total area of pixels above the thresholds was quantified.

Time-lapse tracking

For time-lapse imaging, nuclei were tracked using a nearest-neighbor algorithm between each frame and its previous frame. To increase tracking fidelity, the total nuclear signal (the sum of nuclear intensity) was used as an invariant quantity which does not change significantly between frames. Using this, putative aberrant merging and splitting of nuclei during segmentation could be detected and corrected. Mitotic events are detected when two daughter nuclei are detected within the vicinity of a previous nucleus and have a total nuclear signal which is approximately equal to the previous nucleus.

To match fixed-cells to live cells, fixed-cell images were computationally aligned to live-cell images using 2D cross-correlation, and cells with their associated measurements were assigned to their nearest live-cell neighbor. When matching the 20x fixed-cell images back to 10x live-cell images, live images were resized using bicubic interpolation (for alignment and tracking purposes only), or fixed images were mean-value binned.

Cell cycle annotation of live-cell data

Mitosis was annotated during the process of tracking cells, defined as the separation of the two sets of chromosomes at anaphase. CRL4^{Cdt2} activation (defined as the start of CRL4^{Cdt2} reporter degradation) and CDT1-mCherry degradation start were annotated by subjecting traces following mitosis or mitogen-release to a drop detection algorithm. This algorithm detects degradation of the reporter at a given time based on a set number of frames following it (the number of frames after a corresponds to the minimum detectable time since degradation start, typically ~3 frames). By detecting points using only a set number of frames beyond the degradation point, we avoid biases in the accuracy of detecting cells that just recently degraded compared to cells that degraded much earlier. Points were checked based on the slope and curvature of the trace within the window being low and high enough, respectively, and having a set decrease in the reporter signal (normalized to reporter expression). APC/C^{Cdh1} inactivation (defined as the start of APC/C^{Cdh1} reporter accumulation) was detected in a complementary way, identifying the first point where the slope increases to

a threshold level and the reporter increases from a low level to a threshold-value of persistent increase. All threshold values were empirically determined and validated by eye on at least 200 traces. For both CRL4^{Cdt2} and APC/C^{Cdh1} reporters, the integrated intensity within the nucleus was quantified for trace analysis.

For identification of the start of S phase from PCNA foci, foci were segmented, and the total area of foci was quantified. The transition from G1 to S phase is characterized by an increase of low foci signal to high foci signal. For RT-QIBC analysis, the same algorithm was used for the APC/C^{Cdh1} reporter (as the increase in puncta area mirrors the rise in APC/C^{Cdh1} reporter levels). For confocal imaging, a dual threshold algorithm was used. A high foci signal threshold that robustly identified S phase cells was manually determined (50 pixels), and the first point at which the foci area increased above this threshold and was higher than the previous 4 frames was identified. Since the high foci signal threshold typically identified a point well after S phase entry, the final frame before this high identified point which was below a low threshold (3 pixels) was identified as the true S phase entry point.

RT-QIBC

After automated tracking and quantification of live- and fixed-cell imaging, each cell was associated with its corresponding annotated cell cycle reporter traces, as well as multidimensional fixed-cell measurements from QIBC. Based on this, the time elapsed from a point of interest (such as CRL4^{Cdt2} activation or mitosis) was used to arrange fixed-cell measurements based on time. Conversely, live-cell traces can be selected based on QIBC measurements (such as the expression of ND-CDT1). For analyses with high time resolution (e.g. Figures 1E–1G and S1I), time offsets for each imaging site and well were accounted for based on the order of well acquisition.

Chromatin-bound ND-CDT1 localization analysis

We measured the Pearson correlation coefficient of chromatin-bound ND-CDT1 relative to chromatin-bound CDC45 (which marks CMG at sites of DNA synthesis) as a measure of colocalization. First, nuclei were segmented using Hoechst DNA stain to confine the analysis to signals within the nucleus. ND-CDT1 and CDC45 signals were masked by the nuclear mask, and then the Pearson correlation coefficient was calculated between these signals. To gauge the significance of these correlations, the ND-CDT1 and CDC45 were randomized by shifting the two channels 40 pixels (1.038 μm) relative to each other. Pearson correlation coefficients were calculated between shifted images (only analyzing pixels within the nucleus) in each of 4 shifting directions (up, down, left, and right), and the mean correlation coefficient of these 4 measurements was considered the randomized data for a given cell. To visualize the ND-CDT1 and CDC45 segmented signals in Figure 7D, each signal was processed with a top-hat filter with a 10-pixel radius and then a Gaussian filter with a 1-pixel radius. Signals were segmented by applying a uniform threshold to the filtered images.

Cell gating

To estimate thresholds in an automated manner for determining cells positive and negative for QIBC staining (for example, chromatin-bound PCNA positive cells for S phase cells) or 2N DNA content, cells known to be in either G1 or S phase based on live-cell imaging were identified, and then the 99th percentile (95th for Figure S1I) was chosen as the threshold.

For Dox-inducible cell lines, cells within populations expressed different amounts of protein in response to uniform Dox, with a small subset of cells not inducing protein to detectable levels. To correct for this, cells were selected for analysis based on either immunofluorescence staining of induced-protein at the end of the experiment (e.g. HA-tag, CDT1 or Geminin staining), or in the case of fluorescent protein-tagged protein induction (e.g. ND-CDT1-mCherry), cells were selected based on live-cell imaging of the induced protein. Thresholds were based on uninduced cells.

G1 and S phase cells were identified through a combination of live-cell reporter quantification and fixed-cell stains. In Figure 2A, late G1 to early S cells were defined by 2N DNA and intermediated Cyclin E/A-CDK activity (0.7 – 1.2). In Figure 5A, G1 cells had inactive CRL4^{Cdt2} (reporter not degraded) and 2N DNA. For Figures 6A–6D, G1 cells had active APC/C^{Cdh1} (APC/C^{Cdh1} reporter low) without chromatin-bound PCNA, while S phase cells were 2-3 h after APC/C^{Cdh1} inactivation and chromatin-bound PCNA positive. For Figure 7B, G1 cells had active APC/C^{Cdh1} without chromatin-bound PCNA, while S phase cells were fixed 2-3 h after APC/C^{Cdh1} inactivation and were chromatin-bound PCNA positive. For remaining figures, gating criteria are listed in Figure legends.

Quantification corrections

For experiments with chromatin-bound proteins measured after pre-extraction, there were rare sections of the samples that were incompletely extracted of soluble proteins. As a proxy for extraction efficiency, in experiments with APC/C^{Cdh1} or Cyclin E/A-CDK reporters (which are soluble), the residual fluorescent protein signal was imaged in addition to immunofluorescence. Cells that had high fluorescent protein signal for the reporters were considered incompletely extracted and removed from the analysis.

For the staining of replication factors in Figures 6 and S6, staining was performed in two rounds. In the first round, chromatin-bound replication proteins (CDC45, TIMELESS, POLE2, POLA2, POLD2, PCNA) were stained using Alexa Fluor 647 secondary antibodies simultaneously with EdU staining with AFDye 488. Fluorophores were then bleached for 1 h and re-stained for PCNA using an Alexa Fluor 647 secondary antibody to identify S phase cells. However, there was a low-intensity residual signal from the first round of staining, which was corrected by using an empirically determined residual signal scaling factor.

For Figures S4B and S4C, H2B-iRFP670 was expressed in a bicistronic vector together with the APC/C^{Cdh1} reporter (P2A sequence). As a result, the APC/C^{Cdh1} reporter signal could be normalized by the H2B-iRFP670 signal to control for differential expression of the construct between cells.

For pre-extraction experiments of replication factors (Figures 6 and S6), outliers resulting from incompletely extracted cells and imaging artifacts were removed by removing the top 1 percentile of data. In Figure S3B, the outer 1 percentile of CRL4^{Cdt2} activation delays from APC/C^{Cdh1} inactivation was removed to account for misidentified cell cycle transitions.

When equal numbers of cells were plotted from different conditions, cells were randomly subsampled to equalize conditions without replacement.

Data normalization

For normalized stain quantification, a baseline was calculated from G1 levels of the stain and subtracted off of all values, followed by division by the group to be normalized. For EdU quantification shown on a linear scale (for dose-responses and chromatin-bound stain linear fits), the G1 background signal was subtracted off values for a true zero. For Figures 1F and 1G, measurements were normalized to the median G2 signal of each protein. For Figures 4E, S5F, and S5G, the EdU signal was background subtracted and divided by the Dox(-) EdU signal 1 – 1.2 h after S phase entry to standardize values between replicates. For Figure 5C, the Cyclin E/A-CDK activity at S phase start was subtracted off values to plot the change of Cyclin E/A-CDK activity from S phase start over time.

Statistical analysis

Details of statistical tests can be found in the figure legends. Comparisons were made with either paired *t*-tests (for tests between multiple independent replicate experiments) or two-sample *t*-tests for within-experiment comparisons of measurements with an α of.05.

For linear fits of chromatin-bound stains (Figure 6), a linear model with a fixed zero-intercept was fit using robust fitting with a bisquare weight function (tuning constant of 2). For fitting dose-response curves cells could be stratified based on their ND-CDT1 expression. Single-cell measurements of EdU and the single-cell expression of ND-CDT1 were fit to a Hill equation of the form

$$EdU([NDCdt1]) = EdU_{max} - \frac{EdU_{max} - EdU_{min}}{1 + \left(\frac{IC_{50}}{[NDCdt1]}\right)^{n}}$$

using nonlinear regression. EdU_{max} is the EdU incorporation of cells without ND-CDT1, which was set using cells not expressing ND-CDT1. EdU_{min} represents the minimum EdU incorporation, IC_{50} is the 50% inhibitory concentration of ND-CDT1 concentration $[NDCdt1]$, and n is the Hill coefficient. For Figures 3F and 5D, EdU_{min}, IC_{50} and n were all fit parameters, while for Figures 3G, 3H, S4F, and S4G, EdU_{min} was set based on high levels of ND-CDT1 expression. Nonlinear regression was performed using the Levenberg-Marquardt algorithm (nlinfit() in MATLAB). Initialization parameters for EdU_{inhib} were estimated from the 5th percentile of EdU signal, while EdU_{inhib} was initialized based on the median $[NDCdt1]$ in the cell population, and was n initialized as 1.

For Figure 3F, maximum EdU inhibition (EdU_{min} / EdU_{max}) was estimated from Hill equation fit curve to be 22.0-fold (siCtrl) and 23.0-fold (siGeminin). IC_{50} was 10.2 (siCtrl), and 7.7 (siGeminin). Hill coefficients were 4.2 (siCtrl) and 1.8 (siGeminin). For Figure 5D, maximum EdU inhibition of 25.7-fold (DMSO), 17.6-fold (ATRi), and 17.0-fold (WEE1i). IC_{50} was 5.49 (DMSO), 5.37 (ATRi), and 5.32 (WEE1i).

For bootstrapped estimators, samples were resampled at least 1000 times, and confidence intervals were calculated using the percentile method. For raincloud plots,[68] which are combined violin and jitter plots, the data distribution was estimated using a kernel smoothing density estimate. The solid and dashed lines in the violin plot correspond to the median and inter-quartile range (IQR).

Visualization

All example cells were extracted from full-sized images through MATLAB scripts and selected based on RT-QIBC or time-lapse analysis. For Figure 7D, cells were selected for imaging based on being in early or late S phase. An example cell was chosen and visualized using ImageJ (v1.53, Fiji distribution)[62] with the QuickFigures plugin.[69]

Molecular Cell, Volume *83*

Supplemental information

CDT1 inhibits CMG helicase in early S phase

to separate origin licensing from DNA synthesis

Nalin Ratnayeke, Yasemin Baris, Mingyu Chung, Joseph T.P. Yeeles, and Tobias Meyer

Figure S1

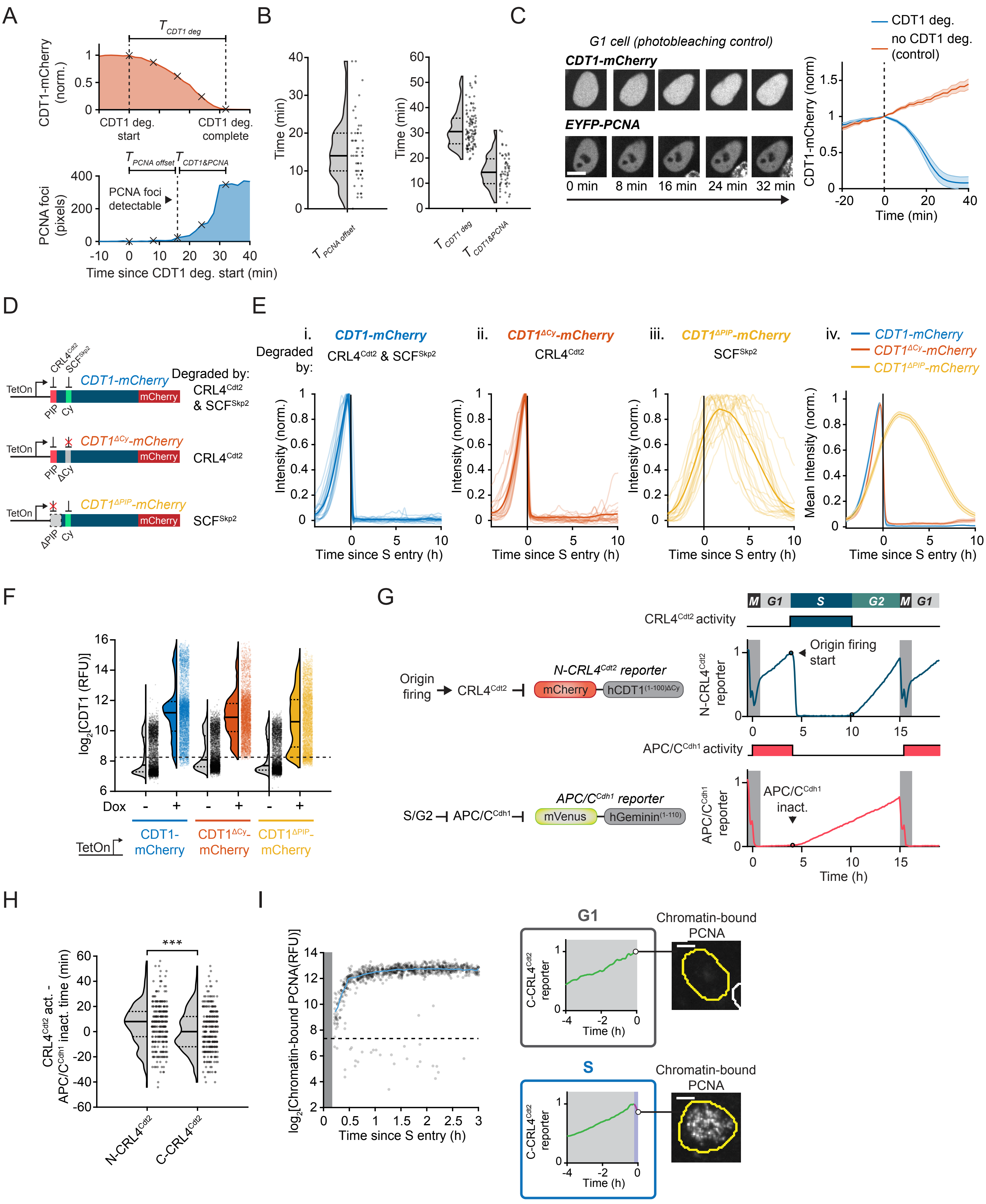

A
B
C
D
E
F
G
H
I
CDT1-mCherry (norm.)
CDT1 deg. start
CDT1 deg. complete
PCNA foci (pixels)
PCNA foci detectable
Time since CDT1 deg. start (min)
Time (min)
G1 cell (photobleaching control)
CDT1-mCherry
EYFP-PCNA
0 min
8 min
16 min
24 min
32 min
CDT1 deg.
no CDT1 deg. (control)
Time (min)
Degraded by:
CDT1-mCherry
Time since S entry (h)
Intensity (norm.)
Mean Intensity (norm.)
Dox
TetOn
Origin firing
N-CRL4Cdt2 reporter
APC/CCdh1 reporter
mCherry
mVenus
Origin firing start
Time (h)
Time since S entry (h)
G1
S
Chromatin-bound PCNA

Figure S1. Active CDT1 is present together with fired origins in early S phase, related to Figure 1

(A-C) MCF10A cells expressing EYFP-PCNA and doxycycline (Dox)-inducible CDT1-mCherry (induced 6 h prior to imaging) were imaged using confocal microscopy.

(A) Quantification of PCNA foci detection relative to CDT1 degradation in cells from Figure 1B. The start of CDT1 degradation and first detectable PCNA foci ($T_{PCNA\ offset}$), the total time it takes for CDT1 to be degraded ($T_{CDT1\ deg}$), and the overlap duration when CDT1 and PCNA foci are simultaneously visible ($T_{CDT1\&PCNA}$) were determined. $T_{PCNA\ offset}$ represents the amount of time it takes for PCNA foci to grow large enough to be detectable over soluble pool of PCNA following the start of origin firing and CDT1 degradation. Black × corresponds to frames shown in example cell in Figure 1B.

(B) Left: $T_{PCNA\ offset}$, n = 54 cells. Right: $T_{CDT1\ deg}$ (n = 99 cells) and $T_{CDT1\&PCNA}$ (n = 54 cells). Cells pooled from 4 independent experiments.

(C) CDT1-mCherry signal in cells not entering S phase as photobleaching control. Note that imaged cells not entering S phase do not reduce CDT1-mCherry signal. Left: representative cell imaged over same interval as Figure 1B. Scale bar = 10 µm. Right: CDT1-mCherry signal in cells undergoing S phase entry (CDT1 deg.; n = 28 cells) and not undergoing S phase entry (no CDT1 deg.; n = 107 cells). Shaded area is mean ± 2×SEM.

(D) Mutational analysis of CDT1 Cy motif (necessary for degradation by SCF^{Skp2}) and PIP degron (necessary for degradation by $CRL4^{Cdt2}$) to identify E3 ubiquitin ligase primarily responsible for early S phase CDT1 degradation. Dox-inducible CDT1 mutants with Cy motif ($CDT1^{\Delta Cy}$) or PIP degron ($CDT1^{\Delta PIP}$) removed were analyzed in MCF10A cells. Double-mutant CDT1(ND-CDT1 from Figure 2 and Figure S3) is stable in S phase.

(E) CDT1 mutant degradation relative to S entry (PCNA foci appearance). (i-iii) Mean trace (bold) and 20 representative cell traces. (iv) Mean trace ± 2×SEM. n ≥ 192 cells. Representative of 3 independent experiments.

(F) QIBC of CDT1 immunofluorescence (detects endogenous and exogenous CDT1) of Dox-induced cells with mutant CDT1 compared to endogenous levels (uninduced cells). Dashed line indicates negative staining threshold. n = 5,000 cells.

(G) Live-cell reporters of $CRL4^{Cdt2}$ and APC/C^{Cdh1} activity. APC/C^{Cdh1} reporter is degraded throughout G1 and rises after APC/C^{Cdh1} inactivation. Example traces of N-$CRL4^{Cdt2}$ reporter and APC/C^{Cdh1} reporter in MCF10A cells are shown on right.

(H) $CRL4^{Cdt2}$ activation timing for N-$CRL4^{Cdt2}$ and C-$CRL4^{Cdt2}$ reporters relative to APC/C^{Cdh1} inactivation in cycling MCF10A cells. Lower values indicate earlier $CRL4^{Cdt2}$ activation relative to APC/C^{Cdh1} inactivation. n = 300 cells each condition, representative of 2 independent experiments. *** *p*-value = 1.3×10^{-4}, two-sample *t*-test. 95% confidence interval 2.6-8.0 min earlier.

(I) RT-QIBC of chromatin-bound PCNA after degradation of the C-$CRL4^{Cdt2}$ reporter starts. Left: Dashed line is PCNA threshold. Grey bar is unobserved period due to the need to have 12 min of reporter degradation to call S phase start. The small percentage of cells below the threshold (2.51% of 10,113 cells) had misidentified C-$CRL4^{Cdt2}$ degradation by automated algorithm, verified manually. Representative of 2 independent experiments. Right: Example traces and chromatin-bound PCNA stain in G1 (no C-$CRL4^{Cdt2}$ degradation) or just after S phase entry (C-$CRL4^{Cdt2}$ degradation). Scale bar = 5 µm.

Dashed and solid lines in violin plots are IQR and median, respectively.

Figure S2

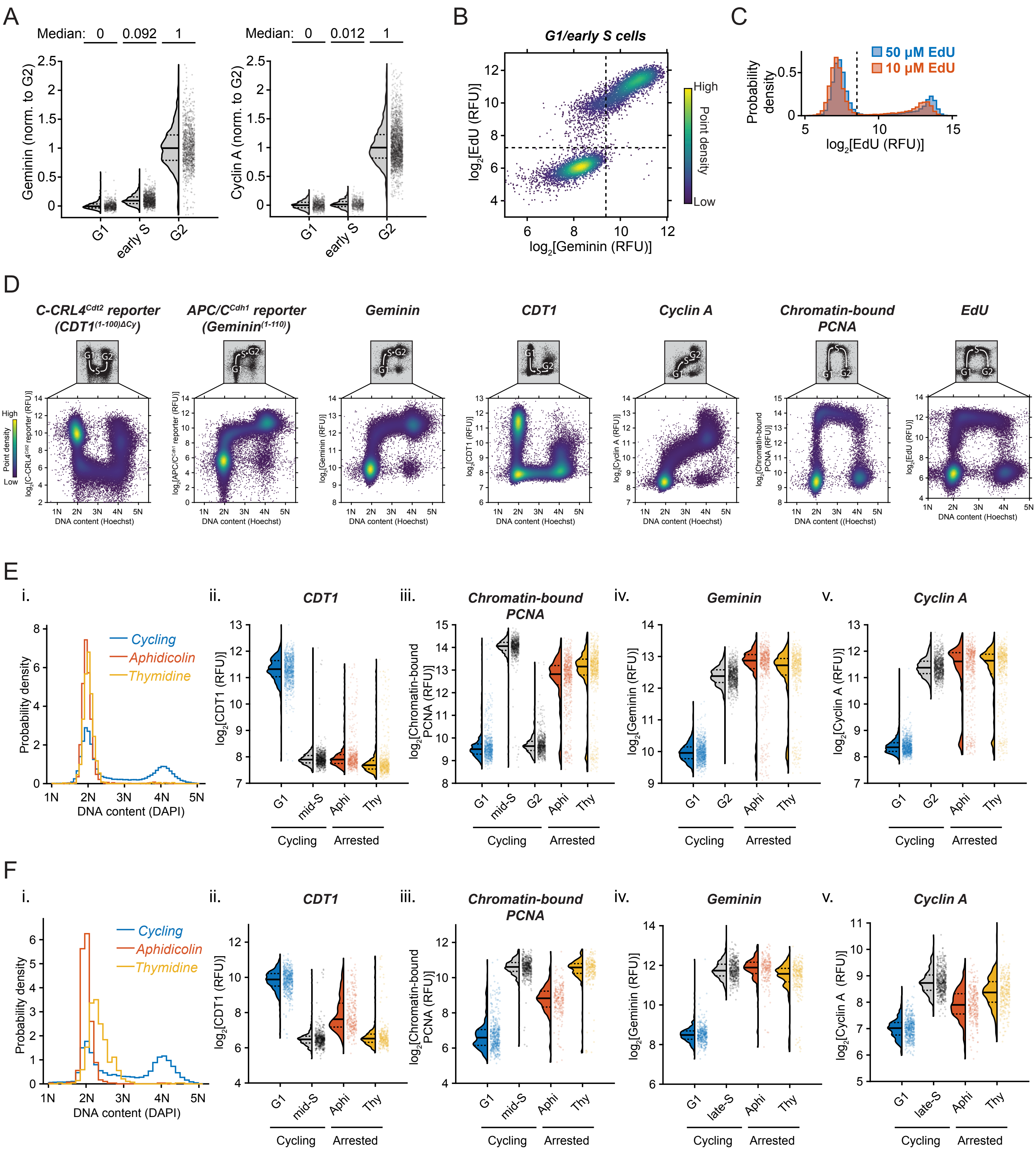
A
Median: 0 0.092 1
Geminin (norm. to G2)
Median: 0 0.012 1
Cyclin A (norm. to G2)
G1
early S
G2
B
G1/early S cells
log2[EdU (RFU)]
log2[Geminin (RFU)]
High
Point density
Low
C
50 μM EdU
10 μM EdU
Probability density
log2[EdU (RFU)]
D
C-CRL4Cdt2 reporter (CDT1(1-100)ΔCy)
APC/CCdh1 reporter (Geminin(1-110))
Geminin
CDT1
Cyclin A
Chromatin-bound PCNA
EdU
DNA content (Hoechst)
E
i.
Cycling
Aphidicolin
Thymidine
Probability density
DNA content (DAPI)
ii.
CDT1
log2[CDT1 (RFU)]
iii.
Chromatin-bound PCNA
log2[Chromatin-bound PCNA (RFU)]
iv.
Geminin
log2[Geminin (RFU)]
v.
Cyclin A
log2[Cyclin A (RFU)]
G1
mid-S
G2
Aphi
Thy
Cycling
Arrested
F
i.
Cycling
Aphidicolin
Thymidine
Probability density
DNA content (DAPI)
ii.
CDT1
log2[CDT1 (RFU)]
iii.
Chromatin-bound PCNA
log2[Chromatin-bound PCNA (RFU)]
iv.
Geminin
log2[Geminin (RFU)]
v.
Cyclin A
log2[Cyclin A (RFU)]
G1
mid-S
late-S
Aphi
Thy
Cycling
Arrested

Figure S2. Quantitative immunofluorescence analysis reveals protein dynamics during cell cycle, related to Figure 1

(A) Geminin and Cyclin A immunofluorescence (IF) levels from RT-QIBC analysis in Figures 1F-G. Representative of 3 independent experiments. Left: Comparison of Geminin levels in G1 (n = 600 cells), early S (first 30 min, n = 1,051 cells) and G2 (4N DNA and EdU(-), n = 1,063 cells) cells. Right: Comparison of Cyclin A levels in G1 (n = 699 cells), early S (first 30 min, n = 503 cells) and G2 (4N DNA and EdU(-), n = 2,637 cells).

(B) RT-QIBC in cycling MCF10A cells of endogenous Geminin and EdU incorporation in cells in G1 to early S (cells selected 3-7 h post mitosis, n = 9,605 cells, representative of 3 independent experiments). Lines demarcate Geminin and EdU positive/negative regions. Note large population of EdU positive, Geminin negative cells, indicating cells which entered S phase with low Geminin.

(C) Comparison of EdU staining intensity from 8 min pulse of either 10 µM or 50 µM EdU. 50 µM EdU was chosen for experiments due to slightly stronger signal. Dashed line is threshold for background EdU stain. n = 16,439 (50 µM), 15,571 (10 µM) cells.

(D) QIBC analysis of C-CRL4^{Cdt2} (mCherry-CDT1$^{(1-100)\Delta Cy}$) and APC/C^{Cdh1} (mVenus-Geminin$^{(1-110)}$) reporter fluorescence, EdU stain, and Geminin, CDT1, Cyclin and chromatin-bound PCNA IF intensity in MCF10A cells. Plotted against DNA content (Hoechst). Diagram above plots indicates inferred cell trajectory from G1 to S to G2. n ≥ 28,046 per condition. Representative of 3 independent experiments.

(E) QIBC analysis. (i) DNA content (DAPI stain) in cycling (no drug) or arrested cells. Quantitative IF analysis of CDT1 (ii), chromatin-bound PCNA (iii), Geminin (iv) and Cyclin A (v) levels in MCF10A cells arrested by 2 µg/mL aphidicolin or 2 mM thymidine for 16 h. Cells were initially live-imaged, and cells that received drugs 0 - 2 h after mitosis (in G1) were analyzed to exclude cells arrested in S or G2 phase. n ≥ 2,777 per condition. (ii-v) IF analysis comparing arrested cells against G1 (1-3 h after mitosis), mid-S (2-3 h after S entry, determined by C-CRL4^{Cdt2} reporter), or G2 (0-3 h after G2 entry, determined by C-CRL4 reporter rise at S/G2 transition) cells in cycling cell population. Note that arrested cells are more similar to S or G2 cells, with low CDT1, and high chromatin-bound PCNA, Geminin, and Cyclin A. n = 1,000 per condition. Data pooled from 4 replicate wells.

(F) Same as (E) performed in U2OS cells arrested by 2 µg/mL aphidicolin or 2 mM thymidine for 15 h. Cells were initially live-imaged, and cells that received drugs 0 - 2 h after mitosis (in G1) were analyzed to rule out cells arrested starting in S or G2 phase. (i) DNA content (DAPI stain) in cycling (no drug) or arrested cells. n ≥ 811 per condition. (ii-v) IF analysis comparing arrested cells against G1 (1-3 h after mitosis), mid-S (2-3 h after S entry, determined by N-CRL4^{Cdt2} reporter), or late-S (15-17 h after mitosis) cells in cycling cell population. n = 400 per condition. Data pooled from 4 replicate wells.

Dashed and solid lines in violin plots are IQR and median, respectively.

Figure S3

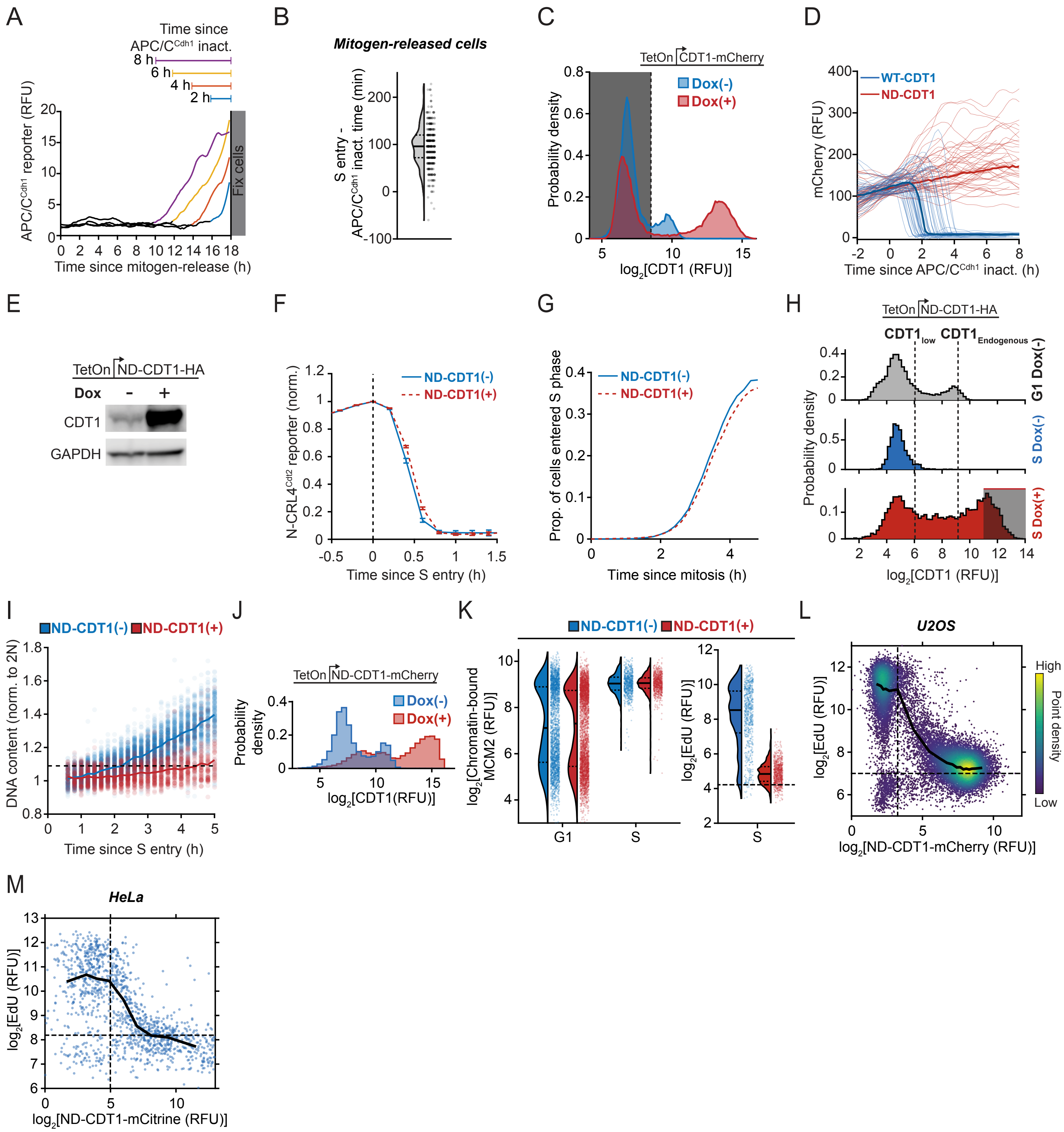

A
Time since APC/C^{Cdh1} inact.
8 h
6 h
4 h
2 h
Fix cells
APC/C^{Cdh1} reporter (RFU)
Time since mitogen-release (h)
B
Mitogen-released cells
S entry - APC/C^{Cdh1} inact. time (min)
C
TetOn CDT1-mCherry
Dox(-)
Dox(+)
Probability density
log$_2$[CDT1 (RFU)]
D
WT-CDT1
ND-CDT1
mCherry (RFU)
Time since APC/C^{Cdh1} inact. (h)
E
TetOn ND-CDT1-HA
Dox
CDT1
GAPDH
F
ND-CDT1(-)
ND-CDT1(+)
N-CRL4^{Cdt2} reporter (norm.)
Time since S entry (h)
G
ND-CDT1(-)
ND-CDT1(+)
Prop. of cells entered S phase
Time since mitosis (h)
H
TetOn ND-CDT1-HA
CDT1$_{low}$
CDT1$_{Endogenous}$
G1 Dox(-)
S Dox(-)
S Dox(+)
Probability density
log$_2$[CDT1 (RFU)]
I
ND-CDT1(-)
ND-CDT1(+)
DNA content (norm. to 2N)
Time since S entry (h)
J
TetOn ND-CDT1-mCherry
Dox(-)
Dox(+)
Probability density
log$_2$[CDT1(RFU)]
K
ND-CDT1(-)
ND-CDT1(+)
log$_2$[Chromatin-bound MCM2 (RFU)]
log$_2$[EdU (RFU)]
G1
S
L
U2OS
High
Low
Point density
log$_2$[EdU (RFU)]
log$_2$[ND-CDT1-mCherry (RFU)]
M
HeLa
log$_2$[EdU (RFU)]
log$_2$[ND-CDT1-mCitrine (RFU)]

Figure S3. DNA synthesis is inhibited in the presence of CDT1, related to Figure 2

(A) Example traces of cells from Figure 2B that inactivated APC/C^{Cdh1} 2, 4, 6, and 8 h prior to fixation.

(B) Time between APC/C^{Cdh1} inactivation and CRL4^{Cdt2} activation (N-CRL4^{Cdt2}) in mitogen-released cells (compare to cycling cells, Figure S1H). n = 400 cells, representative of 3 independent experiments.

(C) Cells from same experiment as Figure 2B. QIBC of CDT1 immunofluorescence (IF) staining (detecting endogenous CDT1 as well as CDT1-mCherry). Dox(+) was induced with doxycycline (Dox) for 24 h. Cells pooled from 5 wells in each condition (n ≥ 22,350 cells).

(D) CDT1-mCherry and ND-CDT1-mCherry as mitogen-released MCF10A cells enter S phase. n = 185 (CDT1) and 205 (ND-CDT1) cells. Traces were aligned to APC/C^{Cdh1} inactivation.

(E) Western blot of ND-CDT1-HA expression in MCF10A cells induced by Dox for 6 h as in Figure 2E. Antibody detects endogenous CDT1 and ND-CDT1. CDT1 MW: 60.4 kDa, ND-CDT1-HA MW: 60.9 kDa.

(F, G) N-CRL4^{Cdt2} reporter degradation with and without ND-CDT1 induction (in same experiment as Figure 2E). Cells were Dox-induced for 6 h during imaging.

(F) Mean N-CRL4^{Cdt2} reporter intensity following degradation start. Error bars are mean ± 2×SEM (ND-CDT1(-): n = 547 cells, ND-CDT1(+): n = 1,644 cells).

(G) Proportion of cells entering S phase (N-CRL4^{Cdt2} reporter degraded) following mitosis. ND-CDT1(-): n = 7,637 cells, ND-CDT1(+): n = 8,880 cells.

(H) ND-CDT1 expression relative to normal G1 expression levels of CDT1 measured by QIBC in mitogen-released MCF10A cells for Figures 2G, 3F, and 3G. Top: CDT1 in G1 cells without Dox (n = 15,544). Typical endogenous CDT1 at the end of G1 ($CDT1_{Endogenous}$) are median value of cells above the mode of CDT1 expression in CDT1 positive cells. Middle: S phase cells (0.5-1 h after CRL4^{Cdt2} activation) without Dox (n = 250), representing fully degraded CDT1 levels ($CDT1_{low}$). Bottom: S phase cells induced with Dox (n = 2,417 cells) in S phase. Shaded bar is gate used for ND-CDT1(+) cells for Figure 2G.

(I) DNA content measured by Hoechst stain in same cells as in Figure 2G, normalized to 2N DNA. Curve is median value. ND-CDT1(-): n = 5,500 cells, ND-CDT1(+): n = 2,000 cells.

(J) ND-CDT1-mCherry in Dox-inducible cell line. ND-CDT1-mCherry was induced with Dox for 18 h, and cells were fixed and stained using anti-CDT1 antibody (detects endogenous and exogenous CDT1). n = 66,817(Dox(-)), 64,545 (Dox(+)) cells.

(K) RT-QIBC of chromatin-bound MCM2 levels (measuring licensed origins) and EdU incorporation. ND-CDT1-mCherry was expressed in mitogen-released cells. G1 cells (no APC/C^{Cdh1} inactivation) and S phase cells (1-2 h post APC/C^{Cdh1} inactivation) were identified. n = 3,418 cells per condition. Representative of 2 independent experiments.

(L) QIBC of U2OS cells with ND-CDT1-mCherry induced by Dox for 6 h. Early S phase cells were identified as 2N DNA and positive for Geminin stain and EdU incorporation. Thresholds are negative stain threshold. Black line is median EdU value in bins of ND-CDT1. n = 19,866 pooled from 8 wells.

(M) ND-CDT1-mCitrine was transiently transfected into HeLa cells expressing the APC/C^{Cdh1} reporter. RT-QIBC was performed after live-imaging both ND-CDT1-mCitrine and APC/C^{Cdh1} reporter levels for 15 h. EdU incorporation in early S phase cells (2N DNA, APC/C^{Cdh1} reporter positive) was measured and plotted according to their ND-CDT1-mCitrine levels. n = 1,269 cells, pooled from 3 wells.

Dashed and solid lines in violin plots are IQR and median, respectively.

Figure S4

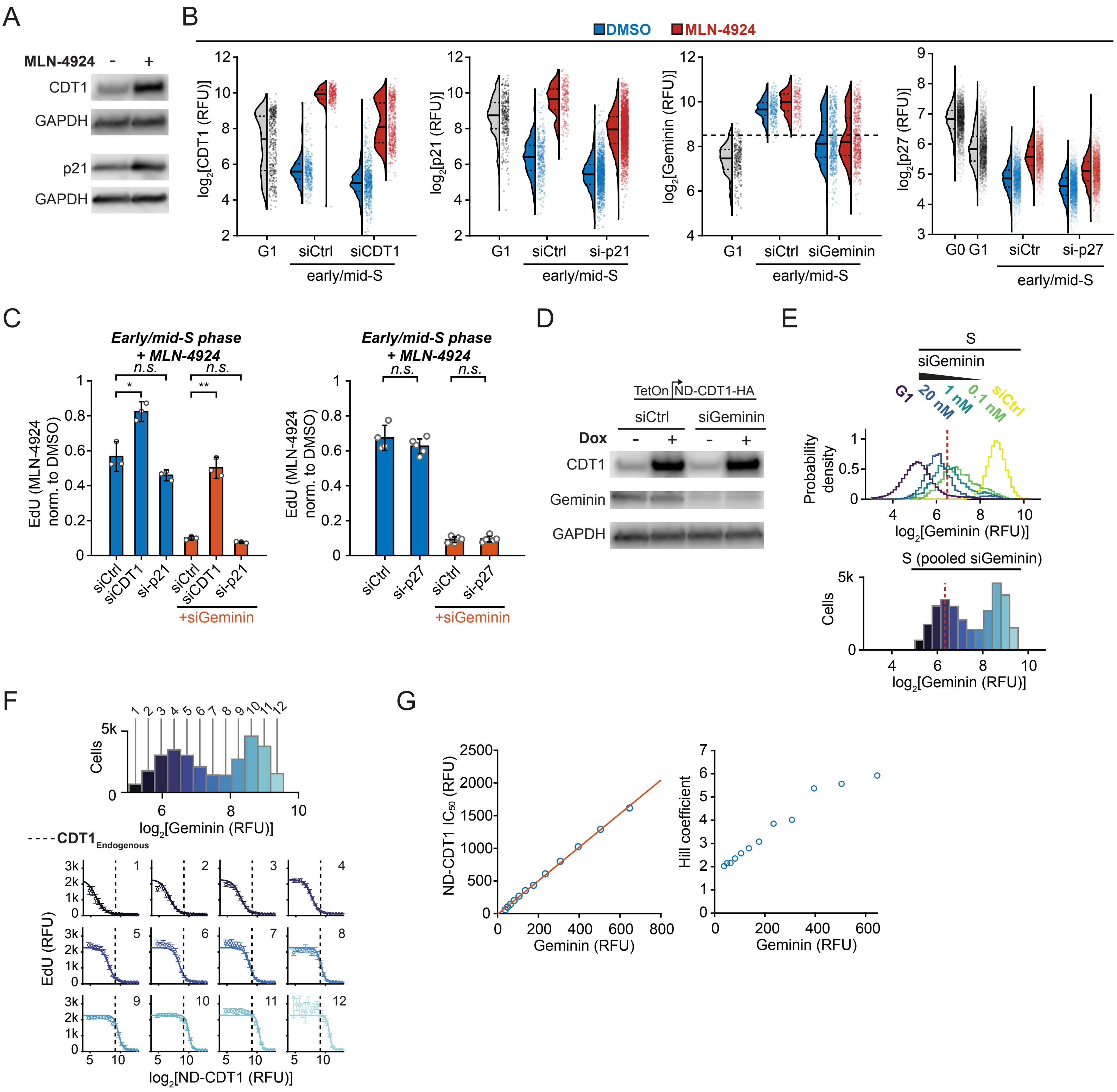
A
MLN-4924 - +
CDT1
GAPDH
p21
GAPDH
B
DMSO
MLN-4924
log2[CDT1 (RFU)]
G1
siCtrl
siCDT1
early/mid-S
log2[p21 (RFU)]
si-p21
log2[Geminin (RFU)]
siGeminin
log2[p27 (RFU)]
G0 G1
siCtr
si-p27
C
Early/mid-S phase + MLN-4924
EdU (MLN-4924 norm. to DMSO)
n.s.
siCtrl
siCDT1
si-p21
+siGeminin
si-p27
D
TetOn ND-CDT1-HA
siCtrl
siGeminin
Dox
CDT1
Geminin
GAPDH
E
S
siGeminin
G1
20 nM
1 nM
0.1 nM
siCtrl
Probability density
log2[Geminin (RFU)]
S (pooled siGeminin)
Cells
F
Cells
log2[Geminin (RFU)]
CDT1 Endogenous
EdU (RFU)
log2[ND-CDT1 (RFU)]
G
ND-CDT1 IC50 (RFU)
Geminin (RFU)
Hill coefficient

Figure S4. Endogenous CDT1 can inhibit DNA synthesis and is counteracted by Geminin, related to Figure 3

(A) MCF10A cells were treated with MLN-4924 for 4 h and analyzed using western blot.

(B-D) QIBC in cells expressing APC/C^{Cdh1} reporter and Cyclin E/A-CDK reporter (see Methods and Figure S5H) were mitogen-released. MLN-4924 was added 4 h prior to fixation. Gating for early/mid-S phase cells is based on APC/C^{Cdh1} reporter intensity in 2N-3N cells with ≥ 0.8 Cyclin E/A-CDK activity.

(B) Validation of changes in protein levels with MLN-4924 and siRNA knockdowns. G1 cells were negative for APC/C^{Cdh1} reporter and EdU incorporation, with intermediate Cyclin E/A-CDK activity (0.5 - 0.8). G0 cells (p27 panel) were serum-starved, unreleased cells (included as positive control for p27 levels since MCF10A cells contain low levels of p27 in serum). G0 and G1 cell groups were determined from control siRNA-treated cells. n ≥ 174 cells for all conditions. For Geminin plot, dashed line is threshold below which cells with siGeminin were considered fully knocked down in Figure S4C. Data pooled from 3 (CDT1, p21, Geminin) or 4 (p27) wells. Dashed and solid lines in violin plots are IQR and median, respectively.

(C) Impact of siRNA knockdown on EdU incorporation in the presence of MLN-4924 in early/mid-S phase. Points are median values of cells in replicate wells, and bars are mean ± 2×SEM of the medians. For siGeminin conditions, cells with low Geminin staining were selected. Left: n ≥ 57 cells per well (3 wells). Two-sample *t*-test: siCtrl – siCDT1(* *p*-value = 3.7×10^{-2}), siCtrl – si-p21 (n.s. *p*-value = 8.6×10^{-2}), siCtrl/siGeminin – siCDT1/siGeminin (** *p*-value = 4.5×10^{-3}), siCtrl/siGeminin – si-p21/ siGeminin (n.s. *p*-value = 8.0×10^{-2}). Right: n ≥ 73 cells per well (4 wells). Two-sample *t*-test: siCtrl – si-p27 (n.s. *p*-value = .24), siCtrl/siGeminin – si-p27/siGeminin (n.s. *p*-value = .78).

(D) Western blot validation of ND-CDT1 overexpression (CDT1 antibody detects endogenous CDT1 and ND-CDT1) and Geminin knockdown.

(E) Top: A range of Geminin levels in cells 2-3 h after S phase entry (N-CRL4^{Cdt2} reporter) were produced by titrating siGeminin (n ≥ 4,572 cells for all conditions) for experiments in Figures 3G-3H. Dashed line is threshold for low Geminin levels, determined from G1 cell Geminin levels. Representative of 3 independent experiments. Bottom: Pooled S phase cells from all siGeminin conditions to generate a range of Geminin expression.

(F) Top: Cells from Figure S4E for all siRNA conditions were pooled together and separated into 12 bins for analysis of the impact of Geminin on EdU incorporation. (n = 29,350 total cells). Bottom: Individual dose-response fits from Figure 3G. Dashed line represents endogenous CDT1 levels, determined from Figure S3H. Points and error bars are mean ± 2×SEM for bins of ND-CDT1 expression for given Geminin level (bins ≥ 6 cells, median bin count 127).

(G) ND-CDT1 IC_{50}(left) and Hill coefficient (right) for fit dose-response as a function of Geminin expression levels. Left: Line is linear regression fit (R^2 = .999).

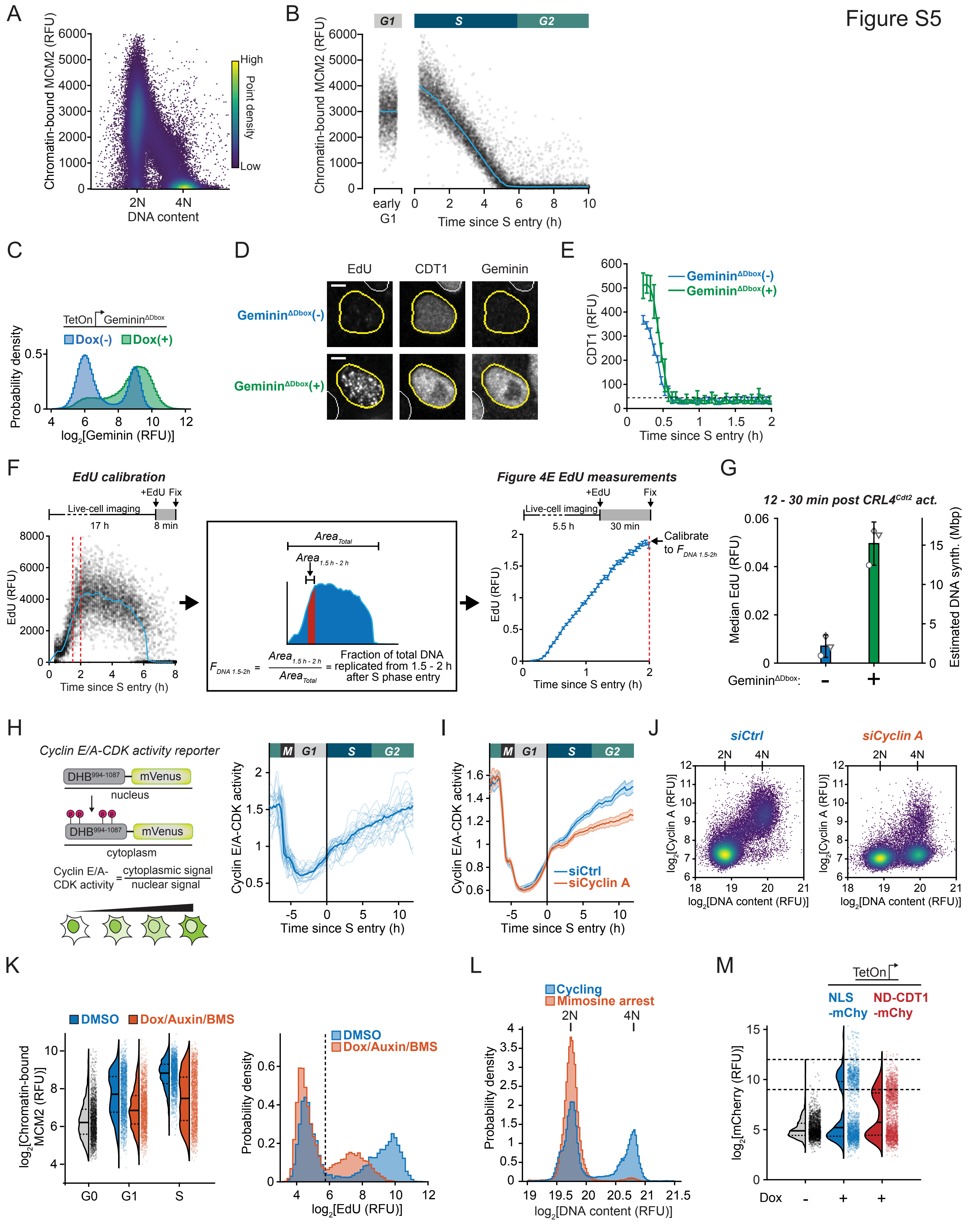
Figure S5
A
Chromatin-bound MCM2 (RFU)
DNA content
2N
4N
High
Point density
Low
B
G1
S
G2
early G1
Time since S entry (h)
C
TetOn Geminin^ΔDbox
Dox(-)
Dox(+)
Probability density
log2[Geminin (RFU)]
D
EdU
CDT1
Geminin
Geminin^ΔDbox(-)
Geminin^ΔDbox(+)
E
CDT1 (RFU)
Geminin^ΔDbox(-)
Geminin^ΔDbox(+)
Time since S entry (h)
F
EdU calibration
+EdU
Fix
Live-cell imaging
17 h
8 min
EdU (RFU)
Time since S entry (h)
Area Total
Area 1.5 h - 2 h
F DNA 1.5-2h = Area 1.5 h - 2 h / Area Total = Fraction of total DNA replicated from 1.5 - 2 h after S phase entry
Figure 4E EdU measurements
Live-cell imaging
5.5 h
30 min
Calibrate to F DNA 1.5-2h
EdU (RFU)
Time since S entry (h)
G
12 - 30 min post CRL4^Cdt2 act.
Median EdU (RFU)
Estimated DNA synth. (Mbp)
Geminin^ΔDbox:
-
+
H
Cyclin E/A-CDK activity reporter
DHB^994-1087
mVenus
nucleus
cytoplasm
Cyclin E/A-CDK activity = cytoplasmic signal / nuclear signal
Cyclin E/A-CDK activity
M
G1
S
G2
Time since S entry (h)
I
Cyclin E/A-CDK activity
siCtrl
siCyclin A
Time since S entry (h)
J
siCtrl
siCyclin A
2N
4N
log2[Cyclin A (RFU)]
log2[DNA content (RFU)]
K
DMSO
Dox/Auxin/BMS
log2[Chromatin-bound MCM2 (RFU)]
G0
G1
S
Probability density
log2[EdU (RFU)]
L
Cycling
Mimosine arrest
2N
4N
Probability density
log2[DNA content (RFU)]
M
TetOn
NLS -mChy
ND-CDT1 -mChy
log2[mCherry (RFU)]
Dox
-
+
+

Figure S5. CDT1 suppresses DNA synthesis during the overlap period of licensing and firing, independently of the global intra-S phase checkpoint and re-replication, related to Figure 4 and Figure 5

(A) QIBC of chromatin-bound MCM2 (n = 96,297 cells) in cycling MCF10A cells as a function of DNA content. 2N DNA content was estimated from G1 DNA intensity. Same experiment as Figure S5B.

(B) RT-QIBC of chromatin-bound MCM2, following S entry (C-CRL4^{Cdt2} reporter, n = 10,000 cells) and in early G1 (30 min – 1 h post anaphase, n = 1,500 cells). Pooled from 5 wells. Curves are median values.

(C) QIBC of Geminin$^{\Delta Dbox}$ in doxycycline (Dox)-inducible cell line used in Figures 4 and S5. Geminin$^{\Delta Dbox}$ was induced with Dox for 6 h and quantified with anti-Geminin antibody (detects endogenous Geminin and Geminin$^{\Delta Dbox}$). n = 147,420 (Dox(-)), 150,706 (Dox(+)) cells.

(D) Same example cells as shown in Figure 4F, co-stained for EdU incorporation, CDT1, and Geminin (detecting both endogenous Geminin and Geminin$^{\Delta Dbox}$). Scale bar = 5 µm. Note, CDT1 levels are likely increased in response to Geminin$^{\Delta Dbox}$ due to co-stabilization. However, CDT1 is inactivated by Geminin, and CDT1 is still degraded over the same 30 min period (Figure S5E).

(E) RT-QIBC aligned to S entry (C-CRL4^{Cdt2} reporter). Same experiment as in Figures 4E-F. Cells that had Dox added ≤ 1 h prior to mitosis were analyzed. Error bars and line are mean ± 2×SEM in bins of cells (Geminin$^{\Delta Dbox}$(-): n = 3,436, Geminin$^{\Delta Dbox}$(+): 2,302 cells total, n ≥ 32 cells per bin). Representative of 3 independent experiments.

(F, G) Calibration of EdU incorporation to absolute DNA synthesis from Figure 4E using RT-QIBC (C-CRL4^{Cdt2} reporter). Absolute DNA synthesis (in base pairs) can be inferred by integrating EdU incorporation measurements (area under the curve), which estimates the total DNA EdU signal. The fraction of total DNA synthesis during a given period (a period 1.5-2 h after S phase entry was chosen as a calibration point. Denoted as $F_{DNA\ 1.5\text{-}2h}$) can be estimated by taking the ratio of the area from 1.5 - 2 h after S phase entry ($Area_{1.5\text{-}2h}$) to the total area ($Area_{Total}$). In the experiment from Figure 4E, a 30 min EdU pulse was used, and thus the EdU intensity in cells 2 h after S phase entry in cells without Dox added would be equivalent to $F_{DNA\ 1.5\text{-}2h}$.

(F) Left: $F_{DNA\ 1.5\text{-}2h}$ was estimated by RT-QIBC of an 8 min EdU pulse at the end of imaging. Median EdU incorporation for each timepoint was used to determine area under the curve (n = 13,626 cells). Middle: Calculation of area under curve. Left: EdU incorporation from a 30 min EdU pulse at the end of imaging. Line and error bars are mean ± 2×SEM in cells within bins. Data pooled from 3 independent experiments (n = 33,208 cells).

(G) The EdU signal in Figure 4E was calibrated based on Figure S5F. Multiplying this by 6×10^9 base pairs (approximate human diploid DNA) gives the equivalent amount of DNA synthesis in base pairs. For each of 3 independent experiments, the median of cells was taken (n ≥ 31 cells per replicate per condition, Geminin$^{\Delta Dbox}$(-) 120 cells total, Geminin$^{\Delta Dbox}$(+) 213 cells total). Error bars are mean ± 2×SEM.

(H) Left: Cyclin E/A-CDK activity reporter is initially nuclear localized in G0 and early G1 and gradually translocates to the cytoplasm in response to Cyclin E/A-CDK activity. Right: 25 sample traces of Cyclin E/A-CDK activity in MCF10A cells aligned to S entry (N-CRL4^{Cdt2} reporter degradation). Thick line is median trace of 87 cells.

(I) MCF10A cells analyzed as in S5H with siRNA knockdown of Cyclin A (siCCNA2). CCNA1 is not expressed in these cells. Line and shaded area are mean ± 2×SEM. n = 129 (siCtrl) or 191 (siCyclin A) cells. Representative of 2 independent experiments.

(J) QIBC of Cyclin A immunofluorescence for validation of Cyclin A siRNA knockdown efficiency in MCF10A cells. n = 27,456 (siCtrl), 22,718 (siCyclin A). Representative of two independent experiments.

(K) RPE-1 *TP53*$^{-/-}$ *CDC6*$^{d/d}$ cells were serum-starved in G0. Cells were then mitogen-released in the presence of Dox, Auxin, and BMS-650032 (BMS) to degrade CDC6 as cells re-enter the cell cycle and inhibit origin licensing, or with vehicle (DMSO) to permit origin licensing. Left: Cells were fixed 12 h after mitogen-release, and QIBC was performed on chromatin-bound MCM2. G0 cells are unreleased cells, and G1 and early S phase cells were chosen based on EdU. n = 2,000 cells for all conditions. Pooled from 2 wells in each condition. Dashed and solid lines in violin plots are IQR and median, respectively. Right: Cells were fixed 15 h after mitogen-release, and QIBC was performed on EdU incorporation. 2N DNA (G1/early S phase cells) were plotted, and dashed line is threshold for EdU incorporation, calculated from G0 cells. n = 12,380 cells (DMSO), 13,543 cells (Dox/Auxin/BMS). Pooled from 2 wells.

(L) QIBC of DNA content in mimosine arrested RPE-1 cells using protocol in Figure 5F, compared to cycling cells. n = 52,433 cells (mimosine arrested) and 61,472 cells (cycling). Representative of 2 independent experiments.

(M) QIBC of mCherry (mChy) fluorescence in RPE-1 cells induced in Figure 5F. Dox(-) cells are uninduced TetOn-NLS-mChy cells to determine background signal. n = 2,000 cells per condition. Dashed and solid lines in violin plots are IQR and median, respectively. Cells with mCherry fluorescence within dashed lines were chosen for analysis in Figure 5H.

Figure S6

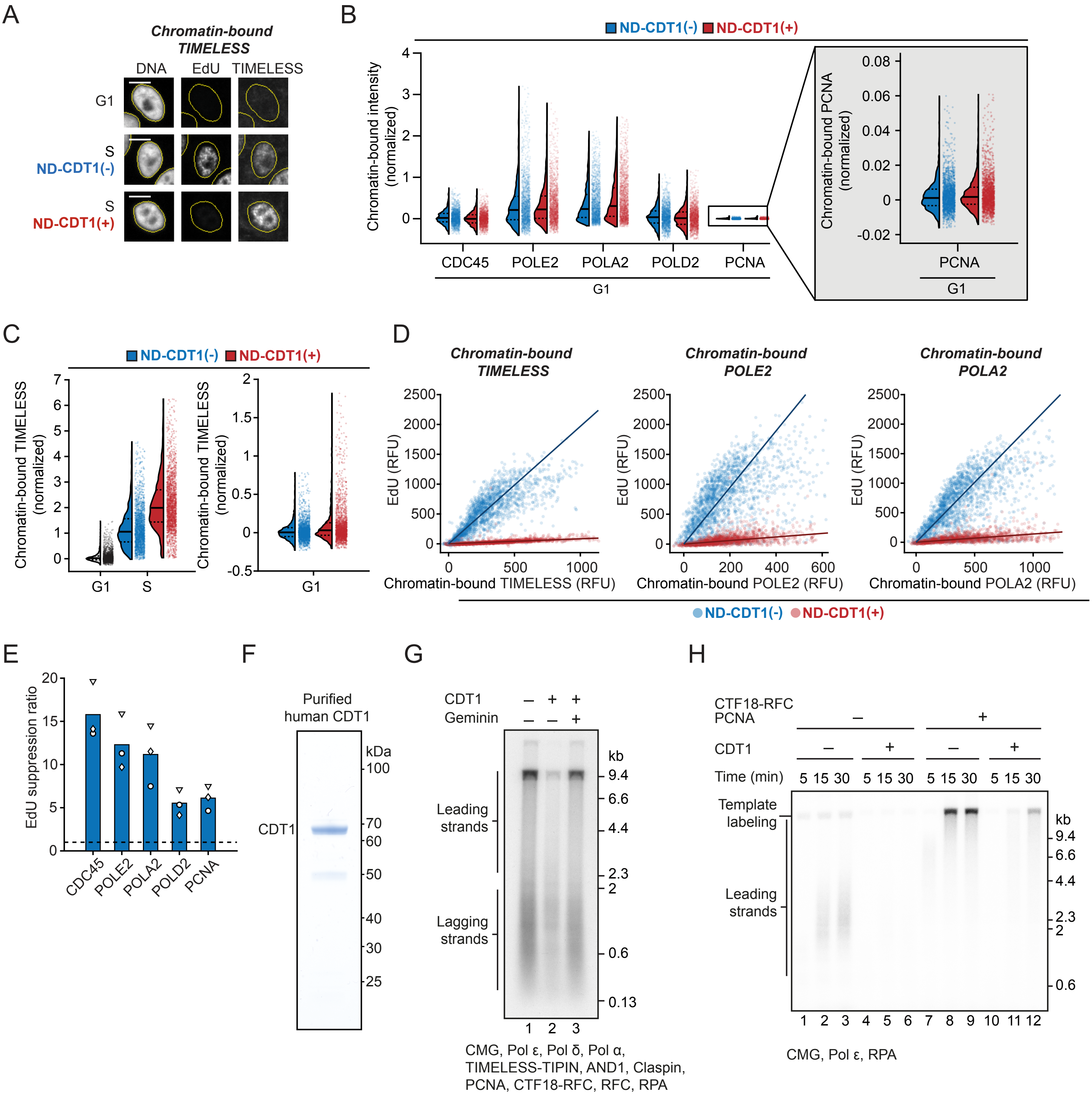
A
Chromatin-bound TIMELESS
DNA
EdU
TIMELESS
G1
S
ND-CDT1(-)
S
ND-CDT1(+)
B
ND-CDT1(-) ND-CDT1(+)
Chromatin-bound intensity (normalized)
CDC45
POLE2
POLA2
POLD2
PCNA
G1
Chromatin-bound PCNA (normalized)
PCNA
G1
C
ND-CDT1(-) ND-CDT1(+)
Chromatin-bound TIMELESS (normalized)
G1
S
Chromatin-bound TIMELESS (normalized)
G1
D
Chromatin-bound TIMELESS
Chromatin-bound POLE2
Chromatin-bound POLA2
EdU (RFU)
Chromatin-bound TIMELESS (RFU)
Chromatin-bound POLE2 (RFU)
Chromatin-bound POLA2 (RFU)
ND-CDT1(-) ND-CDT1(+)
E
EdU suppression ratio
CDC45
POLE2
POLA2
POLD2
PCNA
F
Purified human CDT1
kDa
CDT1
G
CDT1
Geminin
kb
Leading strands
Lagging strands
CMG, Pol ε, Pol δ, Pol α, TIMELESS-TIPIN, AND1, Claspin, PCNA, CTF18-RFC, RFC, RPA
H
CTF18-RFC
PCNA
CDT1
Time (min)
Template labeling
Leading strands
kb
CMG, Pol ε, RPA

Figure S6. CDT1 inhibits replication fork elongation while permitting origin firing, related to Figure 6

(A-E) RT-QIBC of chromatin-bound replisome components in mitogen-released MCF10A cells. G1 cells had active APC/C^{Cdh1} without chromatin-bound PCNA. Cells which expressed ND-CDT1-mCherry during live imaging were selected for ND-CDT1(+). Representative of 3 independent experiments.

(A) Representative cells of chromatin-bound TIMELESS. Scale bar = 10 µm.

(B) Comparison of chromatin-bound replisome components in G1 with ND-CDT1, in same experiment as Figure 6B. G1 mode intensities from ND-CDT1(-) were subtracted off signals, and values were normalized to the ND-CDT1(-) S phase condition from Figure 6B. n = 2,000 cells per condition. Representative of 3 individual experiments.

(C) Comparison of chromatin-bound TIMELESS in S phase (left) and G1 phase (right), analyzed in same way as Figure 6B and Figure S6B. n = 2,000 cells, pooled from 3 wells for ND-CDT1(-), or 7 wells for ND-CDT1(+).

(D) Analysis of EdU incorporation as a function of chromatin-bound protein levels for TIMELESS, POLE2, POLA2 in S phase. G1 mode intensities were subtracted off EdU and chromatin-bound intensity. Line is fit line of linear regression (n = 2000 cells). Representative of 3 independent experiments. Other stains in Figure 6D. TIMELESS staining was done in separate experiment as other stains. Cells were pooled from 3 wells for ND-CDT1(-) or 7 wells for ND-CDT1(+) from a single experiment.

(E) Summary of slopes from fit lines from Figures 6D and S6D. EdU suppression ratio is defined as the ratio of the fit line in the control condition to the ND-CDT1 condition (>1 indicates there is lower EdU incorporation for a given amount of chromatin-bound protein). Bar is mean of 3 independent experiments.

(F) Coomassie-stained SDS-PAGE analysis of purified human CDT1 protein.

(G) Denaturing agarose gel analysis of leading/lagging strand replication reactions (9.7 kbp DNA template) for 20 min with the indicated proteins. 10 nM CDT1 was pre-incubated with equimolar Geminin for 5 min on ice before adding to the reactions where indicated. In all reactions, the concentration of potassium glutamate was 250 mM. Experiment was repeated 3 times.

(H) Denaturing agarose gel analysis of a time course experiment performed on the 15.8 kbp forked DNA template with the indicated proteins. CTF18-RFC, PCNA, and CDT1 were included where indicated. CMG-independent template labeling products are indicated. Experiment was repeated two times.

Dashed and solid lines in violin plots are IQR and median, respectively.

Figure S7

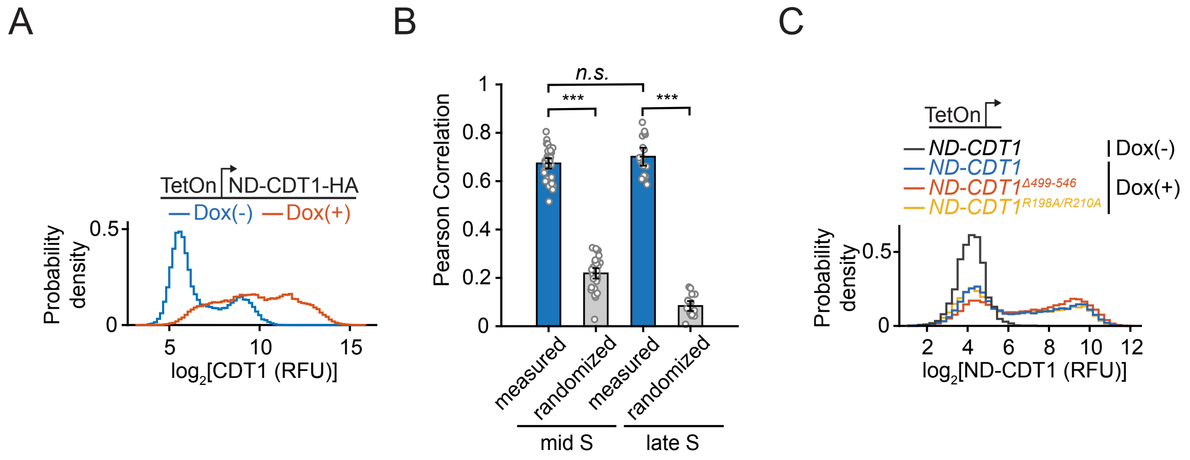
A
TetOn ND-CDT1-HA
Dox(-)
Dox(+)
Probability density
0.5
0
5
10
15
log2[CDT1 (RFU)]
B
n.s.
***
***
Pearson Correlation
1
0.8
0.6
0.4
0.2
0
measured
randomized
measured
randomized
mid S
late S
C
TetOn
ND-CDT1
ND-CDT1
ND-CDT1Δ499-546
ND-CDT1R198A/R210A
Dox(-)
Dox(+)
Probability density
0.5
0
2
4
6
8
10
12
log2[ND-CDT1 (RFU)]

Figure S7. CDT1 inhibits CMG helicase through its MCM-binding domains, related to Figure 7

(A) QIBC of CDT1 immunofluorescence in non-pre extracted U2OS cells overexpressing doxycycline (Dox)-inducible ND-CDT1 as in Figure 7D to compare overexpression levels. n = 56,309 (Dox-), 57,484 (Dox+) cells. Cells were pooled from 6 individual wells per condition.

(B) Quantification of colocalization of ND-CDT1 with chromatin-bound CDC45 from Figure 7D. U2OS cells with endogenously tagged CDC45 and Dox-inducible ND-CDT1 were treated with Dox for 8 h and then pre-extracted and co-stained for ND-CDT1(anti-HA-tag) and CDC45 (anti-GFP) by immunofluorescence. Cells were imaged using SoRa confocal microscopy and staged as either mid or late S phase based on CDC45 pattern. Colocalization of ND-CDT1 and CDC45 were calculated from the Pearson correlation between the two signals within the nucleus. Randomized data was generated from the mean of correlations from images shifted 40 pixels (1.038 µm) up, down, left, and right from each other. n= 35 (mid S) or 18 (late S) cells. Error bars are 2×SEM. Two-sample *t*-test: mid S measured – mid S randomized (*** *p*-value = 6.9×10^{-41}), late S measured – late S randomized (*** *p*-value = 1.1×10^{-25}), mid S measured – late S measured (n.s. *p*-value = .17).

(C) ND-CDT1 expression for ND-CDT1 mutants shown in Figure 7E. Mitogen-released MCF10A cells, treated with siGeminin and induced with Dox. ND-CDT1 was stained using anti-HA antibody. Note that ND-CDT1 expression levels are similar for all mutants. n ≥ 2146 cells for all conditions. Data representative of two independent experiments.
