# Supplementary material for: CDT1 inhibits CMG helicase in early S phase to separate origin licensing from DNA synthesis
Source: Mol Cell. Author manuscript; Available in PMC 2023 Jun 15. (PMC7614657; doi:10.1016/j.molcel.2022.12.004)
Supplement: Figures S1-S7 [file EMS176983-supplement-Figures_S1_S7.pdf]

Molecular Cell, Volume *83*

Supplemental information

CDT1 inhibits CMG helicase in early S phase

to separate origin licensing from DNA synthesis

Nalin Ratnayeke, Yasemin Baris, Mingyu Chung, Joseph T.P. Yeeles, and Tobias Meyer

Figure S1

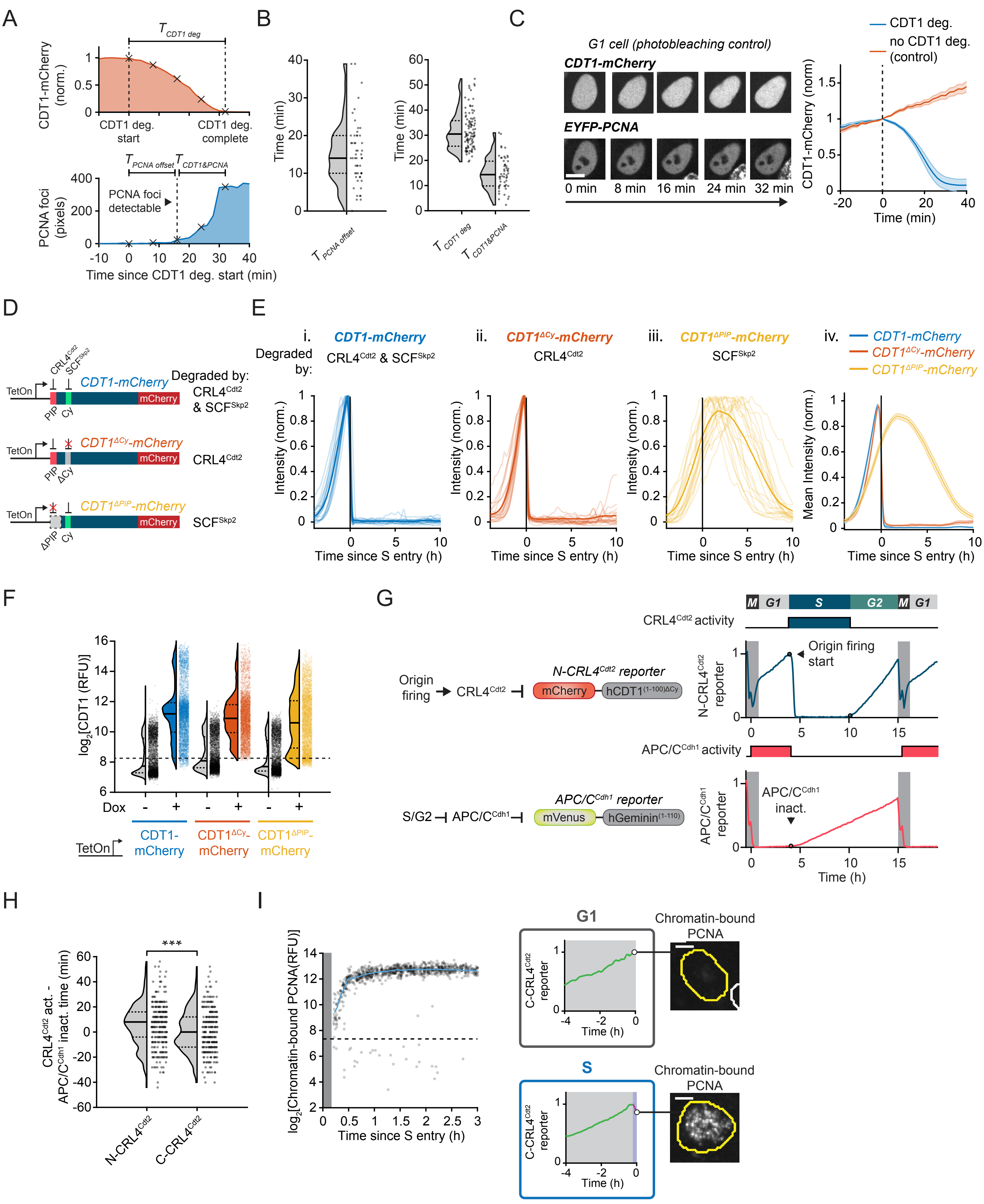

A
CDT1-mCherry (norm.)
CDT1 deg. start
CDT1 deg. complete
PCNA foci detectable
PCNA foci (pixels)
Time since CDT1 deg. start (min)
B
Time (min)
C
G1 cell (photobleaching control)
CDT1-mCherry
EYFP-PCNA
0 min
8 min
16 min
24 min
32 min
CDT1 deg.
no CDT1 deg. (control)
CDT1-mCherry (norm)
Time (min)
D
TetOn
CDT1-mCherry
Degraded by:
PIP
Cy
mCherry
E
i.
ii.
iii.
iv.
Degraded by:
Intensity (norm.)
Mean Intensity (norm.)
Time since S entry (h)
F
Dox
G
CRL4Cdt2 activity
Origin firing start
N-CRL4Cdt2 reporter
Origin firing
APC/CCdh1 activity
APC/CCdh1 inact.
APC/CCdh1 reporter
mCherry
mVenus
Time (h)
H
I
Time since S entry (h)
G1
S
Chromatin-bound PCNA
C-CRL4Cdt2 reporter
Time (h)

Figure S1. Active CDT1 is present together with fired origins in early S phase, related to Figure 1

(A-C) MCF10A cells expressing EYFP-PCNA and doxycycline (Dox)-inducible CDT1-mCherry (induced 6 h prior to imaging) were imaged using confocal microscopy.

(A) Quantification of PCNA foci detection relative to CDT1 degradation in cells from Figure 1B. The start of CDT1 degradation and first detectable PCNA foci ($T_{PCNA\ offset}$), the total time it takes for CDT1 to be degraded ($T_{CDT1\ deg}$), and the overlap duration when CDT1 and PCNA foci are simultaneously visible ($T_{CDT1\&PCNA}$) were determined. $T_{PCNA\ offset}$ represents the amount of time it takes for PCNA foci to grow large enough to be detectable over soluble pool of PCNA following the start of origin firing and CDT1 degradation. Black × corresponds to frames shown in example cell in Figure 1B.

(B) Left: $T_{PCNA\ offset}$, n = 54 cells. Right: $T_{CDT1\ deg}$ (n = 99 cells) and $T_{CDT1\&PCNA}$ (n = 54 cells). Cells pooled from 4 independent experiments.

(C) CDT1-mCherry signal in cells not entering S phase as photobleaching control. Note that imaged cells not entering S phase do not reduce CDT1-mCherry signal. Left: representative cell imaged over same interval as Figure 1B. Scale bar = 10 µm. Right: CDT1-mCherry signal in cells undergoing S phase entry (CDT1 deg.; n = 28 cells) and not undergoing S phase entry (no CDT1 deg.; n = 107 cells). Shaded area is mean ± 2×SEM.

(D) Mutational analysis of CDT1 Cy motif (necessary for degradation by SCF^{Skp2}) and PIP degron (necessary for degradation by $CRL4^{Cdt2}$) to identify E3 ubiquitin ligase primarily responsible for early S phase CDT1 degradation. Dox-inducible CDT1 mutants with Cy motif ($CDT1^{\Delta Cy}$) or PIP degron ($CDT1^{\Delta PIP}$) removed were analyzed in MCF10A cells. Double-mutant CDT1(ND-CDT1 from Figure 2 and Figure S3) is stable in S phase.

(E) CDT1 mutant degradation relative to S entry (PCNA foci appearance). (i-iii) Mean trace (bold) and 20 representative cell traces. (iv) Mean trace ± 2×SEM. n ≥ 192 cells. Representative of 3 independent experiments.

(F) QIBC of CDT1 immunofluorescence (detects endogenous and exogenous CDT1) of Dox-induced cells with mutant CDT1 compared to endogenous levels (uninduced cells). Dashed line indicates negative staining threshold. n = 5,000 cells.

(G) Live-cell reporters of $CRL4^{Cdt2}$ and APC/C^{Cdh1} activity. APC/C^{Cdh1} reporter is degraded throughout G1 and rises after APC/C^{Cdh1} inactivation. Example traces of N-$CRL4^{Cdt2}$ reporter and APC/C^{Cdh1} reporter in MCF10A cells are shown on right.

(H) $CRL4^{Cdt2}$ activation timing for N-$CRL4^{Cdt2}$ and C-$CRL4^{Cdt2}$ reporters relative to APC/C^{Cdh1} inactivation in cycling MCF10A cells. Lower values indicate earlier $CRL4^{Cdt2}$ activation relative to APC/C^{Cdh1} inactivation. n = 300 cells each condition, representative of 2 independent experiments. *** *p*-value = 1.3×10^{-4}, two-sample *t*-test. 95% confidence interval 2.6-8.0 min earlier.

(I) RT-QIBC of chromatin-bound PCNA after degradation of the C-$CRL4^{Cdt2}$ reporter starts. Left: Dashed line is PCNA threshold. Grey bar is unobserved period due to the need to have 12 min of reporter degradation to call S phase start. The small percentage of cells below the threshold (2.51% of 10,113 cells) had misidentified C-$CRL4^{Cdt2}$ degradation by automated algorithm, verified manually. Representative of 2 independent experiments. Right: Example traces and chromatin-bound PCNA stain in G1 (no C-$CRL4^{Cdt2}$ degradation) or just after S phase entry (C-$CRL4^{Cdt2}$ degradation). Scale bar = 5 µm.

Dashed and solid lines in violin plots are IQR and median, respectively.

Figure S2

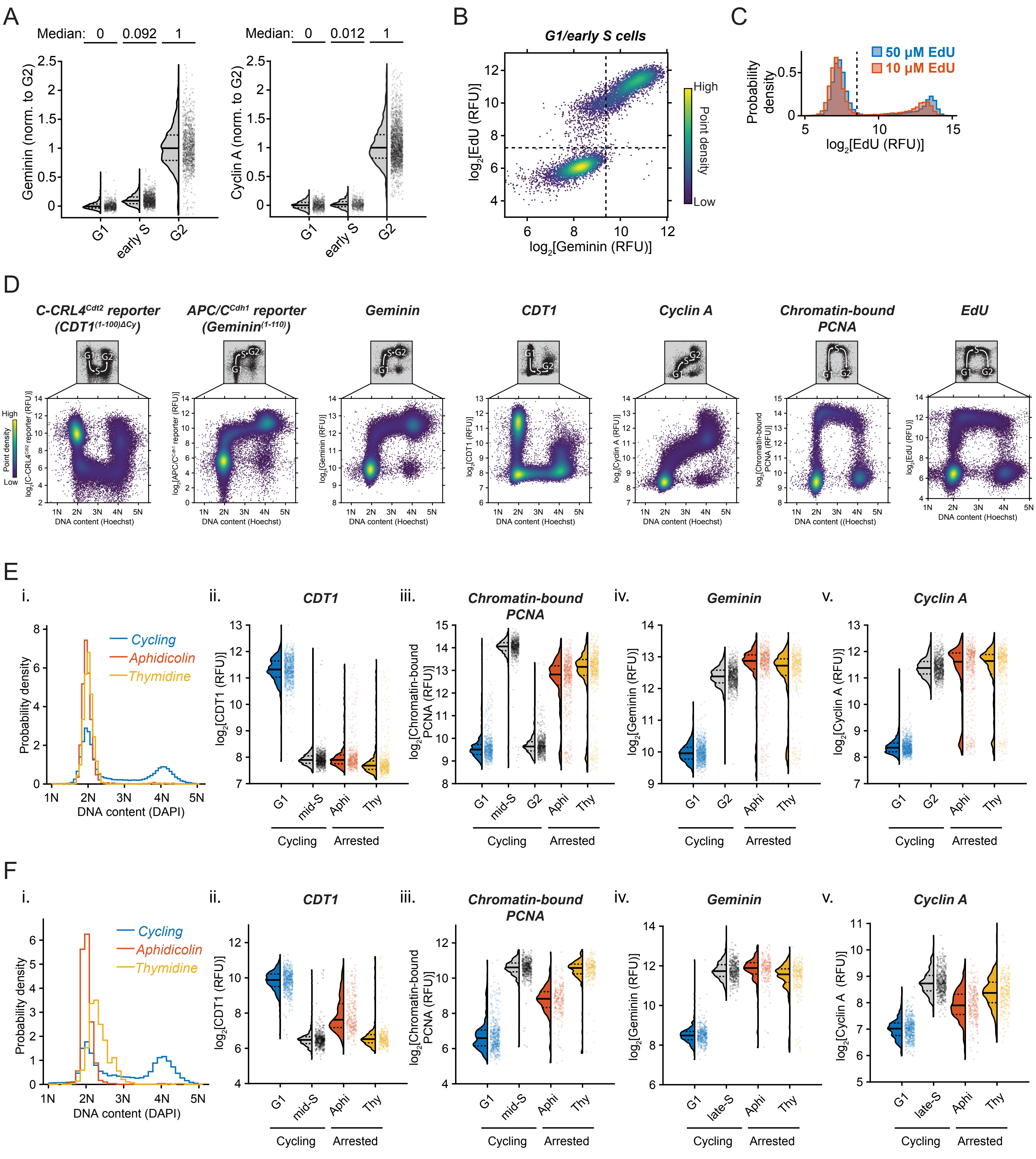

A
Median: 0 0.092 1
Geminin (norm. to G2)
Median: 0 0.012 1
Cyclin A (norm. to G2)
G1
early S
G2
B
G1/early S cells
log2[EdU (RFU)]
log2[Geminin (RFU)]
High
Point density
Low
C
50 μM EdU
10 μM EdU
Probability density
log2[EdU (RFU)]
D
C-CRL4Cdt2 reporter (CDT1(1-100)ΔCy)
APC/CCdh1 reporter (Geminin(1-110))
Geminin
CDT1
Cyclin A
Chromatin-bound PCNA
EdU
DNA content (Hoechst)
E
i.
Cycling
Aphidicolin
Thymidine
Probability density
DNA content (DAPI)
ii.
CDT1
log2[CDT1 (RFU)]
iii.
Chromatin-bound PCNA
log2[Chromatin-bound PCNA (RFU)]
iv.
Geminin
log2[Geminin (RFU)]
v.
Cyclin A
log2[Cyclin A (RFU)]
G1
mid-S
G2
Aphi
Thy
Cycling
Arrested
F
i.
Cycling
Aphidicolin
Thymidine
Probability density
DNA content (DAPI)
ii.
CDT1
log2[CDT1 (RFU)]
iii.
Chromatin-bound PCNA
log2[Chromatin-bound PCNA (RFU)]
iv.
Geminin
log2[Geminin (RFU)]
v.
Cyclin A
log2[Cyclin A (RFU)]
G1
mid-S
late-S
Aphi
Thy
Cycling
Arrested

Figure S2. Quantitative immunofluorescence analysis reveals protein dynamics during cell cycle, related to Figure 1

(A) Geminin and Cyclin A immunofluorescence (IF) levels from RT-QIBC analysis in Figures 1F-G. Representative of 3 independent experiments. Left: Comparison of Geminin levels in G1 (n = 600 cells), early S (first 30 min, n = 1,051 cells) and G2 (4N DNA and EdU(-), n = 1,063 cells) cells. Right: Comparison of Cyclin A levels in G1 (n = 699 cells), early S (first 30 min, n = 503 cells) and G2 (4N DNA and EdU(-), n = 2,637 cells).

(B) RT-QIBC in cycling MCF10A cells of endogenous Geminin and EdU incorporation in cells in G1 to early S (cells selected 3-7 h post mitosis, n = 9,605 cells, representative of 3 independent experiments). Lines demarcate Geminin and EdU positive/negative regions. Note large population of EdU positive, Geminin negative cells, indicating cells which entered S phase with low Geminin.

(C) Comparison of EdU staining intensity from 8 min pulse of either 10 μM or 50 μM EdU. 50 μM EdU was chosen for experiments due to slightly stronger signal. Dashed line is threshold for background EdU stain. n = 16,439 (50 μM), 15,571 (10 μM) cells.

(D) QIBC analysis of C-CRL4^{Cdt2} (mCherry-CDT1$^{(1\text{-}100)\Delta Cy}$) and APC/C^{Cdh1} (mVenus-Geminin$^{(1\text{-}110)}$) reporter fluorescence, EdU stain, and Geminin, CDT1, Cyclin and chromatin-bound PCNA IF intensity in MCF10A cells. Plotted against DNA content (Hoechst). Diagram above plots indicates inferred cell trajectory from G1 to S to G2. n ≥ 28,046 per condition. Representative of 3 independent experiments.

(E) QIBC analysis. (i) DNA content (DAPI stain) in cycling (no drug) or arrested cells. Quantitative IF analysis of CDT1 (ii), chromatin-bound PCNA (iii), Geminin (iv) and Cyclin A (v) levels in MCF10A cells arrested by 2 μg/mL aphidicolin or 2 mM thymidine for 16 h. Cells were initially live-imaged, and cells that received drugs 0 - 2 h after mitosis (in G1) were analyzed to exclude cells arrested in S or G2 phase. n ≥ 2,777 per condition. (ii-v) IF analysis comparing arrested cells against G1 (1-3 h after mitosis), mid-S (2-3 h after S entry, determined by C-CRL4^{Cdt2} reporter), or G2 (0-3 h after G2 entry, determined by C-CRL4 reporter rise at S/G2 transition) cells in cycling cell population. Note that arrested cells are more similar to S or G2 cells, with low CDT1, and high chromatin-bound PCNA, Geminin, and Cyclin A. n = 1,000 per condition. Data pooled from 4 replicate wells.

(F) Same as (E) performed in U2OS cells arrested by 2 μg/mL aphidicolin or 2 mM thymidine for 15 h. Cells were initially live-imaged, and cells that received drugs 0 - 2 h after mitosis (in G1) were analyzed to rule out cells arrested starting in S or G2 phase. (i) DNA content (DAPI stain) in cycling (no drug) or arrested cells. n ≥ 811 per condition. (ii-v) IF analysis comparing arrested cells against G1 (1-3 h after mitosis), mid-S (2-3 h after S entry, determined by N-CRL4^{Cdt2} reporter), or late-S (15-17 h after mitosis) cells in cycling cell population. n = 400 per condition. Data pooled from 4 replicate wells.

Dashed and solid lines in violin plots are IQR and median, respectively.

Figure S3

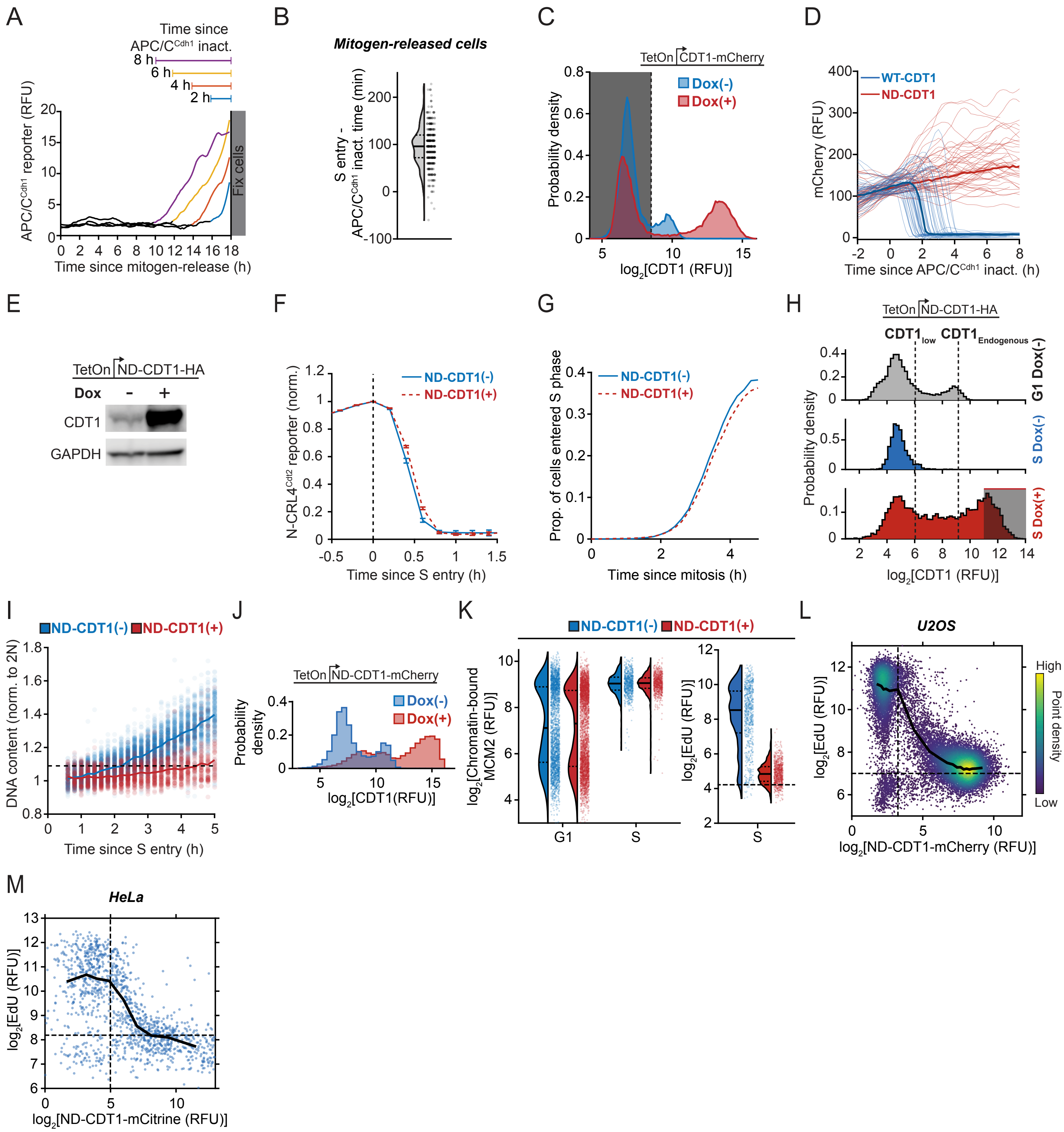

A
Time since APC/C^{Cdh1} inact.
8 h
6 h
4 h
2 h
Fix cells
APC/C^{Cdh1} reporter (RFU)
Time since mitogen-release (h)
B
Mitogen-released cells
S entry - APC/C^{Cdh1} inact. time (min)
C
TetOn CDT1-mCherry
Dox(-)
Dox(+)
Probability density
log$_2$[CDT1 (RFU)]
D
WT-CDT1
ND-CDT1
mCherry (RFU)
Time since APC/C^{Cdh1} inact. (h)
E
TetOn ND-CDT1-HA
Dox
-
+
CDT1
GAPDH
F
ND-CDT1(-)
ND-CDT1(+)
N-CRL4^{Cdt2} reporter (norm.)
Time since S entry (h)
G
ND-CDT1(-)
ND-CDT1(+)
Prop. of cells entered S phase
Time since mitosis (h)
H
TetOn ND-CDT1-HA
CDT1$_{low}$
CDT1$_{Endogenous}$
G1 Dox(-)
S Dox(-)
S Dox(+)
Probability density
log$_2$[CDT1 (RFU)]
I
ND-CDT1(-)
ND-CDT1(+)
DNA content (norm. to 2N)
Time since S entry (h)
J
TetOn ND-CDT1-mCherry
Dox(-)
Dox(+)
Probability density
log$_2$[CDT1(RFU)]
K
ND-CDT1(-)
ND-CDT1(+)
log$_2$[Chromatin-bound MCM2 (RFU)]
log$_2$[EdU (RFU)]
G1
S
S
L
U2OS
High
Low
Point density
log$_2$[EdU (RFU)]
log$_2$[ND-CDT1-mCherry (RFU)]
M
HeLa
log$_2$[EdU (RFU)]
log$_2$[ND-CDT1-mCitrine (RFU)]

Figure S3. DNA synthesis is inhibited in the presence of CDT1, related to Figure 2

(A) Example traces of cells from Figure 2B that inactivated APC/C^{Cdh1} 2, 4, 6, and 8 h prior to fixation.

(B) Time between APC/C^{Cdh1} inactivation and $CRL4^{Cdt2}$ activation (N-$CRL4^{Cdt2}$) in mitogen-released cells (compare to cycling cells, Figure S1H). n = 400 cells, representative of 3 independent experiments.

(C) Cells from same experiment as Figure 2B. QIBC of CDT1 immunofluorescence (IF) staining (detecting endogenous CDT1 as well as CDT1-mCherry). Dox(+) was induced with doxycycline (Dox) for 24 h. Cells pooled from 5 wells in each condition (n ≥ 22,350 cells).

(D) CDT1-mCherry and ND-CDT1-mCherry as mitogen-released MCF10A cells enter S phase. n = 185 (CDT1) and 205 (ND-CDT1) cells. Traces were aligned to APC/C^{Cdh1} inactivation.

(E) Western blot of ND-CDT1-HA expression in MCF10A cells induced by Dox for 6 h as in Figure 2E. Antibody detects endogenous CDT1 and ND-CDT1. CDT1 MW: 60.4 kDa, ND-CDT1-HA MW: 60.9 kDa.

(F, G) N-$CRL4^{Cdt2}$ reporter degradation with and without ND-CDT1 induction (in same experiment as Figure 2E). Cells were Dox-induced for 6 h during imaging.

(F) Mean N-$CRL4^{Cdt2}$ reporter intensity following degradation start. Error bars are mean ± 2×SEM (ND-CDT1(-): n = 547 cells, ND-CDT1(+): n = 1,644 cells).

(G) Proportion of cells entering S phase (N-$CRL4^{Cdt2}$ reporter degraded) following mitosis. ND-CDT1(-): n = 7,637 cells, ND-CDT1(+): n = 8,880 cells.

(H) ND-CDT1 expression relative to normal G1 expression levels of CDT1 measured by QIBC in mitogen-released MCF10A cells for Figures 2G, 3F, and 3G. Top: CDT1 in G1 cells without Dox (n = 15,544). Typical endogenous CDT1 at the end of G1 ($CDT1_{Endogenous}$) are median value of cells above the mode of CDT1 expression in CDT1 positive cells. Middle: S phase cells (0.5-1 h after $CRL4^{Cdt2}$ activation) without Dox (n = 250), representing fully degraded CDT1 levels ($CDT1_{low}$). Bottom: S phase cells induced with Dox (n = 2,417 cells) in S phase. Shaded bar is gate used for ND-CDT1(+) cells for Figure 2G.

(I) DNA content measured by Hoechst stain in same cells as in Figure 2G, normalized to 2N DNA. Curve is median value. ND-CDT1(-): n = 5,500 cells, ND-CDT1(+): n = 2,000 cells.

(J) ND-CDT1-mCherry in Dox-inducible cell line. ND-CDT1-mCherry was induced with Dox for 18 h, and cells were fixed and stained using anti-CDT1 antibody (detects endogenous and exogenous CDT1). n = 66,817(Dox(-)), 64,545 (Dox(+)) cells.

(K) RT-QIBC of chromatin-bound MCM2 levels (measuring licensed origins) and EdU incorporation. ND-CDT1-mCherry was expressed in mitogen-released cells. G1 cells (no APC/C^{Cdh1} inactivation) and S phase cells (1-2 h post APC/C^{Cdh1} inactivation) were identified. n = 3,418 cells per condition. Representative of 2 independent experiments.

(L) QIBC of U2OS cells with ND-CDT1-mCherry induced by Dox for 6 h. Early S phase cells were identified as 2N DNA and positive for Geminin stain and EdU incorporation. Thresholds are negative stain threshold. Black line is median EdU value in bins of ND-CDT1. n = 19,866 pooled from 8 wells.

(M) ND-CDT1-mCitrine was transiently transfected into HeLa cells expressing the APC/C^{Cdh1} reporter. RT-QIBC was performed after live-imaging both ND-CDT1-mCitrine and APC/C^{Cdh1} reporter levels for 15 h. EdU incorporation in early S phase cells (2N DNA, APC/C^{Cdh1} reporter positive) was measured and plotted according to their ND-CDT1-mCitrine levels. n = 1,269 cells, pooled from 3 wells.

Dashed and solid lines in violin plots are IQR and median, respectively.

Figure S4

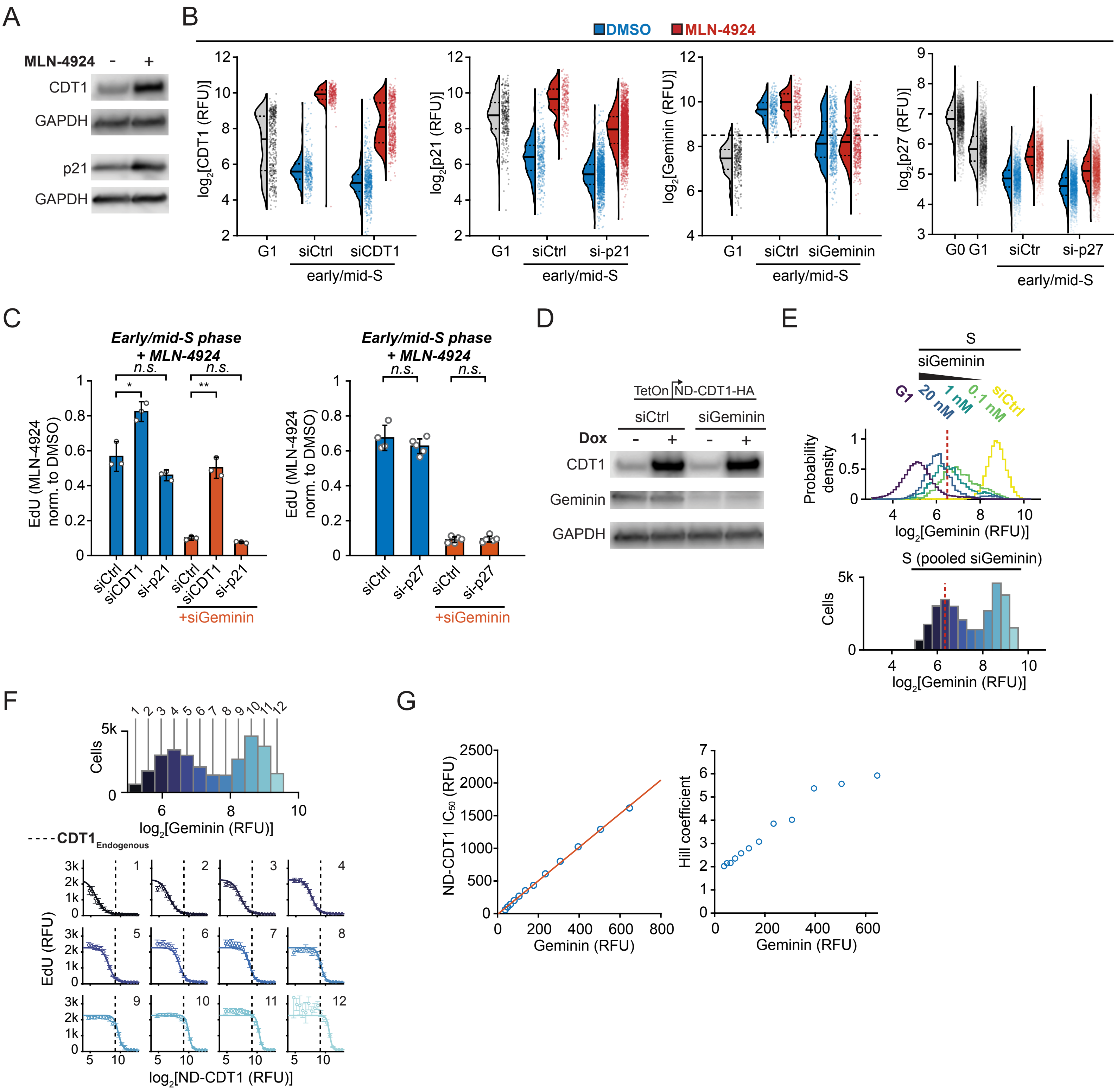

A
MLN-4924 - +
CDT1
GAPDH
p21
GAPDH
B
DMSO
MLN-4924
log2[CDT1 (RFU)]
G1
siCtrl
siCDT1
early/mid-S
log2[p21 (RFU)]
si-p21
log2[Geminin (RFU)]
siGeminin
log2[p27 (RFU)]
G0 G1
siCtr
si-p27
C
Early/mid-S phase + MLN-4924
EdU (MLN-4924 norm. to DMSO)
n.s.
siCtrl
siCDT1
si-p21
+siGeminin
si-p27
D
TetOn ND-CDT1-HA
siCtrl
siGeminin
Dox
CDT1
Geminin
GAPDH
E
S
siGeminin
G1
20 nM
1 nM
0.1 nM
siCtrl
Probability density
log2[Geminin (RFU)]
S (pooled siGeminin)
Cells
F
Cells
log2[Geminin (RFU)]
CDT1 Endogenous
EdU (RFU)
log2[ND-CDT1 (RFU)]
G
ND-CDT1 IC50 (RFU)
Geminin (RFU)
Hill coefficient

Figure S4. Endogenous CDT1 can inhibit DNA synthesis and is counteracted by Geminin, related to Figure 3

(A) MCF10A cells were treated with MLN-4924 for 4 h and analyzed using western blot.

(B-D) QIBC in cells expressing APC/C^{Cdh1} reporter and Cyclin E/A-CDK reporter (see Methods and Figure S5H) were mitogen-released. MLN-4924 was added 4 h prior to fixation. Gating for early/mid-S phase cells is based on APC/C^{Cdh1} reporter intensity in 2N-3N cells with ≥ 0.8 Cyclin E/A-CDK activity.

(B) Validation of changes in protein levels with MLN-4924 and siRNA knockdowns. G1 cells were negative for APC/C^{Cdh1} reporter and EdU incorporation, with intermediate Cyclin E/A-CDK activity (0.5 - 0.8). G0 cells (p27 panel) were serum-starved, unreleased cells (included as positive control for p27 levels since MCF10A cells contain low levels of p27 in serum). G0 and G1 cell groups were determined from control siRNA-treated cells. n ≥ 174 cells for all conditions. For Geminin plot, dashed line is threshold below which cells with siGeminin were considered fully knocked down in Figure S4C. Data pooled from 3 (CDT1, p21, Geminin) or 4 (p27) wells. Dashed and solid lines in violin plots are IQR and median, respectively.

(C) Impact of siRNA knockdown on EdU incorporation in the presence of MLN-4924 in early/mid-S phase. Points are median values of cells in replicate wells, and bars are mean ± 2×SEM of the medians. For siGeminin conditions, cells with low Geminin staining were selected. Left: n ≥ 57 cells per well (3 wells). Two-sample *t*-test: siCtrl – siCDT1(* *p*-value = 3.7×10^{-2}), siCtrl – si-p21 (n.s. *p*-value = 8.6×10^{-2}), siCtrl/siGeminin – siCDT1/siGeminin (** *p*-value = 4.5×10^{-3}), siCtrl/siGeminin – si-p21/ siGeminin (n.s. *p*-value = 8.0×10^{-2}). Right: n ≥ 73 cells per well (4 wells). Two-sample *t*-test: siCtrl – si-p27 (n.s. *p*-value = .24), siCtrl/siGeminin – si-p27/siGeminin (n.s. *p*-value = .78).

(D) Western blot validation of ND-CDT1 overexpression (CDT1 antibody detects endogenous CDT1 and ND-CDT1) and Geminin knockdown.

(E) Top: A range of Geminin levels in cells 2-3 h after S phase entry (N-CRL4^{Cdt2} reporter) were produced by titrating siGeminin (n ≥ 4,572 cells for all conditions) for experiments in Figures 3G-3H. Dashed line is threshold for low Geminin levels, determined from G1 cell Geminin levels. Representative of 3 independent experiments. Bottom: Pooled S phase cells from all siGeminin conditions to generate a range of Geminin expression.

(F) Top: Cells from Figure S4E for all siRNA conditions were pooled together and separated into 12 bins for analysis of the impact of Geminin on EdU incorporation. (n = 29,350 total cells). Bottom: Individual dose-response fits from Figure 3G. Dashed line represents endogenous CDT1 levels, determined from Figure S3H. Points and error bars are mean ± 2×SEM for bins of ND-CDT1 expression for given Geminin level (bins ≥ 6 cells, median bin count 127).

(G) ND-CDT1 IC_{50}(left) and Hill coefficient (right) for fit dose-response as a function of Geminin expression levels. Left: Line is linear regression fit (R^2 = .999).

Figure S5

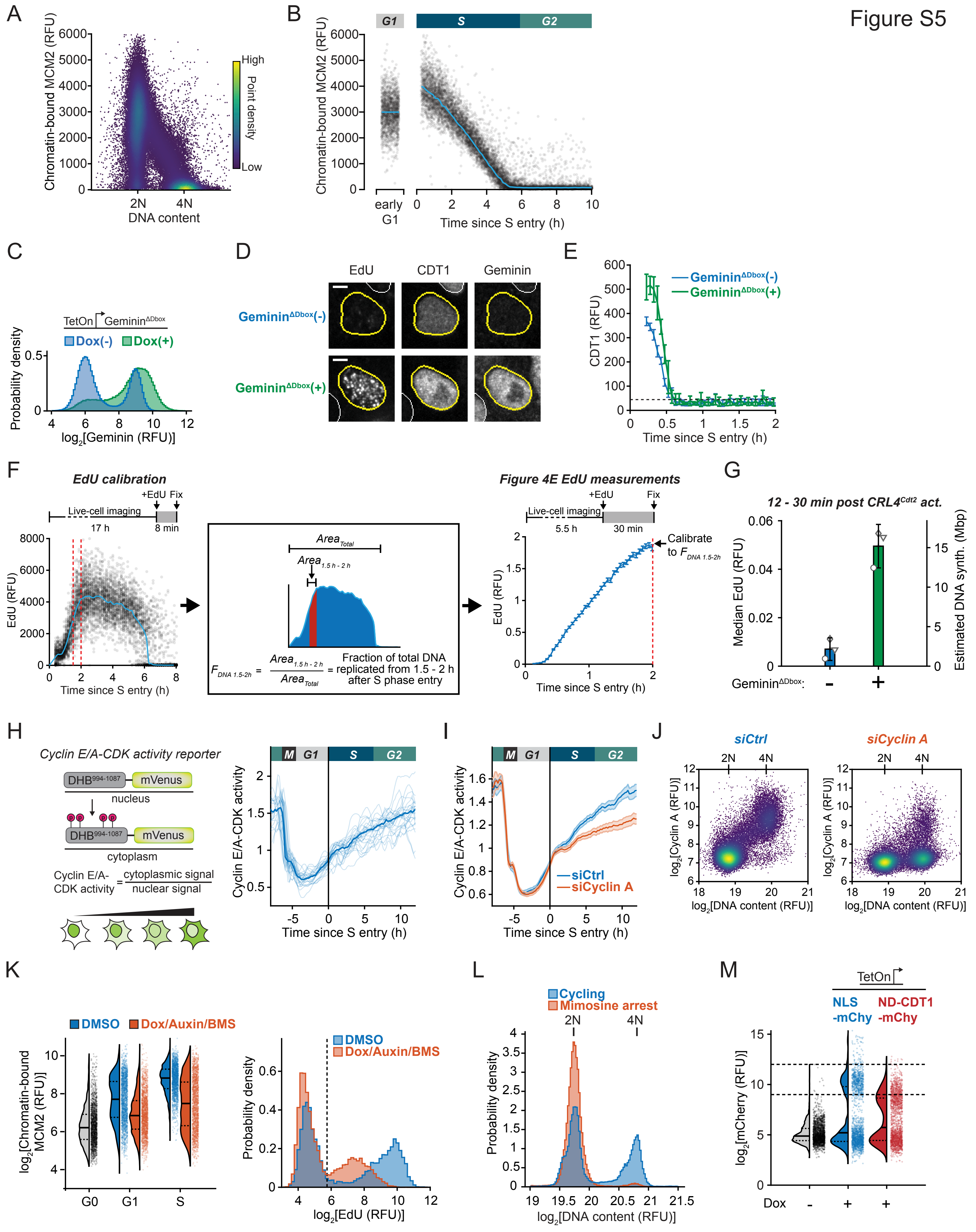
A
Chromatin-bound MCM2 (RFU)
DNA content
2N
4N
High
Point density
Low
B
G1
S
G2
early G1
Time since S entry (h)
C
TetOn Geminin^ΔDbox
Dox(-)
Dox(+)
Probability density
log2[Geminin (RFU)]
D
EdU
CDT1
Geminin
Geminin^ΔDbox(-)
Geminin^ΔDbox(+)
E
Geminin^ΔDbox(-)
Geminin^ΔDbox(+)
CDT1 (RFU)
Time since S entry (h)
F
EdU calibration
+EdU
Fix
Live-cell imaging
17 h
8 min
EdU (RFU)
Time since S entry (h)
Area_Total
Area_1.5 h - 2 h
F_DNA 1.5-2h = Area_1.5 h - 2 h / Area_Total = Fraction of total DNA replicated from 1.5 - 2 h after S phase entry
Figure 4E EdU measurements
5.5 h
30 min
Calibrate to F_DNA 1.5-2h
G
12 - 30 min post CRL4^Cdt2 act.
Median EdU (RFU)
Estimated DNA synth. (Mbp)
Geminin^ΔDbox:
H
Cyclin E/A-CDK activity reporter
DHB^994-1087
mVenus
nucleus
cytoplasm
Cyclin E/A-CDK activity = cytoplasmic signal / nuclear signal
M
Cyclin E/A-CDK activity
Time since S entry (h)
I
siCtrl
siCyclin A
J
siCtrl
siCyclin A
log2[Cyclin A (RFU)]
log2[DNA content (RFU)]
K
DMSO
Dox/Auxin/BMS
log2[Chromatin-bound MCM2 (RFU)]
G0
G1
S
Probability density
log2[EdU (RFU)]
L
Cycling
Mimosine arrest
Probability density
log2[DNA content (RFU)]
M
TetOn
NLS -mChy
ND-CDT1 -mChy
log2[mCherry (RFU)]
Dox

Figure S5. CDT1 suppresses DNA synthesis during the overlap period of licensing and firing, independently of the global intra-S phase checkpoint and re-replication, related to Figure 4 and Figure 5

(A) QIBC of chromatin-bound MCM2 (n = 96,297 cells) in cycling MCF10A cells as a function of DNA content. 2N DNA content was estimated from G1 DNA intensity. Same experiment as Figure S5B.

(B) RT-QIBC of chromatin-bound MCM2, following S entry (C-CRL4^{Cdt2} reporter, n = 10,000 cells) and in early G1 (30 min – 1 h post anaphase, n = 1,500 cells). Pooled from 5 wells. Curves are median values.

(C) QIBC of Geminin$^{\Delta Dbox}$ in doxycycline (Dox)-inducible cell line used in Figures 4 and S5. Geminin$^{\Delta Dbox}$ was induced with Dox for 6 h and quantified with anti-Geminin antibody (detects endogenous Geminin and Geminin$^{\Delta Dbox}$). n = 147,420 (Dox(-)), 150,706 (Dox(+)) cells.

(D) Same example cells as shown in Figure 4F, co-stained for EdU incorporation, CDT1, and Geminin (detecting both endogenous Geminin and Geminin$^{\Delta Dbox}$). Scale bar = 5 µm. Note, CDT1 levels are likely increased in response to Geminin$^{\Delta Dbox}$ due to co-stabilization. However, CDT1 is inactivated by Geminin, and CDT1 is still degraded over the same 30 min period (Figure S5E).

(E) RT-QIBC aligned to S entry (C-CRL4^{Cdt2} reporter). Same experiment as in Figures 4E-F. Cells that had Dox added ≤ 1 h prior to mitosis were analyzed. Error bars and line are mean ± 2×SEM in bins of cells (Geminin$^{\Delta Dbox}$(-): n = 3,436, Geminin$^{\Delta Dbox}$(+): 2,302 cells total, n ≥ 32 cells per bin). Representative of 3 independent experiments.

(F, G) Calibration of EdU incorporation to absolute DNA synthesis from Figure 4E using RT-QIBC (C-CRL4^{Cdt2} reporter). Absolute DNA synthesis (in base pairs) can be inferred by integrating EdU incorporation measurements (area under the curve), which estimates the total DNA EdU signal. The fraction of total DNA synthesis during a given period (a period 1.5-2 h after S phase entry was chosen as a calibration point. Denoted as $F_{DNA\ 1.5\text{-}2h}$) can be estimated by taking the ratio of the area from 1.5 - 2 h after S phase entry ($Area_{1.5\text{-}2h}$) to the total area ($Area_{Total}$). In the experiment from Figure 4E, a 30 min EdU pulse was used, and thus the EdU intensity in cells 2 h after S phase entry in cells without Dox added would be equivalent to $F_{DNA\ 1.5\text{-}2h}$.

(F) Left: $F_{DNA\ 1.5\text{-}2h}$ was estimated by RT-QIBC of an 8 min EdU pulse at the end of imaging. Median EdU incorporation for each timepoint was used to determine area under the curve (n = 13,626 cells). Middle: Calculation of area under curve. Left: EdU incorporation from a 30 min EdU pulse at the end of imaging. Line and error bars are mean ± 2×SEM in cells within bins. Data pooled from 3 independent experiments (n = 33,208 cells).

(G) The EdU signal in Figure 4E was calibrated based on Figure S5F. Multiplying this by 6×10^9 base pairs (approximate human diploid DNA) gives the equivalent amount of DNA synthesis in base pairs. For each of 3 independent experiments, the median of cells was taken (n ≥ 31 cells per replicate per condition, Geminin$^{\Delta Dbox}$(-) 120 cells total, Geminin$^{\Delta Dbox}$(+) 213 cells total). Error bars are mean ± 2×SEM.

(H) Left: Cyclin E/A-CDK activity reporter is initially nuclear localized in G0 and early G1 and gradually translocates to the cytoplasm in response to Cyclin E/A-CDK activity. Right: 25 sample traces of Cyclin E/A-CDK activity in MCF10A cells aligned to S entry (N-CRL4^{Cdt2} reporter degradation). Thick line is median trace of 87 cells.

(I) MCF10A cells analyzed as in S5H with siRNA knockdown of Cyclin A (siCCNA2). CCNA1 is not expressed in these cells. Line and shaded area are mean ± 2×SEM. n = 129 (siCtrl) or 191 (siCyclin A) cells. Representative of 2 independent experiments.

(J) QIBC of Cyclin A immunofluorescence for validation of Cyclin A siRNA knockdown efficiency in MCF10A cells. n = 27,456 (siCtrl), 22,718 (siCyclin A). Representative of two independent experiments.

(K) RPE-1 *TP53*$^{-/-}$ *CDC6*$^{d/d}$ cells were serum-starved in G0. Cells were then mitogen-released in the presence of Dox, Auxin, and BMS-650032 (BMS) to degrade CDC6 as cells re-enter the cell cycle and inhibit origin licensing, or with vehicle (DMSO) to permit origin licensing. Left: Cells were fixed 12 h after mitogen-release, and QIBC was performed on chromatin-bound MCM2. G0 cells are unreleased cells, and G1 and early S phase cells were chosen based on EdU. n = 2,000 cells for all conditions. Pooled from 2 wells in each condition. Dashed and solid lines in violin plots are IQR and median, respectively. Right: Cells were fixed 15 h after mitogen-release, and QIBC was performed on EdU incorporation. 2N DNA (G1/early S phase cells) were plotted, and dashed line is threshold for EdU incorporation, calculated from G0 cells. n = 12,380 cells (DMSO), 13,543 cells (Dox/Auxin/BMS). Pooled from 2 wells.

(L) QIBC of DNA content in mimosine arrested RPE-1 cells using protocol in Figure 5F, compared to cycling cells. n = 52,433 cells (mimosine arrested) and 61,472 cells (cycling). Representative of 2 independent experiments.

(M) QIBC of mCherry (mChy) fluorescence in RPE-1 cells induced in Figure 5F. Dox(-) cells are uninduced TetOn-NLS-mChy cells to determine background signal. n = 2,000 cells per condition. Dashed and solid lines in violin plots are IQR and median, respectively. Cells with mCherry fluorescence within dashed lines were chosen for analysis in Figure 5H.

Figure S6

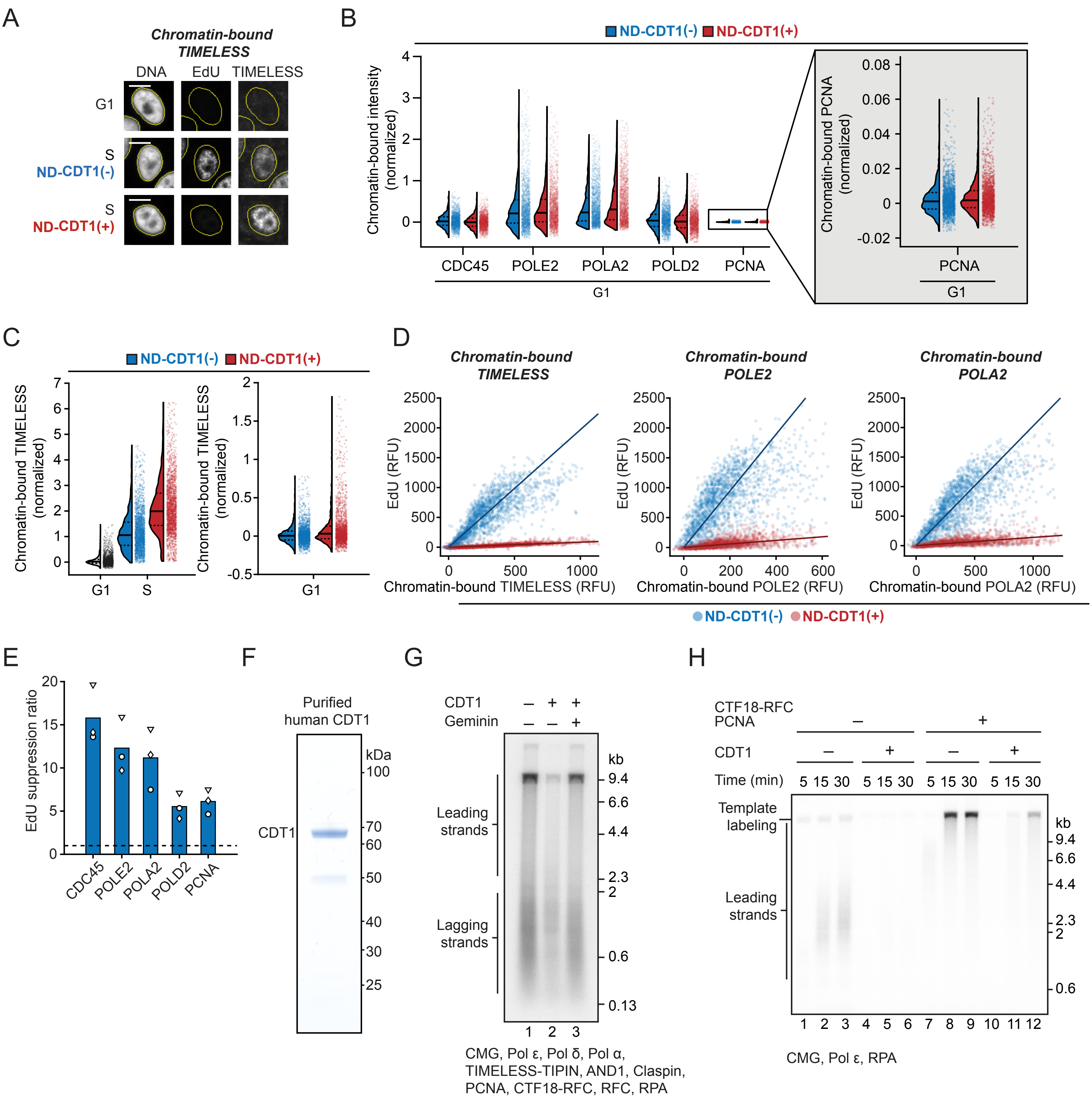
A
Chromatin-bound TIMELESS
DNA
EdU
TIMELESS
G1
S
ND-CDT1(-)
S
ND-CDT1(+)
B
ND-CDT1(-) ND-CDT1(+)
Chromatin-bound intensity (normalized)
CDC45
POLE2
POLA2
POLD2
PCNA
G1
Chromatin-bound PCNA (normalized)
PCNA
G1
C
ND-CDT1(-) ND-CDT1(+)
Chromatin-bound TIMELESS (normalized)
G1
S
Chromatin-bound TIMELESS (normalized)
G1
D
Chromatin-bound TIMELESS
Chromatin-bound POLE2
Chromatin-bound POLA2
EdU (RFU)
Chromatin-bound TIMELESS (RFU)
Chromatin-bound POLE2 (RFU)
Chromatin-bound POLA2 (RFU)
ND-CDT1(-) ND-CDT1(+)
E
EdU suppression ratio
CDC45
POLE2
POLA2
POLD2
PCNA
F
Purified human CDT1
kDa
100
70
60
50
40
30
25
CDT1
G
CDT1
Geminin
kb
9.4
6.6
4.4
2.3
2
0.6
0.13
Leading strands
Lagging strands
1 2 3
CMG, Pol ε, Pol δ, Pol α, TIMELESS-TIPIN, AND1, Claspin, PCNA, CTF18-RFC, RFC, RPA
H
CTF18-RFC
PCNA
CDT1
Time (min)
5 15 30 5 15 30 5 15 30 5 15 30
Template labeling
Leading strands
kb
9.4
6.6
4.4
2.3
2
0.6
1 2 3 4 5 6 7 8 9 10 11 12
CMG, Pol ε, RPA

Figure S6. CDT1 inhibits replication fork elongation while permitting origin firing, related to Figure 6

(A-E) RT-QIBC of chromatin-bound replisome components in mitogen-released MCF10A cells. G1 cells had active APC/C^{Cdh1} without chromatin-bound PCNA. Cells which expressed ND-CDT1-mCherry during live imaging were selected for ND-CDT1(+). Representative of 3 independent experiments.

(A) Representative cells of chromatin-bound TIMELESS. Scale bar = 10 µm.

(B) Comparison of chromatin-bound replisome components in G1 with ND-CDT1, in same experiment as Figure 6B. G1 mode intensities from ND-CDT1(-) were subtracted off signals, and values were normalized to the ND-CDT1(-) S phase condition from Figure 6B. n = 2,000 cells per condition. Representative of 3 individual experiments.

(C) Comparison of chromatin-bound TIMELESS in S phase (left) and G1 phase (right), analyzed in same way as Figure 6B and Figure S6B. n = 2,000 cells, pooled from 3 wells for ND-CDT1(-), or 7 wells for ND-CDT1(+).

(D) Analysis of EdU incorporation as a function of chromatin-bound protein levels for TIMELESS, POLE2, POLA2 in S phase. G1 mode intensities were subtracted off EdU and chromatin-bound intensity. Line is fit line of linear regression (n = 2000 cells). Representative of 3 independent experiments. Other stains in Figure 6D. TIMELESS staining was done in separate experiment as other stains. Cells were pooled from 3 wells for ND-CDT1(-) or 7 wells for ND-CDT1(+) from a single experiment.

(E) Summary of slopes from fit lines from Figures 6D and S6D. EdU suppression ratio is defined as the ratio of the fit line in the control condition to the ND-CDT1 condition (>1 indicates there is lower EdU incorporation for a given amount of chromatin-bound protein). Bar is mean of 3 independent experiments.

(F) Coomassie-stained SDS-PAGE analysis of purified human CDT1 protein.

(G) Denaturing agarose gel analysis of leading/lagging strand replication reactions (9.7 kbp DNA template) for 20 min with the indicated proteins. 10 nM CDT1 was pre-incubated with equimolar Geminin for 5 min on ice before adding to the reactions where indicated. In all reactions, the concentration of potassium glutamate was 250 mM. Experiment was repeated 3 times.

(H) Denaturing agarose gel analysis of a time course experiment performed on the 15.8 kbp forked DNA template with the indicated proteins. CTF18-RFC, PCNA, and CDT1 were included where indicated. CMG-independent template labeling products are indicated. Experiment was repeated two times.

Dashed and solid lines in violin plots are IQR and median, respectively.

Figure S7

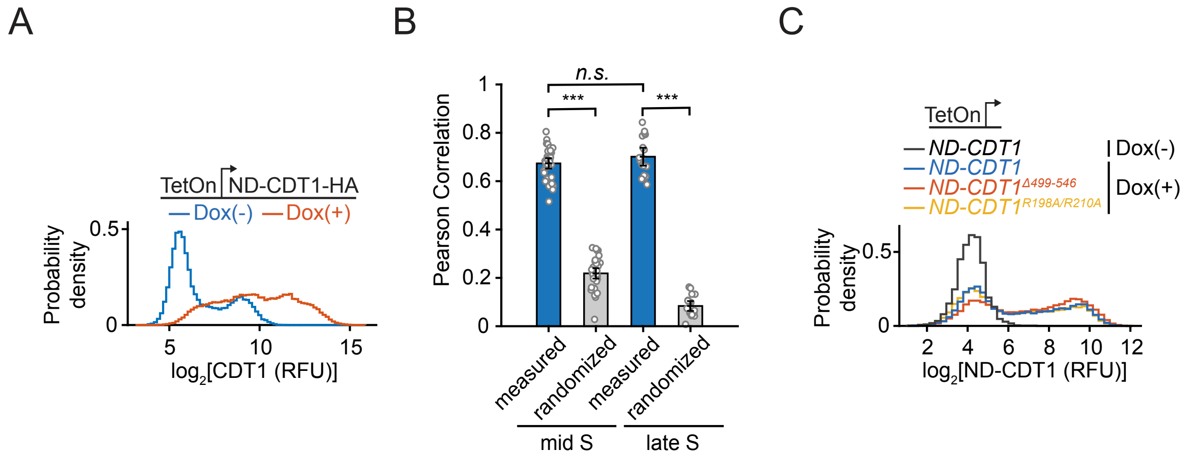

A
TetOn ND-CDT1-HA
Dox(-)
Dox(+)
Probability density
0.5
0
5
10
15
log2[CDT1 (RFU)]
B
n.s.
***
***
Pearson Correlation
1
0.8
0.6
0.4
0.2
0
measured
randomized
measured
randomized
mid S
late S
C
TetOn
ND-CDT1
ND-CDT1
ND-CDT1Δ499-546
ND-CDT1R198A/R210A
Dox(-)
Dox(+)
Probability density
0.5
0
2
4
6
8
10
12
log2[ND-CDT1 (RFU)]

Figure S7. CDT1 inhibits CMG helicase through its MCM-binding domains, related to Figure 7

(A) QIBC of CDT1 immunofluorescence in non-pre extracted U2OS cells overexpressing doxycycline (Dox)-inducible ND-CDT1 as in Figure 7D to compare overexpression levels. n = 56,309 (Dox-), 57,484 (Dox+) cells. Cells were pooled from 6 individual wells per condition.

(B) Quantification of colocalization of ND-CDT1 with chromatin-bound CDC45 from Figure 7D. U2OS cells with endogenously tagged CDC45 and Dox-inducible ND-CDT1 were treated with Dox for 8 h and then pre-extracted and co-stained for ND-CDT1(anti-HA-tag) and CDC45 (anti-GFP) by immunofluorescence. Cells were imaged using SoRa confocal microscopy and staged as either mid or late S phase based on CDC45 pattern. Colocalization of ND-CDT1 and CDC45 were calculated from the Pearson correlation between the two signals within the nucleus. Randomized data was generated from the mean of correlations from images shifted 40 pixels (1.038 µm) up, down, left, and right from each other. n= 35 (mid S) or 18 (late S) cells. Error bars are 2×SEM. Two-sample *t*-test: mid S measured – mid S randomized (*** p-value = 6.9×10^{-41}), late S measured – late S randomized (*** p-value = 1.1×10^{-25}), mid S measured – late S measured (n.s. p-value = .17).

(C) ND-CDT1 expression for ND-CDT1 mutants shown in Figure 7E. Mitogen-released MCF10A cells, treated with siGeminin and induced with Dox. ND-CDT1 was stained using anti-HA antibody. Note that ND-CDT1 expression levels are similar for all mutants. n ≥ 2146 cells for all conditions. Data representative of two independent experiments.
